# Ligated Pd-Catalyzed Aminations of Aryl/Heteroaryl Halides with *Aliphatic* Amines under Sustainable Aqueous Micellar Conditions

**DOI:** 10.1021/jacsau.3c00742

**Published:** 2024-02-12

**Authors:** Karthik S. Iyer, Rahul D. Kavthe, Robert M. Lammert, Jordan R. Yirak, Bruce H. Lipshutz

**Affiliations:** Department of Chemistry and Biochemistry, University of California, Santa Barbara, California 93106, United States

**Keywords:** aminations, aliphatic amines, BippyPhos, pharmaceutical diversification, API synthesis, micellar catalysis

## Abstract

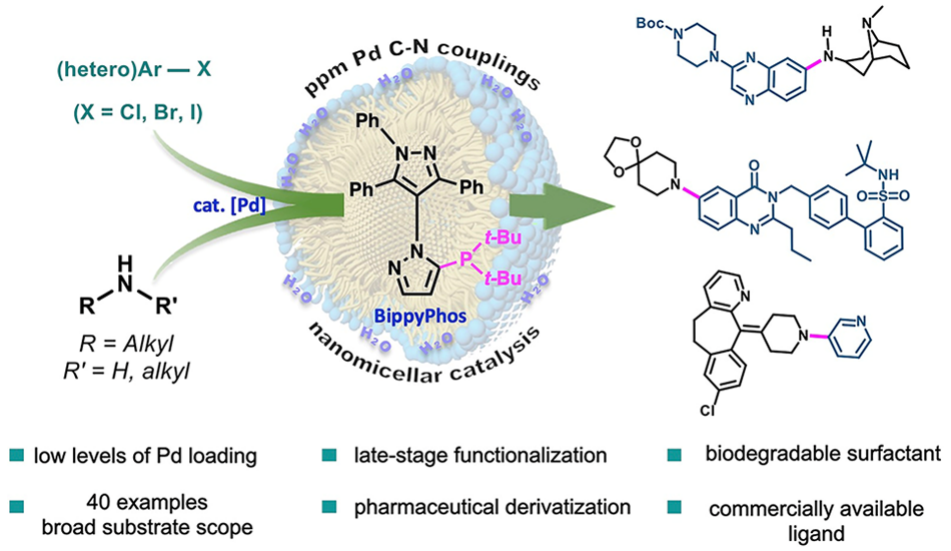

Sustainable technology for constructing Pd-catalyzed C–N bonds involving aliphatic amines is reported. A catalytic system that relies on *low levels of recyclable precious metal*, a known and *commercially available ligand*, and a *recyclable aqueous medium* are combined, leading to a newly developed procedure. This new technology can be used in ocean water with equal effectiveness. Applications involving highly challenging reaction partners constituting late-stage functionalization are documented, as is a short but efficient synthesis of the drug naftopidil. Comparisons with existing aminations highlight the many advances being offered.

## Introduction

Palladium-catalyzed aminations of aryl/heteroaryl halides and pseudohalides are indispensable tools in synthesis, finding particularly extensive use in the synthesis of pharmaceuticals,^1−4^ natural products,^1,5−7^ and various materials.^8−10^ Given the ubiquitous nature of aliphatic amines in compounds of synthetic interest and the lack of alternative methodologies for their synthesis that are both mild and generally applicable, Pd-catalyzed aminations remain an important methodology in both academia and industrial laboratories.^11^ However, with the changing times, sustainability concerns associated with these reactions have spurred the need for methods that are both versatile and economical. Perhaps equally important have become the principles of green chemistry^12^ that focus on safety, conservation of planetary resources, and the impact that C–N bond formations have on the environment.

Within the landscape of metal-catalyzed aminations featuring numerous methodologies, various approaches including group 10 transition metal-catalyzed aryl and heteroaryl aminations^13,14^ and to a lesser extent Cu-catalyzed Ullmann couplings^15−18^ have garnered extensive recognition for their applicability as well as their ability to accommodate a diverse array of functional groups.^19,20^ Considerable progress has also been made of late insofar as Chan–Evans–Lam couplings are concerned,^21^ along with catalysts developed by Ma and co-workers^22^ that have allowed a significant reduction in catalyst loadings and reaction temperatures.

Notable examples of effective ligands for aminations include various dialkylbiarylphosphanes^23,24^ and palladacycles incorporating these ligands.^25−33^ Additionally, *N*-heterocyclic carbene (NHC)-based precatalysts^26,29,34^ have also shown promising activity in the coupling of secondary amines with unfunctionalized aryl halides. Notwithstanding their demonstrated potential, the applicability of most methods has generally been limited to relatively simple amines and coupling partners. Moreover, all are conducted in organic solvents, with some solvents being already prohibited,^35^ and that additional significant drawbacks including generation of organic waste raise environmental concerns as it contributes to the depletion of our limited planetary petroleum resources. Furthermore, combustion of organic solvents leads to the generation of extensive levels of carbon dioxide being released into the atmosphere, thereby exacerbating climate change.

Adding to these concerns, amination reactions as practiced over the past 25 years^36^ have required palladium catalysts used at loadings typically ranging from 1 to 10 mol %, which is both costly and consumes finite platinum group metal reserves. Moreover, rarely is the level of residual metal to be found in each coupled product evaluated when catalysts are utilized at these levels. Given the intricate interplay of these circumstances, it is becoming more obvious that alternative processes that are environmentally respectful (i.e., sustainable) are needed to affect *traditional* palladium-catalyzed aminations so vital to the fine chemicals industry in general, and the pharmaceutical industry, in particular.

**Scheme 1:**
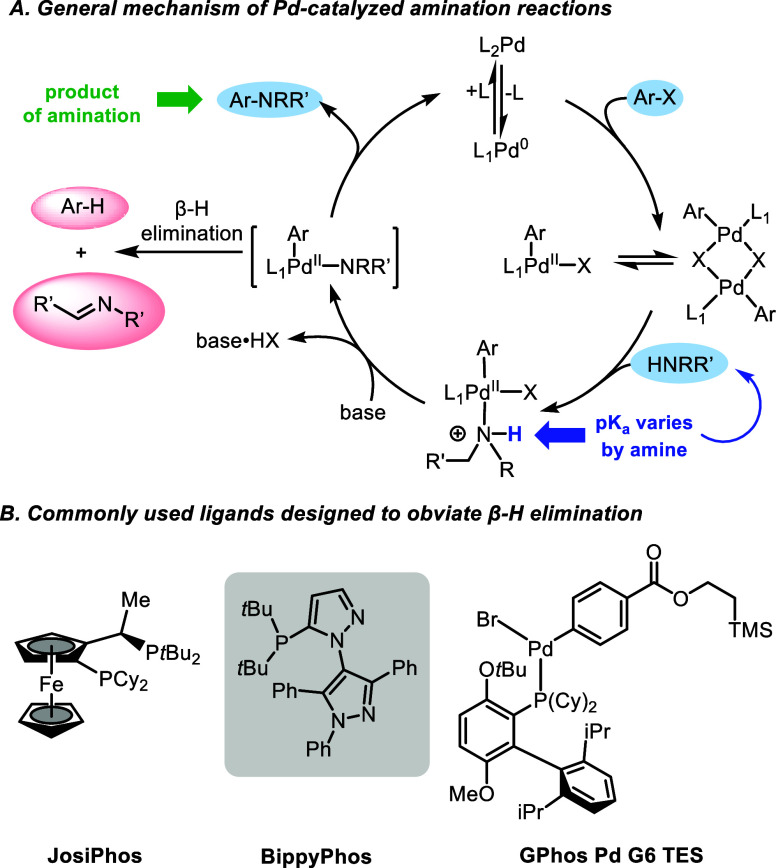
Various Aspects Associated with Pd-Catalyzed Aminations

The mechanism of Pd-catalyzed C–N cross-coupling reactions is well documented (Scheme 1A).^37−43^ In cases involving primary aliphatic amines, the reaction can follow an undesired pathway, resulting in β-hydride elimination of a Pd amide species, ultimately affording the corresponding hydrodehalogenated arene. Efforts to minimize this undesired outcome have led to the development of numerous precatalysts,^25−33^ such as sterically demanding bidentate phosphine (JosiPhos) ligands^14^ and bipyrazole-based bulky monodentate phosphine ligands (e.g., BippyPhos; Scheme 1B).^44,45^ Recently, oxidative addition complexes have been introduced that exhibit noteworthy reactivity across a wide spectrum of aryl and pseudohalide electrophiles, including those featuring base-sensitive 5-membered heterocycles.^46−48^ In a big picture sense, however, obtaining catalysts in the GPhos series containing oxidative addition complexes entails a series of intricate and resource-intensive synthetic steps. This complexity is manifested in their elevated costs, and thus far, such species are not readily available and hence pose logistical challenges for widespread adoption.^47^ Furthermore, they have been designed for use exclusively in organic solvents, without recycling or any other metrics associated with environmental considerations in mind (e.g., E Factors^49−51^ or process mass intensity (PMI)^52,53^ values). These parameters, yet again, highlight the pressing need for an alternative, far more environmentally attractive, and sustainable process for aminations.

**Scheme 2:**
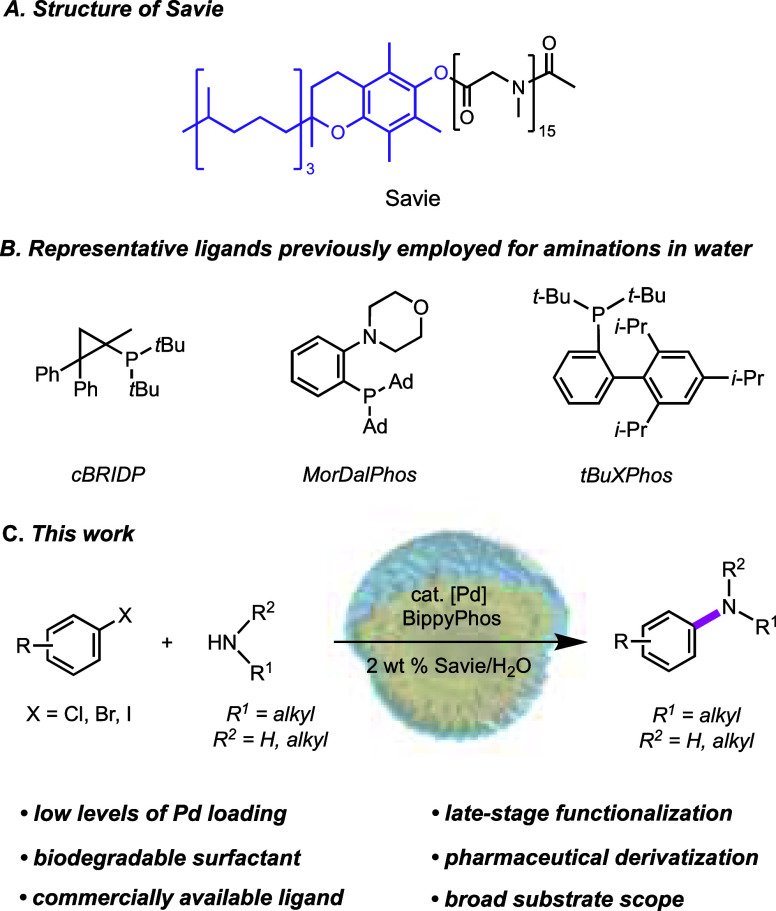
Efforts To Develop a More Sustainable Amination Protocol

One potential solution for aminations involving homogeneous catalysis employing aliphatic amines employs micellar catalysis,^54−58^ where the presence of a designer surfactant leads to nanomicelles that function as nanoreactors in which water-insoluble coupling partners and catalyst interact, leading to the desired product under both aqueous and mild conditions. Our most recent amphiphile is Savie,^59^ in which the MPEG hydrophilic portion (as in TPGS-750-M) is replaced with a polypeptoid derived from polysarcosine (Scheme 2A).^59,60^ We, and others, have previously identified ligand scaffolds that complex Pd forming catalysts that mediate aminations under micellar conditions in water (Scheme 2B).^61−67^ However, the limited substrate scope and lack of late-stage functionalization may hinder applications of these methods at scale. Hence, we have developed a new protocol that (1) maintains or reduces the loadings of the Pd catalyst; (2) utilizes a commercially available ligand; (3) can be run in water and even ocean water; (4) is applicable to a large array of functionalized halides; (5) leads to products that contain low levels of residual Pd, thereby avoiding additional time and effort to meet FDA standards; (6) allows for recycling of the reaction medium and catalyst; and (7) can be used as part of a telescoped sequence en route to a known pharmaceutical, all being done in a single pot operation (Scheme 2C).

## Results and Discussion

### Optimization of Aminations between Aromatic Bromides and *Aliphatic* Amines

Amination with aliphatic amines is oftentimes highly sensitive to the nature of the ligand involved,^68,69^ where it has been designed to (1) promote the formation of a monoligated Pd(0) complex; (2) activate palladium toward oxidative addition; (3) provide steric protection to the coordination sphere of the ligated palladium as a means of promoting selectivity; and (4) encourage C–N bond formation *via* facilitated reductive elimination. These features may necessitate that the ligand be both electron-rich and sterically demanding. The coupling between 1-bromo-3-(methylsulfonyl)benzene (**1a**) and 3,4-dimethoxyphenethylamine (**1b**) to afford **1** (Table 1) was specifically chosen as a representative model reaction to test the potential for a primary aliphatic amine to undergo the desired cross coupling, versus β-hydride elimination to the undesired imine along with the hydrodehalogenated arene. Since many known aryl aminations utilize alkoxide bases,^70−73^ potassium *t*-butoxide (KO*t*Bu) was initially selected for this purpose.

**Table 1:**
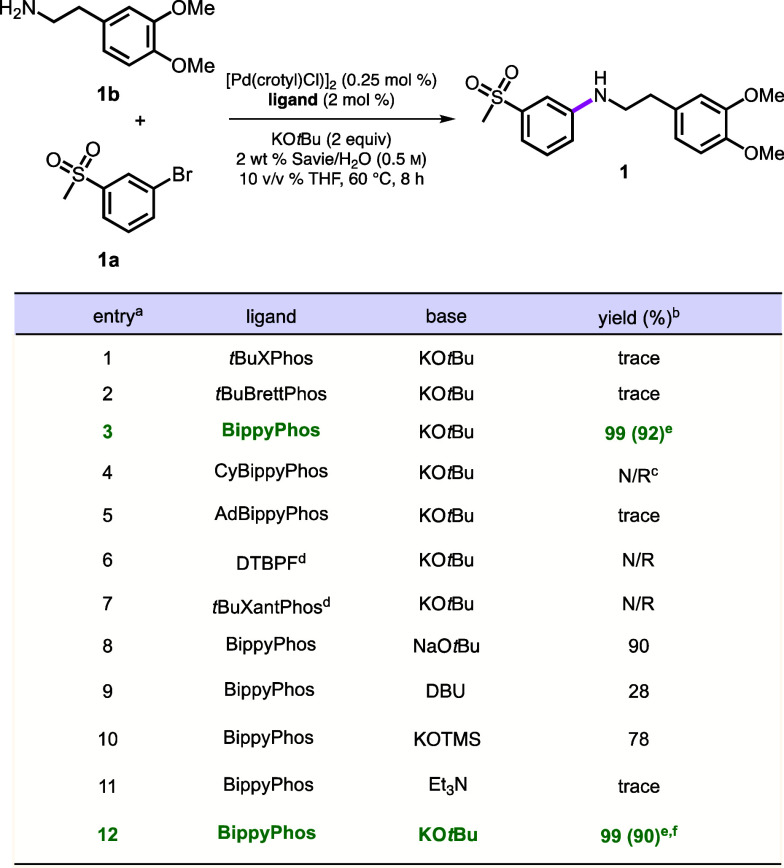
Screening of Reaction Conditions for the Coupling of Aliphatic Amines^a^^,^^b^^,^^c^^,^^d^^,^^e^^,^^f^

a Reactions were carried our at 0.25 mL scale.

b NMR yields using 1,3,5,trimethoxybenzene as internal standard.

c N/R = no reaction.

d Reaction was run at 70 °C for 24 h.

e Isolated yield.

f Reaction was run for 1.5 h.

With the Pd loading maintained at 0.5 mol % (i.e., 0.25 mol % of the dimer; Table 1), screening for the optimal ligand was carried out for this coupling under aqueous micellar conditions. To circumvent β-hydride elimination, bidentate ligands such as 1,1’-bis(di-*t-*butylphosphino)ferrocene (D*t*BPF) and *t*BuXantphos were evaluated (entries 6, 7) but without success. The Buchwald ligand, *t*BuBrettPhos, known to efficiently catalyze aminations of aryl (pseudo) halides with 1° amines^74^ afforded only trace amounts of **1** (entry 2). Only BippyPhos^44,45^ shown by Stradiotto et al. to couple aryl/heteroaryl *chlorides* with relatively simple amine nucleophiles in organic solvent proved to be effective (92% isolated yield, entry 3). An investigation of bases indicated that KO*t*Bu performed the best (entries 9–12, also see SI, Table S2). Alternatively, use of KOH and *t*-BuOH (2 equiv) afforded identical results, indicative of the equilibration taking place in water.^76^ Unfortunately, lowering the Pd loading to 0.1 mol % of the dimer significantly reduced the yields (see SI, Table S3). Reducing the reaction time to 1.5 h from 8 h afforded **1** reproducibly in 90% isolated yield (entry 12). Optimized conditions for aminations of aryl halides, therefore, were determined to be Colacot’s pre-catalyst: [Pd(crotyl)Cl]_2_ (0.25 mol %);^75^ ligand: BippyPhos (2 mol %); base: KO*t*Bu (2 equiv); and reaction medium: 2 wt % Savie/H_2_O, at 60 °C.

### Representative Examples of C–N Couplings

**Scheme 3:**
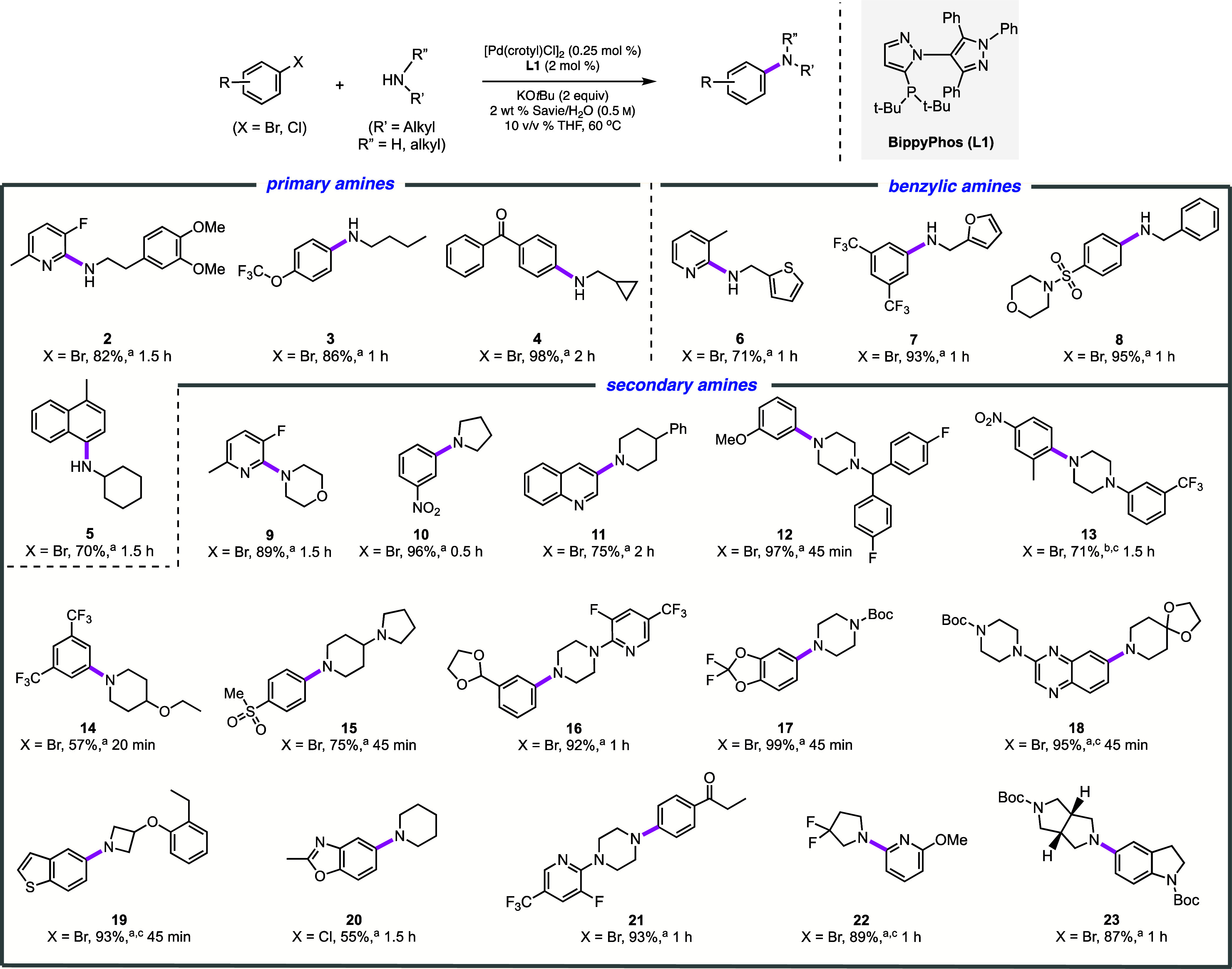
Representative Examples of *Aliphatic* Amines Used in Pd-Catalyzed C–N Bond Formation Aryl halide (0.25 mmol), R”NHR’ (0.375 mmol), [Pd(crotyl)Cl]_2_ (0.5 mol % Pd; 0.25 mol % of the dimer), BippyPhos (2 mol %), KO*t*Bu (0.5 mmol), 2 wt % Savie/H_2_O (0.45 mL), THF (0.05 mL), 60 °C. [Pd] (0.75 mol %, 0.375 mol % dimer). HCl salt of the amine was used.

The scope of the C–N bond construction using the combination of [Pd(crotyl)Cl]_2_ and BippyPhos was examined under aqueous micellar conditions. As illustrated in Scheme 3, a variety of aryl/heteroaryl bromides undergo amination with an array of *aliphatic* amines to provide the desired aminated products. Primary aliphatic amines, including 3,4-dimethoxyphenethylamine, *n-*butylamine, cyclohexylamine, and cyclopropylmethylamine, all participated in couplings with various aryl/heteroaryl bromides in good-to-excellent isolated yields. Moreover, complete selectivity for monoarylation products (**2**–**5**) was seen. Likewise, benzylic amines reacted efficiently, affording the desired arylamines in good yields (products **6**–**8**). Cyclic amines of varying sizes and nucleophilicity, being among the most prevalent nitrogen-containing heterocycles in FDA-approved drugs,^1^ include products containing four- (**19**), five- (**10, 22**, and **23**), and six-membered rings (**9, 11–18, 20, 21**). It is also noteworthy that in addition to *N-*containing heterocycles, other heteroatom-containing heterocycles, such as 1,1’-difluorobenzodioxole (leading to product **17**) and benzothiophene (affording **19**), were amenable to these coupling conditions. In general, most amines are water-insoluble, whether primary of secondary, since, as examined previously under very different conditions and in only a few cases,^66^ those that are water-soluble can be quite challenging (see products **9**, **10**, and **20**).

### C–N Bond Formation in Ocean Water

**Scheme 4:**
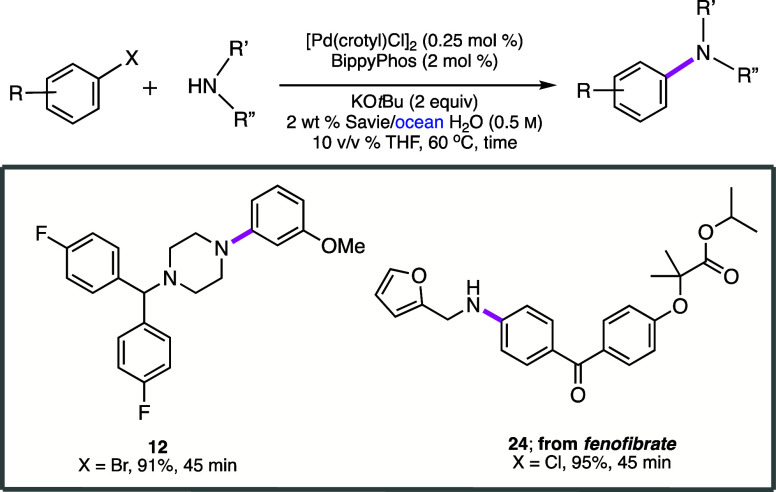
Comparisons of Reactions Run in Ocean Water

The remarkable impact of added salts on the properties of aqueous micelles has previously been evaluated in terms of their effects on *selected Pd-catalyzed C–C bond forming cross-coupling reactions*.^77^ Thus, the potential for using ocean water for aminations,^76^ rather than more expensive and potentially less readily available purified water, was briefly examined (Scheme 4). Two amination reactions led to products **12** and **24** in excellent isolated yields. Hence, the prospects for carrying out C–N bond formation under such conditions look encouraging, although these are likely to be done early in a sequence where ocean-derived impurities can be removed and the cost of the reaction medium may be a factor.

### Recycling Studies

**Scheme 5:**
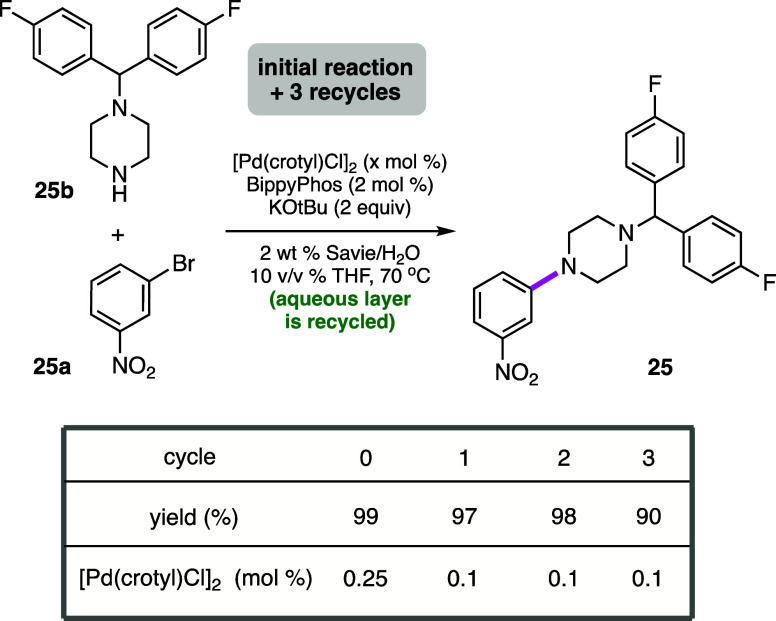
Recycling Studies

A commonly utilized metric for rapidly assessing the environmental friendliness of a reaction is Sheldon’s E Factor,^49−51^ which has stood the test of time although others are certainly becoming more prevalent, such as PMI^52,53^ and especially a life cycle assessment (LCA).^78,79^ The latter, however, is far more accessible within industrial circles. Recycling of an aqueous reaction mixture can have a significant impact on these values, highlighting the major advantage of conducting various processes in water. Thus, following an initial reaction between 1-bromo-3-nitrobenzene **25a** and a functionalized piperazine **25b** (a precursor to the calcium channel blocker Flunarizine (Sibelium; Scheme 5), the desired product can be readily isolated via an in-flask extraction with recyclable amounts of MTBE (see SI, section 5). Reuse of the aqueous layer remaining *in the same vessel* for three additional cycles led to excellent results in terms of product formation and isolation. Only additional catalyst, ligand, base, and starting materials need to be added, preferably under an inert atmosphere, after each coupling. Overall, therefore, these four reactions involved a total investment of only 1.1 mol % Pd or *0.275 mol % per amination*. After the fourth reaction (i.e., the third recycle), salt buildup leading to increased viscosity precluded additional recycling. Associated E Factors were calculated to be only 1.51 (when recyclable MTBE is not considered waste; see SI, section 5) and 10.5 (when MTBE is considered waste). ICP-MS analysis of a product isolated from amination after standard workup and purification showed a residual metal level of 0.78 ppm Pd, unlike levels to be expected for aminations run with far higher loadings of Pd in organic solvents.^36^*Since values on this order are far below those allowed by the FDA (10 ppm Pd per dose per day)*,^80^*no additional processing to remove residual Pd is needed, which can otherwise be costly and time-consuming*.

### Late-Stage Aminations with Pharmaceutically Relevant Substrates

**Scheme 6:**
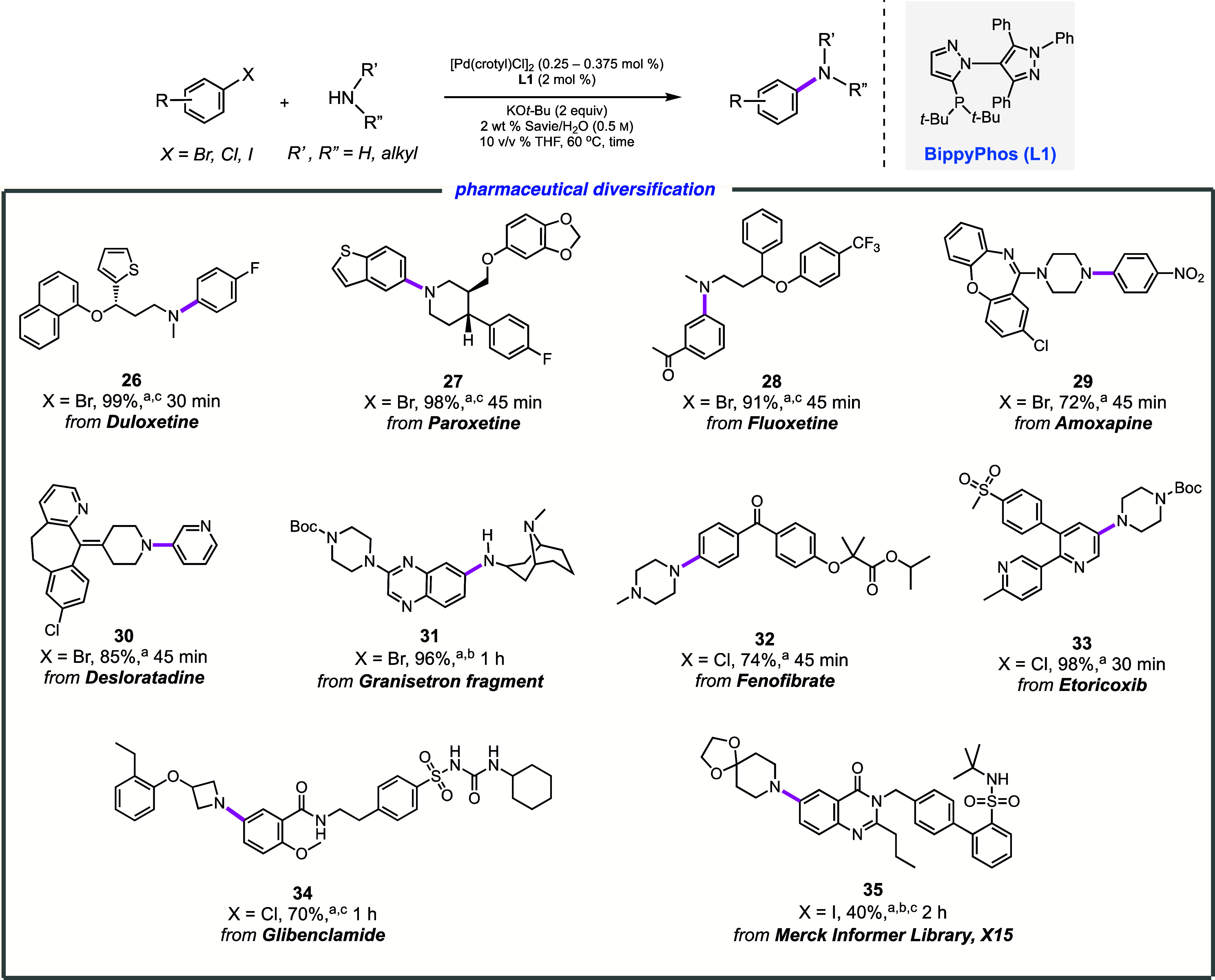
Representative Examples of Late-Stage C–N Bond Formation Aryl halide (0.25 mmol), R″NHR′ (0.375 mmol), [Pd(crotyl)Cl]_2_ (0.25 mol %; 0.5 mol % [Pd]), BippyPhos (2 mol %), KO*t*Bu (0.5 mmol), 2 wt % Savie/H_2_O (0.45 mL), THF (0.05 mL), 70 °C. [Pd(crotyl)Cl]_2_ (0.375 mol %, 0.75 mol % [Pd]). HCl salt of the amine was used.

Both medicinal and process chemistry place significant value on Pd-catalyzed C–N coupling reactions,^81−86^ given the large number of nitrogen-containing biologically active compounds. Several structurally challenging, free-amine-containing pharmaceuticals were coupled with aryl or heteroaryl halides, including complex cases selected from the Merck Informer library (e.g., product **35**; Scheme 6)^87^ in efforts to examine the true limits of this new technology. Highly functionalized reaction products, including derivatives of antidepressants such as Duloxetine (**26**; Cymbalta), Paroxetine (**27**; Paxil), Fluoxetine (**28**; Prozac), and Amoxapine (**29**; Asendin), were formed in good-to-excellent yields. Additionally, *N*-arylation of (i) Desloratadine (Clarinex), an antihistamine medication (leading to product **30**); (ii) a bicyclic 2° amine precursor to the antiemetic drug Granisetron (Sancuso; affording product **31**); (iii) Fenofibrate (Lipofen), which which is an FDA-approved drug for the treatment of hypertriglyceridemia and hypercholesterolemia (leading to **32**); (iv) Etoricoxib (Arcoxia), a selective COX-2 inhibitor for the treatment of osteoarthritis (giving product **33**), and (v) the highly functionalized aryl chloride Glibenclamide (Diabeta) used for the treatment of type 2 diabetes (producing product **34**); all gave the anticipated aminated derivatives. Particularly noteworthy is the rate of these aminations, a key factor especially for those in pharma, which in most cases requires only an hour or less notwithstanding relatively low catalyst loadings. Collectively, C–N couplings of this nature involving complex pharmaceuticals used under environmentally responsible conditions further establish generality but also attest to the additional elements of sustainability involved, as a new and important tool based on chemistry in water can now be added to the growing toolbox.

### Comparison Aminations with Existing Literature

**Scheme 7:**
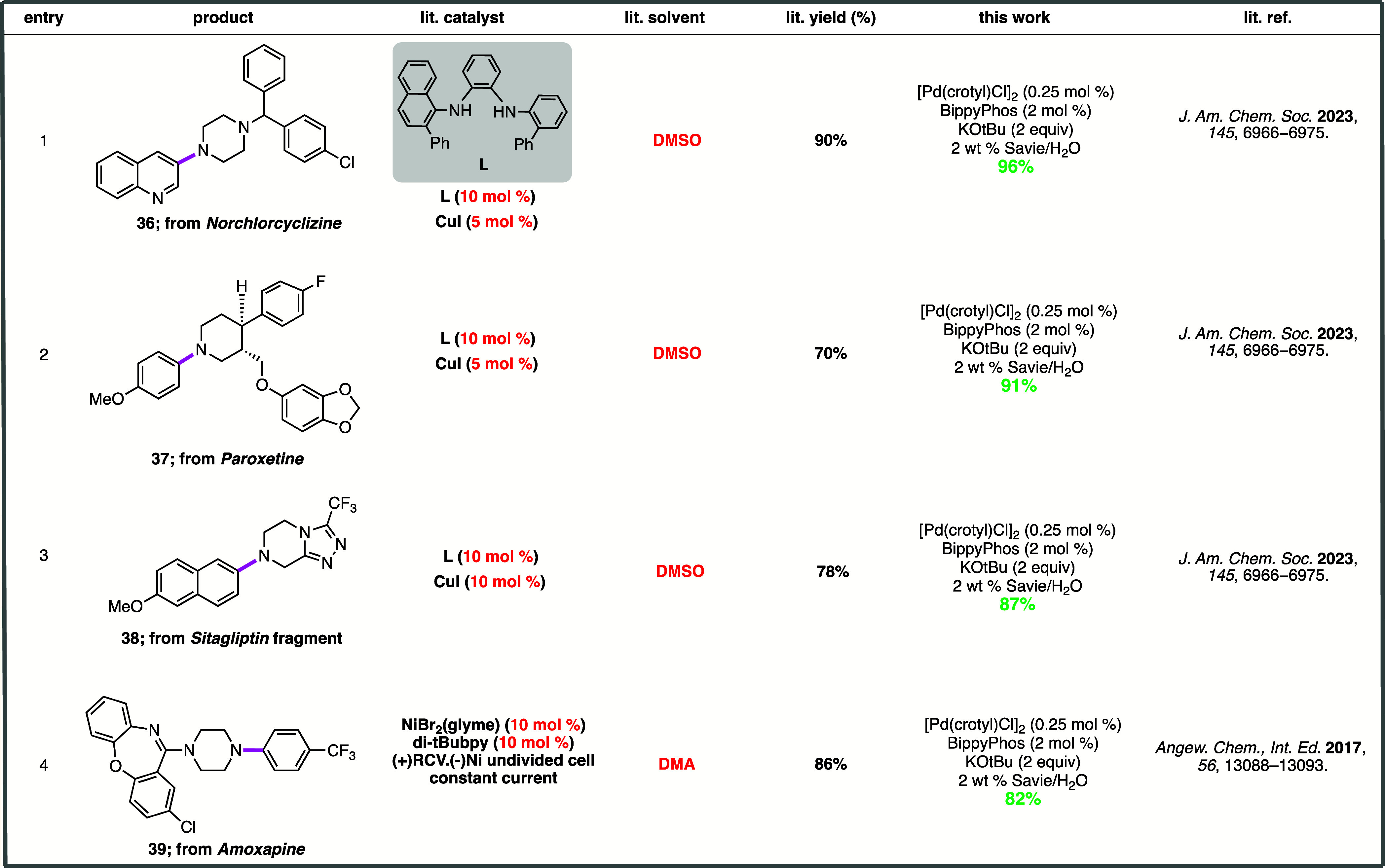
Comparisons with Recent Examples of Late-Stage C–N Bond Constructions

Direct comparisons with state-of-the-art procedures were also made, focusing for the most part on the realization of intermediates associated with pharmaceutically relevant compounds (Scheme 7).^18,88^ Aminations arriving at products **36**–**39** indicate that by using this catalytic aqueous micellar system (i.e., [Pd(crotyl)Cl]_2_–BippyPhos), enhanced rates of couplings versus the corresponding reactions in organic solvents are to be expected. Moreover, yields tend to be comparable to, if not higher than, those reported previously. Lastly, and from the perspective of sheer convenience, the commercial availability of the Pd dimer and associated ligands that avoid pre-catalyst formation^89^ suggests that this new catalytic combination offers several advantages to the practitioner previously unavailable.

### Synthesis of Naftopidil

**Scheme 8:**
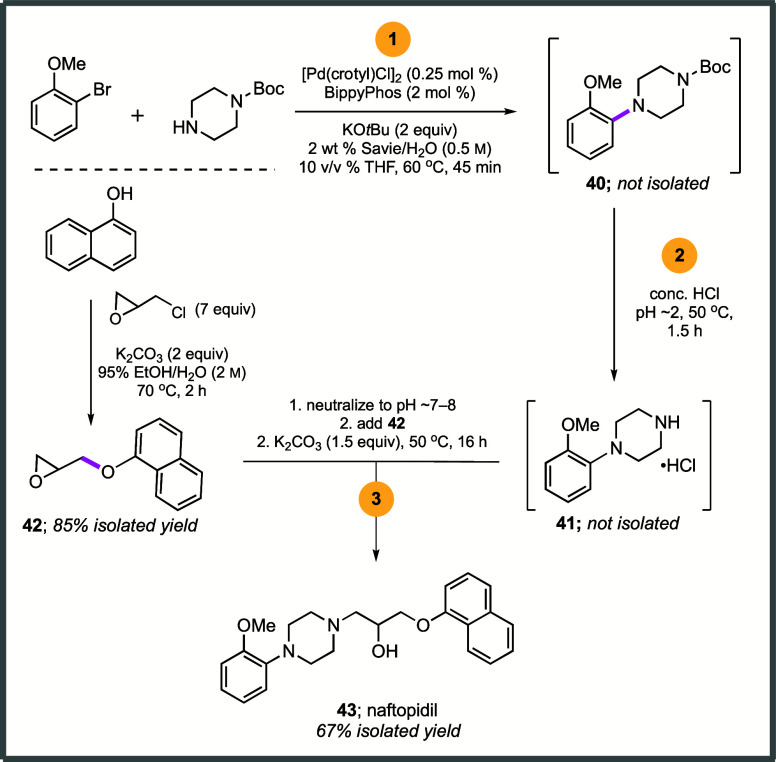
Compounds Formed En Route to Naftopidil

To further illustrate the potential of this technology, several effective and greener syntheses of naftopidil (**43**; Flivas), a selective α_1-_adrenergic receptor antagonist used in the treatment of benign prostatic hypertrophy, were undertaken.^90^ One route shown in Scheme 8 (see SI, section 6 for optimization) calls for an initial amination of 2-bromoanisole with *N-*Boc piperazine leading to the coupled product **40**,^91^ which can be isolated in 89% yield (see SI, section 6). Its *N*-Boc deprotection was accomplished using HCl giving isolable (of desired) amine **41** as the HCl salt.

Alternatively, a streamlined 2-step, *one-pot sequence* was also developed where, following initial amination to **40**, after which lowering the pH to ∼2 with conc. aq. HCl, intermediate **41** could be generated in freebase form and isolated in 86% overall yield (see SI, section 6.3). In parallel, epoxide **42** was prepared in 85% yield using 1-naphthol and epichlorohydrin in 95% EtOH. Epoxide opening by intermediate secondary amine **41** under aqueous micellar conditions afforded naftopidil (**43**) in 83% isolated yield (see SI, section 6.5). However, and perhaps even more attractive, is a 3-step, one-pot sequence (considering the longest linear sequence), starting with a Pd-catalyzed amination to give intermediate **40**, which was used without isolation for its *N-*Boc deprotection to afford **41**. Again, without isolation, epoxide opening by secondary amine **41** ultimately afforded racemic naftopidil (Flivas; **43**) in 67% isolated overall yield. ICP–MS analysis of this material revealed no detectable levels of Pd, while the FDA-approved limit is 10 ppm per dose per day.^47^

## Conclusions

In summary, a protocol has been developed for aminations of aryl halides in water, employing homogeneous catalysis that utilizes sustainable loadings of precious metal. Thus, in addition to several aspects associated with sustainability, this work brings to the chemistry community a number of novel and timely advances, including the following:

- the application of BippyPhos for C–N bond formation employing *aliphatic* amines in an aqueous micellar medium;
- reliance on only commercially available catalyst precursors;
- use of Pd at low catalyst loadings in recyclable water in multiple steps within a sequence, thereby documenting a rare case of “metal economy”;
- reactions that can be in ocean water, addressing potential limitations due to availability of fresh water;
- applications to aminations of structurally diverse aryl and heteroaryl halides together with *aliphatic amines*, which include highly functionalized, complex pharmaceuticals, and related species.

Furthermore, aminations via continuous flow have also been carried out successfully not only using aliphatic amines but also with aromatic amines on aromatic/heteroaromatic halides.^92^

## Experimental Procedures

### Preparation of a Stock Solution of [Pd(crotyl)Cl]_2_ in THF

To a 1 dram vial with a PTFE-coated magnetic stir bar was added [Pd(crotyl)Cl]_2_ (5 mg, 0.012 mmol). The vial was sealed with a rubber septum and evacuated and backfilled with argon three times using an argon/vacuum manifold. This was followed by the addition of anhydrous THF (obtained from a solvent purification system; 1 mL). The vial was stirred gently at rt until [Pd(crotyl)Cl]_2_ dissolved completely. For a 0.25 mmol scale reaction, 50 μL of the stock solution contained 0.25 mol % of the dimer (0.5 mol % Pd), which was used directly for further optimization.

### General Procedure for the Coupling of Aryl Halides with Aliphatic Amines

To a 1 dram vial equipped with a PTF-coated magnetic stir bar was added the aryl halide (1 equiv, 0.25 mmol, if solid), followed by the addition of the amine (0.375 mmol, 1.5 equiv, if solid). The vial was sealed with a rubber septum, evacuated, and backfilled with argon three times using an argon/vacuum manifold and then taken into an argon-filled glovebox, where BippyPhos (2 mol %, 2.5 mg) and KO*t*Bu (2 equiv, 56 mg) were then added. The vial was taken out of the glovebox, and aryl bromide was added (if liquid), followed by the addition of the amine (if liquid) under an atmosphere of argon. Subsequently, a solution of 2 wt % Savie/H_2_O (0.45 mL) was added, followed by the addition of [Pd(crotyl)Cl]_2_ (0.5–0.75 mol % Pd, 0.25–0.375 mol % of dimer) as a stock solution in THF (50 μL, see SI, section 2). The reaction mixture was allowed to stir at 60 °C for the designated amount of time. Upon completion (as monitored by TLC), the reaction mixture was extracted with EtOAc (4 × 1 mL). The combined extracts were dried over anhydrous Na_2_SO_4_, filtered, concentrated in vacuo, and subjected to flash chromatography using the desired eluent (EtOAc/hexanes or MeOH/CH_2_Cl_2_, see analytical section for choice of eluents) to obtain the desired coupled products.

## Supporting Information Available

Experimental procedures, optimization details, and analytical data of isolated materials (NMR, HRMS) (PDF)
