# Supplementary material for: Ligated Pd-Catalyzed Aminations of Aryl/Heteroaryl Halides with Aliphatic Amines under Sustainable Aqueous Micellar Conditions
Source: JACS Au. 2024 Feb 12;4(2):680–9. doi: 10.1021/jacsau.3c00742 (PMC10900223; doi:10.1021/jacsau.3c00742)
Supplement: Supplementary file 1 — au3c00742_si_001.pdf [file au3c00742_si_001.pdf]

Supporting Information

Ligated Pd–catalyzed aminations of aryl/heteroaryl halides with *aliphatic amines* under sustainable aqueous micellar conditions

Karthik S. Iyer, Rahul D. Kavthe, Robert M. Lammert Jr., Jordan R. Yirak, and Bruce H. Lipshutz*

Department of Chemistry and Biochemistry, University of California, Santa Barbara, CA 93106 USA

Phone: 805–893–2521
Fax: 805–893–8265
Email: lipshutz@chem.ucsb.edu
Website: https://lipshutz.chem.ucsb.edu/

Table of Contents

1. General Information S3

2. Optimization of reaction conditions S5

2.1. Ligand screen S5

2.2. Screening of bases S8

2.3. Catalyst loading screen S8

2.4. Additional experiments S9

3. General procedure for the coupling of aryl halides with aliphatic amines: Procedure 1 .... S9

4. General procedure for aminations using ocean water: Procedure 2 .... S10

4.1. Preparation of a 2 wt % solution of Savie in ocean water S10

4.2. Procedure for aminations using ocean water S10

5. Recycling studies and E Factor calculations S12

5.1. Recycling studies S12

5.2. E Factor calculations S14

6. Synthesis of naftopidil S15

6.1. General procedure for amination to afford intermediate 40 .... S15

6.2. Optimization of *N*–Boc deprotection of intermediate 40 .... S16

6.3. 2-Step, one-pot sequence to afford intermediate 41b (free base) S17

6.4. **Synthesis of intermediate 42** **S18**

6.5. **Synthesis of naftopidil** **S19**

6.6. **3-Step, one pot synthesis of naftopidil – Procedure 3** **S19**

7. ICP–MS analysis for residual palladium *S21*

8. References *S21*

9. Analytical data *S22*

9.1. **Analytical data for starting materials and intermediates** **S22**

9.2. **Analytical data for products** **S23**

10. NMR spectra of intermediates and products *S50*

1. General Information

Safety Statement:

No unexpected or unusual safety hazards were encountered. However, areas where extra safety precautions were taken have been explicitly noted.

Reagents:

All commercially available reagents were used without further purification with the exception of N–Boc–Sar (Sar = sarcosine) which was purified by hot filtration with EtOAc or *i*–PrOAc followed by recrystallization from hot EtOAc or *i*–PrOAc. Reagents were purchased from Sigma–Aldrich, Combi–Blocks, Alfa Aesar, Acros Organics, A2B chemicals, BLD Pharma, or AK Scientific, unless otherwise noted.

Surfactant Solution Preparation:

A 2 wt % TPGS–750–M / H_2O solution was prepared by dissolving TPGS–750–M (from PHT International) in degassed HPLC grade water; likewise, 2 wt % aqueous solutions of Savie, Kolliphor ES, Triton X, MC–1 and PTS 600 were prepared in the same manner. TPGS–750–M was made as described previously[1] and is also commercially available from Sigma–Aldrich (catalog #733857 (solution) or #763896 (wax)), although the surfactant used in this study was supplied by PHT and is of consistent high quality. Savie was prepared as described previously[2] and will soon be available from Sigma–Aldrich (catalog #926981) and in larger quantities from PHT International. HPLC–grade water was obtained from Sigma Aldrich and Fischer Scientific and was purged with argon before use. Seawater was obtained from the Pacific Ocean, from the beach associated with UCSB.

Chromatography:

Silica gel TLC plates (UV 254 indicator, thickness 200 mm standard grade, glass backed and 230–400 mesh from Merck, and Silicycle) were used. The developed TLC plate was analyzed with a UV lamp (254 nm). The plates were further analyzed with use of an aqueous ceric ammonium molybdate stain, potassium permanganate stain, or ethanolic vanillin and developed with a heat gun. Flash chromatography was performed using Silicycle Silicaflash® P60 unbonded grade silica.

Nuclear Magnetic Resonance Spectroscopy (NMR):

^{1}H, ^{13}C, and ^{19}F NMR were recorded at 25 ºC on an Agilent Technologies 400 MHz, a Bruker Avance III HD 400 MHz, a Bruker Avance NEO 500 MHz, a Varian Unity Inova 500 MHz, or a Varian Unity Inova 600 MHz spectrometer in $CDCl_3$ or DMSO–d_6 with residual $CHCl_3$ (^{1}H = 7.26 ppm, ^{13}C = 77.16 ppm) or DMSO (^{1}H = 2.54 ppm, ^{13}C = 40.45 ppm) as the internal standard. Deuterated solvents were purchased

from Cambridge Isotope Laboratories. Chemical shifts are reported in parts per million (ppm). The datapresented will be reported as follows; chemical shift, multiplicity (s = singlet, bs = broad singlet, d = doublet, dd = doublet of doublet, t = triplet, q = quartet, quin = quintet, m = multiplet), coupling constant (if applicable), and integration.

Mass Spectrometry (MS):

HRMS analyses (ESI–MS, CI–MS, or GC–EI) were performed by the UC Santa Barbara mass spectrometry facility or the UC Irvine mass spectrometry facility. ICP–MS analysis was performed at the California NanoSystems Institute (CNSI), located at UCLA. ESI–MS analysis was performed on a Waters LCT Premier mass spectrometer equipped with an Alliance 2695 Separations module. EI–MS analysis was performed on a Waters GCT Premier mass spectrometer equipped with an Agilent 7890A GC oven and J&W Scientific DB–5ms+DG narrow bore column using helium carrier gas.

Inductively coupled plasma mass spectrometry (ICP–MS, NexION 2000, PerkinElmer) analysis was performed to detect palladium in powder samples. All samples were used as received without further purification or modification. Each sample transferred to clean Teflon vessel for acid digestion. Digestion was carried out with a mixture of concentrated HNO_3 (65–70%, Trace Metal Grade, Fisher Scientific) and HCl (35–38%, Trace Metal Grade, Fisher Scientific) in a ratio of 1:3 with a supplement of H_2O_2 (30%, Certified ACS, Fisher Scientific) at 200 °C for 50 min in a microwave digestion system (Titan MPS, PerkinElmer). Once the sample was cooled to rt, it was subsequently diluted to make a final volume of 50 mL by adding filtered DI water for analysis. The calibration curve was established using a standard solution while the dwell time was 50 ms with thirty sweeps and three replicates with background correction.

2. Optimization of reaction conditions

Preparation of a stock solution of $[Pd(crotyl)Cl]_2$ in THF:

To a 1–dram vial with a PTFE coated magnetic stir–bar was added $[Pd(crotyl)Cl]_2$ (5 mg, 0.012 mmol). The vial was sealed with a rubber septum and evacuated and backfilled with argon three times using an argon/vacuum manifold. This was followed by the addition of anhydrous THF (obtained from a solvent purification system; 1 mL). The vial was stirred gently at rt until the $[Pd(crotyl)Cl]_2$ dissolved completely. For a 0.25 mmol scale reaction, 50 µL of the stock solution would contain 0.25 mol % of the dimer (0.5 mol % [Pd]), which was used directly for further optimization.

2.1. Ligand screen

Reaction setup: To a 1–dram vial equipped with a PTFE coated magnetic stir–bar was added 1–bromo–3–(methylsulfonyl)benzene (**1a**; 1 equiv, 0.25 mmol, 58.7 mg). The vial was sealed with a rubber septum, evacuated, and backfilled with argon three times using an argon/vacuum manifold and then taken into an argon filled glovebox, where the specified ligand (2 mol %), and KO*t*Bu (2 equiv, 56 mg) was added. The vial was taken out of the glovebox and 2–(3,4–dimethoxyphenyl)ethan–1–amine (**1b**; 1.5 equiv, 0.375 mmol, 64µL) was added under an atmosphere of argon. Subsequently, a solution of 2 wt % Savie/H_2O (0.45 mL) was added followed by the addition of $[Pd(crotyl)Cl]_2$ (0.5 mol % Pd, 0.25 mol % of dimer) as a stock solution in THF (50 µL, see SI section 2). The reaction was allowed to stir at 60 °C for 8 h. Upon completion (as monitored by TLC), the reaction was extracted with EtOAc (4 x 1 mL). The combined extracts were dried over anhydrous Na_2SO_4, filtered, and concentrated *in vacuo*. Subsequently, 2 mL of $CDCl_3$ was added followed by the addition of 1,3,5–trimethoxybenzene (5–10 mg) as internal standard and the sample was analyzed by ^1H NMR (10s relaxation delay).

Table S10. Ligand screening for coupling of aryl bromides with aliphatic amines.

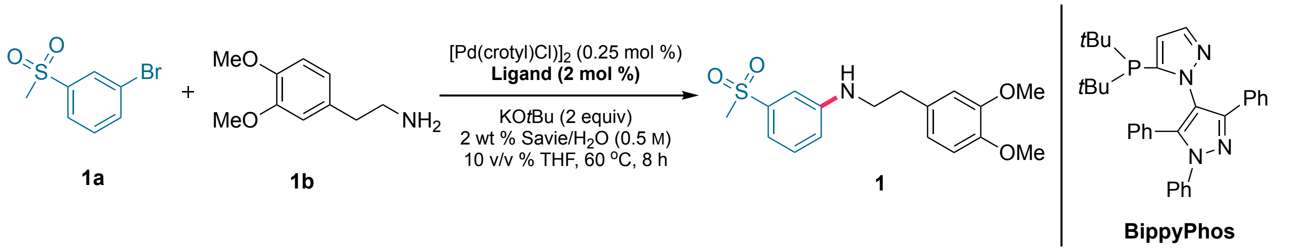

entry[a]	ligand	yield (%)[b]
1	*t*BuXPhos	trace
2	*t*BuBrettPhos	trace
**3**	**BippyPhos**	**99(90)[e]**
4	CyBippyPhos	N/R[c]
5	AdBippyPhos	trace
6	dtbpf [d]	NR
7	*t*BuXantPhos [d]	NR
8	$[Pd(P^tBu_3)Br]_2$	trace
9	*c*BRIDP	trace
10	*v*BRIDP	40
11	QPhos	trace
12	MorDalPhos	trace
13	XPhos	28
14	RuPhos	trace
16	PAd_3	20

[a] Reactions were carried out on a 0.25 mmol scale; [b] NMR yields using 1,3,5 –trimethoxybenzene as internal standard; [c] N/R: no reaction; [d] Reaction was run at 70 °C for 24 h; [e] isolated yield.

Structures of Ligands

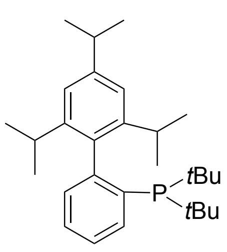

***t*BuXPhos**

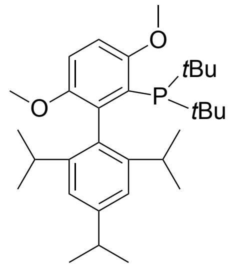

***t*BuBrettPhos**

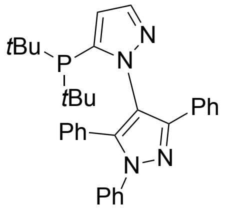

BippyPhos

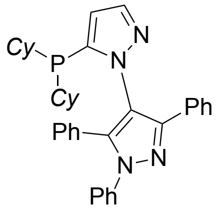

CyBippyPhos

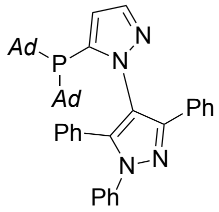

AdBippyPhos

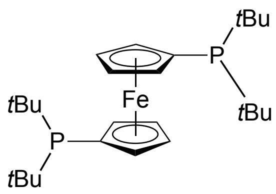

D*t*BPF

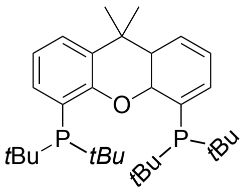

***t*BuXantPhos**

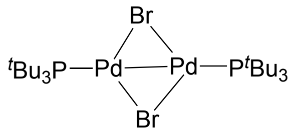

[Pd(P^{t}Bu$_3$)Br]$_2$

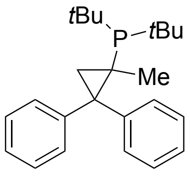

***c*BRIDP**

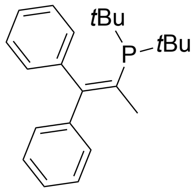

***v*BRIDP**

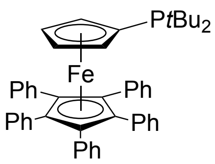

QPhos

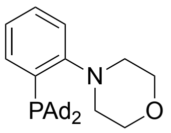

MorDalPhos

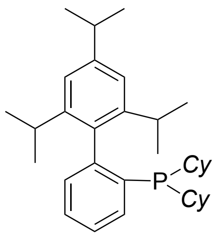

XPhos

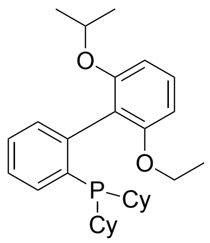

RuPhos

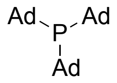

PAd$_3$

2.2. Screening of bases

Table S2. Screening of bases for coupling of aryl bromides with aliphatic amines.

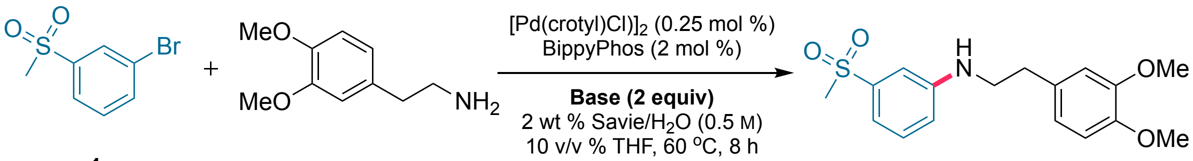

1a 1b 1

entry[a]	base	yield (%)[b]
1	NaO*t*Bu	90
2	KO*t*Bu	99
3	$K_3PO_4 \cdot H_2O$	70
4	Cs_2CO_3	55
5	Et_3N	trace
**6**	**KOH (2 equiv) / *t*BuOH (2.5 equiv)**	**96**
7	DBU[c]	26
8	KOTMS[d]	78

[a] Reaction conditions: 0.25 mmol **1a**, 0.375 mmol **1b**, $[Pd(crotyl)Cl]_2$ (50 µL, 0.5 mol % Pd, 0.25 mol % of the dimer used, administered as a stock solution in THF), BippyPhos (2 mol %, 2.5 mg) , base (2 equiv), 2 wt % Savie/H_2O (0.45 mL, 0.5 M), 60 °C, 8 h; [b] NMR yields using 1,3,5–trimethoxybenzene as internal standard; [c] DBU: 1,8–Diazabicyclo[5.4.0]undec–7–ene; [d] KOTMS: Potassium trimethylsilanoate.

2.3. Catalyst loading screen

Table S3. Effect of different loadings of catalyst.

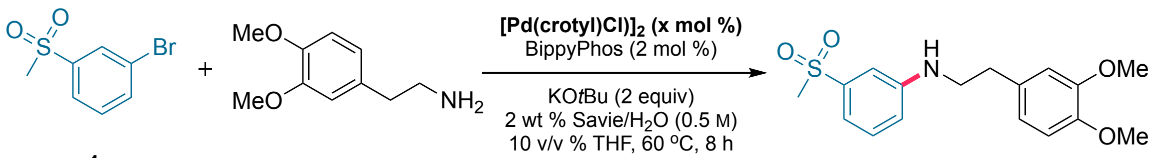

1a 1b 1

entry[a]	catalyst loading (Pd)	yield (%)[b]
1	0.075 mol % dimer (0.15 mol % Pd)	trace
2	0.1 mol % dimer (0.2 mol % Pd)	65
3	0.25 mol % dimer (0.5 mol % Pd)	99

4	0.5 mol % dimer (1 mol % Pd)	99

[a] Reaction conditions: 0.25 mmol **1a**, 0.375 mmol **1b**, $[Pd(crotyl)Cl]_2$ (0.5 – 0.15 mol % Pd, 0.25 – 0.075 mol % of the dimer used, administered as a stock solution in THF), BippyPhos (2 mol %, 2.5 mg), KO*t*Bu (2 equiv), 2 wt % Savie/H_2O (0.45 mL, 0.5 M), 60 °C, 8 h were carried out on a 0.25 mmol scale; [b] NMR yields using 1,3,5–trimethoxybenzene as internal standard.

2.4. Additional experiments

Table S4. Variation in reaction conditions for coupling of aryl bromides with aliphatic amines.

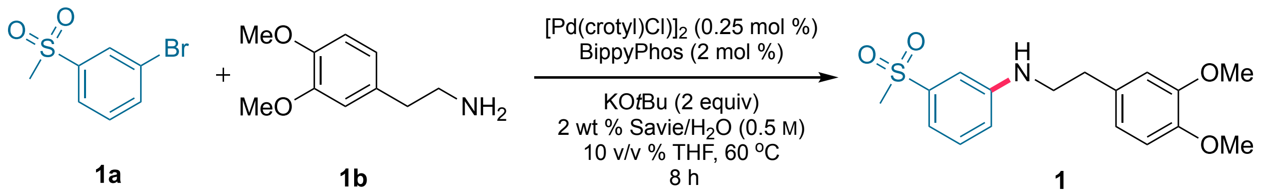

entry[a]	deviations from standard conditions	yield (%)[b]
1	none	99
2	35 °C	43
3	45 °C	68
**4**	**60 °C, 1.5 h**	**99 (90)**[c]
5	No Pd	0
6	No ligand	trace
7	Pure H_2O instead of Savie	60%

[a] Reactions were carried out on a 0.25 mmol scale; [b] NMR yields using 1,3,5 – trimethoxybenzene as internal standard; [c] isolated yield.

3. General procedure for the coupling of aryl halides with aliphatic amines: Procedure 1

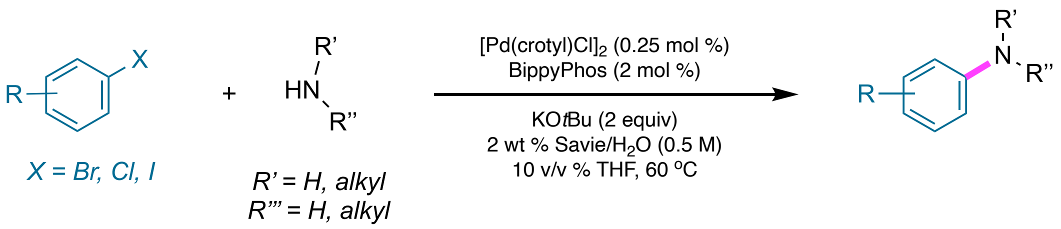

Scheme S1. Coupling of aryl halides with aliphatic amines.

Reaction setup: To a 1–dram vial equipped with a PTFE coated magnetic stir–bar was added the aryl halide (1 equiv, 0.25 mmol, if solid) followed by the addition of the amine (0.375 mmol, 1.5 equiv, if solid). The vial was sealed with a rubber septum, evacuated, and backfilled with argon three times using an argon/vacuum manifold and then taken into an argon filled glovebox, where BippyPhos (2 mol %, 2.5 mg), and KO*t*Bu (2 equiv, 56 mg) were then added. The vial was taken out of the glovebox and aryl bromide was added (if liquid) followed by the addition of the amine (if liquid) under an atmosphere of argon. Subsequently, a solution of 2 wt % Savie/H_2O (0.45 mL) was added followed by the addition of $[Pd(crotyl)Cl]_2$ (0.5 – 0.75 mol % Pd, 0.25 – 0.375 mol % of dimer) as a stock solution in THF (50 µL, see SI, section 2). The reaction was allowed to stir at 60 °C for the designated amount of time. Upon completion (as monitored by TLC), the reaction was extracted with EtOAc (4 x 1 mL). The combined extracts were dried over anhydrous Na_2SO_4, filtered, concentrated in vacuo, and subjected to flash chromatography using the desired eluent (EtOAc/hexanes or MeOH/CH_2Cl_2, see analytical section for choice of eluents) to obtain the desired coupled products.

4. General procedure for aminations using ocean water: Procedure 2

4.1. Preparation of a 2 wt % solution of Savie in ocean water

Ocean water (100 mL; from the Pacific Ocean) was first filtered through a medium porosity sintered glass funnel to remove any undissolved material and then transferred into a 100 mL round bottom flask. Subsequently, the flask was sealed with a rubber septum and the water was degassed overnight with argon, employing a vent needle.

In an oven dried 6–dram vial equipped with a PTFE coated magnetic stir–bar was added Savie (100 mg). The vial was sealed with a rubber septum, evacuated, and backfilled with argon three times using an argon/vacuum manifold. Subsequently, 4.9 mL of degassed ocean water was added via a syringe and the solution was allowed to stir at rt until complete dissolution of Savie in water (typically 10 min), resulting in a 2 wt % solution of Savie in ocean water.

4.2. Procedure for aminations using ocean water

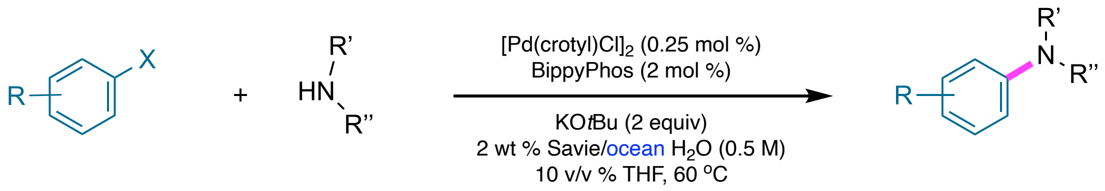

Scheme S2. Aminations using ocean water.

Reaction setup: To a 1–dram vial equipped with a PTFE coated magnetic stir–bar was added the aryl halide (1 equiv, 0.25 mmol, if solid) followed by the addition of the amine (0.375 mmol, 1.5 equiv, if solid). The vial was sealed with a rubber septum, evacuated, and backfilled with argon three times using an argon/vacuum manifold and then taken into an argon filled glovebox, where BippyPhos (2 mol %, 2.5 mg), and KO*t*Bu (2 equiv, 56 mg) were added. The vial was taken out of the glovebox and aryl halide was added (if liquid) followed by the addition of the amine (if liquid) under an atmosphere of argon. Subsequently, a solution of 2 wt % Savie/ocean H_2O (0.45 mL) was added followed by the addition of $[Pd(crotyl)Cl]_2$ (0.25 mol %, 0.5 mol % Pd) as a stock solution in THF (50 μL, see SI, section 2). The reaction was allowed to stir at 60 °C for a designated amount of time. Upon completion (as monitored by TLC), the reaction was extracted with EtOAc (4 x 1 mL). The combined extracts were dried over anhydrous Na_2SO_4, filtered, concentrated *in vacuo*, and subjected to flash chromatography using the desired eluent (EtOAc/hexanes or $MeOH/CH_2Cl_2$, see analytical section for choice of eluents) to obtain the desired coupled products (Note: This experiment was run on two substrates, compounds **12** and **24**; see Scheme 4).

Following the procedure above, Product **12** was prepared at 60 °C for 45 min using 3–bromoanisole (47 mg, 32 μL, 0.250 mmol), 1–(bis(4–fluorophenyl)methyl)piperazine (108.12 mg, 0.375 mmol), 2500 ppm (0.25 mol %) $[Pd(crotyl)Cl]_2$ as catalyst, and 2 mol % BippyPhos (2.5 mg) as ligand. Chromatography conditions: 5–7% EtOAc/hexanes. Yield: 97%, 95.6 mg; white solid; R_f = 0.29 (10% EtOAc/hexanes, UV, CAM stain).

Following the procedure above, Product **24** was prepared according at 60 °C for 1 h using fenofibrate (90.2 mg, 0.250 mmol), furfurylamine (36.4 mg, 0.375 mmol), 2500 ppm (0.25 mol %) $[Pd(crotyl)Cl]_2$ as catalyst, 2 mol % BippyPhos (2.5 mg) as ligand. Chromatography conditions: 15–25% EtOAc/hexanes. Yield: 95%, 100 mg; yellow oil ; R_f = 0.12 (15% EtOAc/hexanes, UV, CAM stain).

For analytical data see SI section 9.

5. Recycling studies and E Factor calculations

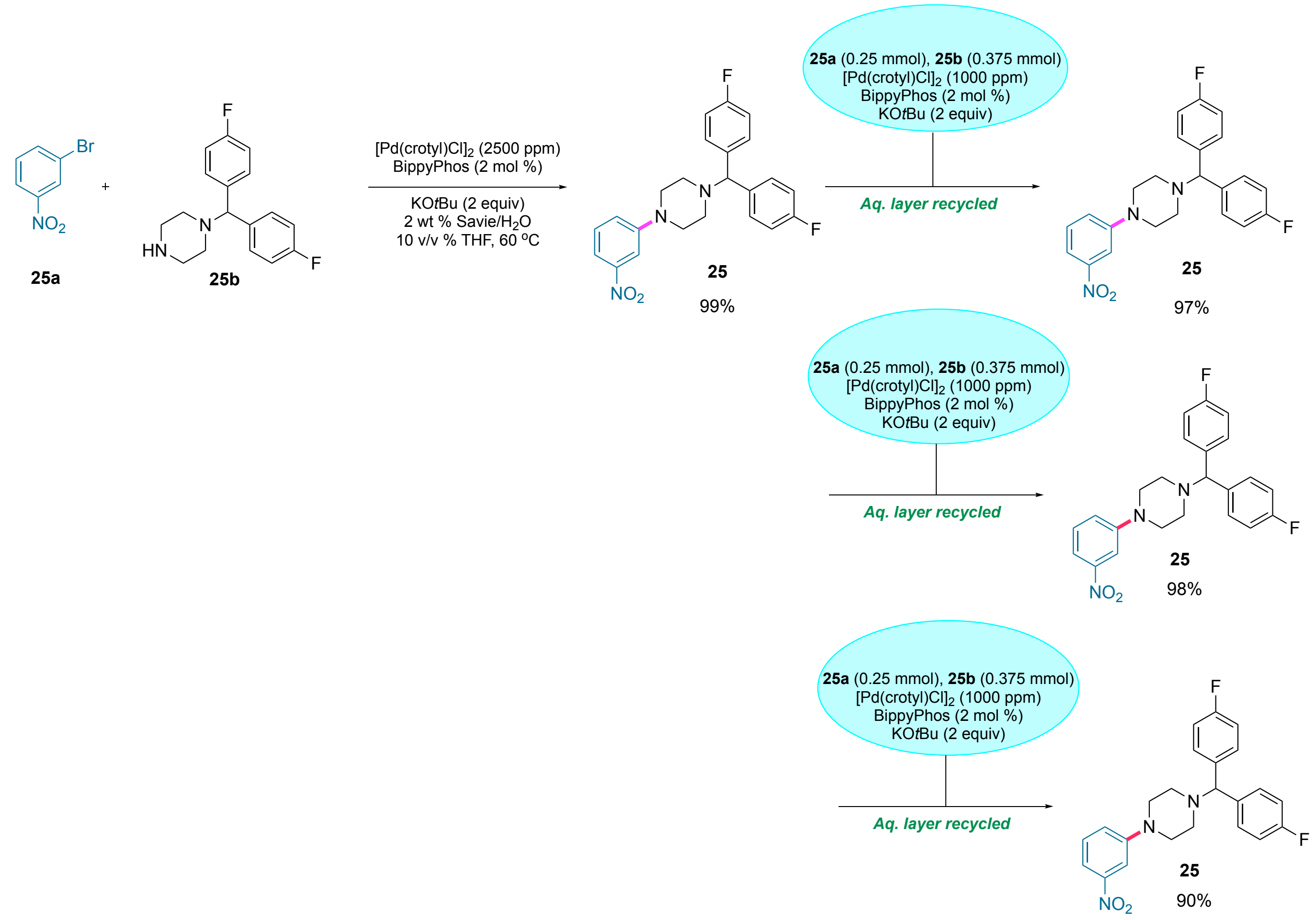

Scheme S3. Representative example demonstrating the recyclability of the aqueous medium.

5.1. Recycling studies

1st Reaction:

To a 1–dram vial equipped with a PTFE coated magnetic stir–bar was added 1–bromo–3–nitrobenzene **25a** (1 equiv, 0.25 mmol, 50.5 mg) followed by the addition of 1–(*bis*(4–fluorophenyl)methyl)piperazine **25b** (0.375 mmol, 1.5 equiv, 108.1 mg). The vial was sealed with a rubber septum, evacuated, and backfilled with argon three times using an argon/vacuum manifold and then taken into an argon filled glovebox, where BippyPhos (2 mol %, 2.5 mg), and KO*t*Bu (2 equiv, 56 mg) were added. The vial was taken out of the glovebox and under an atmosphere of argon, a solution of 2 wt % Savie/H_2O (0.45 mL) was added followed by the addition of $[Pd(crotyl)Cl]_2$ (2500 ppm, 0.5 mol % Pd, 0.25 mol % of dimer) as a stock solution in THF (50 μL, see SI, section 2). The reaction was allowed to stir at 60 °C for 1 h. Upon completion (as

monitored by TLC), the reaction was extracted with MTBE (2 x 0.6 mL). The organic layer was carefully removed via syringe. The combined organic extracts were concentrated in vacuo and purified by flash chromatography over silica gel (5–10% EtOAc/hexanes) to afford 1–(bis(4–fluorophenyl)methyl)–4–(3–nitrophenyl)piperazine **25** as a yellow solid (101.3 mg, 99% yield).

2nd Reaction (1st recycle):

To the same vial containing the aqueous layer from the previous reaction was added 1–bromo–3–nitrobenzene **25a** (1 equiv, 0.25 mmol, 50.5 mg) followed by the addition of 1–(bis(4–fluorophenyl)methyl)piperazine **25b** (0.375 mmol, 1.5 equiv, 108.1 mg). Subsequently, BippyPhos (2 mol %, 2.5 mg), and KO*t*Bu (2 equiv, 56 mg) were quickly added under an atmosphere of argon and the vial was sealed with a rubber septum and purged with argon for 5 min, employing a vent needle. Then, $[Pd(crotyl)Cl]_2$ (1000 ppm, 0.2 mol % Pd, 0.1 mol % of dimer) was added as a stock solution in THF (40 μL, see SI, section 2), and the reaction was stirred at 60 °C for 1 h. Upon completion (as monitored by TLC), the reaction was extracted with MTBE (2 x 0.6 mL). The organic layer was carefully removed via syringe. The combined organic extracts were concentrated in vacuo and purified by flash chromatography over silica gel (5–10% EtOAc/hexanes) to afford 1–(bis(4–fluorophenyl)methyl)–4–(3–nitrophenyl)piperazine **25** as a yellow solid (99.3 mg, 97% yield).

3rd Reaction (2nd recycle):

To the same vial containing the aqueous layer from the previous reaction was added 1–bromo–3–nitrobenzene **25a** (1 equiv, 0.25 mmol, 50.5 mg) followed by the addition of 1–(bis(4–fluorophenyl)methyl)piperazine **25b** (0.375 mmol, 1.5 equiv, 108.1 mg). Subsequently, BippyPhos (2 mol %, 2.5 mg), and KO*t*Bu (2 equiv, 56 mg) were quickly added under an atmosphere of argon and the vial was sealed with a rubber septum and purged with argon for 5 min, employing a vent needle. Then, $[Pd(crotyl)Cl]_2$ (1000 ppm, 0.2 mol % Pd, 0.1 mol % of dimer) was added as a stock solution in THF (40 μL, see SI section 2), and the reaction was stirred at 60 °C for 1 h. Upon completion (as monitored by TLC), the reaction was extracted with MTBE (2 x 0.6 mL). The organic layer was carefully removed via syringe. The combined organic extracts were concentrated in vacuo and purified by flash chromatography over silica gel (5–10% EtOAc/hexanes) to afford 1–(bis(4–fluorophenyl)methyl)–4–(3–nitrophenyl)piperazine **25** as a yellow solid (100.2 mg, 98% yield).

4th Reaction (3rd recycle):

To the same vial containing the aqueous layer from the previous reaction was added 1–bromo–3–nitrobenzene **25a** (1 equiv, 0.25 mmol, 50.5 mg) followed by the addition of 1–(bis(4–

fluorophenyl)methyl)piperazine **25b** (0.375 mmol, 1.5 equiv, 108.1 mg). Subsequently, BippyPhos (2 mol %, 2.5 mg), and KO*t*Bu (2 equiv, 56 mg) were quickly added under an atmosphere of argon and the vial was sealed with a rubber septum and purged with argon for 5 min, employing a vent needle. Then, $[Pd(crotyl)Cl]_2$ (1000 ppm, 0.2 mol % Pd, 0.1 mol % of dimer) was added as a stock solution in THF (40 µL, see SI section 2), and the reaction was stirred at 60 °C for 1 h. Upon completion (as monitored by TLC), the reaction was extracted with MTBE (2 x 0.6 mL). The organic layer was carefully removed via syringe. The combined organic extracts were concentrated in vacuo and purified by flash chromatography over silica gel (5–10% EtOAc/hexanes) to afford 1–(*bis*(4–fluorophenyl)methyl)–4–(3–nitrophenyl)piperazine **25** as a yellow solid (92.1 mg, 90% yield).

5.2. E Factor calculations

$$\textbf{E factor} = \frac{\textbf{mass of organic waste}}{\textbf{mass of product}}$$

$$\text{E factor (with water)} = \frac{\text{mass of organic waste + mass of water}}{\text{mass of product}}$$

Organic waste:

1. Excess reagents

- Amine **25b**: 0.5 equiv excess used in 4 reactions.

  Mass: (0.25 x 0.5 x 288.34 x 4) = **144.17 mg (0.144 g)**

2. Extraction solvent

- MTBE: (0.6 x 2) = 1.2 mL used in each reaction

  Total volume = (1.2 x 4) = 4.8 mL
  Density of MTBE = 0.74 g/cm^3

  Total mass of MTBE = (4.8 x 0.74) = **3.552 g**

3. Mass of water = **0.45 g**

Mass of product = (0.1013 + 0.0993 + 0.1002 + 0.0921) = **0.393 g**

$$\text{E factor (with water)} = \frac{0.144 + 3.552 + 0.45}{0.393}$$

$$= \mathbf{10.5}$$

$$\text{E factor (without water)} = \frac{0.144 + 3.552}{0.393}$$

$$= \mathbf{9.4}$$

$$\text{E factor (without extraction solvent)} = \frac{0.144 + 0.45}{0.393}$$

$$= \mathbf{1.51}$$

6. Synthesis of naftopidil

6.1. General procedure for amination to afford intermediate 40

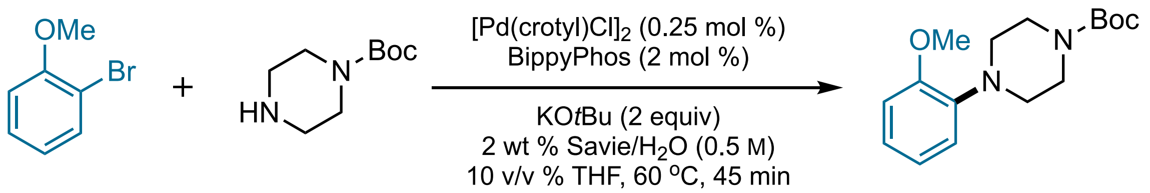

40; *89%*

Scheme S4. Amination to afford intermediate **40**.

To a 1–dram vial equipped with a PTFE coated magnetic stir–bar was added 2-bromoanisole (1 equiv, 0.25 mmol, 47 mg) followed by the addition of *t*–butyl piperazine–1–carboxylate (139.7 mg, 0.375 mmol). The vial was sealed with a rubber septum, evacuated, and backfilled with argon three times using an argon/vacuum manifold and then taken into an argon filled glovebox, where BippyPhos (2 mol %, 2.5 mg), and KO*t*Bu (2 equiv, 56 mg) were added. The vial was taken out of the glovebox and under an atmosphere of argon, a solution of 2 wt % Savie/H_2O (0.45 mL) was added followed by the addition of $[Pd(crotyl)Cl]_2$ (2500 ppm, 0.5 mol % Pd, 0.25 mol % of dimer) as a stock solution in THF (50 µL, see SI, section 2). The reaction was allowed to stir at 60 °C for 1 h. Upon completion (as monitored by TLC), the reaction was

extracted with EtOAc (4 x 1 mL). The combined organic extracts were concentrated *in vacuo* and purified by flash chromatography over silica gel (5–10% EtOAc/hexanes) to afford *t*-butyl 4-(2-methoxyphenyl)piperazine-1-carboxylate **40** as a colorless oil (65 mg, 89% yield).

Procedure for gram scale reaction:

To a 25 ml round bottom flask vial equipped with a PTFE coated magnetic stir–bar was added 2-bromoanisole (1 equiv, 6 mmol, 1.12 g) followed by the addition of *t*–butyl piperazine–1–carboxylate (1.5 equiv, 1.67 g, 9 mmol). The vial was sealed with a rubber septum, evacuated, and backfilled with argon three times using an argon/vacuum manifold and then taken into an argon filled glovebox, where BippyPhos (2 mol %, 61 mg), and KO*t*Bu (2 equiv, 1.34 g) were added. The vial was taken out of the glovebox and under an atmosphere of argon, a solution of 2 wt % Savie/H_2O (12 mL) was added followed by the addition of $[Pd(crotyl)Cl]_2$ (2500 ppm, 0.5 mol % Pd, 0.25 mol % of dimer; 5.9 mg) The reaction was allowed to stir at 60 °C for 1 h. Upon completion (as monitored by TLC), the reaction was extracted with EtOAc (4 x 15 mL). The combined organic extracts were concentrated *in vacuo* and purified by flash chromatography over silica gel (5–10% EtOAc/hexanes) to afford *t*-butyl 4-(2-methoxyphenyl)piperazine-1-carboxylate **40** as a colorless oil (1.49 g, 85% yield).

6.2. Optimization of *N*–Boc deprotection of intermediate 40

Reaction setup: To a 1–dram vial with a PTFE coated magnetic stir–bar was added compound **40** (1 equiv, 0.25 mmol, 73.1 mg), followed by the addition of HCl in different solvents (5 equiv). The reaction was allowed to stir at 50 °C for 2 h. Upon completion, the crude reaction mixture was concentrated *in vacuo* to remove most of the HCl (or TFA) and then precipitated with diethyl ether (precipitated solids were already observed). The resulting reaction mixture was centrifuged, and the ether layer was decanted via pipette. This procedure was repeated two more times and the resulting solid was dried under high vacuum to afford crude compound **41** (For the reaction using 4 M HCl/H_2O, the crude reaction mixture was free-based using a 10% K_2CO_3 solution and was subsequently extracted using EtOAc). Crude NMRs were taken in $CDCl_3$, and conversions were determined based on the disappearance of the Boc group.

Table S5. Optimization of *N*–Boc deprotection on model compound **41**.

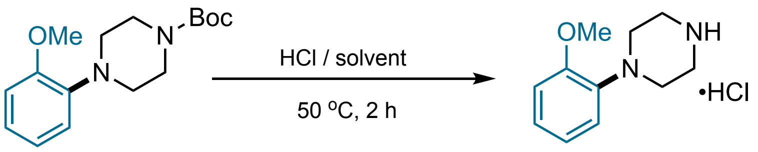

entry[a]	conditions	conversion (%)[b]
1	1 M HCl in EtOAc (5 equiv)	50
2	4 M HCl in dioxane (5 equiv)	>99
3	1 M HCl in AcOH (5 equiv)	>99
**4**	**4 M HCl (in H_2O) (5 equiv)**	**>99**
5	3 M HCl in MeOH (5 equiv)	
**6**	**5–6 M HCl in *i*PrOH (5 equiv)**	**>99**

[a] Reactions were carried out on a 0.25 mmol scale for 2 h; [b] conversions were determined by crude NMR analysis.

Note: For purposes of NMR spectroscopy, entry 6 was used to isolate the HCl salt of the amine (see analytical section for NMR spectra).

6.3. 2-Step, one-pot sequence to afford intermediate 41b (free base)

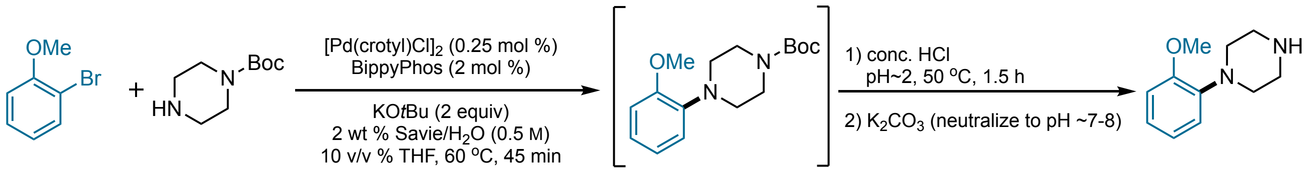

Scheme S5. 2-Step, one pot sequence to afford intermediate **41b.**

Reaction setup: To a 1–dram vial equipped with a PTFE coated magnetic stir–bar was added 2-bromoanisole (1 equiv, 0.25 mmol, 47 mg) followed by the addition of *t*–butyl piperazine–1–carboxylate (139.7 mg, 0.375 mmol). The vial was sealed with a rubber septum, evacuated, and backfilled with argon three times using an argon/vacuum manifold and then taken into an argon filled glovebox, where BippyPhos (2 mol %, 2.5 mg), and KO*t*Bu (2 equiv, 56 mg) were then added. The vial was taken out of the glovebox and under an atmosphere of argon, a solution of 2 wt % Savie/H_2O (0.45 mL) was added followed by the addition of $[Pd(crotyl)Cl]_2$ (2500 ppm, 0.5 mol % Pd, 0.25 mol % of dimer) as a stock solution in THF (50 µL, see SI, section 2). The reaction was allowed to stir at 60 °C for 1 h. Upon completion (as monitored by TLC), the reaction mixture was acidified with conc. HCl to pH~2 (approx. 2-3 drops) and was stirred at 50

°C for 1.5 h. Complete deprotection of the Boc group was observed by TLC. Upon completion, the aqueous layer was first extracted with diethyl ether (2 x 1 mL) to remove any organic impurities from the previous step. Subsequently, solid K_2CO_3 was added portion wise to neutralize the reaction mixture to pH 7–8, and the resulting aqueous layer was extracted with EtOAc (3 x 2 mL). The combined organics were concentrated *in vacuo* to afford intermediate **41b** as the free base (41.3 mg, colorless oil).

Note: For purposes of NMR spectroscopy, HCl salt of the amine was isolated (see analytical section for NMR spectra).

6.4. Synthesis of intermediate 42

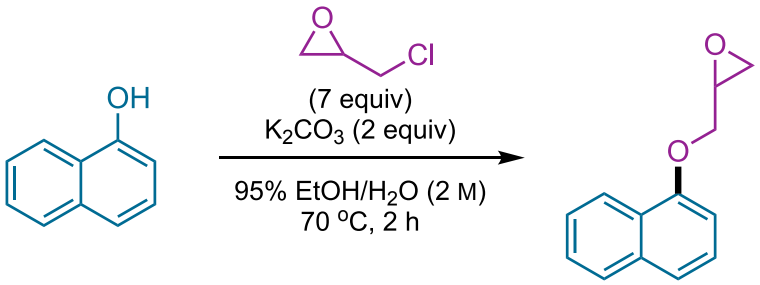

42; *85% isolated yield*

Scheme S6. S_N2 Reaction to afford intermediate **42**.

Reaction setup: To a 6-dram vial with a PTFE coated magnetic stir bar was added 1-naphthol (7 mmol, 1 g, 1 equiv) followed by the addition of K_2CO_3 (14 mmol, 1.6 g, 2 equiv), and 95% EtOH (3.5 mL, 2 M). This mixture was allowed to stir at 50 °C for 30 min. Subsequently, epichlorohydrin (49 mmol, 4.7 g, 4 mL, 7 equiv) was added dropwise over a period of 10 min, and the resultant reaction mixture was allowed to stir at 70 °C for 2 h. Upon completion, as observed by TLC analysis, the reaction mixture was concentrated *in vacuo* to remove most of the EtOH and was subsequently neutralized with 1 M HCl. The resultant aqueous layer was extracted with EtOAc (3 x 5 mL). The combined organic extracts were concentrated *in vacuo* and purified by flash chromatography over silica gel (5–10% EtOAc/hexanes) to afford 2-((naphthalen-1-yloxy)methyl)oxirane (**42**) as a yellow oil (1.2 g, 85% yield).

6.5. Synthesis of naftopidil

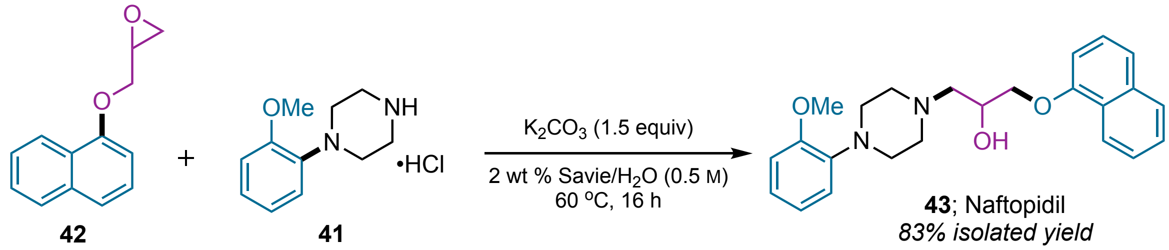

Scheme S7. Epoxide opening to afford naftopidil **43**.

Reaction setup: To a 1-dram vial with a PTFE coated magnetic stir bar was added 2-((naphthalen-1-yloxy)methyl)oxirane (**42**; 0.25 mmol, 50 mg, 1 equiv), followed by the addition of 1-(2-methoxyphenyl)piperazine hydrochloride (**41**; 0.3 mmol, 68.6 mg, 1.2 equiv) and K_2CO_3 (1.5 equiv, 0.375 mmol, 52 mg). Subsequently, a solution of 2 wt % Savie/H_2O was added (0.5 mL) and the resultant reaction mixture was allowed to stir at 60 °C for 16 h. Upon completion (as monitored by TLC analysis), the reaction mixture was neutralized to pH 7–8 using 1 M HCl, and the aqueous layer was extracted using 5% MeOH/CH_2Cl_2 (4 x 1 mL). The combined organic extracts were concentrated *in vacuo* and purified by flash chromatography over silica gel (1–5% MeOH/CH_2Cl_2) to afford naftopidil **43** as an off–white solid (81 mg, 83% yield).

6.6. 3-Step, one pot synthesis of naftopidil – Procedure 3

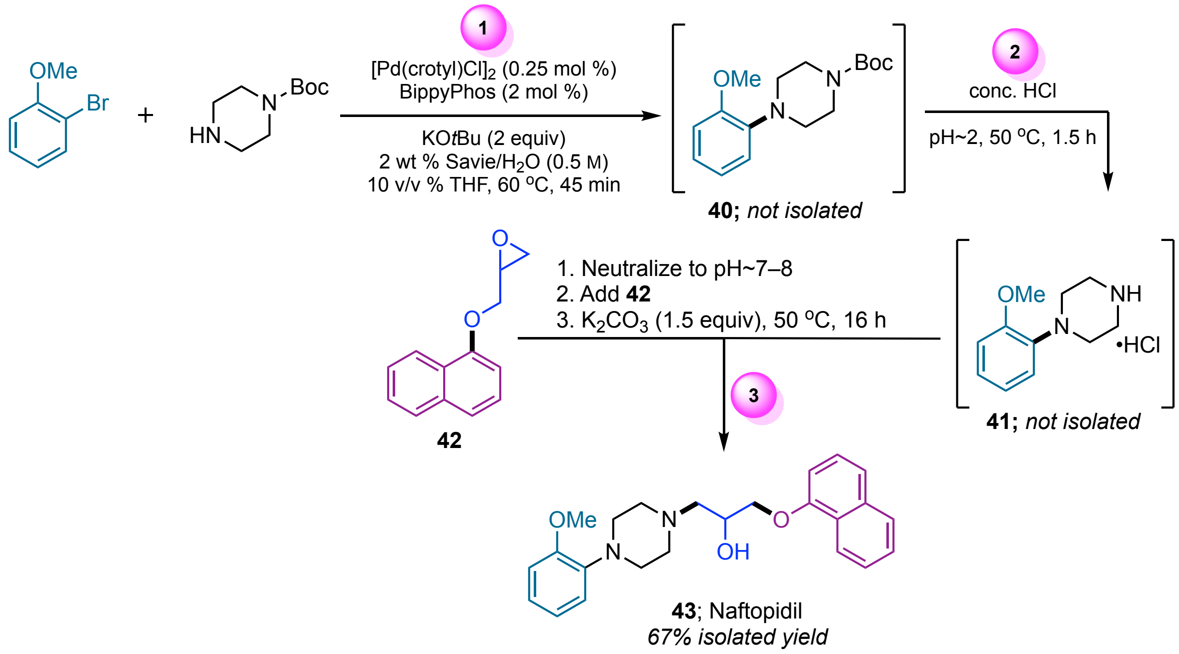

Step 1: Pd–catalyzed amination

To a 1–dram vial equipped with a PTFE coated magnetic stir–bar was added 2-bromoanisole (1 equiv, 0.25 mmol, 47 mg) followed by the addition of *t*–butyl piperazine–1–carboxylate (139.7 mg, 0.375 mmol). The vial was sealed with a rubber septum, evacuated, and backfilled with argon three times using an argon/vacuum manifold and then taken into an argon filled glovebox, where BippyPhos (2 mol %, 2.5 mg), and KO*t*Bu (2 equiv, 56 mg) were then added. The vial was taken out of the glovebox and under an atmosphere of argon, a solution of 2 wt % Savie/H_2O (0.45 mL) was added followed by the addition of $[Pd(crotyl)Cl]_2$ (2500 ppm, 0.5 mol % Pd, 0.25 mol % of dimer) as a stock solution in THF (50 μL, see SI, section 2). The reaction was allowed to stir at 60 °C for 1 h, until completion.

Step 2: *N*-Boc deprotection

Upon completion of the previous step (as monitored by TLC), the reaction mixture was acidified with conc. HCl to pH ~2 (approx. 2-3 drops) and was stirred at 50 °C for 1.5 h. Complete deprotection of the Boc group was observed by TLC. Upon completion, the aqueous layer was first extracted with diethyl ether (2 x 1 mL) to remove any organic impurities from the previous step. Subsequently, solid K_2CO_3 was added portion–wise to neutralize the reaction mixture to pH 7–8.

Step 3: Epoxide opening

Upon neutralizing the reaction mixture from the previous step, 2-((naphthalen-1-yloxy)methyl)oxirane (**42**; 0.25 mmol, 50 mg, 1 equiv), was added, followed by the addition of K_2CO_3 (0.375 mmol, 52 mg, 1.5 equiv). The resulting mixture was stirred at 60 °C for 16 h. Upon completion (as monitored by TLC analysis), the reaction mixture was neutralized to pH 7–8 using 1 M HCl, and the aqueous layer was extracted using 5% MeOH/CH_2Cl_2 (4 x 1 mL). The combined organic extracts were concentrated *in vacuo* and purified by flash chromatography over silica gel (1–5% MeOH/CH_2Cl_2) to afford naftopidil **43** as an off white solid (66 mg, 67% yield).

7. ICP–MS analysis for residual palladium

ICP–MS data for residual Pd was obtained from the University of California, Los Angeles, ICP–MS Core Facility.

		**palladium** [μg/g]		**sample source**
sample #	sample weight in analysis [mg]	average*	std dev	
KI–943-3	7.60	**0.780**	0.046	recycling studies – Reaction 1
KI–994–1	5.40	**0.000**	0.000	one–pot sequence – final compound **43** (naftopidil)

*Each sample was run in triplicate, with background correction.

8. References

1. Lipshutz, B. H.; Ghorai, S.; Abela, A. R.; Moser, R.; Nishikata, T.; Duplais, C.; Krasovskiy, A.; Gaston, R. D.; Gadwood, R. C. TPGS–750–M: A Second–Generation Amphiphile for Metal–Catalyzed Cross Couplings in Water at Room Temperature. *J. Org. Chem*. **2011**, *76*, 4379−4391.
2. Kincaid, J. R. A.; Wong, M. J.; Akporji, N.; Gallou, F.; Fialho, D. M.; and Lipshutz, B. H. Introducing Savie: A Biodegradable Surfactant Enabling Chemo–and Biocatalysis and Related Reactions in Recyclable Water. *J. Am. Chem. Soc*. **2023**, *145*, 4266–4278.
3. Kanno, T.; Tanaka, A.; Shimizu, T.; Nakano, T.; Nishizaki, T. Novel anticancer agent . U.S. Patent Application No. 14/298,400.
4. Kim, S. T.; Strauss, M. J.; Cabré, A.; Buchwald, S. L. Room Temperature Cu–Catalyzed Amination of Aryl Bromides Enabled by DFT–Guided Ligand Design. *J. Am. Chem. Soc.* **2023**, *145*, 6966−6975.
5. Li, C.; Kawamata, Y.; Nakamura, H.; Vantourout, J. C.; Liu, Z.; Hou, Q.; Bao, D.; Starr, J. T.; Chen, J.; Yan, M; and Baran, P. S. Electrochemically enabled, nickel-catalyzed amination. *Angew. Chem., Int. Ed.* **2017**, *129*, 13268–13273.

9. Analytical data

9.1. Analytical data for starting materials and intermediates

***t*-Butyl 4-(2-methoxyphenyl)piperazine-1-carboxylate (40)**

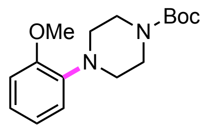

40

Compound **40** was prepared according to General Procedure 1 at 60 °C for 1 h using 2-bromoanisole (47 mg, 0.250 mmol), t–butyl piperazine–1–carboxylate (70 mg, 0.375 mmol), 2500 ppm (0.25 mol %) $[Pd(crotyl)Cl]_2$ as catalyst, and 2 mol % BippyPhos (2.5 mg) as ligand. Chromatography conditions: 5–10% EtOAc/hexanes. Yield: 89%, 65 mg; colorless oil; R_f = 0.21 (10% EtOAc/hexanes, UV, CAM stain).

^{1}H NMR (400 MHz, $CDCl_3$) δ 7.02 (ddd, *J* = 8.0, 5.5, 3.6 Hz, 1H), 6.96 – 6.90 (m, 2H), 6.90 – 6.83 (m, 1H), 3.87 (s, 3H), 3.66 – 3.55 (m, 4H), 3.05 – 2.94 (m, 4H), 1.48 (s, 9H).

^{13}C NMR (101 MHz, $CDCl_3$) δ 155.0, 152.4, 141.3, 123.4, 121.1, 118.5, 111.4, 79.8, 55.5, 50.8, 43.9, 28.6.

Spectral data matches that previously reported in the literature.[3]

2-Methoxyphenyl)piperazine hydrochloride (41)

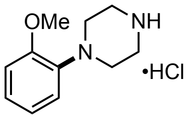

41

^{1}H NMR (500 MHz, D_2O) δ 7.53 – 7.43 (m, 2H), 7.23 (dd, *J* = 8.4, 1.3 Hz, 1H), 7.13 (td, *J* = 7.8, 1.3 Hz, 1H), 3.96 (s, 3H), 3.92 – 3.81 (m, 4H), 3.76 – 3.67 (m, 4H).

^{13}C NMR (126 MHz, D_2O) δ 151.4, 130.7, 130.0, 121.5, 120.5, 113.1, 55.9, 49.5, 41.6.

Spectral data matches that previously reported in the literature.[3]

2-((Naphthalen-1-yloxy)methyl)oxirane (42)

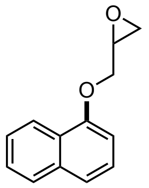

42

^{1}H NMR (500 MHz, $CDCl_3$) δ 8.37 (ddd, *J* = 6.1, 3.2, 1.4 Hz, 1H), 7.88 – 7.81 (m, 1H), 7.58 – 7.47 (m, 3H), 7.40 (t, *J* = 7.9 Hz, 1H), 6.81 (d, *J* = 7.6 Hz, 1H), 4.38 (ddd, *J* = 10.9, 3.1, 1.2 Hz, 1H), 4.15 – 4.08 (m, 1H), 3.49 (dqd, *J* = 5.5, 2.9, 1.4 Hz, 1H), 2.96 (td, *J* = 4.5, 1.0 Hz, 1H), 2.87 – 2.82 (m, 1H).

^{13}C NMR (126 MHz, $CDCl_3$) δ 154.2, 134.6, 127.5, 126.5, 125.8, 125.6, 125.4, 122.1, 120.9, 105.0, 68.9, 50.2, 44.7.

Spectral data matches that previously reported in the literature.[3]

9.2. Analytical data for products

***N*–(3,4–Dimethoxyphenethyl)–3–(methylsulfonyl)aniline (1)**

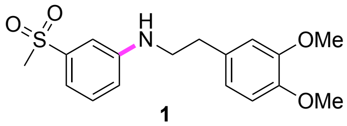

1

Product **1** was prepared according to General Procedure 1 at 60 °C for 1.5 h using 1–bromo–3–(methylsulfonyl)benzene (58.7 mg, 0.250 mmol), 3,4–dimethoxyphenethylamine (68 mg, 64 µL, 0.375 mmol), 2500 ppm (0.25 mol %) $[Pd(crotyl)Cl]_2$ as catalyst, and 2 mol % BippyPhos (2.5 mg) as ligand. Chromatography conditions: 50–60% EtOAc/hexanes. Yield: 90%, 75.4 mg; off white solid; R_f = 0.21 (50% EtOAc/hexanes, UV, CAM stain).

^{1}H NMR (500 MHz, $CDCl_3$) δ 7.31 (t, *J* = 7.9 Hz, 1H), 7.19 (dt, *J* = 7.8, 1.1 Hz, 1H), 7.09 (t, *J* = 2.1 Hz, 1H), 6.85 – 6.70 (m, 4H), 3.86 (d, *J* = 1.9 Hz, 6H), 3.41 (t, *J* = 6.8 Hz, 2H), 3.01 (s, 3H), 2.87 (t, *J* = 6.8 Hz, 2H).

^{13}C NMR (126 MHz, $CDCl_3$) δ 149.2, 148.8, 147.9, 141.5, 131.2, 130.3, 120.8, 117.8, 115.5, 112.0, 111.6, 110.4, 56.1, 56.0, 44.9, 44.5, 34.9.

HRMS (ESI$^+$): Calcd for $C_{17}H_{21}NO_4SNa$, $[M+Na]^+$ 358.1089; found 358.1081.

***N*–(3,4–Dimethoxyphenethyl)–3–fluoro–6–methylpyridin–2–amine (2)**

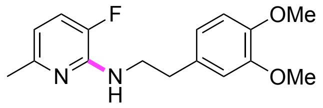

2

Product **2** was prepared according to General Procedure 1 at 60 °C for 1.5 h using 2–bromo–3–fluoro–6–methylpyridine (47.5 mg, 0.250 mmol), 3,4–dimethoxyphenethylamine (68 mg, 64 µL, 0.375 mmol), 2500 ppm (0.25 mol %) $[Pd(crotyl)Cl]_2$ as catalyst, and 2 mol % BippyPhos (2.5 mg) as ligand. Chromatography conditions: 5–15% EtOAc/hexanes. Yield: 82%, 59.5 mg; light yellow solid; R_f = 0.19 (15% EtOAc/hexanes, UV, CAM stain).

^{1}H NMR (500 MHz, $CDCl_3$) δ 6.98 (dd, *J* = 11.1, 7.8 Hz, 1H), 6.82 (d, *J* = 8.0 Hz, 1H), 6.80 – 6.74 (m, 2H), 6.32 (dd, *J* = 7.8, 3.0 Hz, 1H), 4.52 (d, *J* = 6.4 Hz, 1H), 3.87 (d, *J* = 1.1 Hz, 6H), 3.72 (td, *J* = 6.9, 5.8 Hz, 2H), 2.87 (t, *J* = 6.9 Hz, 2H), 2.37 (d, *J* = 1.0 Hz, 3H).

^{13}C NMR (126 MHz, $CDCl_3$) δ 151.63 (d, *J* = 5.5 Hz), 149.1, 147.7, 147.6, 147.5, 146.6, 144.6, 132.2, 120.9, 120.4, 120.3, 112.2, 111.5, 110.71 (d, *J* = 1.8 Hz), 56.1, 55.9, 42.4, 35.6, 24.0.

^{19}F NMR (471 MHz, $CDCl_3$) δ –147.5, –147.5.

HRMS (ESI$^+$): Calcd for $C_{16}H_{19}FN_2O_2Na$, $[M+Na]^+$ 313.1328; found 313.1334.

***N*–Butyl–4–(trifluoromethoxy)aniline (3)**

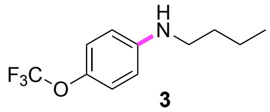

3

Product **29** was prepared according to General Procedure 1 at 60 °C for 1 h using 1–bromo–4–(trifluoromethoxy)benzene (60.3 mg, 37 µL, 0.250 mmol), *n*–butylamine (27.4 mg, 37 µL, 0.375 mmol), 2500 ppm (0.25 mol %) $[Pd(crotyl)Cl]_2$ as catalyst, and 2 mol % BippyPhos (2.5 mg) as ligand. Chromatography conditions: 0–2% EtOAc/hexanes. Yield: 86%, 50 mg; orange oil; R_f = 0.31 (1% EtOAc/hexanes, UV, CAM stain).

^{1}H NMR (500 MHz, CDCl$_3$) δ 7.03 (d, *J* = 8.4 Hz, 2H), 6.58 – 6.51 (m, 2H), 3.66 (s, 1H), 3.09 (t, *J* = 7.1 Hz, 2H), 1.61 (p, *J* = 7.2 Hz, 2H), 1.48 – 1.37 (m, 2H), 0.97 (t, *J* = 7.4 Hz, 3H).

^{13}C NMR (126 MHz, CDCl$_3$) δ 147.4, 140.32 (q, *J* = 2.0 Hz), 122.5, 120.89 (q, *J* = 255.1 Hz), 112.9, 44.0, 31.7, 20.4, 14.0.

^{19}F NMR (471 MHz, CDCl$_3$) δ –58.5.

HRMS (ESI$^+$): Calcd for $C_{11}H_{15}F_3NO$, $[M+H]^+$ 234.1106; found 234.1104.

(4–((Cyclopropylmethyl)amino)phenyl)(phenyl)methanone (4)

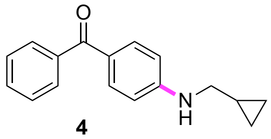

4

Product **4** was prepared according to General Procedure 1 at 60 °C for 2 h using 4–bromobenzophenone (65.3 mg, 0.250 mmol), (aminomethyl)cyclopropane (26.7 mg, 33 µL, 0.375 mmol), 2500 ppm (0.25 mol %) [Pd(crotyl)Cl]$_2$ as catalyst, and 2 mol % BippyPhos (2.5 mg) as ligand. Chromatography conditions: 10–12.5% EtOAc/hexanes. Yield: 98%, 61.5 mg; light yellow solid; R_f = 0.15 (10% EtOAc/hexanes, UV, CAM stain).

^{1}H NMR (500 MHz, CDCl$_3$) δ 7.73 (ddd, *J* = 15.6, 7.6, 1.8 Hz, 4H), 7.56 – 7.48 (m, 1H), 7.48 – 7.41 (m, 2H), 6.61 – 6.55 (m, 2H), 4.43 (s, 1H), 3.04 (d, *J* = 7.0 Hz, 2H), 1.17 – 1.05 (m, 1H), 0.62 – 0.56 (m, 2H), 0.27 (dt, *J* = 6.1, 4.6 Hz, 2H).

^{13}C NMR (126 MHz, CDCl$_3$) δ 195.2, 152.3, 139.3, 133.1, 131.3, 129.6, 128.1, 126.1, 111.3, 48.4, 10.8, 3.7.

HRMS (ESI$^+$): Calcd for $C_{17}H_{18}NO$, $[M+H]^+$ 252.1388; found 252.1386.

***N*–Cyclohexyl–4–methylnaphthalen–1–amine (5)**

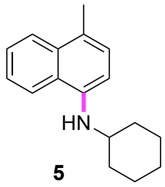

5

Product **5** was prepared according to General Procedure 1 at 60 °C for 1.5 h using 1–bromo–4–methylnaphthalene (55.3 mg, 39 µL, 0.250 mmol), cyclohexylamine (37.2 mg, 43 µL, 0.375 mmol), 2500 ppm (0.25 mol %) $[Pd(crotyl)Cl]_2$ as catalyst, and 2 mol % BippyPhos (2.5 mg) as ligand. Chromatography conditions: 1–2% EtOAc/hexanes. Yield: 70%, 42 mg; brown viscous oil; R_f = 0.44 (10% EtOAc/hexanes, UV–fluorescent spot, CAM stain).

^{1}H NMR (400 MHz, $CDCl_3$) δ 7.95 (dd, *J* = 8.2, 1.4 Hz, 1H), 7.88 – 7.81 (m, 1H), 7.55 – 7.41 (m, 2H), 7.18 (d, *J* = 7.7 Hz, 1H), 6.58 (d, *J* = 7.7 Hz, 1H), 3.45 (tt, *J* = 9.9, 3.7 Hz, 1H), 2.59 (s, 3H), 2.18 (dd, *J* = 12.9, 4.0 Hz, 2H), 1.83 (dp, *J* = 11.6, 3.9 Hz, 2H), 1.74 – 1.66 (m, 1H), 1.54 – 1.22 (m, 6H).

^{13}C NMR (101 MHz, $CDCl_3$) δ 141.1, 133.5, 127.2, 125.6, 125.1, 124.4, 124.1, 122.5, 120.6, 105.1, 52.1, 33.5, 26.2, 25.2, 19.0.

HRMS (ESI$^+$): Calcd for $C_{17}H_{22}N$, $[M+H]^+$ 240.1752; found 240.1755.

3–Methyl–*N*–(thiophen–2–ylmethyl)pyridin–2–amine (6)

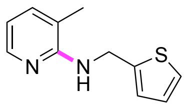

6

Product **6** was prepared according to General Procedure 1 at 60 °C for 1 h using 2–bromo–3–methylpyridine (43 mg, 28 µL, 0.250 mmol), 2–thiophenemethylamine (42.4 mg, 39 µL, 0.375 mmol), 2500 ppm (0.25 mol %) $[Pd(crotyl)Cl]_2$ as catalyst, and 2 mol % BippyPhos (2.5 mg) as ligand. Chromatography conditions: 2–5% EtOAc/hexanes. Yield: 71%, 36.2 mg; yellow oil; R_f = 0.26 (10% EtOAc/hexanes, UV, CAM stain).

^{1}H NMR (500 MHz, $CDCl_3$) δ 8.07 (dd, *J* = 5.2, 1.8 Hz, 1H), 7.26 – 7.19 (m, 2H), 7.04 (dt, *J* = 3.3, 1.1 Hz, 1H), 6.97 (dd, *J* = 5.1, 3.4 Hz, 1H), 6.58 (dd, *J* = 7.1, 5.1 Hz, 1H), 4.87 (dd, *J* = 5.5, 0.9 Hz, 2H), 4.41 (t, *J* = 5.9 Hz, 1H), 2.09 (s, 3H).

^{13}C NMR (126 MHz, CDCl$_3$) δ 156.3, 145.5, 143.2, 137.1, 126.9, 125.6, 124.8, 116.9, 113.4, 40.8, 17.0.

HRMS (ESI$^+$): Calcd for $C_{11}H_{13}N_2S$, $[M+H]^+$ 205.0799; found 205.0802.

***N*–(Furan–2–ylmethyl)–3,5–bis(trifluoromethyl)aniline (7)**

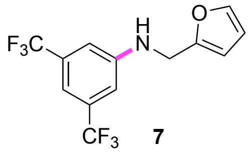

7

Product **7** was prepared according to General Procedure 1 at 60 °C for 1 h using 1–bromo–3,5–bis(trifluoromethyl)benzene (73.2 mg, 43 µL, 0.250 mmol), furfurylamine (36.4 mg, 33µL, 0.375 mmol), 2500 ppm (0.25 mol %) $[Pd(crotyl)Cl]_2$ as catalyst, and 2 mol % BippyPhos (2.5 mg) as ligand. Chromatography conditions: 5–7% EtOAc/hexanes. Yield: 93%, 71.9 mg; red oil ; R_f = 0.27 (10% EtOAc/hexanes, UV, CAM stain).

^{1}H NMR (400 MHz, CDCl$_3$) δ 7.39 (d, *J* = 1.8 Hz, 1H), 7.19 (s, 1H), 7.01 (s, 2H), 6.35 (dd, *J* = 3.3, 1.9 Hz, 1H), 6.28 (d, *J* = 3.2 Hz, 1H), 4.48 (t, *J* = 5.7 Hz, 1H), 4.38 (d, *J* = 5.7 Hz, 2H).

^{13}C NMR (101 MHz, CDCl$_3$) δ 151.0, 148.2, 142.6, 132.58 (q, *J* = 32.8 Hz), 123.66 (q, *J* = 272.5 Hz), 112.35 (d, *J* = 4.4 Hz), 111.00 (p, *J* = 4.0 Hz), 110.6, 108.0, 77.5, 77.4, 77.2, 76.8, 41.0.

^{19}F NMR (376 MHz, CDCl$_3$) δ –63.2.

HRMS (ESI$^+$): Calcd for $C_{13}H_9F_6NO$, $[M+H]^+$ 308.0510; found 308.0497.

***N*–Benzyl–4–(morpholinosulfonyl)aniline (8)**

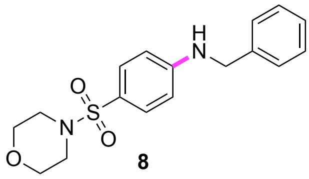

8

Product **8** was prepared according to General Procedure 1 at 60 °C for 1 h using 4–((4–bromophenyl)sulfonyl)morpholine (76.5 mg, 0.250 mmol), benzylamine (40.2 mg, 41 µL, 0.375 mmol), 2500 ppm (0.25 mol %) $[Pd(crotyl)Cl]_2$ as catalyst, and 2 mol % BippyPhos (2.5 mg) as ligand. Chromatography conditions: 30–60% EtOAc/hexanes. Yield: 95%, 79 mg; tan solid ; R_f = 0.35 (50% EtOAc/hexanes, UV, CAM stain).

^{1}H NMR (400 MHz, $CDCl_3$) δ 7.56 – 7.50 (m, 2H), 7.41 – 7.28 (m, 5H), 6.69 – 6.60 (m, 2H), 4.68 (t, *J* = 5.5 Hz, 1H), 4.39 (d, *J* = 5.4 Hz, 2H), 3.76 – 3.69 (m, 4H), 3.00 – 2.91 (m, 4H).

^{13}C NMR (101 MHz, $CDCl_3$) δ 151.8, 138.0, 130.1, 129.0, 127.8, 127.5, 121.9, 112.0, 66.3, 47.8, 46.2.

HRMS (ESI$^+$): Calcd for $C_{17}H_{21}N_2O_3S$, $[M+H]^+$ 333.1273; found 333.1263.

4 –(3–Fluoro–6–methylpyridin–2–yl)morpholine (9)

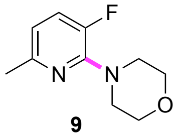

9

Product **9** was prepared according to General Procedure 1 at 60 °C for 1.5 h using 2–bromo–3–fluoro–6–methylpyridine (47.5 mg, 0.250 mmol), morpholine (32.6 mg, 32 µL, 0.375 mmol), 2500 ppm (0.25 mol %) $[Pd(crotyl)Cl]_2$ as catalyst, and 2 mol % BippyPhos (2.5 mg) as ligand. Chromatography conditions: 5–15% EtOAc/hexanes. Yield: 86%, 75.4 mg; light yellow oil; R_f = 0.31 (10% EtOAc/hexanes, UV, CAM stain).

^{1}H NMR (500 MHz, $CDCl_3$) δ 7.10 (dd, *J* = 13.1, 7.9 Hz, 1H), 6.57 (dd, *J* = 7.9, 2.6 Hz, 1H), 3.84 – 3.81 (m, 4H), 3.48 – 3.44 (m, 4H), 2.38 (s, 3H).

^{13}C NMR (126 MHz, $CDCl_3$) δ 151.63 (d, *J* = 5.1 Hz), 149.4, 148.67 (d, *J* = 6.4 Hz), 147.4, 123.7, 123.6, 115.15 (d, *J* = 2.7 Hz), 67.1, 48.18 (d, *J* = 5.6 Hz), 23.77 (d, *J* = 1.6 Hz).

^{19}F NMR (471 MHz, $CDCl_3$) δ –134.5.

HRMS (ESI$^+$): Calcd for $C_{10}H_{14}FN_2O$, $[M+H]^+$ 197.1090; found 197.1081.

1–(3–Nitrophenyl)pyrrolidine (10)

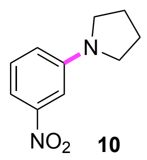

10

Product **10** was prepared according to General Procedure 1 at 60 °C for 30 min using 1–bromo–3–nitrobenzene (50.5 mg, 0.250 mmol), pyrrolidine (26.6 mg, 31 µL, 0.375 mmol), 2500 ppm (0.25 mol %) $[Pd(crotyl)Cl]_2$ as catalyst, and 2 mol % BippyPhos (2.5 mg) as ligand. Chromatography conditions: 1–2% EtOAc/hexanes. Yield: 96%, 46.1 mg; orange solid; R_f = 0.21 (1% EtOAc/hexanes, UV, CAM stain).

^{1}H NMR (400 MHz, $CDCl_3$) δ 7.39 (dd, *J* = 8.1, 2.2 Hz, 1H), 7.26 (d, *J* = 4.2 Hz, 1H), 7.25 – 7.18 (m, 1H), 6.73 (dd, *J* = 8.3, 2.5 Hz, 1H), 3.33 – 3.22 (m, 4H), 2.06 – 1.93 (m, 4H).

^{13}C NMR (101 MHz, $CDCl_3$) δ 149.5, 148.3, 129.6, 117.3, 109.9, 105.7, 47.9, 25.6.

HRMS (ESI$^+$): Calcd for $C_{10}H_{13}N_2O_2$, $[M+H]^+$ 193.0977; found 193.0985.

3–(4–Phenylpiperidin–1–yl)quinoline (11)

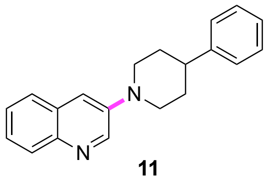

11

Product **11** was prepared according to General Procedure 1 at 60 °C for 2 h using 3–bromoquinoline (52 mg, 34 µL, 0.250 mmol), 4–phenylpiperidine (60.5 mg, 0.375 mmol), 2500 ppm (0.25 mol %) $[Pd(crotyl)Cl]_2$ as catalyst, and 2 mol % BippyPhos (2.5 mg) as ligand. Chromatography conditions: 15–30% EtOAc/hexanes. Yield: 75%, 54 mg; off white solid; R_f = 0.22 (30% EtOAc/hexanes, UV, CAM stain).

^{1}H NMR (400 MHz, $CDCl_3$) δ 8.86 (d, *J* = 2.9 Hz, 1H), 8.00 (d, *J* = 8.1 Hz, 1H), 7.68 (dd, *J* = 7.7, 1.8 Hz, 1H), 7.54 – 7.43 (m, 2H), 7.42 – 7.31 (m, 3H), 7.30 – 7.26 (m, 2H), 7.23 (d, *J* = 7.3 Hz, 1H), 3.94 (dt,

J = 12.6, 3.3 Hz, 2H), 2.95 (td, J = 12.0, 3.5 Hz, 2H), 2.72 (tt, J = 11.4, 4.4 Hz, 1H), 2.02 (qd, J = 10.5, 4.5 Hz, 4H).

^{13}C NMR (101 MHz, CDCl$_3$) δ 145.8, 145.8, 145.4, 142.9, 129.1, 129.1, 128.7, 127.0, 127.0, 126.7, 126.6, 126.4, 117.1, 50.6, 42.4, 33.2.

HRMS (ESI$^+$): Calcd for $C_{20}H_{21}N_2$, $[M+H]^+$ 289.1705; found 289.1704.

1–(*bis*(4–Fluorophenyl)methyl)–4–(3–methoxyphenyl)piperazine (12)

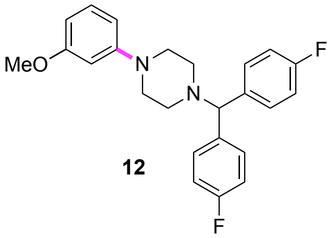

12

Product **12** was prepared according to General Procedure 1 at 60 °C for 45 min using 3–bromoanisole (47 mg, 32 µL, 0.250 mmol), 1–(bis(4–fluorophenyl)methyl)piperazine (108.12 mg, 0.375 mmol), 2500 ppm (0.25 mol %) $[Pd(crotyl)Cl]_2$ as catalyst, and 2 mol % BippyPhos (2.5 mg) as ligand. Chromatography conditions: 5–7% EtOAc/hexanes. Yield: 97%, 95.6 mg; white solid; R_f = 0.29 (10% EtOAc/hexanes, UV, CAM stain).

^{1}H NMR (500 MHz, CDCl$_3$) δ 7.43 – 7.35 (m, 4H), 7.17 (t, J = 8.2 Hz, 1H), 7.04 – 6.96 (m, 4H), 6.53 (dd, J = 8.1, 2.3 Hz, 1H), 6.48 – 6.40 (m, 2H), 4.26 (s, 1H), 3.79 (s, 3H), 3.23 – 3.17 (m, 4H), 2.56 – 2.50 (m, 4H).

^{13}C NMR (126 MHz, CDCl$_3$) δ 163.0, 161.0, 160.7, 152.7, 138.3 (d, J = 3.2 Hz), 129.9, 129.4, 129.3, 115.7, 115.5, 108.8, 104.4, 102.4, 74.6, 55.3, 51.9, 49.2.

^{19}F NMR (471 MHz, CDCl$_3$) δ –115.5.

HRMS (ESI$^+$): Calcd for $C_{24}H_{25}F_2N_2O$, $[M+H]^+$ 395.1935; found 395.1930.

1–(2–Methyl–4–nitrophenyl)–4–(3–(trifluoromethyl)phenyl)piperazine (13)

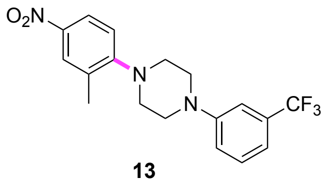

13

Product **13** was prepared according to General Procedure 1 at 60 °C for 1.5 h using 1–bromo–2–methyl–4–nitrobenzene (54 mg, 0.250 mmol), 1–(3–(trifluoromethyl)phenyl)piperazine hydrochloride (100 mg, 0.375 mmol), 2500 ppm (0.25 mol %) [Pd(crotyl)Cl]$_2$ as catalyst, 2 mol % BippyPhos (2.5 mg) as ligand, and KOtBu (112 mg; 4 equiv). Chromatography conditions: 5–10% EtOAc/hexanes. Yield: 71%, 64.9 mg; orange solid; $\mathbf{R_f}$ = 0.16 (5% EtOAc/hexanes, UV, CAM stain).

^{1}H NMR (400 MHz, CDCl$_3$) δ 7.93 – 7.85 (m, 2H), 7.43 – 7.31 (m, 2H), 7.21 – 7.10 (m, 3H), 3.45 – 3.38 (m, 4H), 3.17 – 3.10 (m, 4H), 2.44 (s, 3H).

^{13}C NMR (101 MHz, CDCl$_3$) δ 152.0, 151.4, 147.2, 140.8, 131.7, 131.70 (d, *J* = 32.0 Hz), 129.8, 124.43 (q, *J* = 272.3 Hz), 119.2, 118.5, 116.44 (q, *J* = 3.8 Hz), 114.3, 112.48 (q, *J* = 3.9 Hz), 51.6, 49.3, 18.5.

^{19}F NMR (376 MHz, CDCl$_3$) δ –62.7.

HRMS (ESI$^+$): Calcd for $C_{18}H_{19}F_3N_3O_2$, [M+H]$^+$ 366.1429; found 366.1431.

1–(3,5–bis(Trifluoromethyl)phenyl)–4–ethoxypiperidine (14)

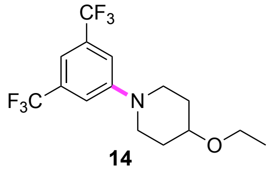

14

Product **14** was prepared according to General Procedure 1 at 60 °C for 2 h using 1–bromo–3,5–bis(trifluoromethyl)benzene (73.2 mg, 43 µL, 0.250 mmol), 4–ethoxypiperidine (48.5 mg, 0.375 mmol), 2500 ppm (0.25 mol %) [Pd(crotyl)Cl]$_2$ as catalyst, and 2 mol % BippyPhos (2.5 mg) as ligand. Chromatography conditions: 1–4% EtOAc/hexanes. Yield: 57%, 48.6 mg; tan solid; $\mathbf{R_f}$ = 0.14 (1% EtOAc/hexanes, UV, CAM stain).

^{1}H NMR (400 MHz, CDCl$_3$) δ 7.24 (d, *J* = 3.3 Hz, 3H), 3.63 – 3.57 (m, 2H), 3.57 – 3.49 (m, 3H), 3.09 (ddd, *J* = 12.6, 9.1, 3.4 Hz, 2H), 2.00 (dddd, *J* = 10.9, 7.3, 3.7, 1.8 Hz, 2H), 1.73 (dtd, *J* = 12.8, 8.6, 3.8 Hz, 2H), 1.23 (t, *J* = 7.0 Hz, 3H).

^{13}C NMR (101 MHz, CDCl$_3$) δ 151.8, 132.42 (q, *J* = 32.6 Hz), 125.1, 122.4, 119.7, 115.0, 111.59 (d, *J* = 3.9 Hz), 73.8, 63.5, 46.3, 30.8, 15.8.

^{19}F NMR (376 MHz, CDCl$_3$) δ –63.1.

HRMS (ESI$^+$): Calcd for $C_{15}H_{18}F_6NO$, $[M+H]^+$ 342.1293; found 342.1301.

1–(4–(Methylsulfonyl)phenyl)–4–(pyrrolidin–1–yl)piperidine (15)

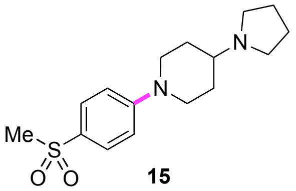

15

Product **15** was prepared according to General Procedure 1 at 60 °C for 45 min using 1–bromo–4–(methylsulfonyl)benzene (58.7 mg, 0.250 mmol), 4–(pyrrolidin–1–yl)piperidine (57.8 mg, 0.375 mmol), 2500 ppm (0.25 mol %) [Pd(crotyl)Cl]$_2$ as catalyst, and 2 mol % BippyPhos (2.5 mg) as ligand. Chromatography conditions: 1–6% MeOH/CH_2Cl_2, 0.5% NH_4OH. Yield: 75%, 57.8 mg; off white solid ; R_f = 0.20 (10% MeOH/CH_2Cl_2, UV, CAM stain).

^{1}H NMR (400 MHz, CDCl$_3$) δ 7.76 – 7.68 (m, 2H), 6.95 – 6.88 (m, 2H), 3.85 (dt, *J* = 13.2, 4.0 Hz, 2H), 3.01 – 2.89 (m, 5H), 2.62 (d, *J* = 6.0 Hz, 4H), 2.26 (tt, *J* = 10.5, 3.7 Hz, 1H), 2.04 – 1.95 (m, 2H), 1.81 (d, *J* = 16.3 Hz, 4H), 1.71 – 1.57 (m, 2H).

^{13}C NMR (126 MHz, CDCl$_3$) δ 154.2, 129.2, 128.0, 114.0, 61.5, 51.6, 46.5, 45.1, 30.8, 23.4.

HRMS (ESI$^+$): Calcd for $C_{16}H_{25}N_2O_2S$, $[M+H]^+$ 309.1637; found 309.1634.

1–(3–(1,3–Dioxolan–2–yl)phenyl)–4–(3–fluoro–5–(trifluoromethyl)pyridin–2–yl)piperazine (16)

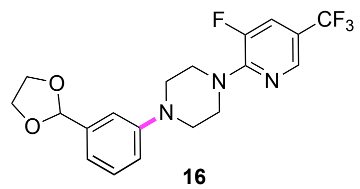

16

Product **16** was prepared according to General Procedure 1 at 60 °C for 1 h using 2–(3–bromophenyl)–1,3–dioxolane (57.2 mg, 38 µL, 0.250 mmol), 1–(3–fluoro–5–(trifluoromethyl)pyridin–2–yl)piperazine (93.4 mg, 0.375 mmol), 2500 ppm (0.25 mol %) $[Pd(crotyl)Cl]_2$ as catalyst, and 2 mol % BippyPhos (2.5 mg) as ligand. Chromatography conditions: 5–15% EtOAc/hexanes. Yield: 92%, 91.4 mg; white solid ; R_f = 0.26 (30% EtOAc/hexanes, UV, CAM stain).

^{1}H NMR (400 MHz, $CDCl_3$) δ 8.28 – 8.23 (m, 1H), 7.41 (dd, *J* = 13.3, 2.0 Hz, 1H), 7.30 (t, *J* = 7.9 Hz, 1H), 7.09 (t, *J* = 2.0 Hz, 1H), 7.04 – 6.93 (m, 2H), 5.79 (s, 1H), 4.18 – 4.11 (m, 2H), 4.08 – 4.01 (m, 2H), 3.86 – 3.79 (m, 4H), 3.36 – 3.29 (m, 4H).

^{13}C NMR (126 MHz, $CDCl_3$) δ 151.4, 151.22 (d, *J* = 5.7 Hz), 149.3, 147.2, 140.43 (p, *J* = 4.7 Hz)f, 139.0, 129.4, 123.57 (q, *J* = 271.0 Hz), 120.48 (dt, *J* = 22.5, 3.3 Hz), 118.4, 117.70 (qd, *J* = 33.7, 2.4 Hz), 117.3, 114.1, 103.9, 65.4, 49.3, 47.06 (d, *J* = 6.6 Hz).

^{19}F NMR (376 MHz, $CDCl_3$) δ –61.1, –127.7.

HRMS (ESI$^+$): Calcd for $C_{19}H_{20}F_4N_3O_2$, $[M+H]^+$ 398.1492; found 398.1473.

***t*–Butyl 4–(2,2–difluorobenzo[d][1,3]dioxol–5–yl)piperazine–1–carboxylate (17)**

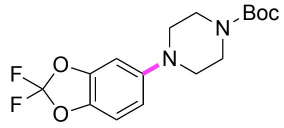

17

Product **17** was prepared according to General Procedure 1 at 60 °C for 1 h using 5–bromo–2,2–difluorobenzo[d][1,3]dioxole (59.2 mg, 34 µL, 0.250 mmol), t–butyl piperazine–1–carboxylate (70 mg, 0.375 mmol), 2500 ppm (0.25 mol %) $[Pd(crotyl)Cl]_2$ as catalyst, and 2 mol % BippyPhos (2.5 mg) as

ligand. Chromatography conditions: 5–20% EtOAc/hexanes. Yield: 99%, 84.7 mg; light pink solid ; R_f = 0.45 (20% EtOAc/hexanes, UV, CAM stain).

^{1}H NMR (400 MHz, CDCl$_3$) δ 6.93 (d, *J* = 8.7 Hz, 1H), 6.70 (d, *J* = 2.4 Hz, 1H), 6.57 (dd, *J* = 8.8, 2.4 Hz, 1H), 3.57 (t, *J* = 5.2 Hz, 4H), 3.03 (t, *J* = 5.1 Hz, 4H), 1.48 (s, 9H).

^{13}C NMR (101 MHz, CDCl$_3$) δ 154.8, 148.9, 144.6, 138.1, 131.90 (t, *J* = 254.4 Hz), 111.7, 109.5, 100.3, 80.2, 50.7, 43.6, 28.5.

^{19}F NMR (376 MHz, CDCl$_3$) δ –50.3.

HRMS (ESI$^+$): Calcd for $C_{16}H_{20}F_2N_2O_4Na$, $[M+Na]^+$ 365.1289; found 365.1277.

***t*–Butyl 4–(7–(1,4–dioxa–8–azaspiro[4.5]decan–8–yl)quinoxalin–2–yl)piperazine–1–carboxylate (18)**

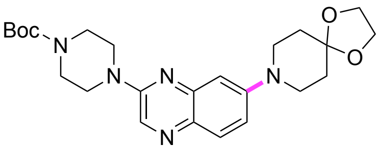

18

Product **18** was prepared according to General Procedure 1 at 60 °C for 45 min using *t*–butyl 4–(7–bromoquinoxalin–2–yl)piperazine–1–carboxylate (98.3 mg, 0.250 mmol), 1,4–dioxa–8–azaspiro[4.5]decane hydrochloride (67.3 mg, 0.375 mmol), 2500 ppm (0.25 mol %) $[Pd(crotyl)Cl]_2$ as catalyst, 2 mol % BippyPhos (2.5 mg) as ligand, and KOtBu (112 mg; 4 equiv). Chromatography conditions: 50–90% EtOAc/hexanes. Yield: 95%, 108.2 mg; yellow solid; R_f = 0.15 (50% EtOAc/hexanes, UV, CAM stain).

^{1}H NMR (400 MHz, CDCl$_3$) δ 8.31 (s, 1H), 7.71 (d, *J* = 9.1 Hz, 1H), 7.16 (dd, *J* = 9.1, 2.7 Hz, 1H), 6.97 (d, *J* = 2.7 Hz, 1H), 4.00 (s, 4H), 3.73 (dd, *J* = 6.7, 3.9 Hz, 4H), 3.59 (dd, *J* = 6.7, 3.8 Hz, 4H), 3.55 – 3.47 (m, 4H), 1.89 – 1.82 (m, 4H), 1.49 (s, 9H).

^{13}C NMR (101 MHz, CDCl$_3$) δ 154.9, 152.9, 152.0, 143.3, 132.0, 131.9, 129.3, 117.3, 108.3, 107.3, 80.3, 64.5, 47.1, 44.8, 34.5, 28.6.

HRMS (ESI$^+$): Calcd for $C_{24}H_{34}N_5O_4$, $[M+H]^+$ 456.2611; found 456.2612.

1–(Benzo[b]thiophen–5–yl)–3–(2–ethylphenoxy)azetidine (19)

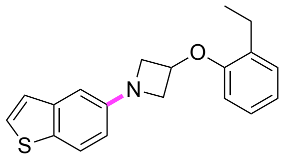

19

Product **19** was prepared according to General Procedure 1 at 60 °C for 45 min using 5–bromobenzothiophene (53.2 mg, 0.250 mmol), 3–(2–ethylphenoxy)azetidine hydrochloride (80.1 mg, 0.375 mmol), 2500 ppm (0.25 mol %) $[Pd(crotyl)Cl]_2$ as catalyst, 2 mol % BippyPhos (2.5 mg) as ligand, and KOtBu (112 mg; 4 equiv). Chromatography conditions: 5–10% EtOAc/hexanes. Yield: 93%, 72 mg; white solid ; R_f = 0.29 (5% EtOAc/hexanes, UV, CAM stain).

^{1}H NMR (400 MHz, $CDCl_3$) δ 7.72 (d, *J* = 8.6 Hz, 1H), 7.41 (d, *J* = 5.4 Hz, 1H), 7.24 – 7.14 (m, 3H), 6.96 (t, *J* = 7.4 Hz, 1H), 6.91 (d, *J* = 2.2 Hz, 1H), 6.69 – 6.61 (m, 2H), 5.14 (p, *J* = 5.2 Hz, 1H), 4.41 (dd, *J* = 7.8, 6.1 Hz, 2H), 3.95 (dd, *J* = 8.0, 4.6 Hz, 2H), 2.67 (q, *J* = 7.5 Hz, 2H), 1.21 (t, *J* = 7.5 Hz, 3H).

^{13}C NMR (101 MHz, $CDCl_3$) δ 154.8, 149.4, 140.7, 133.1, 130.1, 129.6, 127.4, 126.9, 123.4, 122.9, 121.3, 111.6, 111.2, 105.2, 66.6, 59.9, 23.4, 14.3.

HRMS (ESI$^+$): Calcd for $C_{19}H_{20}NOS$, $[M+H]^+$ 310.1266; found 310.1270.

2–Methyl–5–(piperidin–1–yl)benzo[d]oxazole (20)

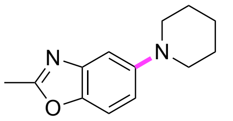

20

Product **20** was prepared according to General Procedure 1 at 65 °C for 1.5 h using 5–chloro–2–methylbenzo[d]oxazole (42 mg, 0.250 mmol), piperidine (31.9 mg, 37 µL, 0.375 mmol), 2500 ppm (0.25 mol %) $[Pd(crotyl)Cl]_2$ as catalyst, 2 mol % BippyPhos (2.5 mg) as ligand. Chromatography conditions:

10–30% EtOAc/hexanes. Yield: 55%, 30 mg; Brown oil ; R_f = 0.20 (30% EtOAc/hexanes, UV, CAM stain).

^{1}H NMR (400 MHz, $CDCl_3$) δ 7.31 (d, *J* = 8.8 Hz, 1H), 7.17 (d, *J* = 2.4 Hz, 1H), 6.96 (dd, *J* = 8.9, 2.4 Hz, 1H), 3.16 – 3.07 (m, 4H), 2.59 (s, 3H), 1.79 – 1.70 (m, 4H), 1.62 – 1.52 (m, 2H).

^{13}C NMR (126 MHz, $CDCl_3$) δ 164.2, 150.7, 145.6, 142.4, 116.3, 110.0, 107.4, 61.7, 52.7, 26.2, 24.3, 14.7.

HRMS (ESI$^+$): Calcd for $C_{13}H_{17}N_2O$, $[M+H]^+$ 217.1341; found 217.1336.

1-(4-(4-(3-Fluoro-5-(trifluoromethyl)pyridin-2-yl)piperazin-1-yl)phenyl)propan-1-one (21)

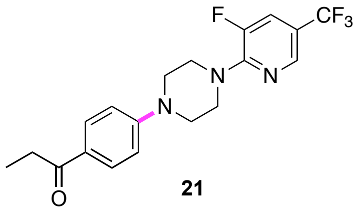

21

Product **21** was prepared according to General Procedure 1 at 65 °C for 1 h using 4-Bromo propiophenone (53.2 mg, 0.250 mmol), 1–(3–fluoro–5–(trifluoromethyl)pyridin–2–yl)piperazine (93.4 mg, 0.375 mmol), 2500 ppm (0.25 mol %) $[Pd(crotyl)Cl]_2$ as catalyst, 2 mol % BippyPhos (2.5 mg) as ligand, and 4 equiv KO*t*Bu. Chromatography conditions: 5–20% EtOAc/hexanes. Yield: 93%, 88.6 mg; Bright yellow solid ; R_f = 0.43 (15% EtOAc/hexanes, UV, CAM stain).

^{1}H NMR (500 MHz, $CDCl_3$) δ 8.25 (s, 1H), 7.94 – 7.89 (m, 2H), 7.42 (dd, *J* = 13.1, 2.1 Hz, 1H), 6.93 – 6.88 (m, 2H), 3.84 (dd, *J* = 6.3, 4.1 Hz, 4H), 3.47 (t, *J* = 5.2 Hz, 4H), 2.93 (q, *J* = 7.3 Hz, 2H), 1.21 (t, *J* = 7.3 Hz, 3H).

^{13}C NMR (126 MHz, $CDCl_3$) δ 199.4, 153.9, 151.03 (d, *J* = 5.7 Hz), 149.2, 147.2, 140.46 (p, *J* = 4.7 Hz), 130.2, 128.0, 123.50 (q, *J* = 271.1 Hz), 120.59 (dq, *J* = 22.3, 3.4 Hz), 118.04 (qd), 113.8, 47.5, 46.67 (d, *J* = 6.5 Hz), 31.3, 8.7.

^{19}F NMR (471 MHz, $CDCl_3$) δ -61.1, -128.0, -128.0.

HRMS (ESI$^+$): Calcd for $C_{19}H_{19}F_4N_3ONa$, $[M+Na]^+$ 404.1362; found 404.1372.

2-(3,3-Difluoropyrrolidin-1-yl)-6-methoxypyridine (22)

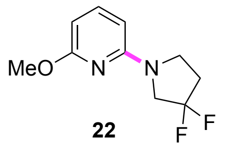

Product **22** was prepared according to General Procedure 1 at 60 °C for 1 h using 2-bromo-6-methoxy pyridine (47 mg, 0.250 mmol), 3,3-difluoropyrrolidine hydrochloride (54 mg, 0.375 mmol), 2500 ppm (0.25 mol %) [Pd(crotyl)Cl]$_2$ as catalyst, 2 mol % BippyPhos (2.5 mg) as ligand and 4 equiv KO*t*Bu. Chromatography conditions: 2–5% EtOAc/hexanes. Yield: 89%, 47.6 mg; red oil ; R_f = 0.42 (5% EtOAc/hexanes, UV, CAM stain).

^{1}H NMR (500 MHz, CDCl$_3$) δ 7.43 (t, *J* = 7.9 Hz, 1H), 6.11 (d, *J* = 7.9 Hz, 1H), 5.91 (d, *J* = 7.9 Hz, 1H), 3.90 (s, 3H), 3.86 (t, *J* = 13.4 Hz, 2H), 3.68 (t, *J* = 7.2 Hz, 2H), 2.49 (tt, *J* = 14.0, 7.2 Hz, 2H).

^{13}C NMR (126 MHz, CDCl$_3$) δ 163.5, 155.8, 139.9, 128.0 (t, *J* = 247.1 Hz), 97.4, 97.3, 54.0 (t, *J* = 31.8 Hz), 53.0, 44.3 (t, *J* = 3.2 Hz), 34.2 (t, *J* = 24.1 Hz).

^{19}F NMR (471 MHz, CDCl$_3$) δ -100.1, -100.1, -100.2, -100.2, -100.2.

HRMS (ESI$^+$): Calcd for $C_{11}H_{12}F_2N_2O$, $[M+H]^+$ 215.0996; found 215.1004.

***t*-Butyl 5-(5-(*tert*-butoxycarbonyl)hexahydropyrrolo[3,4-*c*]pyrrol-2(1*H*)-yl)indoline-1-carboxylate**

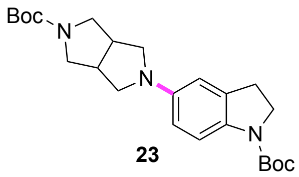

(23)

Product **23** was prepared according to General Procedure 1 at 60 °C for 1 h using *N*-Boc-5-bromoindoline (74.5 mg, 0.250 mmol), *t*-butyl hexahydropyrrolo[3,4-*c*]pyrrole-2(1*H*)-carboxylate (79.6 mg, 0.375 mmol), 2500 ppm (0.25 mol %) [Pd(crotyl)Cl]$_2$ as catalyst, 2 mol % BippyPhos (2.5 mg) as ligand. Chromatography conditions: 15–30% EtOAc/hexanes. Yield: 87%, 93 mg; tan solid ; R_f = 0.13 (15% EtOAc/hexanes, UV, CAM stain).

^{1}H NMR (400 MHz, MeOD) δ 7.65 – 7.20 (m, 1H), 6.49 (d, *J* = 2.5 Hz, 1H), 6.42 – 6.35 (m, 1H), 3.89 (t, *J* = 8.5 Hz, 2H), 3.66 – 3.58 (m, 2H), 3.42 (t, *J* = 8.1 Hz, 2H), 3.27 (dd, *J* = 11.5, 3.8 Hz, 2H), 3.14 (dd, *J* = 9.9, 3.4 Hz, 2H), 3.02 (q, *J* = 7.9 Hz, 4H), 1.54 (s, 9H), 1.45 (s, 9H).

^{13}C NMR (126 MHz, MeOD) δ 156.4, 146.1, 116.1, 111.9, 110.4, 81.0, 54.1, 52.1, 51.7, 43.5, 42.6, 28.8, 28.7.

HRMS (ESI$^+$): Calcd for $C_{24}H_{36}N_3O_4$, $[M+H]^+$ 430.2706; found 430.2708.

Isopropyl 2-(4-(4-((furan-2-ylmethyl)amino)benzoyl)phenoxy)-2-methylpropanoate (24)

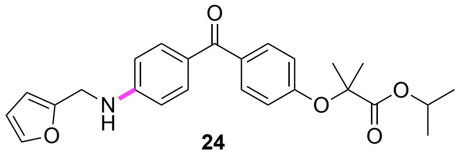

24

Product **24** was prepared according to General Procedure 1 at 60 °C for 1 h using fenofibrate (90.2 mg, 0.250 mmol), furfurylamine (36.4 mg, 0.375 mmol), 2500 ppm (0.25 mol %) $[Pd(crotyl)Cl]_2$ as catalyst, 2 mol % BippyPhos (2.5 mg) as ligand. Chromatography conditions: 15–25% EtOAc/hexanes. Yield: 95%, 100 mg; yellow oil ; R_f = 0.12 (15% EtOAc/hexanes, UV, CAM stain).

^{1}H NMR (400 MHz, $CDCl_3$) δ 7.73 – 7.64 (m, 4H), 7.42 – 7.35 (m, 1H), 6.89 – 6.81 (m, 2H), 6.69 – 6.61 (m, 2H), 6.33 (dd, *J* = 3.2, 1.9 Hz, 1H), 6.25 (d, *J* = 3.2 Hz, 1H), 5.08 (hept, *J* = 6.3 Hz, 1H), 4.60 (t, *J* = 5.7 Hz, 1H), 4.39 (d, *J* = 5.2 Hz, 2H), 1.65 (s, 6H), 1.20 (d, *J* = 6.2 Hz, 6H).

^{13}C NMR (101 MHz, $CDCl_3$) δ 194.3, 173.5, 158.7, 151.8, 151.2, 142.3, 132.7, 132.1, 131.5, 127.3, 117.3, 111.8, 110.5, 107.5, 79.4, 69.3, 40.9, 25.5, 21.6.

HRMS (GC–EI$^+$): Calcd for $C_{25}H_{28}NO_5$, $[M+H]^+$ 422.1967; found 422.1973.

1–(bis(4–Fluorophenyl)methyl)–4–(3–nitrophenyl)piperazine (25)

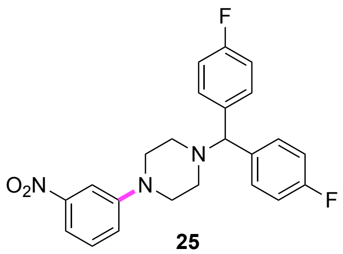

25

Product **25** was prepared according to General Procedure 2 (see SI, section S5 for recycling studies) at 70 °C for 45 min using 1–bromo–3–nitrobenzene (50.5 mg, 0.250 mmol), 1–(bis(4–fluorophenyl)methyl)piperazine (108.12 mg, 0.375 mmol), 2500 ppm (0.25 mol %) $[Pd(crotyl)Cl]_2$ as catalyst, and 2 mol % BippyPhos (2.5 mg) as ligand. Chromatography conditions: 10–20% EtOAc/hexanes. Yield: 99%, 101.3 mg; bright yellow solid ; R_f = 0.27 (15% EtOAc/hexanes, UV, CAM stain).

^{1}H NMR (400 MHz, $CDCl_3$) δ 7.71 – 7.61 (m, 2H), 7.43 – 7.33 (m, 5H), 7.15 (dd, *J* = 8.3, 2.5 Hz, 1H), 7.00 (t, *J* = 8.6 Hz, 4H), 4.28 (s, 1H), 3.31 – 3.24 (m, 4H), 2.58 – 2.51 (m, 4H).

^{13}C NMR (101 MHz, $CDCl_3$) δ 163.3, 160.8, 151.9, 149.4, 138.00 (d, *J* = 3.1 Hz), 129.8, 129.34 (d, *J* = 7.9 Hz), 121.1, 115.8, 115.6, 113.8, 109.6, 74.5, 51.5, 48.6.

^{19}F NMR (376 MHz, $CDCl_3$) δ –115.2.

HRMS (ESI$^+$): Calcd for $C_{23}H_{22}F_2N_3O_2$, $[M+H]^+$ 410.1680; found 410.1660.

(*S*)-4-Fluoro-*N*-methyl-*N*-(3-(naphthalen-1-yloxy)-3-(thiophen-2-yl)propyl)aniline (26)

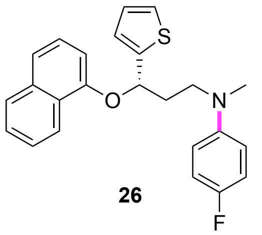

26

Product **26** was prepared according to General Procedure 1 at 60 °C for 1 h using 1-Bromo-4-fluorobenzene (44 mg, 0.250 mmol), duloxetine hydrochloride (125.2 mg, 0.375 mmol), 2500 ppm (0.25 mol %) $[Pd(crotyl)Cl]_2$ as catalyst, 2 mol % BippyPhos (2.5 mg) as ligand and 4 equiv KO*t*Bu. Chromatography

conditions: 5–7% EtOAc/hexanes. Yield: 99%, 97 mg; colorless oil ; R_f = 0.48 (15% EtOAc/hexanes, UV, CAM stain).

^{1}H NMR (400 MHz, $CDCl_3$) δ 8.49 – 8.39 (m, 1H), 7.89 – 7.79 (m, 1H), 7.61 – 7.50 (m, 2H), 7.45 (d, *J* = 8.2 Hz, 1H), 7.33 – 7.29 (m, 1H), 7.27 – 7.24 (m, 1H), 7.09 (dt, *J* = 3.6, 0.9 Hz, 1H), 6.97 (dd, *J* = 5.1, 3.5 Hz, 1H), 6.94 – 6.87 (m, 2H), 6.85 (d, *J* = 7.6 Hz, 1H), 6.72 – 6.62 (m, 2H), 5.75 (dd, *J* = 8.2, 4.5 Hz, 1H), 3.70 – 3.53 (m, 2H), 2.91 (s, 3H), 2.58 – 2.45 (m, 1H), 2.38 (dddd, *J* = 14.1, 8.3, 7.3, 4.5 Hz, 1H).

^{13}C NMR (126 MHz, $CDCl_3$) δ 156.5, 154.6, 153.3, 146.1, 146.1, 145.0, 134.8, 127.7, 126.7, 126.5, 126.2, 125.8, 125.5, 125.0, 124.8, 122.1, 120.9, 115.7, 115.5, 113.90 (d, *J* = 7.3 Hz), 107.1, 74.1, 50.0, 39.1, 36.3.

^{19}F NMR (376 MHz, $CDCl_3$) δ -129.1.

HRMS (ESI$^+$): Calcd for $C_{24}H_{22}FNOSNa$, $[M+Na]^+$ 414.1304; found 414.1333.

(3S,4R)–1–(Benzo[b] thiophen–5–yl)–3–((benzo[d][1,3] dioxol–5–yloxy) methyl)–4–(4–fluorophenyl) piperidine (27)

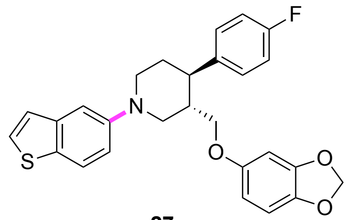

27

Product **27** was prepared according to General Procedure 1 at 70 °C for 45 min using 5–bromobenzothiophene (53.2 mg, 0.250 mmol), paroxetine hydrochloride (137.2 mg, 0.375 mmol), 2500 ppm (0.25 mol %) $[Pd(crotyl)Cl]_2$ as catalyst, 2 mol % BippyPhos (2.5 mg) as ligand, and KOtBu (112 mg; 4 equiv). Chromatography conditions: 5–10% EtOAc/hexanes. Yield: 98%, 112.8 mg; white solid; R_f = 0.38 (10% EtOAc/hexanes, UV, CAM stain).

^{1}H NMR (500 MHz, $CDCl_3$) δ 7.83 (d, *J* = 8.8 Hz, 1H), 7.50 – 7.43 (m, 2H), 7.34 – 7.21 (m, 4H), 7.12 – 7.04 (m, 2H), 6.72 (dd, *J* = 8.5, 2.2 Hz, 1H), 6.46 (d, *J* = 2.5 Hz, 1H), 6.25 (dd, *J* = 8.4, 2.5 Hz, 1H), 5.96 (s, 2H), 4.11 (ddd, *J* = 12.1, 4.0, 1.9 Hz, 1H), 3.89 (ddt, *J* = 12.2, 4.4, 2.3 Hz, 1H), 3.74 (dd, *J* = 9.5, 2.9 Hz, 1H), 3.62 (dd, *J* = 9.4, 7.3 Hz, 1H), 2.99 – 2.86 (m, 2H), 2.70 (td, *J* = 11.8, 4.1 Hz, 1H), 2.46 (dtt, *J* = 10.8, 7.5, 3.3 Hz, 1H), 2.17 – 1.99 (m, 2H).

^{13}C NMR (126 MHz, CDCl$_3$) δ 162.7, 160.8, 154.4, 149.6, 148.3, 141.8, 140.9, 139.56 (d, J = 3.2 Hz), 132.0, 128.96 (d, J = 7.6 Hz), 127.1, 123.9, 122.9, 117.6, 115.7, 115.6, 110.5, 108.0, 105.7, 101.2, 98.1, 69.6, 54.9, 52.0, 44.2, 42.0, 34.4, 22.5, 14.2.

^{19}F NMR (471 MHz, CDCl$_3$) δ –116.2.

HRMS (ESI$^+$): Calcd for $C_{27}H_{25}FNO_3S$, $[M+H]^+$ 462.1539; found 462.1530.

1–(3–(Methyl(3–phenyl–3–(4–(trifluoromethyl)phenoxy)propyl)amino)phenyl)ethan–1–one (28)

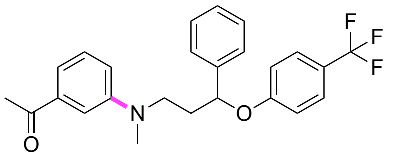

28

Product **28** was prepared according to General Procedure 1 at 70 °C for 45 min using 3–bromoacetophenone (50 mg, 33μL, 0.250 mmol), fluoxetine hydrochloride (130 mg, 0.375 mmol), 2500 ppm (0.25 mol %) $[Pd(crotyl)Cl]_2$ as catalyst, 2 mol % BippyPhos (2.5 mg) as ligand, and KOtBu (112 mg; 4 equiv). Chromatography conditions: 5 – 17.5% EtOAc/hexanes. Yield: 91%, 96.8 mg; yellow oil; R_f = 0.21 (10% EtOAc/hexanes, UV, CAM stain).

^{1}H NMR (400 MHz, CDCl$_3$) δ 7.40 (d, J = 8.5 Hz, 2H), 7.30 (dd, J = 7.3, 1.6 Hz, 4H), 7.25 – 7.16 (m, 4H), 6.91 – 6.80 (m, 3H), 5.17 (dd, J = 8.4, 4.3 Hz, 1H), 3.68 – 3.51 (m, 2H), 2.95 (s, 3H), 2.51 (s, 3H), 2.14 (dqd, J = 14.2, 7.9, 4.6 Hz, 2H).

^{13}C NMR (126 MHz, CDCl$_3$) δ 199.0, 160.4, 149.3, 140.8, 138.2, 129.4, 129.0, 128.1, 126.93 (q, J = 3.8 Hz), 125.8, 124.48 (q, J = 271.1 Hz), 123.08 (q, J = 32.6 Hz), 116.95 (d, J = 3.5 Hz), 115.8, 111.2, 77.9, 49.1, 38.6, 36.1, 26.9.

^{19}F NMR (471 MHz, CDCl$_3$) δ –61.6.

HRMS (ESI$^+$): Calcd for $C_{25}H_{25}F_3NO_2$, $[M+H]^+$ 428.1837; found 428.1817.

2–Chloro–11–(4–(4–nitrophenyl)piperazin–1–yl)dibenzo[b,f][1,4]oxazepane (29)

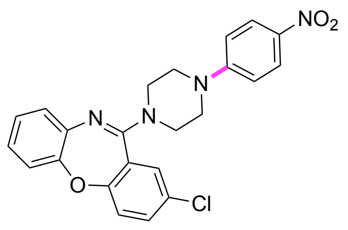

29

Product **29** was prepared according to General Procedure 1 at 70 °C for 45 min using 1–bromo–4–nitrobenzene (50.5 mg, 0.250 mmol), amoxapine (117.6 mg, 0.375 mmol), 2500 ppm (0.25 mol %) $[Pd(crotyl)Cl]_2$ as catalyst, 2 mol % BippyPhos (2.5 mg) as ligand. Chromatography conditions: 60% CH_2Cl_2/hexanes to 2% MeOH/CH_2Cl_2. Yield: 72%, 78.5 mg; yellow solid; R_f = 0.20 (60% CH_2Cl_2/hexanes, UV, CAM stain).

Note: This compound has poor solubility in EtOAc. The first few fractions can be collected using 20% EtOAc/hexanes, but then, CH_2Cl_2/hexanes is required to flush the compound out from the column. Alternatively, precipitation followed by filtration could also be used for isolation.

^{1}H NMR (400 MHz, $CDCl_3$) δ 8.06 (d, J = 9.2 Hz, 2H), 7.33 (dd, J = 8.6, 2.6 Hz, 1H), 7.27 (s, 1H), 7.21 – 6.98 (m, 5H), 6.98 – 6.89 (m, 1H), 6.77 (d, J = 9.1 Hz, 2H), 3.61 (t, J = 5.2 Hz, 4H), 3.46 (s, 4H).

^{13}C NMR (126 MHz, $CDCl_3$) δ 159.6, 158.8, 154.9, 151.9, 139.9, 139.1, 133.0, 130.6, 129.0, 127.3, 126.1, 126.1, 125.2, 124.9, 123.0, 120.4, 113.2, 47.0, 29.9.

HRMS (ESI$^+$): Calcd for $C_{23}H_{20}ClN_4O_3$, $[M+H]^+$ 435.1224; found 435.1233.

8–Chloro–11–(1–(pyridin–3–yl)piperidin–4–ylidene)–6,11–dihydro–5H–benzo[5,6]cyclohepta[1,2–b]pyridine (30)

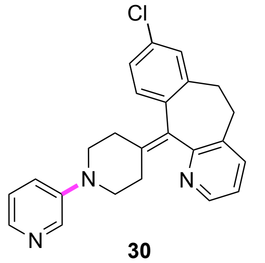

30

Product **30** was prepared according to General Procedure 1 at 70 °C for 45 min using 3–bromopyridine (39.5 mg, 25μL, 0.250 mmol), desloratadine (116.5 mg, 0.375 mmol), 2500 ppm (0.25 mol %) [Pd(crotyl)Cl]$_2$ as catalyst, 2 mol % BippyPhos (2.5 mg) as ligand. Chromatography conditions: 1–5% MeOH/CH_2Cl_2. Yield: 85%, 83 mg; tan solid; R_f = 0.23 (5% MeOH/CH_2Cl_2, UV, CAM stain).

^{1}H NMR (400 MHz, CDCl$_3$) δ 8.42 (dd, *J* = 4.8, 1.7 Hz, 1H), 8.29 (d, *J* = 2.7 Hz, 1H), 8.06 (dd, *J* = 4.3, 1.7 Hz, 1H), 7.44 (dd, *J* = 7.7, 1.7 Hz, 1H), 7.19 – 7.08 (m, 6H), 3.54 (tt, *J* = 11.5, 5.0 Hz, 2H), 3.46 – 3.31 (m, 2H), 3.00 (dddd, *J* = 12.2, 9.6, 5.7, 3.8 Hz, 2H), 2.91 – 2.76 (m, 2H), 2.69 (ddd, *J* = 14.2, 9.7, 4.4 Hz, 1H), 2.61 – 2.44 (m, 3H).

^{13}C NMR (101 MHz, CDCl$_3$) δ 157.3, 146.9, 140.5, 139.7, 138.9, 137.8, 137.7, 137.6, 133.9, 133.5, 133.0, 130.8, 129.1, 126.3, 123.6, 122.7, 122.4, 50.3, 50.2, 31.9, 31.7, 30.6, 30.3.

HRMS (ESI$^+$): Calcd for $C_{24}H_{23}ClN_3$, $[M+H]^+$ 388.1581; found 388.1595.

***t*–Butyl 4–(7–(((1R,5S)–9–methyl–9–azabicyclo[3.3.1]nonan–3–yl)amino)quinoxalin–2–yl)piperazine–1–carboxylate (31):**

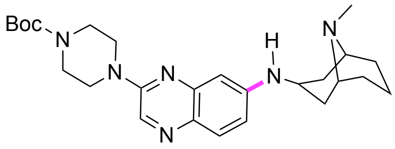

31

Product **31** was prepared according to General Procedure 1 at 60 °C using *t*–butyl 4–(7–bromoquinoxalin–2–yl)piperazine–1–carboxylate (98.2 mg, 0.25 mmol), (*1R, 5S*)–9–methyl–9–azabicyclo[3.3.1]nonan–3–amine (57.84 mg, 0.375 mmol), 1750 ppm (0.175 mol %) [Pd(crotyl)Cl]$_2$ as catalyst, and 2 mol % BippyPhos (2.2 mg) as ligand. Chromatography conditions: 2% / 0.1% / 97.9% MeOH/NH_4OH/CH_2Cl_2. Yield: 96%, 111.1 mg; yellow solid; R_f = 0.30 (2% / 0.5% / 97.5% MeOH/ NH_4OH/CH_2Cl_2).

^{1}H NMR (400 MHz, CDCl$_3$) δ 8.21 (s, 1H), 7.72 – 7.46 (m, 1H), 6.75 (d, *J* = 7.8 Hz, 2H), 4.16 – 3.91 (m, 1H), 3.76 – 3.46 (m, 8H), 3.10 (d, *J* = 10.8 Hz, 2H), 2.62 (td, *J* = 12.4, 6.2 Hz, 2H), 2.51 (s, 3H), 1.99 (dd, *J* = 9.2, 4.1 Hz, 3H), 1.66 (d, *J* = 10.4 Hz, 2H), 1.49 (s, 10H).

^{13}C NMR (101 MHz, CDCl$_3$) δ 154.8, 152.9, 149.2, 144.0, 131.4, 130.3, 129.4, 116.8, 103.1, 80.2, 53.5, 51.4, 44.8, 44.4, 43.5, 40.1, 33.6, 28.5, 23.8, 14.4.

HRMS (ESI$^+$): Calcd for $C_{26}H_{39}N_6O_2$, [M+H]$^+$ 467.3134; found 467.3144.

Isopropyl 2–methyl–2–(4–(4–(4–methylpiperazin–1–yl)benzoyl)phenoxy)propanoate (32)

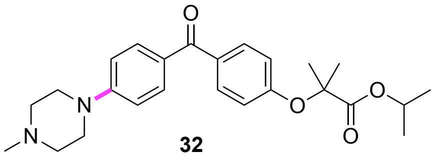

32

Product **32** was prepared according to General Procedure 1 at 60 °C for 45 min using fenofibrate (90.2 mg, 0.250 mmol), 1–methylpiperazine (37.5 mg, 42 µL, 0.375 mmol), 2500 ppm (0.25 mol %) [Pd(crotyl)Cl]$_2$ as catalyst, 2 mol % BippyPhos (2.5 mg) as ligand. Chromatography conditions: 1–4% MeOH/CH_2Cl_2. Yield: 74%, 78.5 mg; yellow oil; R_f = 0.15 (2% MeOH/CH_2Cl_2, UV, CAM stain).

^{1}H NMR (500 MHz, CDCl$_3$) δ 7.78 – 7.64 (m, 4H), 6.94 – 6.80 (m, 4H), 5.08 (hept, *J* = 6.3 Hz, 1H), 3.37 (t, *J* = 5.0 Hz, 4H), 2.57 (t, *J* = 5.1 Hz, 4H), 2.35 (s, 3H), 1.64 (s, 6H), 1.20 (d, *J* = 6.3 Hz, 6H).

^{13}C NMR (126 MHz, CDCl$_3$) δ 194.3, 173.4, 158.9, 153.8, 132.3, 131.9, 131.6, 127.9, 117.3, 113.5, 69.3, 54.8, 47.5, 46.2, 25.5, 21.6.

HRMS (ESI$^+$): Calcd for $C_{25}H_{33}N_2O_4$, [M+H]$^+$ 425.2440; found 425.2443.

***t*–Butyl 4–(6'–Methyl–3–(4–(methylsulfonyl)phenyl)–[2,3'–bipyridin]–5–yl)piperazine–1–carboxylate (33)**

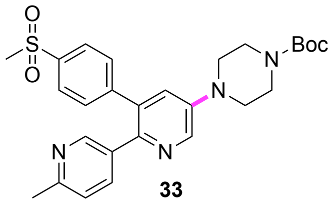

33

Product **33** was prepared according to General Procedure 1 at 60 °C for 45 min using etoricoxib (90 mg, 0.250 mmol), *t*–butyl piperazine–1–carboxylate (139.7 mg, 0.375 mmol), 2500 ppm (0.25 mol %) [Pd(crotyl)Cl]$_2$ as catalyst, 2 mol % BippyPhos (2.5 mg) as ligand. Chromatography conditions: 1–5% MeOH/CH_2Cl_2. Yield: 98%, 124.6 mg; off–white solid; R_f = 0.26 (5% MeOH/CH_2Cl_2, UV, CAM stain).

^{1}H NMR (500 MHz, CDCl$_3$) δ 8.43 (d, *J* = 2.8 Hz, 1H), 8.32 (d, *J* = 2.4 Hz, 1H), 7.89 – 7.84 (m, 2H), 7.53 (dd, *J* = 8.0, 2.4 Hz, 1H), 7.41 – 7.36 (m, 2H), 7.13 (d, *J* = 2.8 Hz, 1H), 7.04 (d, *J* = 8.0 Hz, 1H), 3.62 (t, *J* = 5.1 Hz, 4H), 3.27 (t, *J* = 5.1 Hz, 4H), 3.07 (s, 3H), 2.50 (s, 3H), 1.47 (s, 9H).

^{13}C NMR (126 MHz, CDCl$_3$) δ 157.4, 154.7, 149.9, 145.9, 145.8, 145.0, 139.7, 138.3, 137.3, 134.5, 132.2, 130.6, 127.8, 124.6, 122.8, 80.4, 48.3, 44.6, 43.1, 28.5, 24.3.

HRMS (ESI$^+$): Calcd for $C_{27}H_{33}N_4O_4S$, $[M+H]^+$ 509.2223; found 509.2240.

***N*-(4-(*N*-(Cyclohexylcarbamoyl)sulfamoyl)phenethyl)-5-(3-(2-ethylphenoxy)azetidin-1-yl)-2-methoxybenzamide (34)**

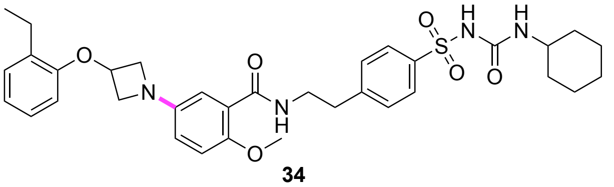

34

Product **34** was prepared according to General Procedure 1 at 60 °C for 45 min using glibenclamide (123.5 mg, 0.250 mmol), 3–(2–ethylphenoxy)azetidine hydrochloride (80.1 mg, 0.375 mmol), 2500 ppm (0.25 mol %) [Pd(crotyl)Cl]$_2$ as catalyst, 2 mol % BippyPhos (2.5 mg) as ligand, and KOtBu (112 mg; 4 equiv).

Chromatography conditions: 1–6% MeOH/CH_2Cl_2. Yield: 70%, 111 mg; off–white solid ; R_f = 0.1 (1% MeOH/CH_2Cl_2, UV, CAM stain).

^{1}H NMR (500 MHz, CDCl$_3$) δ 8.11 – 8.01 (m, 1H), 7.83 (d, *J* = 7.7 Hz, 2H), 7.20 – 7.00 (m, 4H), 6.76 (t, *J* = 9.5 Hz, 1H), 6.55 – 6.48 (m, 2H), 5.76 (s, 1H), 4.99 (t, *J* = 5.8 Hz, 1H), 4.26 (t, *J* = 7.0 Hz, 2H), 3.81 (dd, *J* = 8.1, 4.7 Hz, 2H), 3.73 – 3.52 (m, 6H), 3.25 (s, 1H), 2.79 (d, *J* = 8.0 Hz, 2H), 2.61 (q, *J* = 7.5 Hz, 2H), 1.61 (s, 2H), 1.47 (d, *J* = 11.7 Hz, 2H), 1.40 (d, *J* = 10.8 Hz, 1H), 1.16 (t, *J* = 7.5 Hz, 3H), 1.11 – 1.03 (m, 2H), 0.89 (q, *J* = 13.6 Hz, 3H).

^{13}C NMR (126 MHz, CDCl$_3$) δ 165.5, 164.1, 156.1, 154.7, 150.5, 146.3, 133.0, 132.9, 132.4, 131.7, 129.5, 129.4, 129.0, 126.9, 122.9, 121.9, 121.3, 121.2, 116.0, 115.1, 113.0, 111.4, 111.2, 66.5, 59.9, 56.5, 56.4, 40.9, 35.8, 33.5, 25.7, 25.2, 23.3, 14.2.

HRMS (ESI$^+$): Calcd for $C_{34}H_{43}N_4O_6S$, $[M+H]^+$ 635.2903; found 635.2883.

N–(*t*–Butyl)–4'–((4–oxo–2–propyl–6–(1,4–dioxa–8–azaspiro[4.5]decan–8–yl)quinazolin–3(4H)–yl)methyl)–[1,1'–biphenyl]–2–sulfonamide (35)

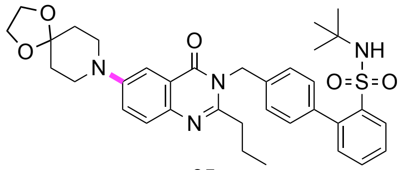

35

Product **35** was prepared according to General Procedure 1 at 70 °C for 45 min using N–(t–butyl)–4'–((6–iodo–4–oxo–2–propylquinazolin–3(4H)–yl)methyl)–[1,1'–biphenyl]–2–sulfonamide (154 mg, 0.250 mmol), 1,4–dioxa–8–azaspiro[4.5]decane hydrochloride (67.3 mg, 0.375 mmol), 2500 ppm (0.25 mol %) $[Pd(crotyl)Cl]_2$ as catalyst, 2 mol % BippyPhos (2.5 mg) as ligand and 4 equiv KO*t*Bu. Chromatography conditions: 0.5–3% MeOH/CH_2Cl_2. Yield: 40%, 63 mg; off white solid; R_f = 0.15 (5% MeOH/CH_2Cl_2, UV, CAM stain).

^{1}H NMR (500 MHz, CDCl$_3$) δ 8.15 (dd, *J* = 7.9, 1.4 Hz, 1H), 7.66 (d, *J* = 3.0 Hz, 1H), 7.60 – 7.53 (m, 2H), 7.50 – 7.42 (m, 4H), 7.31 – 7.26 (m, 2H), 5.45 (s, 2H), 4.00 (s, 4H), 3.50 (s, 1H), 3.49 – 3.43 (m, 4H), 2.77 – 2.68 (m, 2H), 1.90 – 1.78 (m, 6H), 1.25 (s, 2H), 1.02 (t, *J* = 7.4 Hz, 3H), 0.97 (s, 9H).

^{13}C NMR (126 MHz, CDCl$_3$) δ 162.7, 153.9, 149.4, 142.2, 140.7, 139.4, 139.0, 136.9, 132.4, 132.0, 130.5, 128.4, 128.1, 128.1, 126.3, 124.8, 121.1, 110.4, 107.2, 64.5, 54.5, 47.5, 46.4, 37.2, 34.5, 29.9, 21.0, 14.1.

HRMS (ESI$^+$): Calcd for $C_{35}H_{43}N_4O_5S$, $[M+H]^+$ 631.2954; found 631.2941.

3–(4–((4–Chlorophenyl)(phenyl)methyl)piperazin–1–yl)quinoline (36)

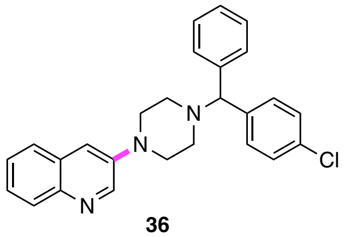

36

Product **36** was prepared according to General Procedure 1 at 70 °C for 45 min using 3–bromoquinoline (52 mg, 34 µL, 0.250 mmol), 1–((4–chlorophenyl)(phenyl)methyl)piperazine (norchlorcyclizine) (107.5 mg, 0.375 mmol), 2500 ppm (0.25 mol %) $[Pd(crotyl)Cl]_2$ as catalyst, and 2 mol % BippyPhos (2.5 mg) as ligand. Chromatography conditions: 10–30% EtOAc/hexanes. Yield: 96%, 99.3 mg; off white solid ; R_f = 0.21 (30% EtOAc/hexanes, UV, CAM stain).

^{1}H NMR (500 MHz, CDCl$_3$) δ 8.78 (d, *J* = 2.9 Hz, 1H), 7.99 (d, *J* = 8.2 Hz, 1H), 7.66 (dd, *J* = 8.1, 1.6 Hz, 1H), 7.53 – 7.45 (m, 2H), 7.42 (dd, *J* = 7.7, 5.1 Hz, 4H), 7.35 – 7.19 (m, 7H), 4.30 (s, 1H), 3.32 (t, *J* = 5.0 Hz, 4H), 2.62 (dd, *J* = 6.3, 3.8 Hz, 4H).

^{13}C NMR (126 MHz, CDCl$_3$) δ 144.9, 144.8, 143.0, 142.0, 141.2, 132.9, 129.3, 129.0, 129.0, 128.9, 128.9, 127.9, 127.5, 127.0, 126.7, 126.5, 116.6, 75.5, 51.7, 49.4.
Spectral data matches that previously reported in the literature.[4]

(3S,4R)–3–((Benzo[d][1,3]dioxol–5–yloxy)methyl)–4–(4–fluorophenyl)–1–(4–methoxyphenyl)piperidine (37)

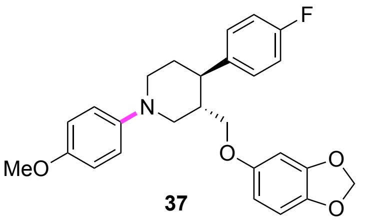

37

Product **37** was prepared according to General Procedure 1 at 60 °C for 45 min using 4–bromoanisole (46.7 mg, 31 µL, 0.250 mmol), paroxetine hydrochloride (137.2 mg, 0.375 mmol), 2500 ppm (0.25 mol %)

[Pd(crotyl)Cl]$_2$ as catalyst, and 2 mol % BippyPhos (2.5 mg) as ligand and 4 equiv KO*t*Bu. Chromatography conditions: 5–15% EtOAc/hexanes. Yield: 91%, 99.1 mg; light pink fluffy solid; R_f = 0.40 (30% EtOAc/hexanes, CAM stain).

^{1}H NMR (500 MHz, CDCl$_3$) δ 7.23 – 7.17 (m, 2H), 7.00 (dt, *J* = 8.7, 4.1 Hz, 4H), 6.91 – 6.84 (m, 2H), 6.64 (d, *J* = 8.5 Hz, 1H), 6.37 (d, *J* = 2.5 Hz, 1H), 6.16 (dd, *J* = 8.5, 2.5 Hz, 1H), 5.89 (s, 2H), 3.87 (ddd, *J* = 11.9, 3.9, 1.8 Hz, 1H), 3.79 (s, 3H), 3.64 (dt, *J* = 8.3, 4.1 Hz, 2H), 3.53 (dd, *J* = 9.4, 7.4 Hz, 1H), 2.82 – 2.68 (m, 2H), 2.58 (td, *J* = 11.8, 4.2 Hz, 1H), 2.36 (dtt, *J* = 10.6, 7.4, 3.0 Hz, 1H), 2.07 – 1.89 (m, 2H).

^{13}C NMR (126 MHz, CDCl$_3$) δ 162.7, 160.7, 154.4, 154.0, 148.3, 146.2, 141.7, 139.7 (d, *J* = 3.2 Hz), 129.0 (d, *J* = 7.8 Hz), 119.2, 115.7, 115.5, 114.6, 108.0, 105.7, 101.2, 98.1, 69.6, 55.7, 55.5, 52.3, 44.1, 42.2, 34.6.

^{19}F NMR (471 MHz, CDCl$_3$) δ –116.3.

Spectral data matches previously reported literature.[4]

7-(6-Methoxynaphthalen-2-yl)-3-(trifluoromethyl)-5,6,7,8-tetrahydro-[1,2,4]triazolo[4,3-*a*]pyrazine (38)

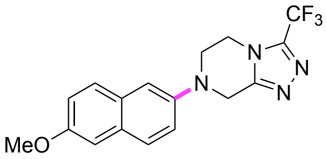

38

Product **38** was prepared according to General Procedure 1 at 60 °C for 1 h using 2–bromo-6-methoxynaphthalene (59.2 mg, 0.250 mmol), 3-(trifluoromethyl)-5,6,7,8-tetrahydro-[1,2,4]triazolo[4,3-*a*]pyrazine (72 mg, 0.375 mmol), 2500 ppm (0.25 mol %) [Pd(crotyl)Cl]$_2$ as catalyst, and 2 mol % BippyPhos (2.5 mg) as ligand. Chromatography conditions: 2–5% MeOH/CH_2Cl_2. Yield: 87%, 75.7 mg; off White solid solid ; R_f = 0.27 (2% MeOH/CH_2Cl_2, CAM stain).

^{1}H NMR (500 MHz, CDCl$_3$) δ 7.80 (d, *J* = 8.9 Hz, 1H), 7.71 (d, *J* = 8.9 Hz, 1H), 7.32 (dd, *J* = 9.7, 3.0 Hz, 1H), 7.23 (dd, *J* = 9.1, 2.5 Hz, 2H), 7.17 (s, 1H), 4.75 (s, 2H), 4.34 (t, *J* = 5.4 Hz, 2H), 3.98 (s, 3H), 3.85 (t, *J* = 5.4 Hz, 2H).

^{13}C NMR (126 MHz, CDCl$_3$) δ 157.1, 151.8, 144.6, 143.7 (q, *J* = 39.9 Hz), 130.6, 129.5, 128.6, 120.1, 119.7, 112.9, 106.0, 55.5, 47.6, 46.9, 43.4.

^{19}F NMR (471 MHz, $CDCl_3$) δ -63.0.

Spectral data matches previously reported literature.[4]

2–Chloro–11–(4–(4–(trifluoromethyl)phenyl)piperazin–1–yl)dibenzo[b,f][1,4]oxazepane (39)

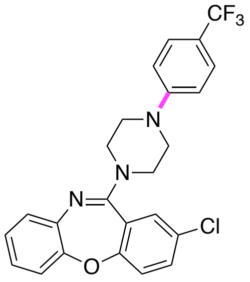

39

Product **39** was prepared according to General Procedure 1 at 70 °C for 45 min using 4–bromobenzotrifluoride (56.3 mg, 35µL, 0.250 mmol), amoxapine (117.6 mg, 0.375 mmol), 2500 ppm (0.25 mol %) $[Pd(crotyl)Cl]_2$ as catalyst, and 2 mol % BippyPhos (2.5 mg) as ligand. Chromatography conditions: 5–10% EtOAc/hexanes. Yield: 82%, 93.8 mg; white solid; R_f = 0.16 (5% EtOAc/hexanes, UV, CAM stain).

^{1}H NMR (400 MHz, $CDCl_3$) δ 7.52 (d, J = 8.6 Hz, 2H), 7.42 (dd, J = 8.6, 2.6 Hz, 1H), 7.36 (d, J = 2.6 Hz, 1H), 7.23 – 7.16 (m, 2H), 7.11 (td, J = 7.7, 1.3 Hz, 2H), 7.03 (dd, J = 7.2, 2.0 Hz, 1H), 6.97 (d, J = 8.5 Hz, 2H), 3.69 (t, J = 5.2 Hz, 4H), 3.40 (s, 4H).

^{13}C NMR (126 MHz, $CDCl_3$) δ 159.5, 159.0, 153.3, 151.9, 140.1, 132.9, 130.5, 129.1, 127.3, 126.6 (q, J = 3.7 Hz), 126.0, 125.0, 124.8 (q, J = 270.1 Hz), 123.0, 121.3 (q, J = 32.6 Hz), 120.3, 115.1, 48.1, 47.3.

^{19}F NMR (376 MHz, $CDCl_3$) δ –61.4.

Spectral data matches that previously reported in the literature.[5]

1-(4-(2-Methoxyphenyl)piperazin-1-yl)-3-(naphthalen-1-yloxy)propan-2-ol (Naftopidil; 43)

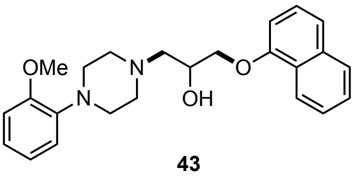

43

Product **43** was prepared according to General Procedure 3 at 60 °C. Chromatography conditions: 1–5% MeOH/CH_2Cl_2. Yield: 67%, 66 mg; off–white solid; R_f = 0.16 (2% MeOH/CH_2Cl_2, UV, CAM stain).

^{1}H NMR (500 MHz, $CDCl_3$) δ 8.30 – 8.25 (m, 1H), 7.83 – 7.77 (m, 1H), 7.52 – 7.43 (m, 3H), 7.38 (t, *J* = 7.9 Hz, 1H), 7.05 – 6.99 (m, 1H), 6.98 – 6.92 (m, 2H), 6.90 – 6.84 (m, 2H), 4.31 (dt, *J* = 7.5, 5.2 Hz, 1H), 4.24 (dd, *J* = 9.5, 5.1 Hz, 1H), 4.18 (dd, *J* = 9.5, 4.9 Hz, 1H), 3.88 (s, 3H), 3.15 (s, 4H), 2.99 – 2.91 (m, 2H), 2.78 – 2.70 (m, 4H).

^{13}C NMR (126 MHz, $CDCl_3$) δ 154.5, 152.4, 141.2, 134.6, 127.6, 126.5, 125.9, 125.7, 125.3, 123.2, 122.0, 121.1, 120.7, 118.3, 111.3, 105.0, 70.7, 65.7, 61.1, 55.5, 53.7, 50.8.

Spectral data matches that previously reported in the literature.[3]

10. NMR spectra of intermediates and products

NMR spectra of starting materials and intermediates

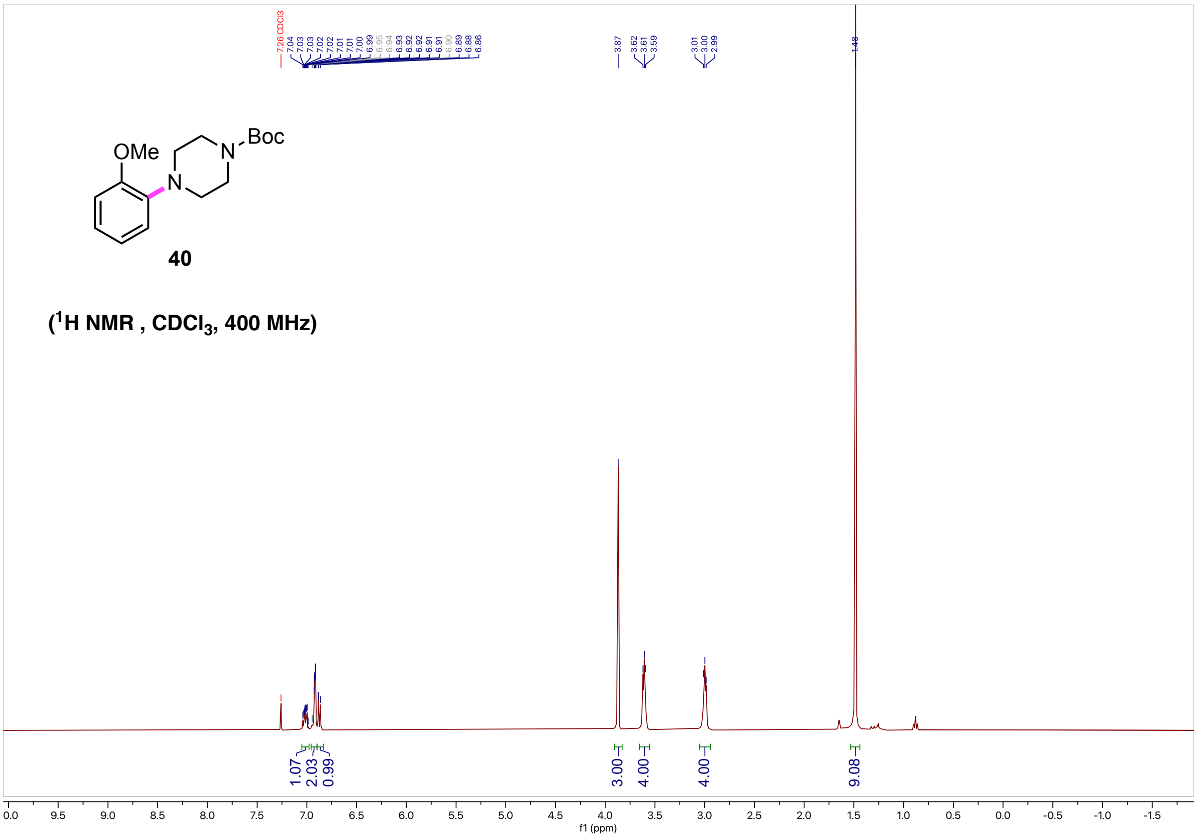

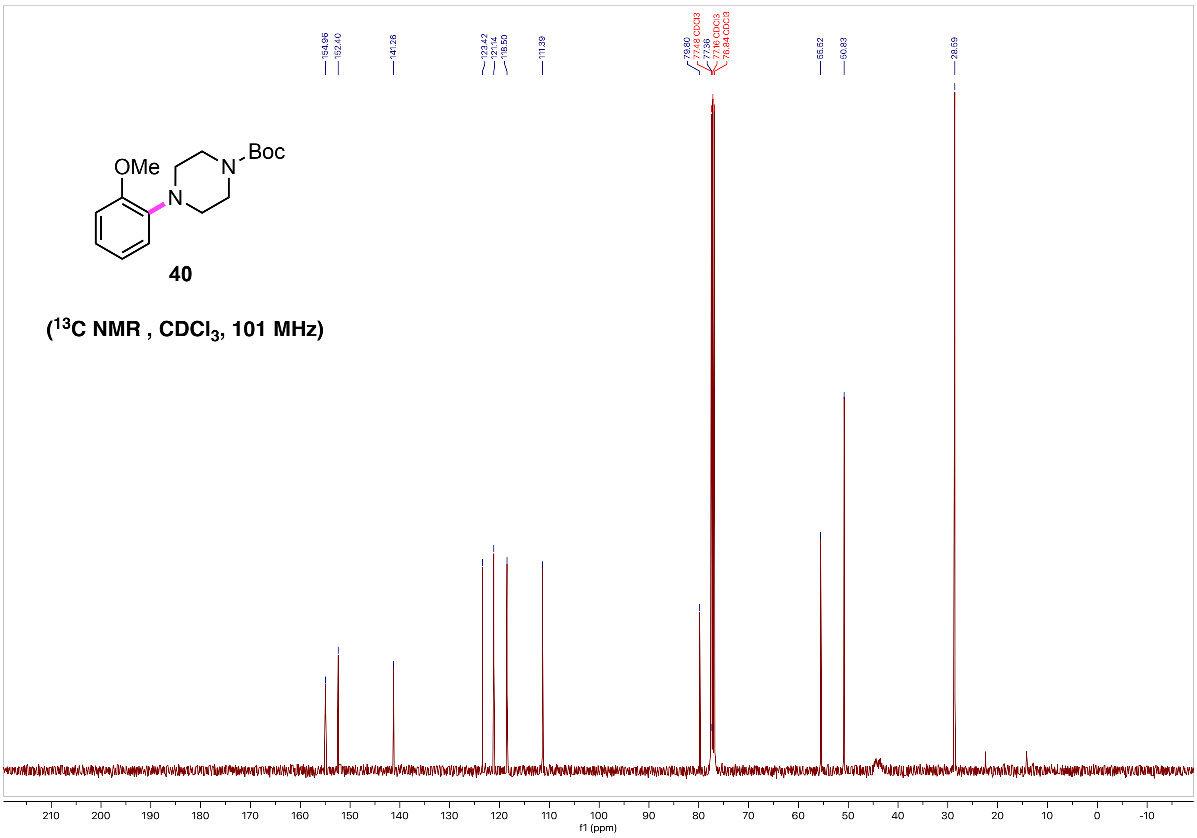

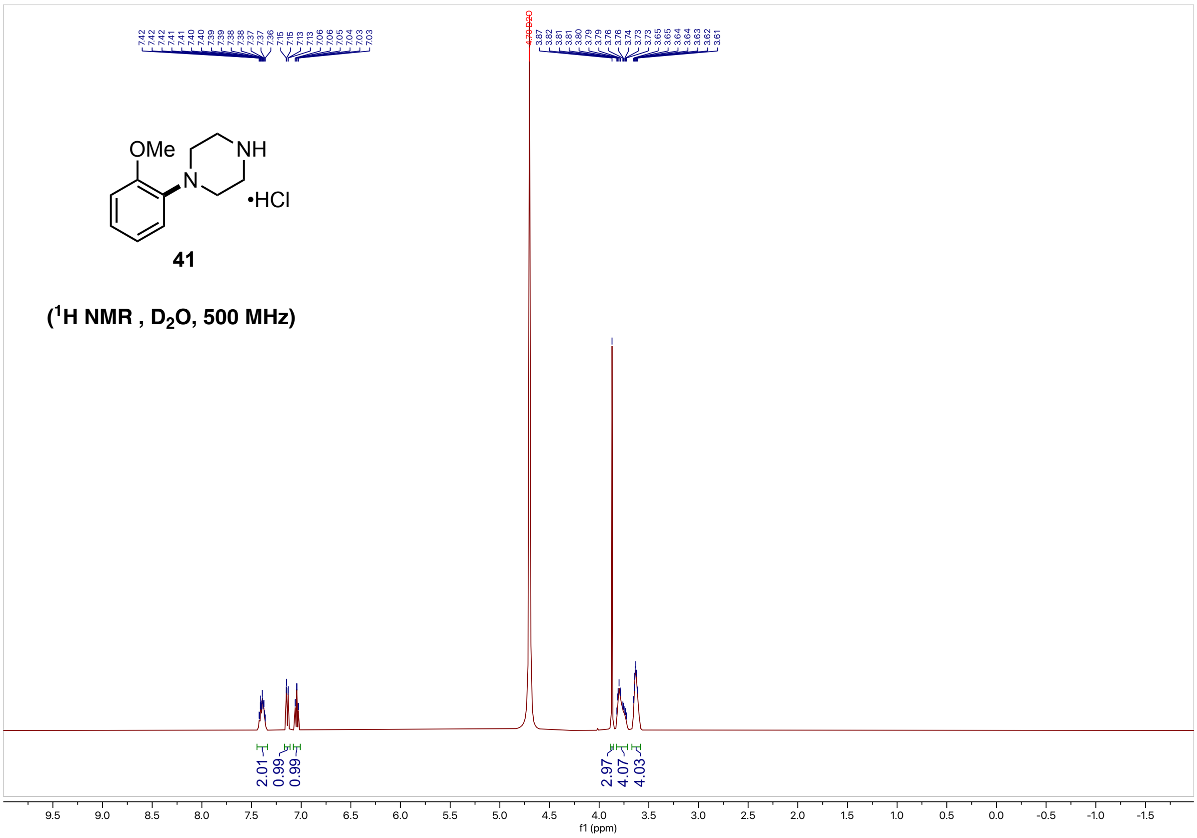
7.42 7.42 7.41 7.41 7.40 7.39 7.39 7.38 7.38 7.37 7.37 7.36 7.15 7.15 7.13 7.13 7.06 7.06 7.05 7.04 7.03 7.03
4.70 D2O
3.87 3.82 3.81 3.81 3.80 3.79 3.79 3.76 3.76 3.74 3.73 3.73 3.65 3.65 3.64 3.64 3.63 3.62 3.61
OMe
NH
N
•HCl
41
(1H NMR , D2O, 500 MHz)
2.01
0.99
0.99
2.97
4.07
4.03
f1 (ppm)

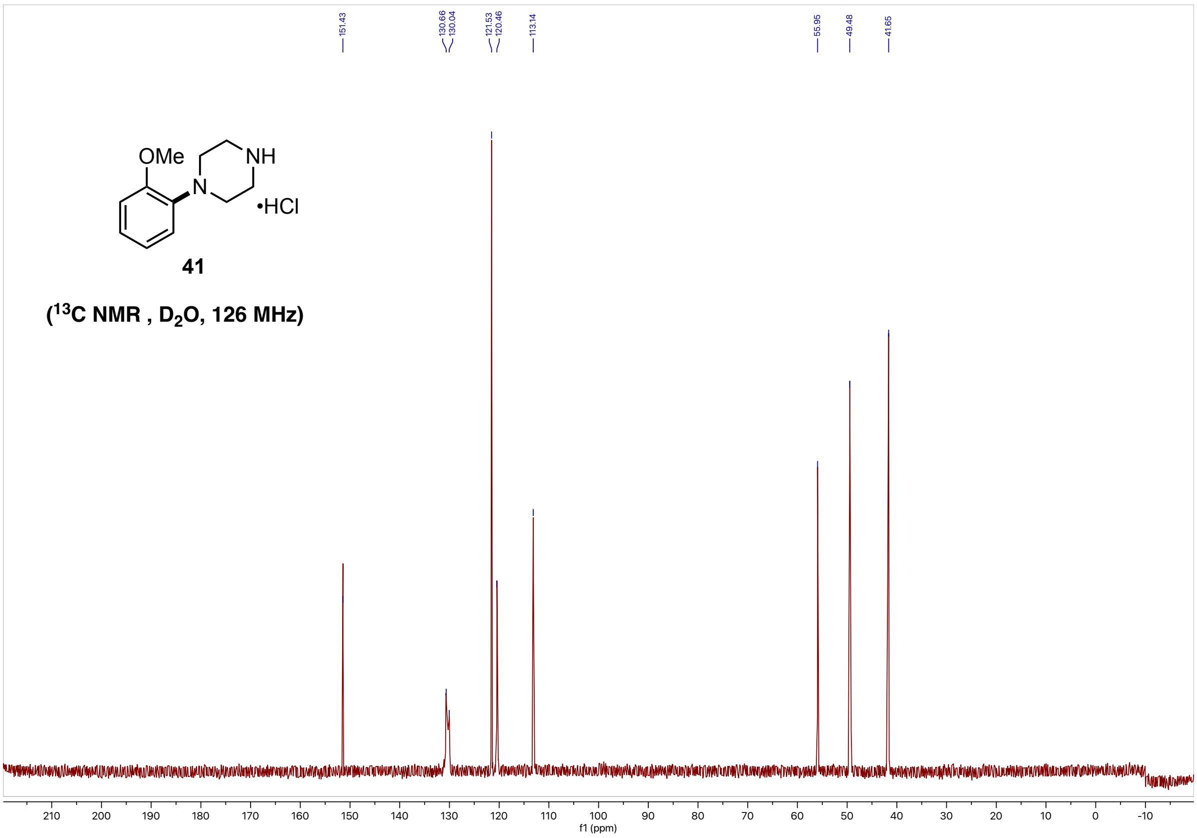
151.43
130.66
130.04
121.53
120.46
113.14
55.95
49.48
41.65
OMe
NH
N
•HCl
41
(13C NMR , D2O, 126 MHz)
f1 (ppm)

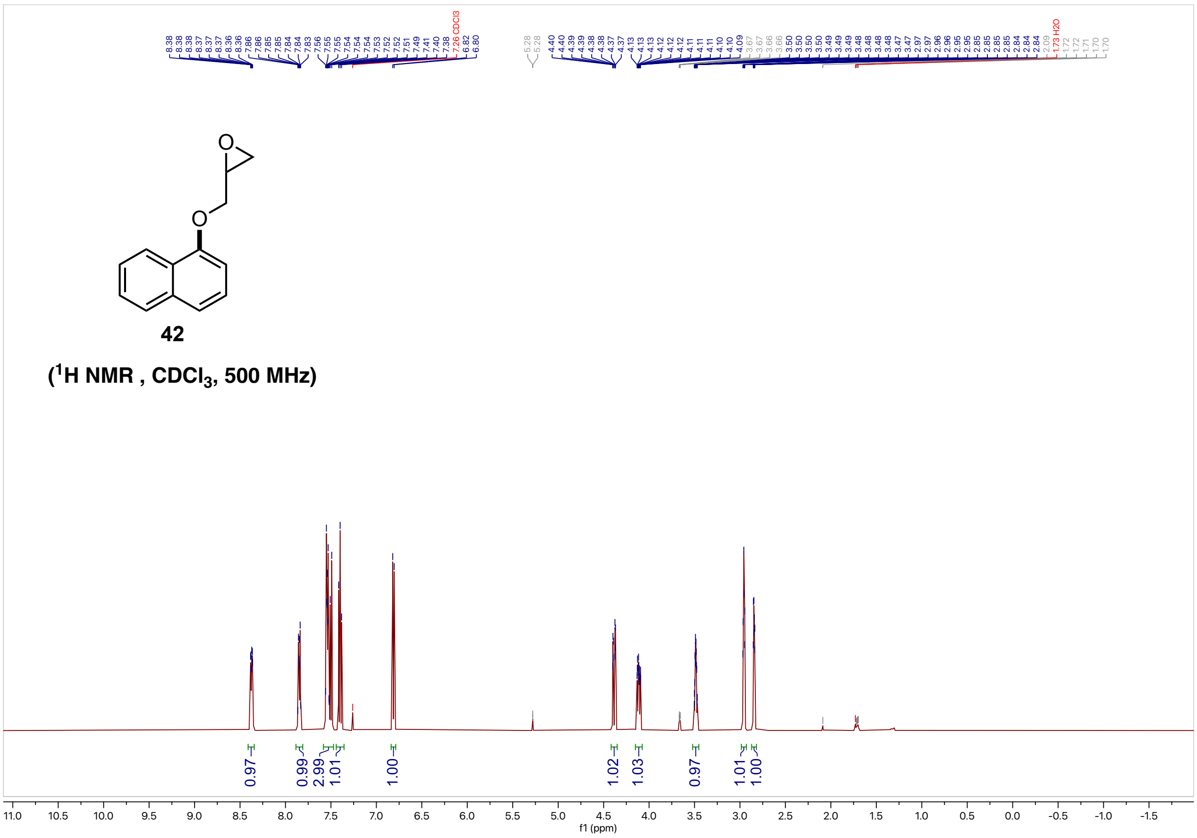
42
(^{1}H NMR , $CDCl_3$, 500 MHz)
f1 (ppm)

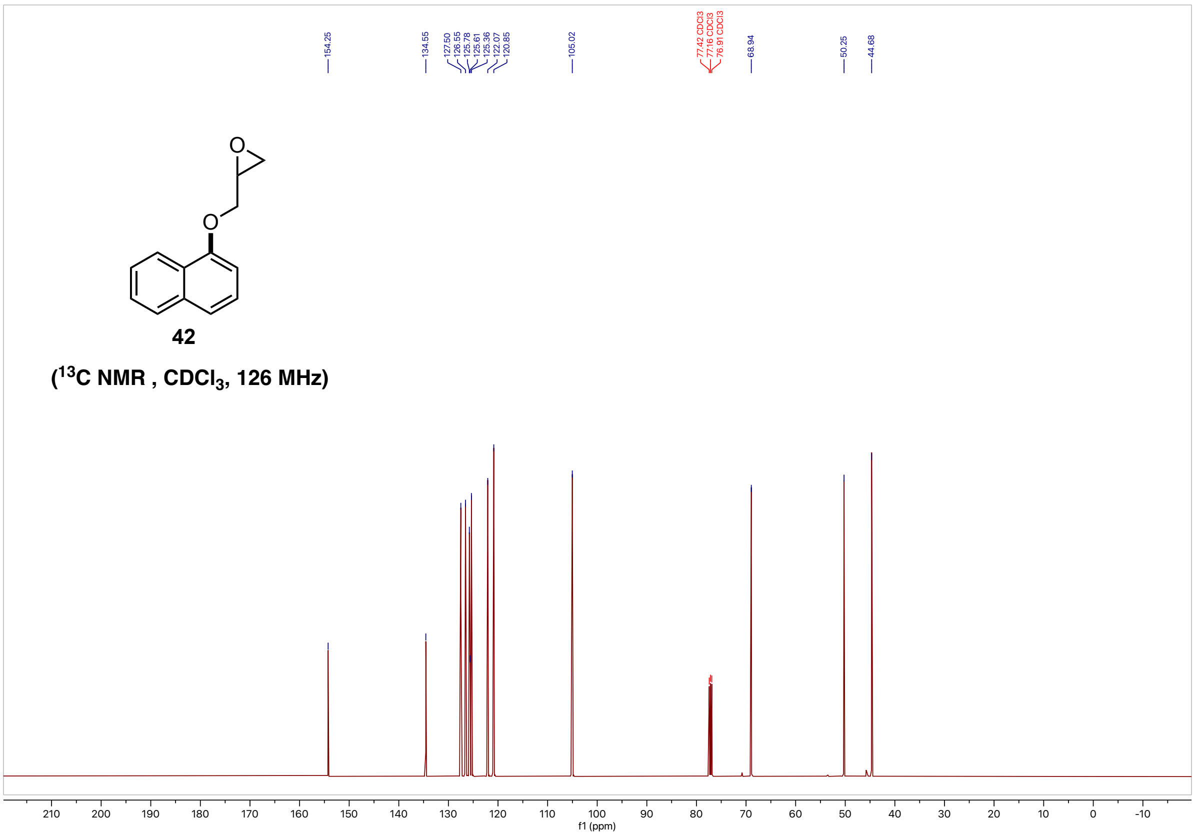
42
(^{13}C NMR , $CDCl_3$, 126 MHz)
f1 (ppm)

NMR spectra for products

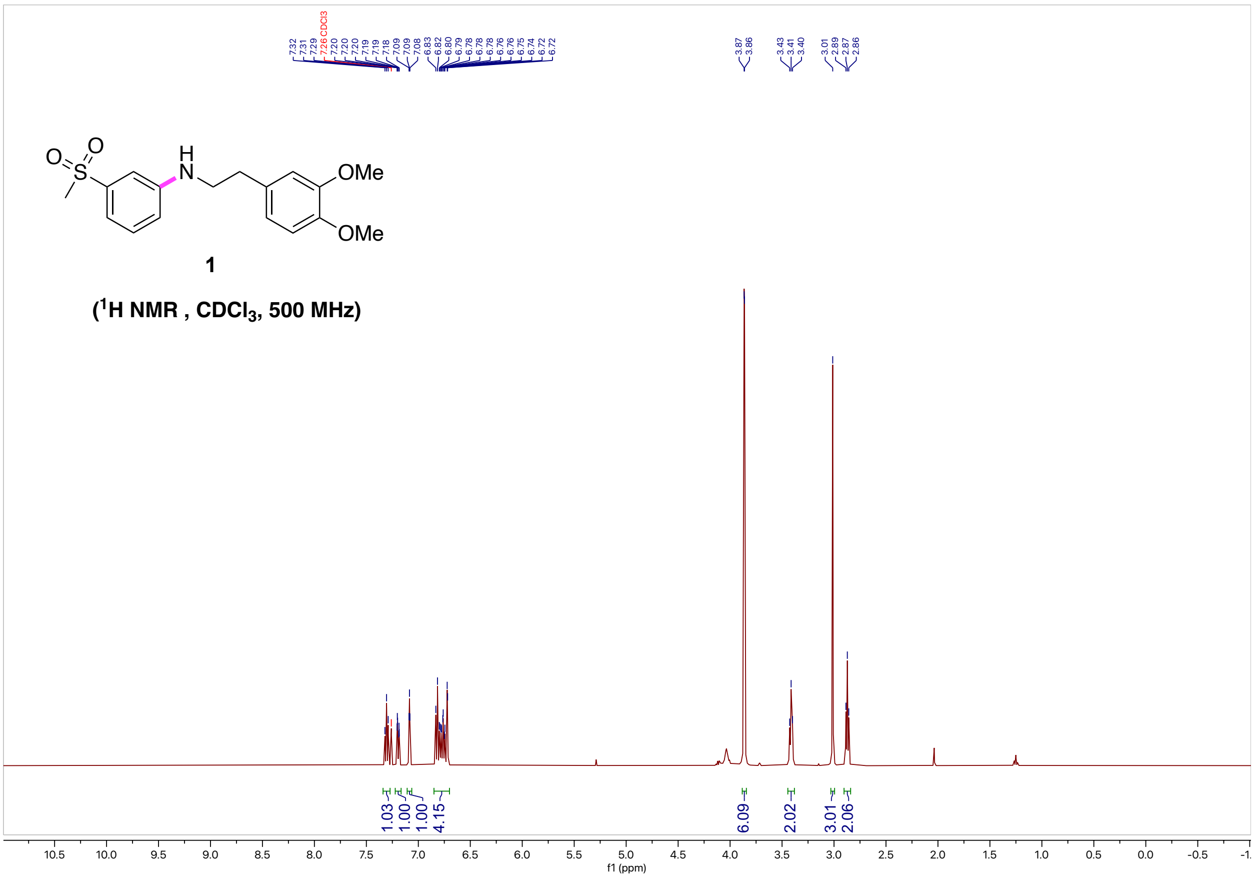

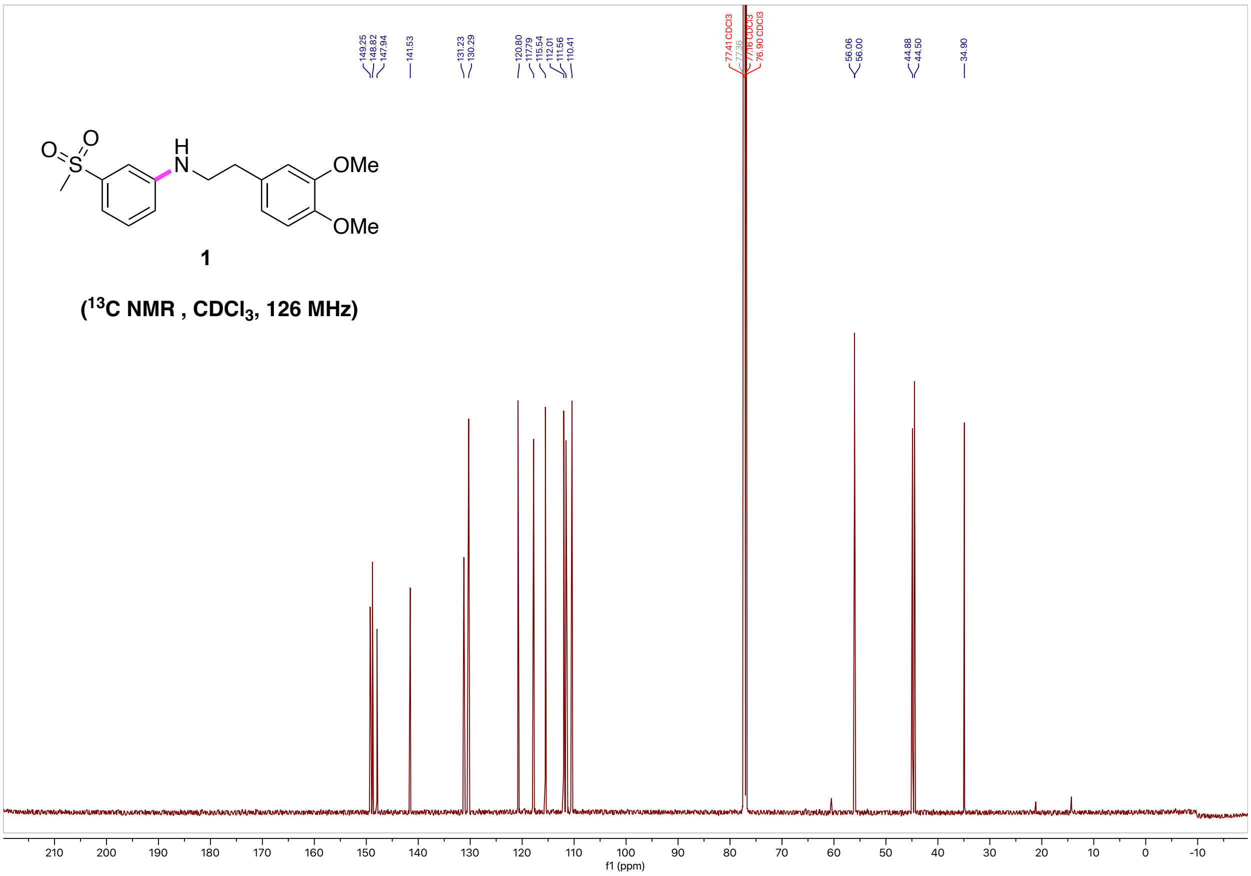

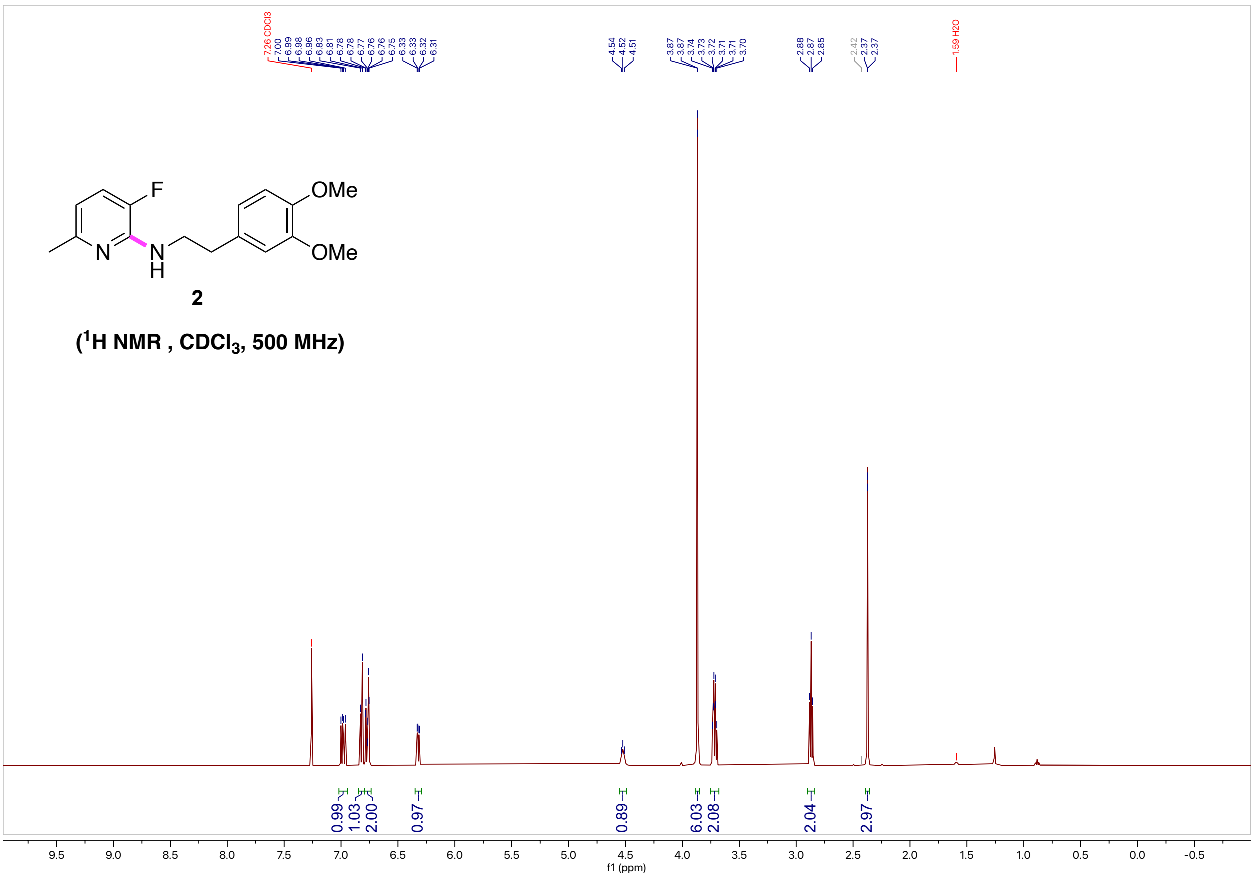

F
OMe
N
N
H
OMe
2
(¹H NMR , CDCl₃, 500 MHz)
f1 (ppm)

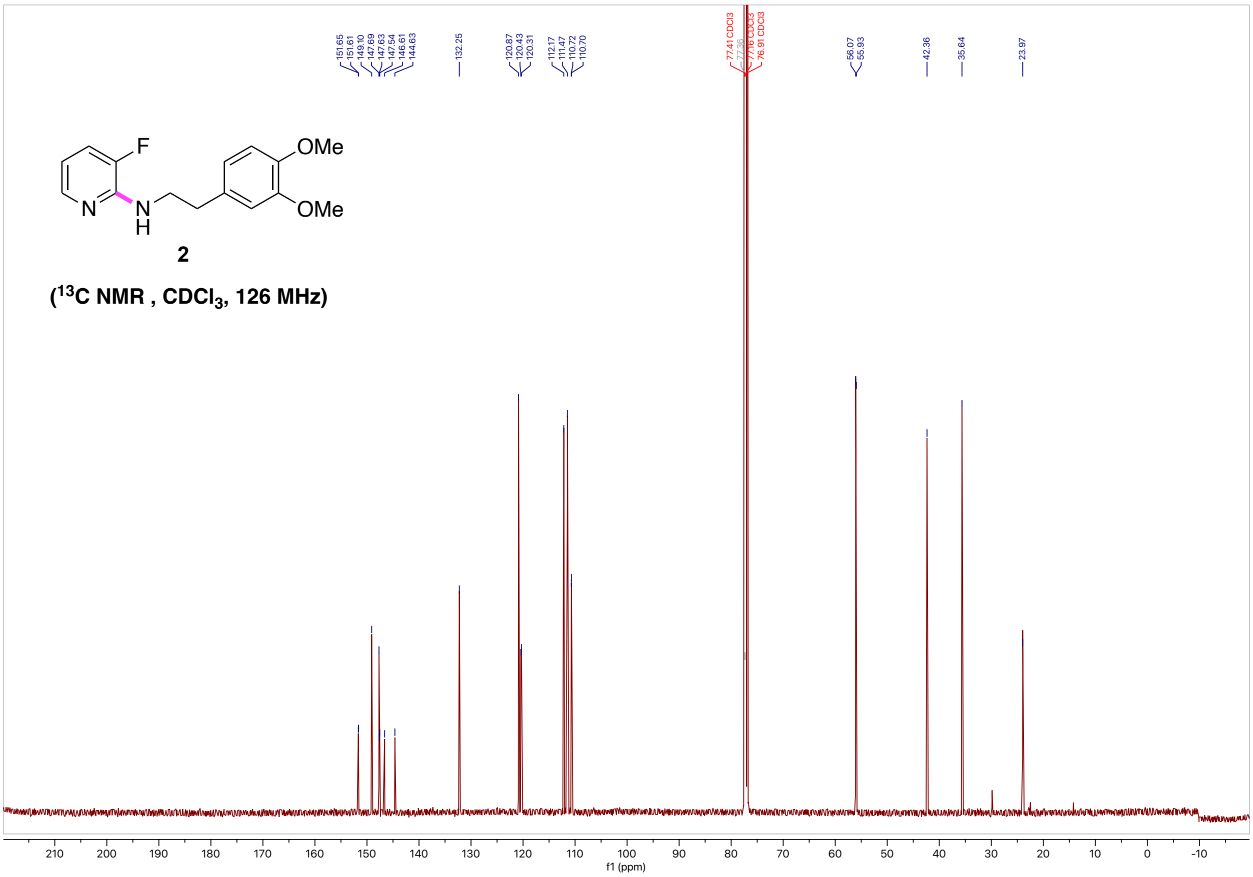

F
OMe
N
N
H
OMe
2
(¹³C NMR , CDCl₃, 126 MHz)
f1 (ppm)

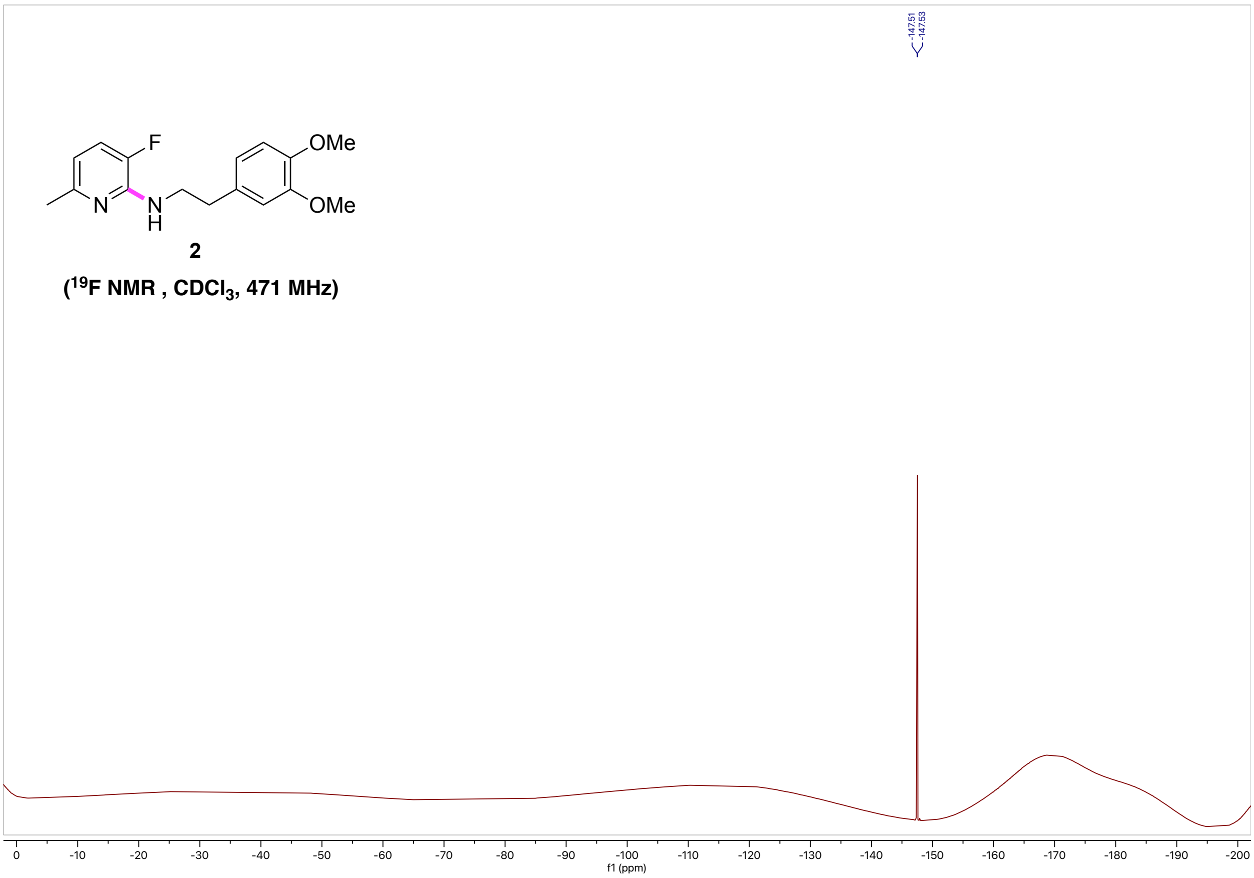

-147.51
-147.53
F
OMe
N
N
H
OMe
2
(19F NMR , CDCl3, 471 MHz)
0
-10
-20
-30
-40
-50
-60
-70
-80
-90
-100
-110
-120
-130
-140
-150
-160
-170
-180
-190
-200
f1 (ppm)

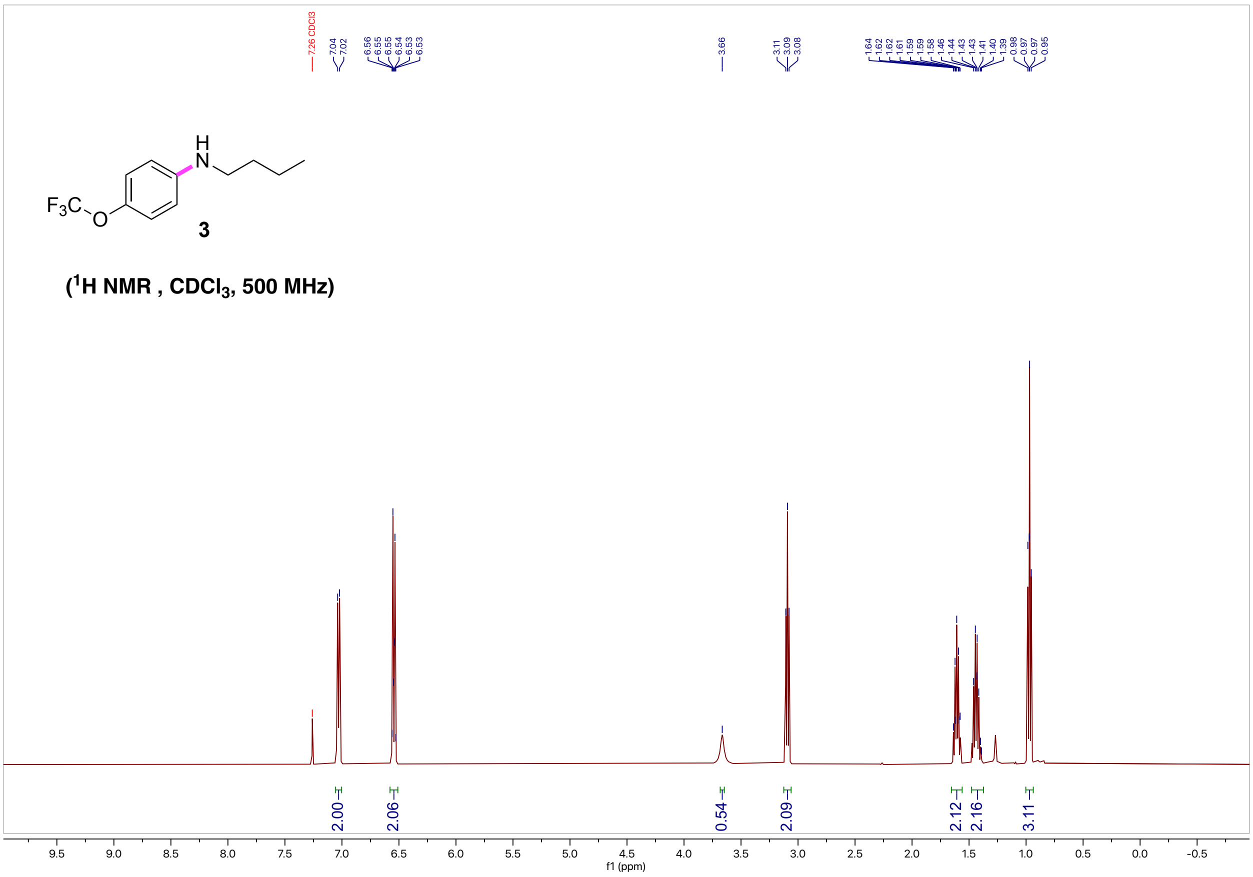
3
(1H NMR , CDCl3, 500 MHz)
f1 (ppm)

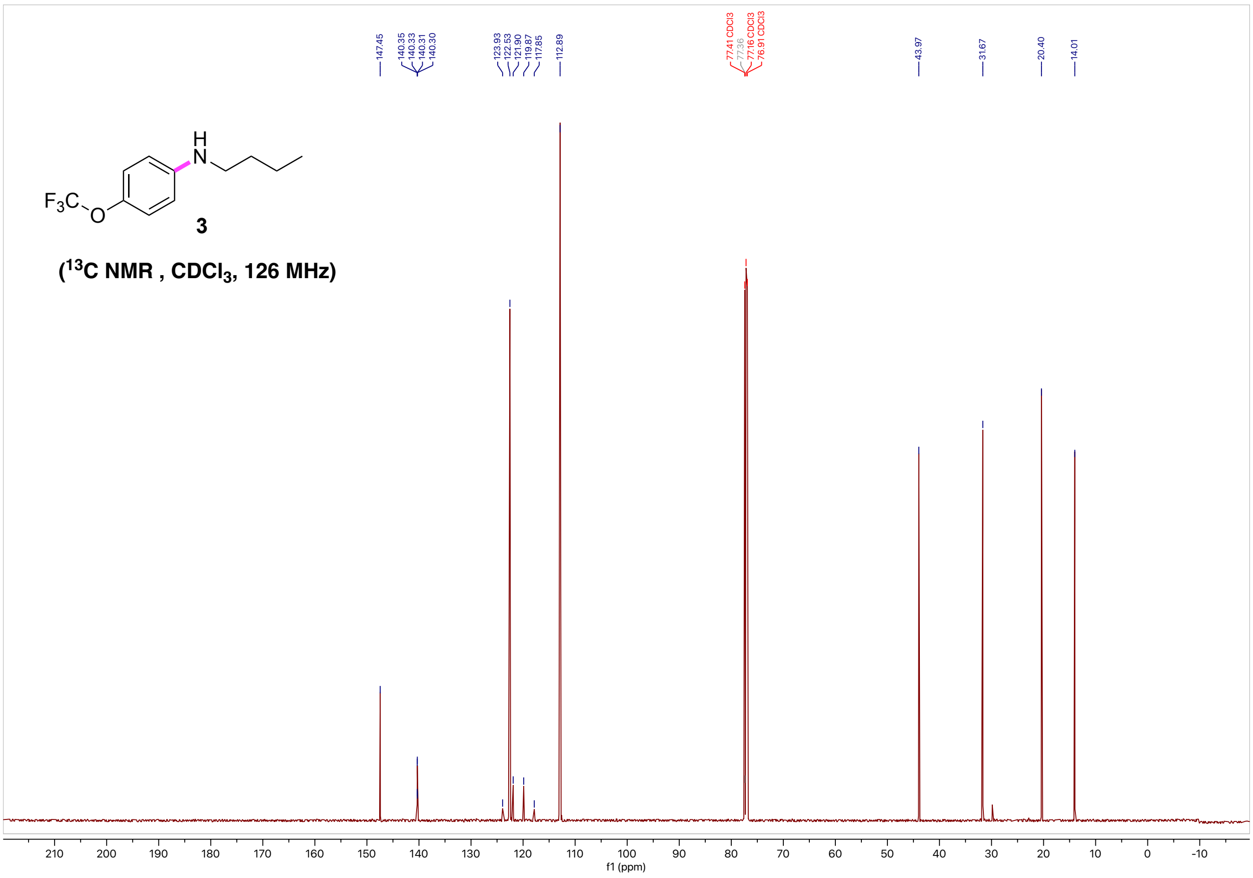
3
(13C NMR , CDCl3, 126 MHz)
f1 (ppm)

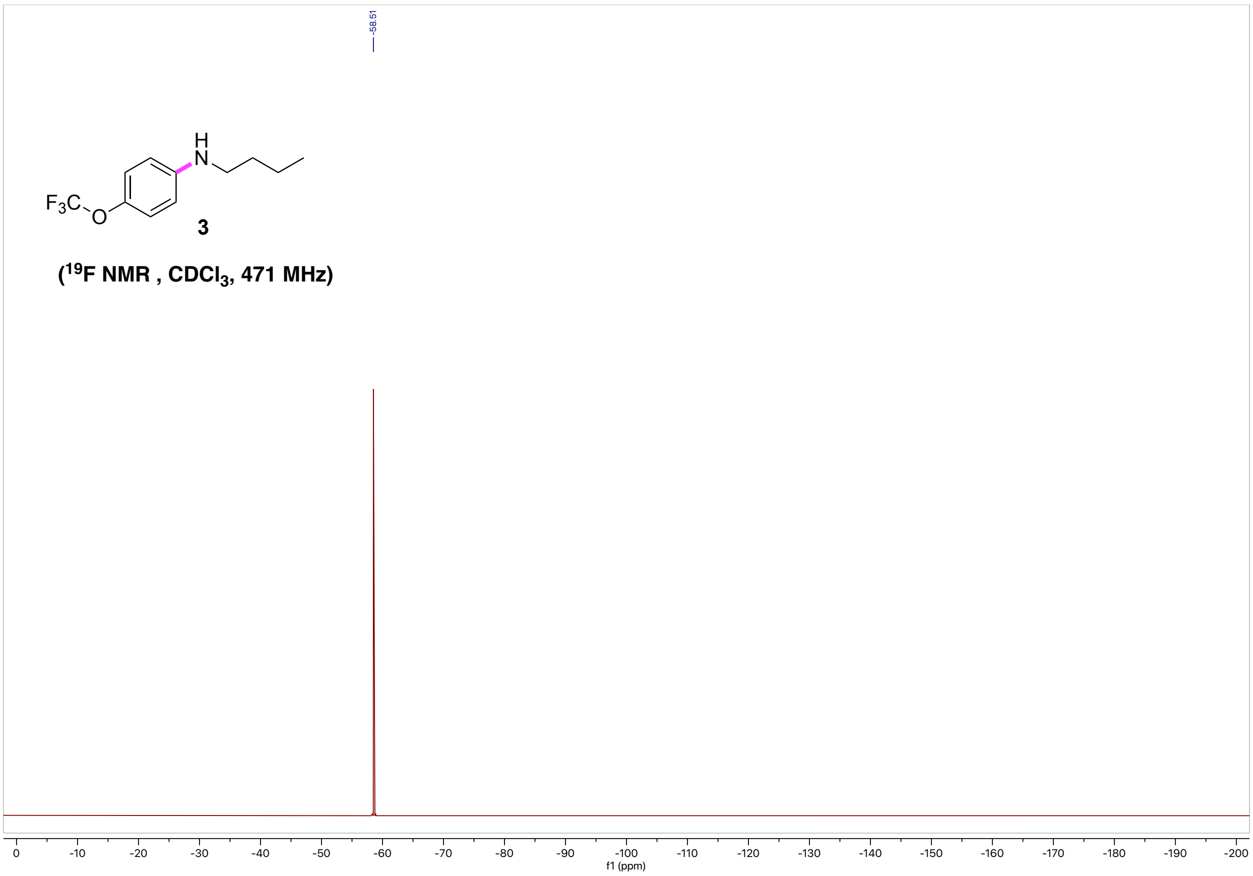
-58.51
F3C
O
H
N
3
(19F NMR , CDCl3, 471 MHz)
0
-10
-20
-30
-40
-50
-60
-70
-80
-90
-100
-110
-120
-130
-140
-150
-160
-170
-180
-190
-200
f1 (ppm)

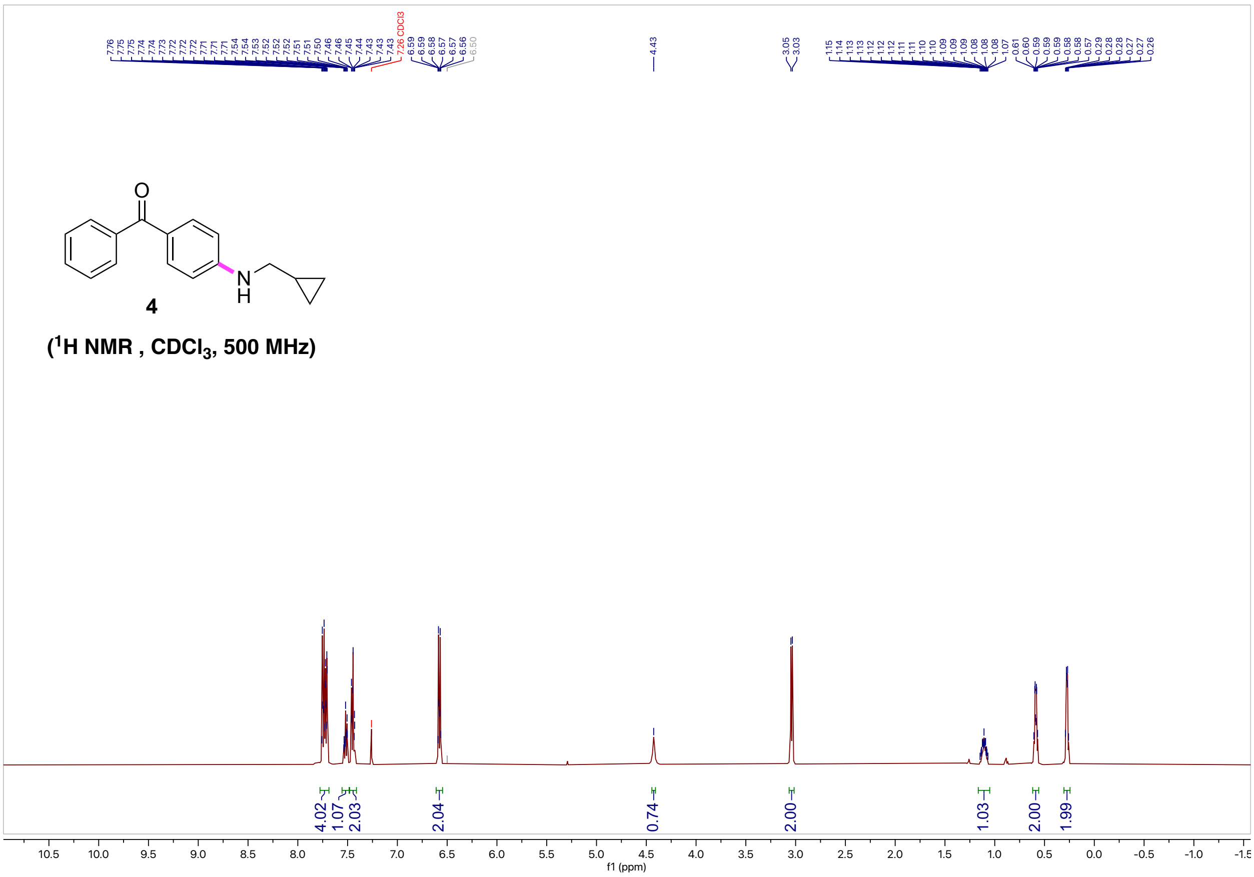

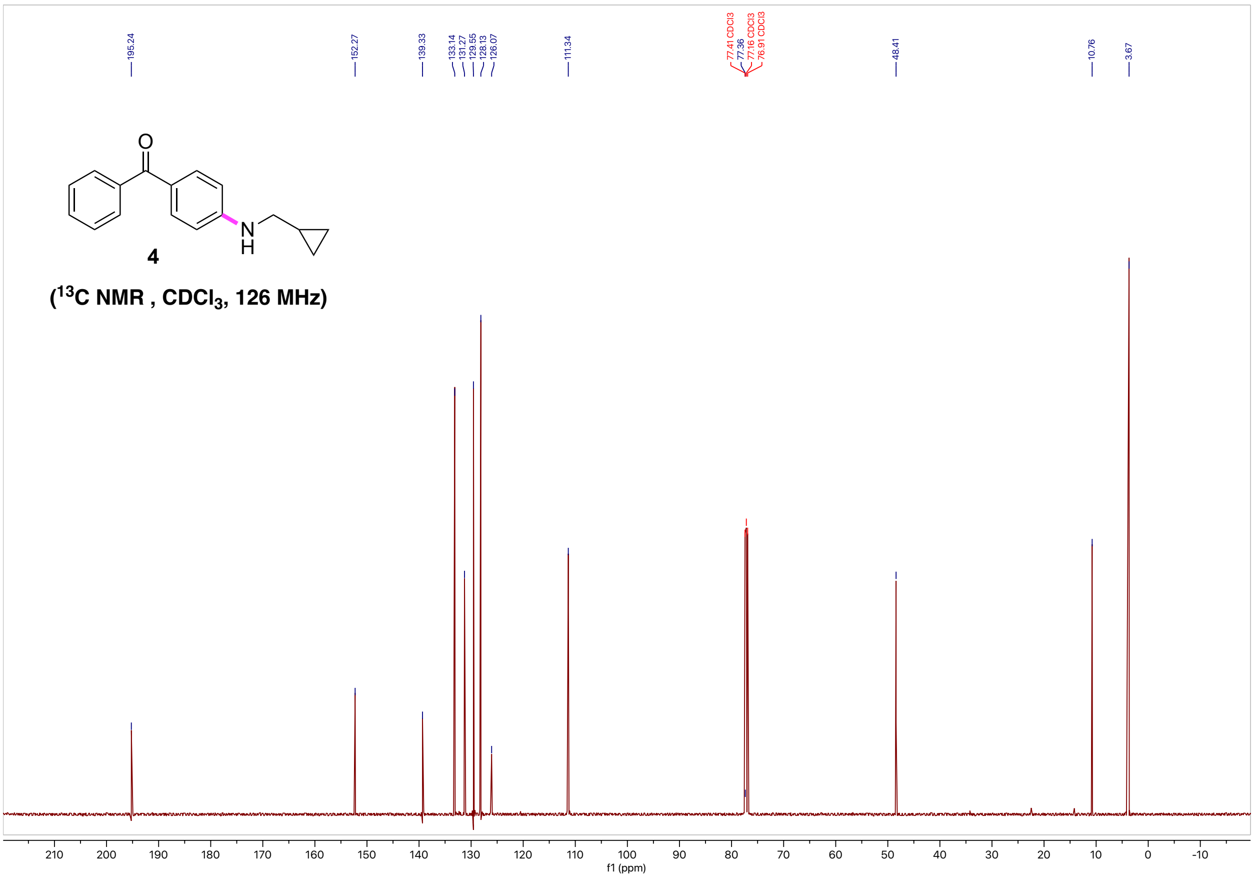

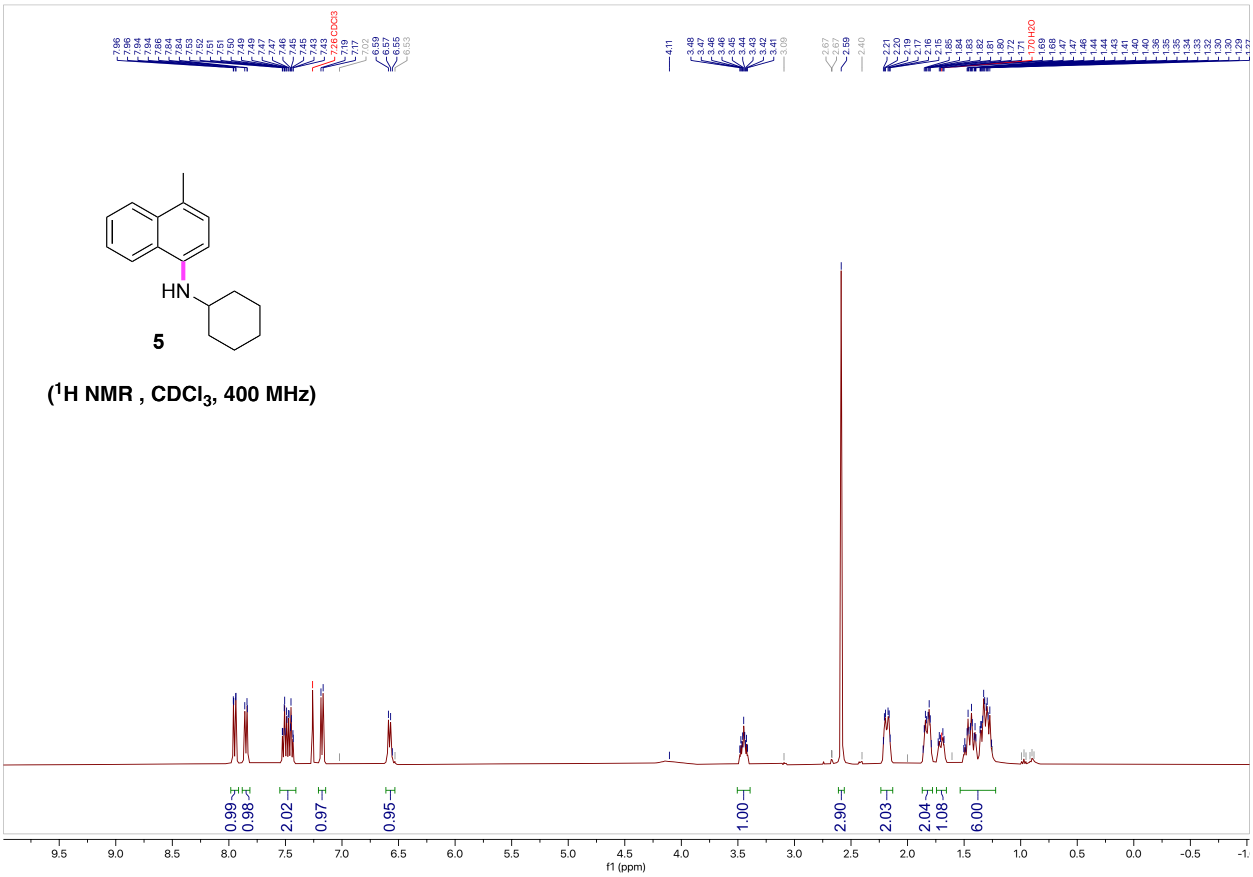

5

(^{1}H NMR , $CDCl_3$, 400 MHz)

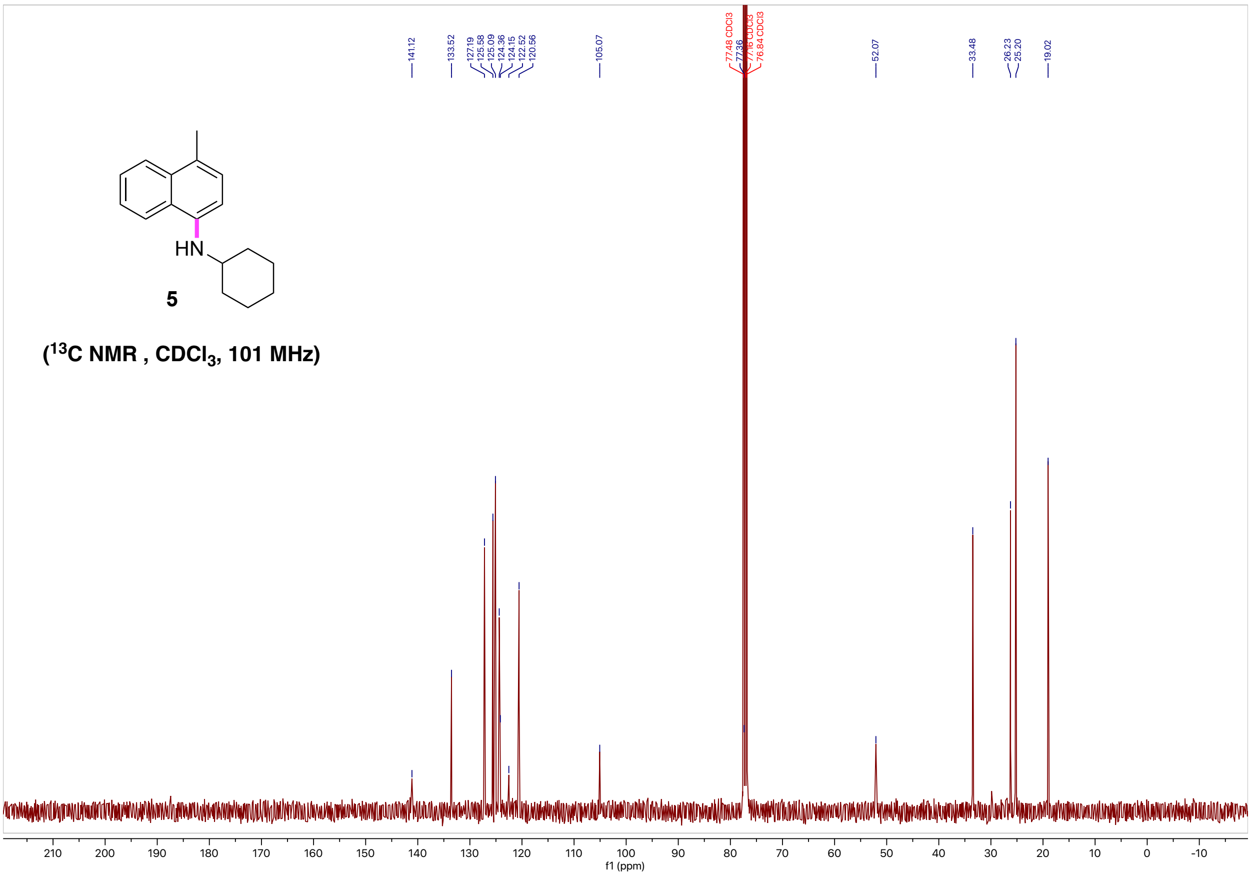

5

(^{13}C NMR , $CDCl_3$, 101 MHz)

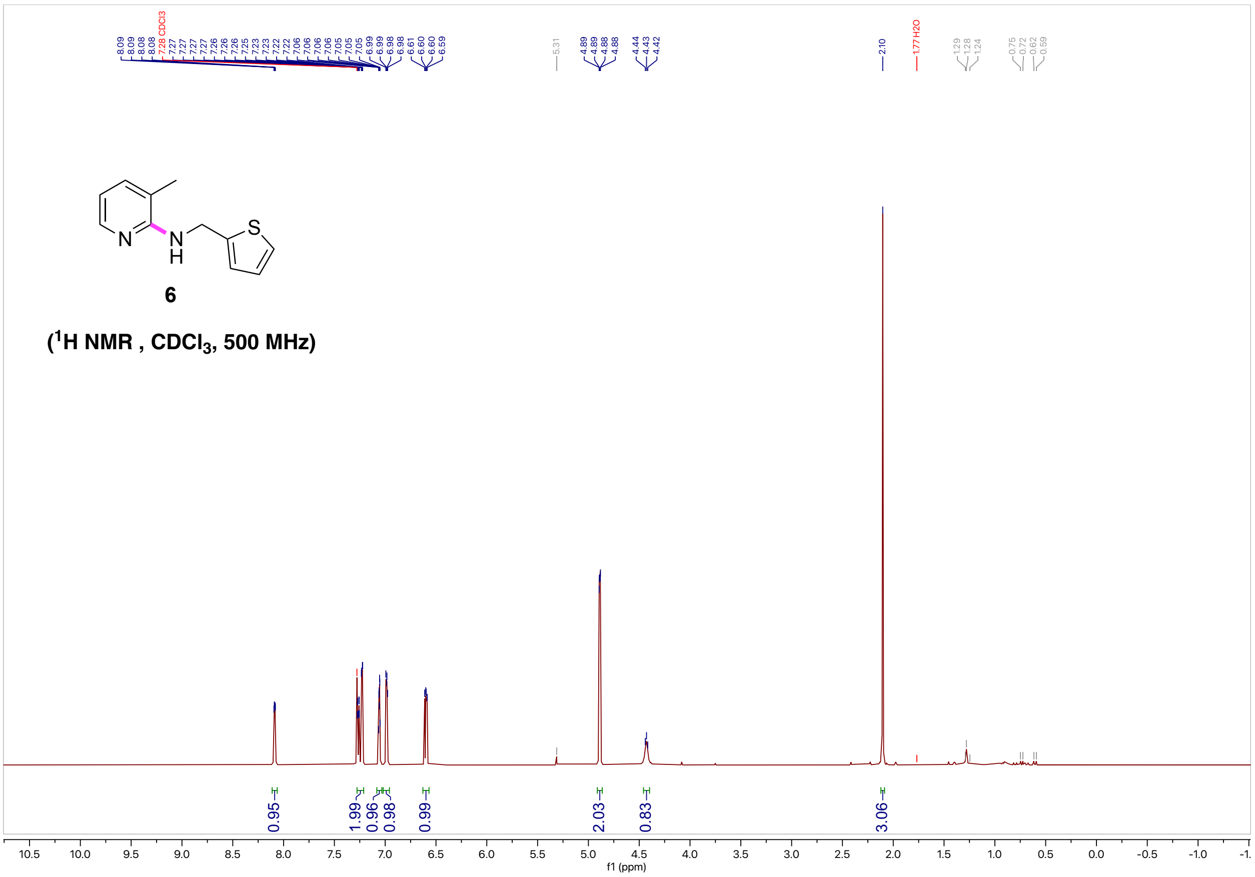
6
(1H NMR , CDCl3, 500 MHz)
0.95
1.99
0.96
0.98
0.99
2.03
0.83
3.06
f1 (ppm)

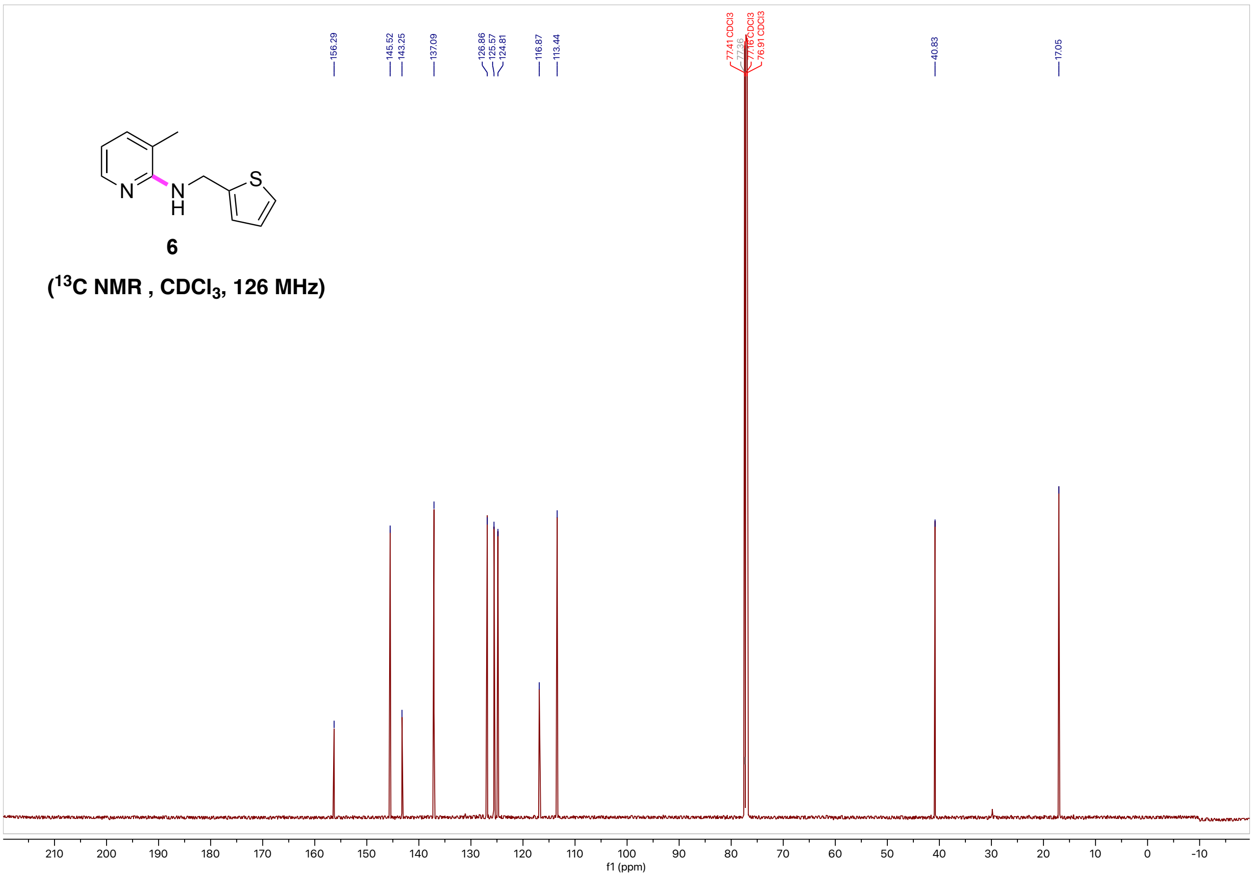
156.29
145.52
143.25
137.09
126.86
125.57
124.81
116.87
113.44
40.83
17.05
6
(13C NMR , CDCl3, 126 MHz)
f1 (ppm)

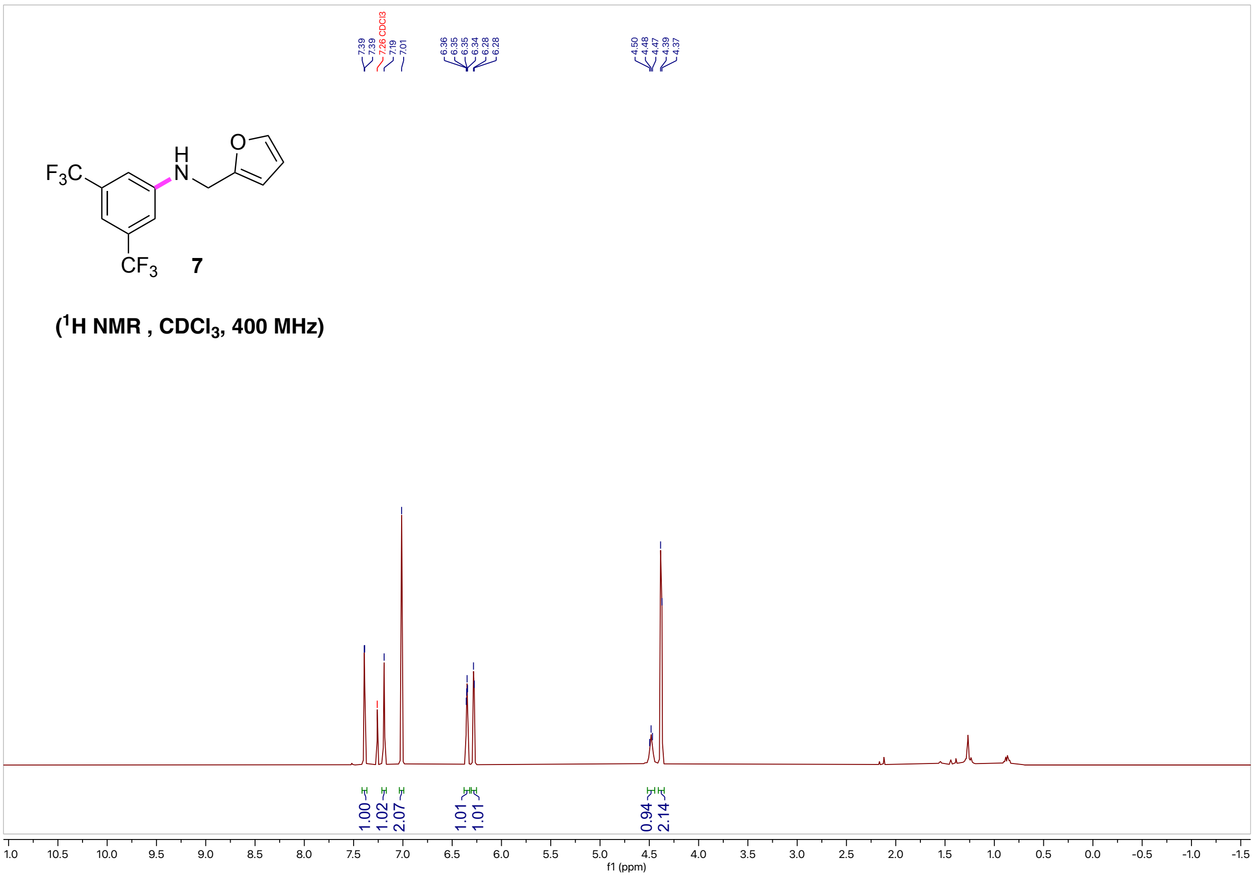
7
(¹H NMR , CDCl3, 400 MHz)
f1 (ppm)

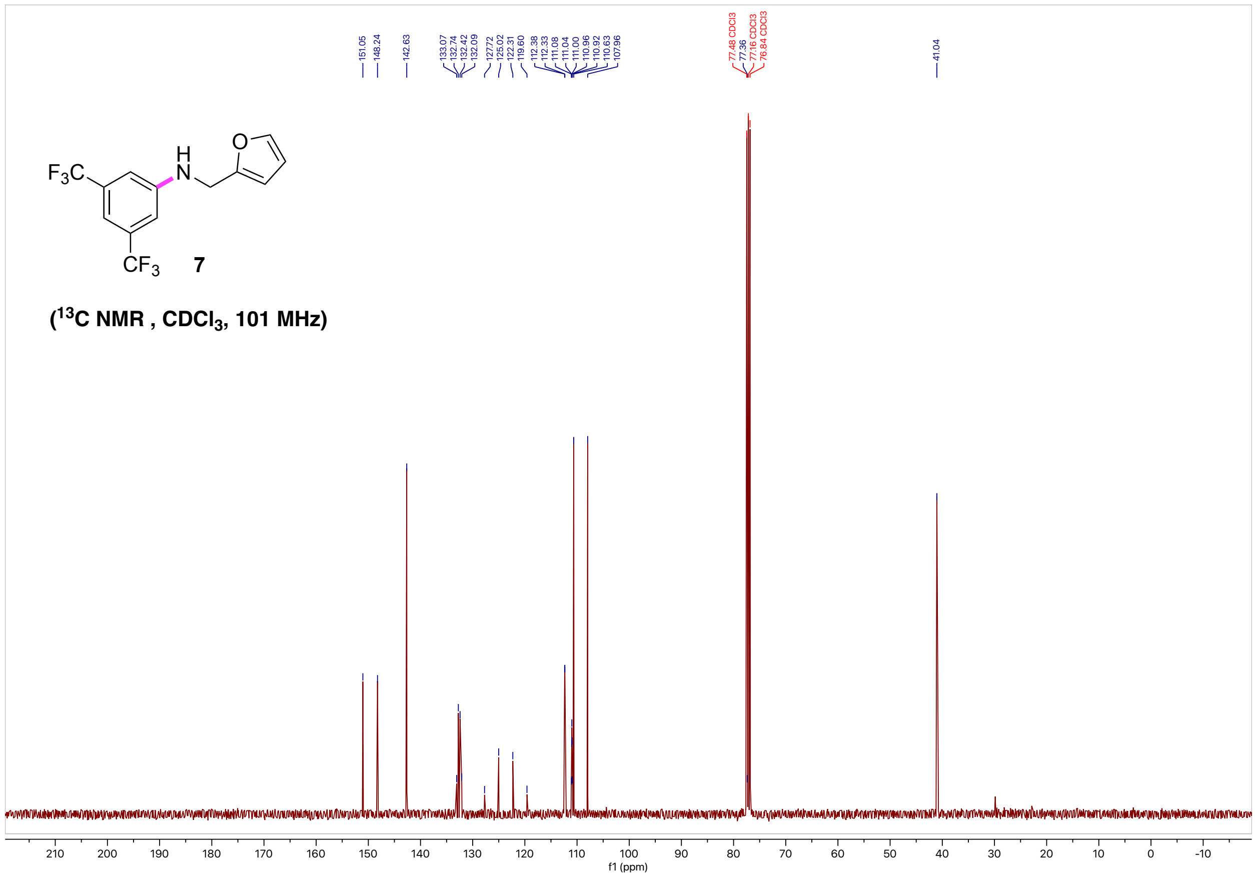
7
(¹³C NMR , CDCl3, 101 MHz)
f1 (ppm)

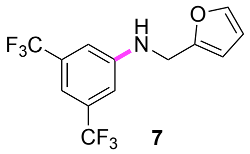

7

(^{19}F NMR , $CDCl_3$, 376 MHz)

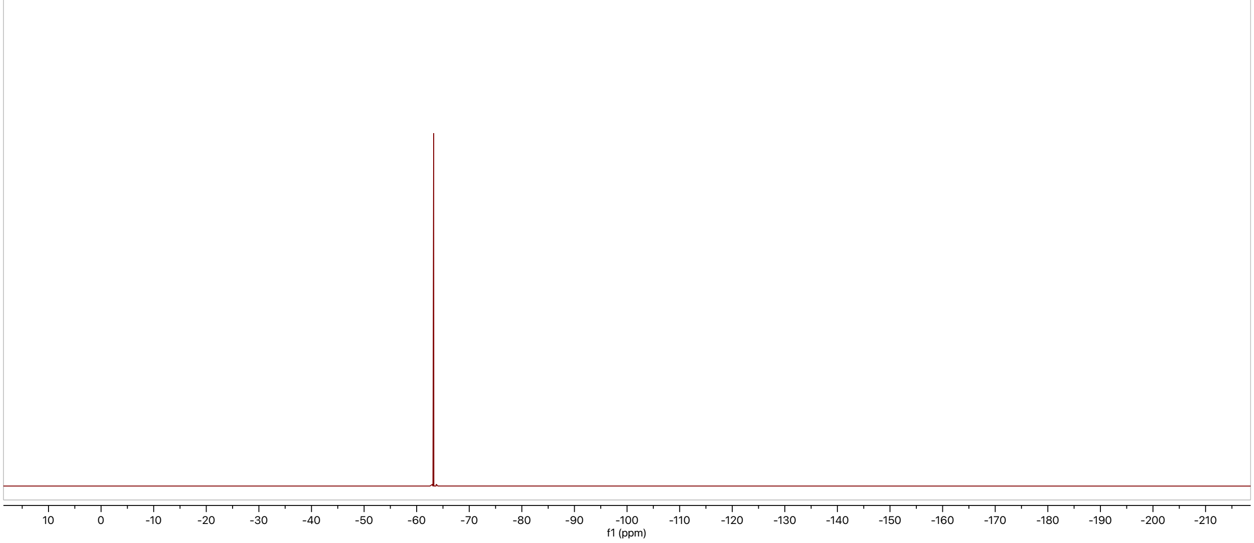

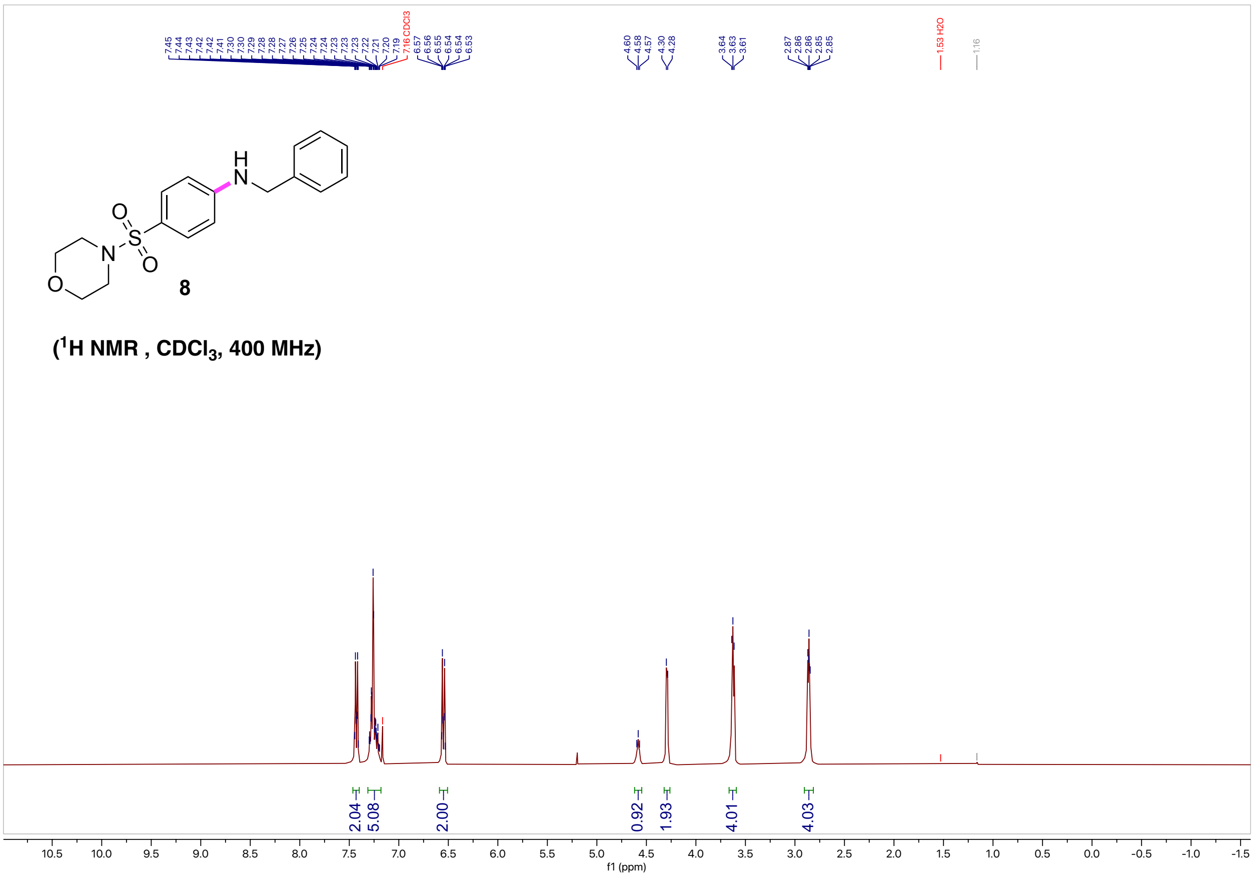
H
N
O
S
O
N
O
8
(1H NMR , CDCl3, 400 MHz)
7.16 CDCl3
1.53 H2O
1.16
2.04
5.08
2.00
0.92
1.93
4.01
4.03
f1 (ppm)

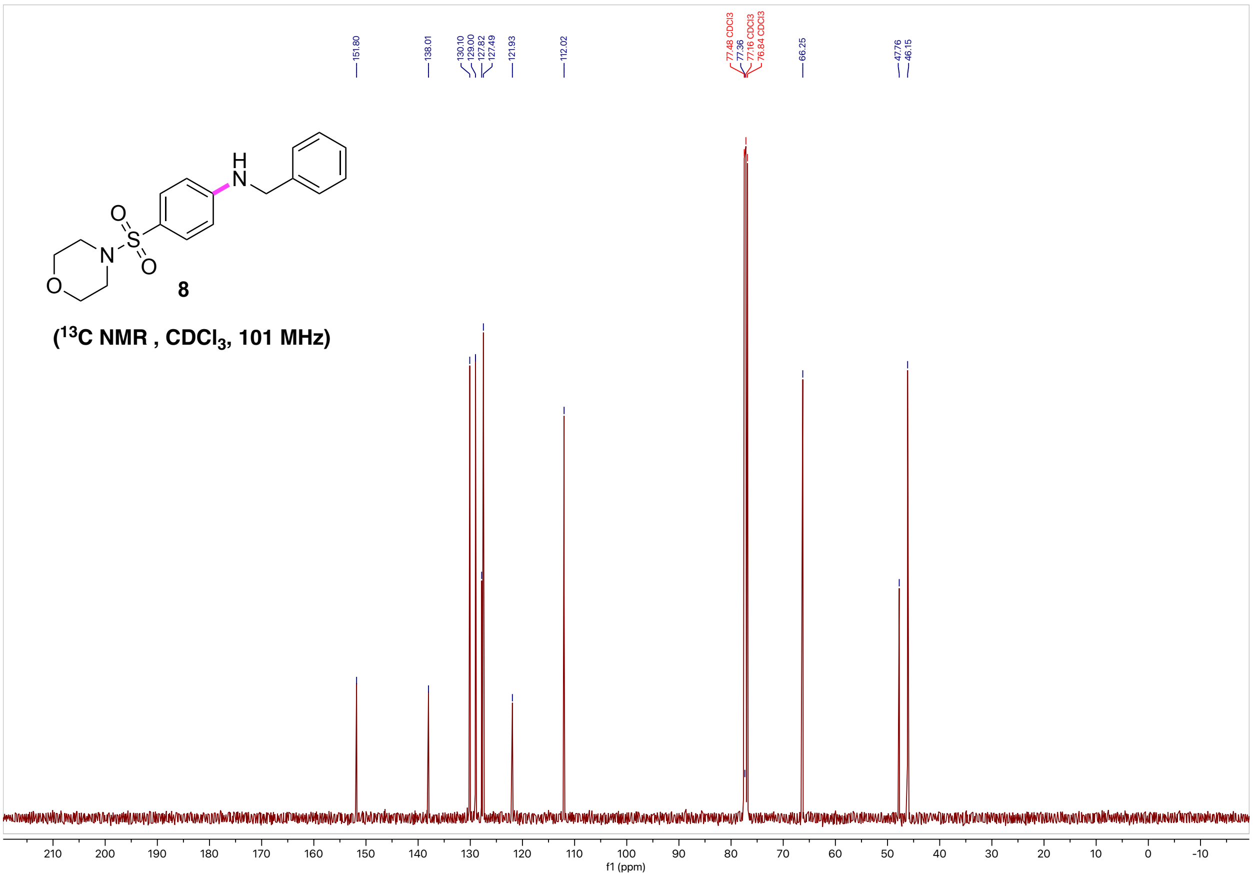
H
N
O
S
O
N
O
8
(13C NMR , CDCl3, 101 MHz)
151.80
138.01
130.10
129.00
127.82
127.49
121.93
112.02
77.48 CDCl3
77.36
77.16 CDCl3
76.84 CDCl3
66.25
47.76
46.15
f1 (ppm)

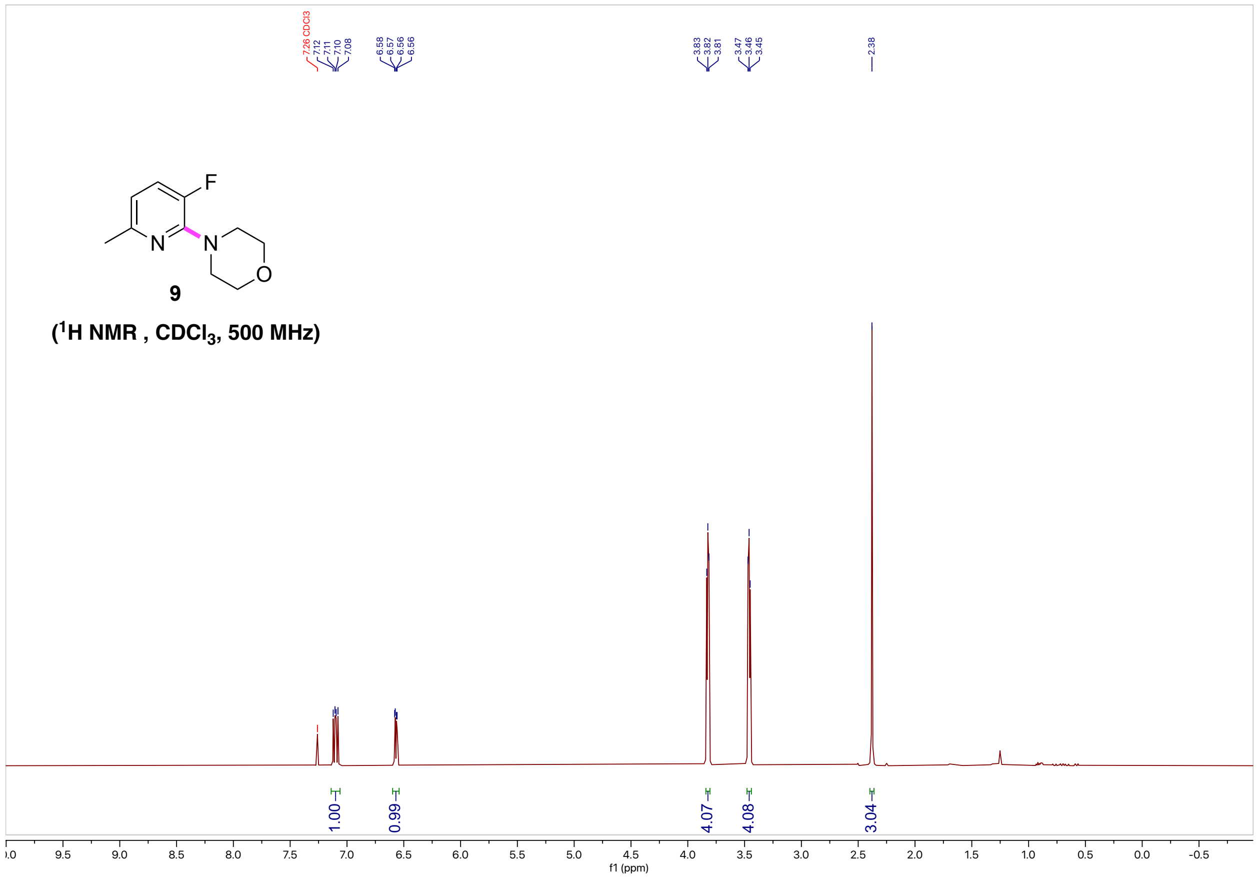
9
(1H NMR , CDCl3, 500 MHz)
7.26 CDCl3
7.12
7.11
7.10
7.08
6.58
6.57
6.56
6.56
3.83
3.82
3.81
3.47
3.46
3.45
2.38
1.00
0.99
4.07
4.08
3.04
f1 (ppm)

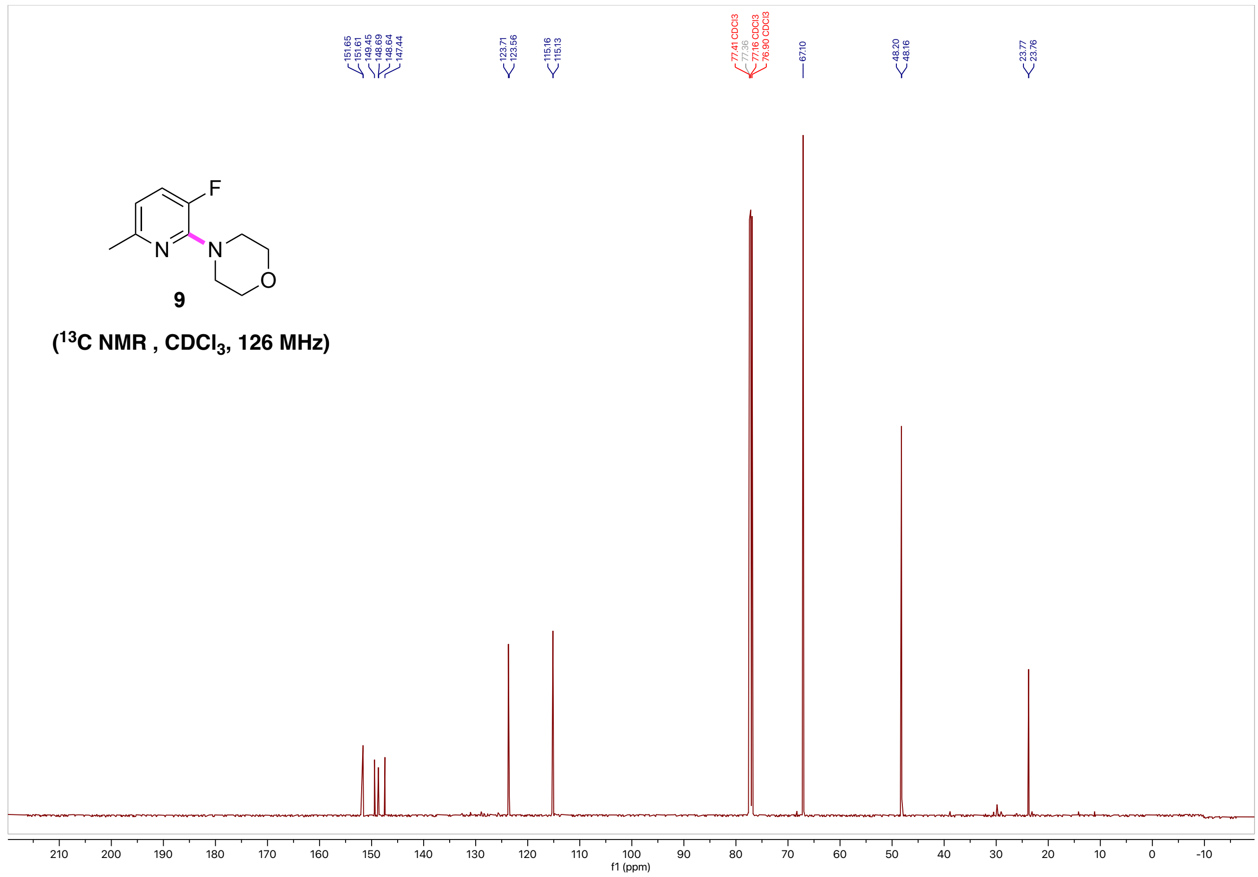
9
(13C NMR , CDCl3, 126 MHz)
151.65
151.61
149.45
148.69
148.64
147.44
123.71
123.56
115.16
115.13
77.41 CDCl3
77.16 CDCl3
76.90 CDCl3
67.10
48.20
48.16
23.77
23.76
f1 (ppm)

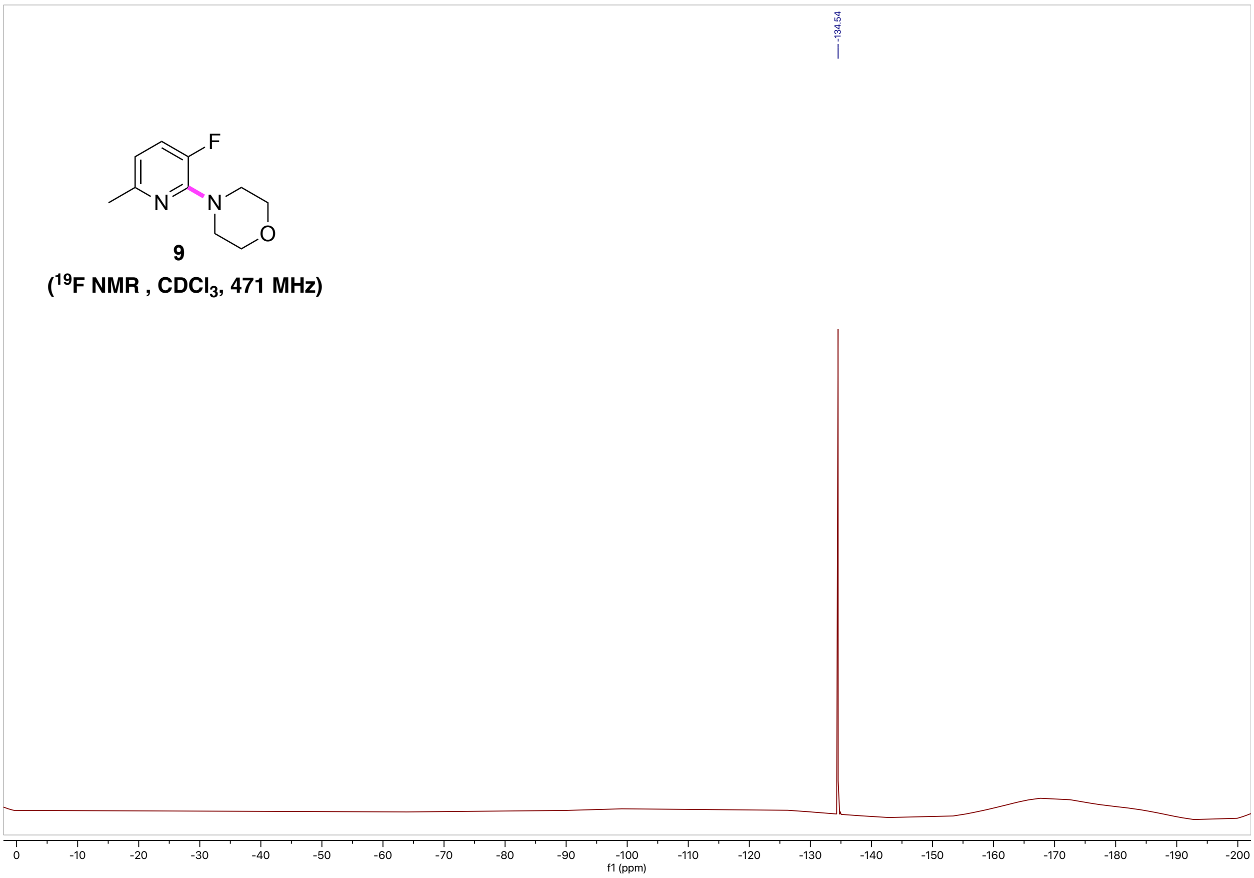

-134.54
F
N
N
O
9
(^{19}F NMR , $CDCl_3$, 471 MHz)
0 -10 -20 -30 -40 -50 -60 -70 -80 -90 -100 -110 -120 -130 -140 -150 -160 -170 -180 -190 -200
f1 (ppm)

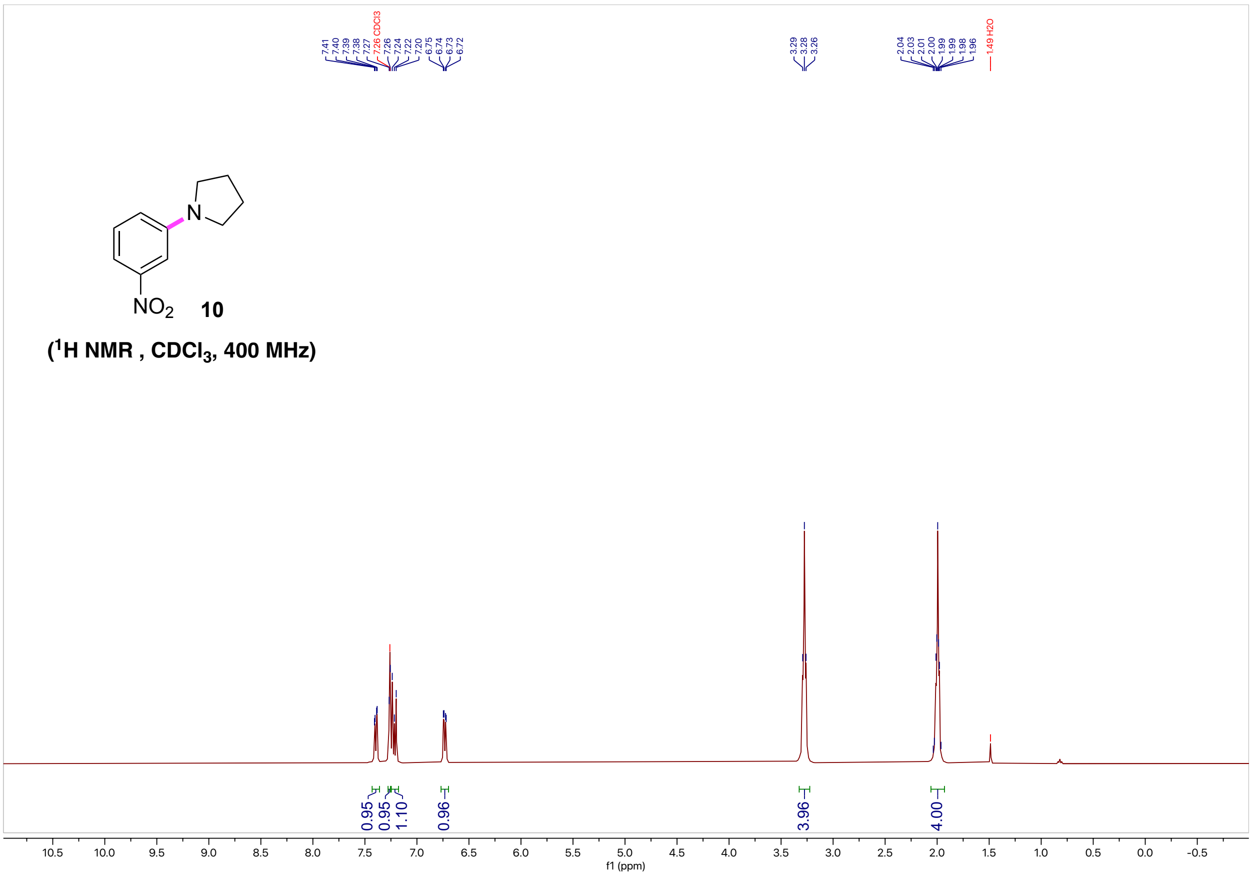
N
NO2
10
(1H NMR , CDCl3, 400 MHz)
f1 (ppm)

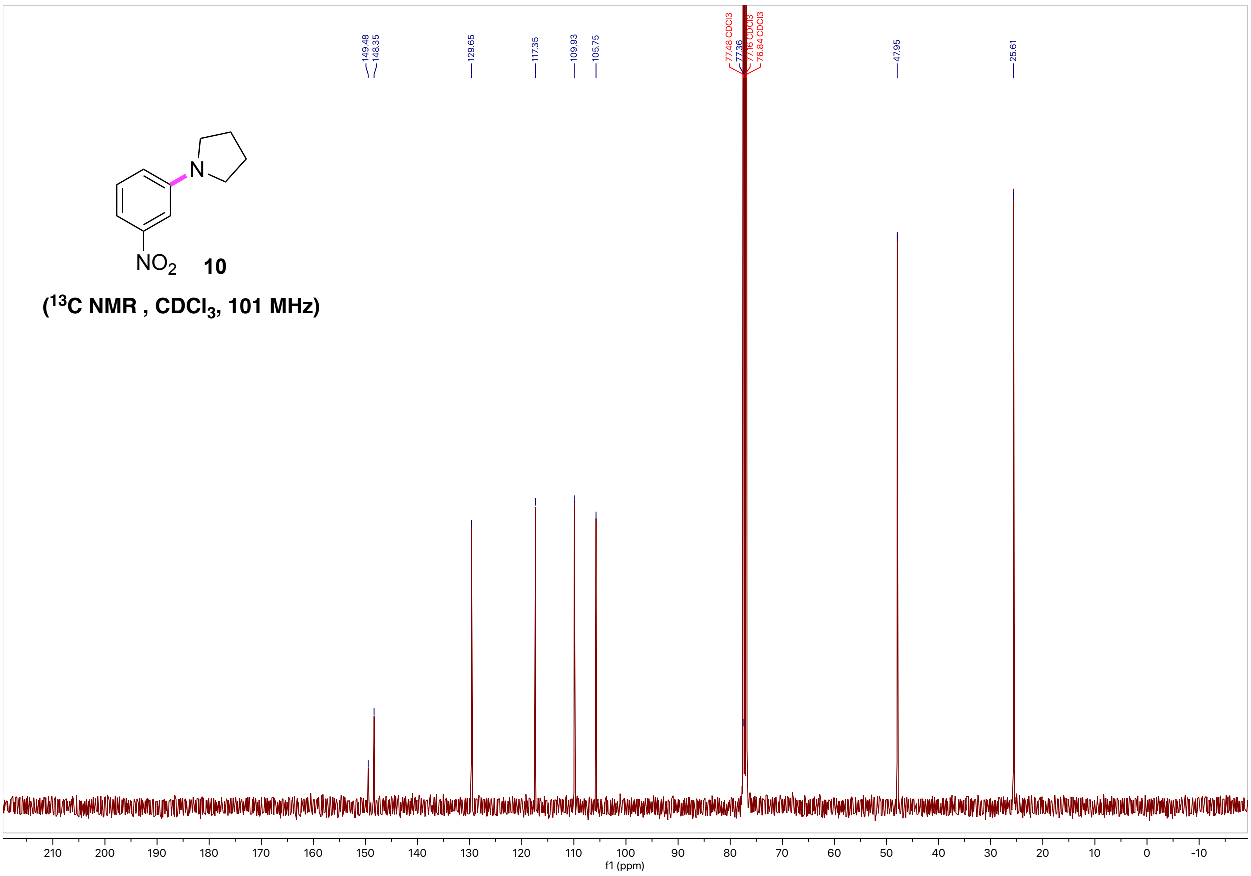
N
NO2
10
(13C NMR , CDCl3, 101 MHz)
149.48
148.35
129.65
117.35
109.93
105.75
77.48 CDCl3
76.84 CDCl3
47.95
25.61
f1 (ppm)

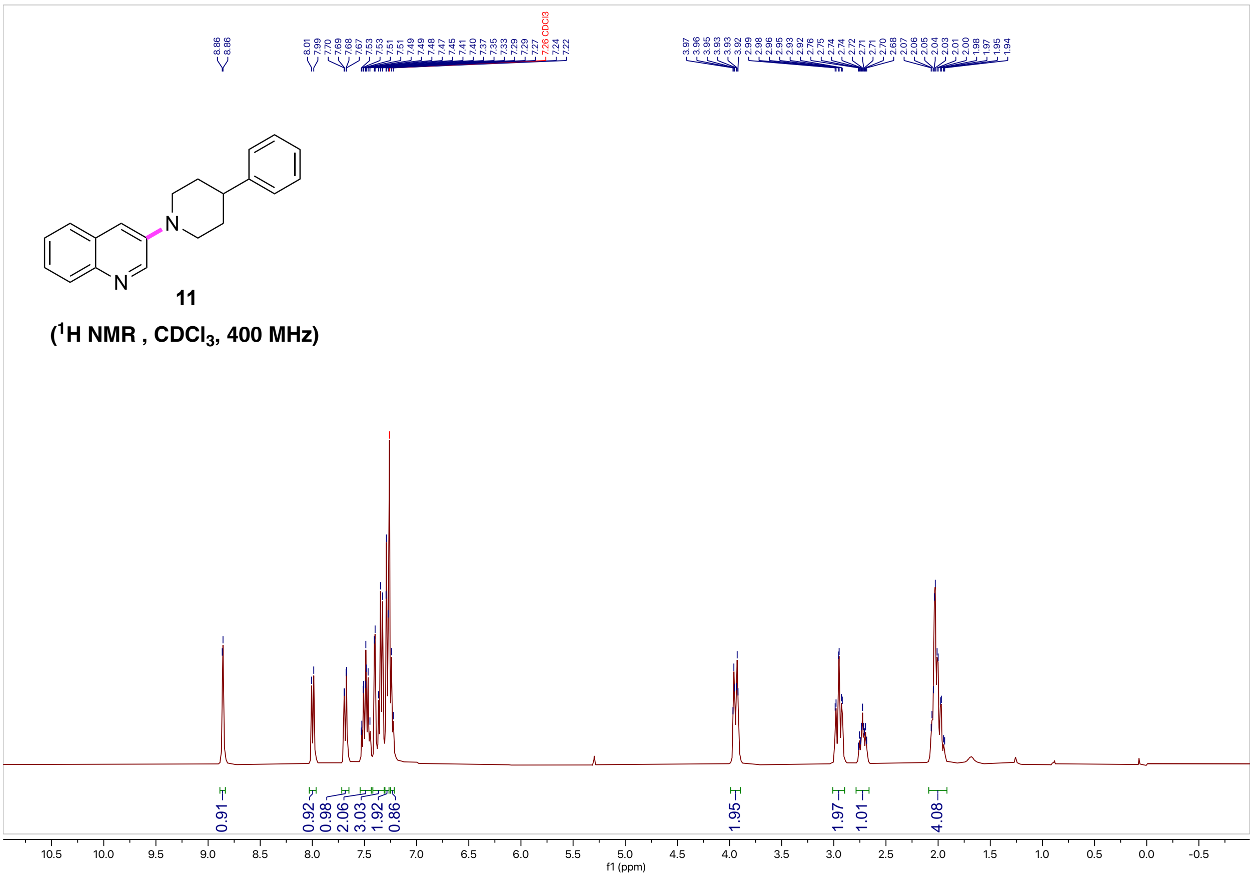
8.86
8.86
8.01
7.99
7.70
7.69
7.68
7.67
7.53
7.53
7.51
7.51
7.49
7.48
7.47
7.45
7.41
7.40
7.37
7.35
7.33
7.29
7.29
7.27
7.26 CDCl3
7.24
7.22
3.97
3.96
3.95
3.93
3.93
3.92
2.99
2.98
2.96
2.95
2.93
2.92
2.76
2.75
2.74
2.74
2.72
2.71
2.71
2.70
2.68
2.07
2.06
2.05
2.04
2.03
2.01
2.00
1.98
1.97
1.95
1.94
N
N
11
(1H NMR , CDCl3, 400 MHz)
0.91
0.92
0.98
2.06
3.03
1.92
0.86
1.95
1.97
1.01
4.08
10.5
10.0
9.5
9.0
8.5
8.0
7.5
7.0
6.5
6.0
5.5
5.0
4.5
4.0
3.5
3.0
2.5
2.0
1.5
1.0
0.5
0.0
-0.5
f1 (ppm)

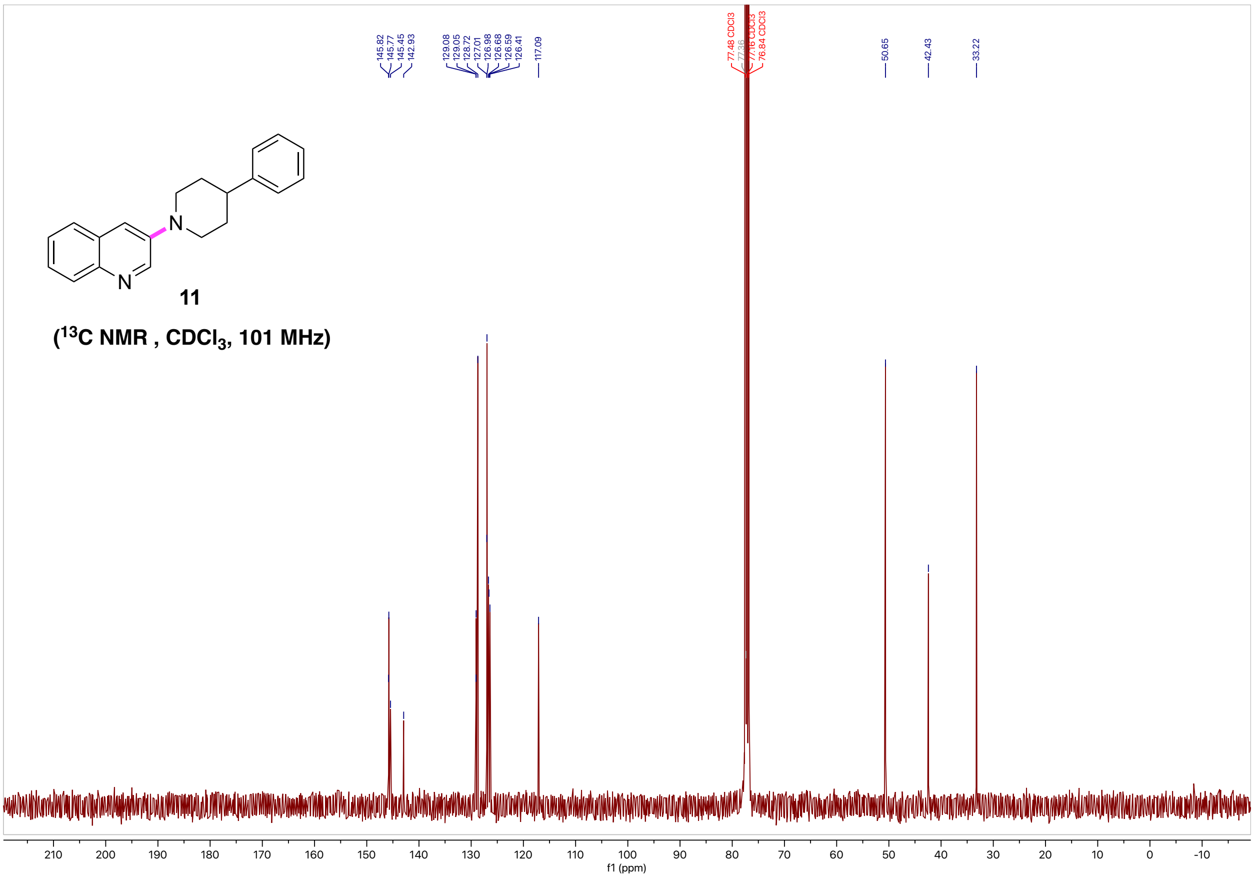
145.82
145.77
145.45
142.93
129.08
129.05
128.72
127.01
126.98
126.68
126.59
126.41
117.09
77.48 CDCl3
77.36
77.16 CDCl3
76.84 CDCl3
50.65
42.43
33.22
N
N
11
(13C NMR , CDCl3, 101 MHz)
210
200
190
180
170
160
150
140
130
120
110
100
90
80
70
60
50
40
30
20
10
0
-10
f1 (ppm)

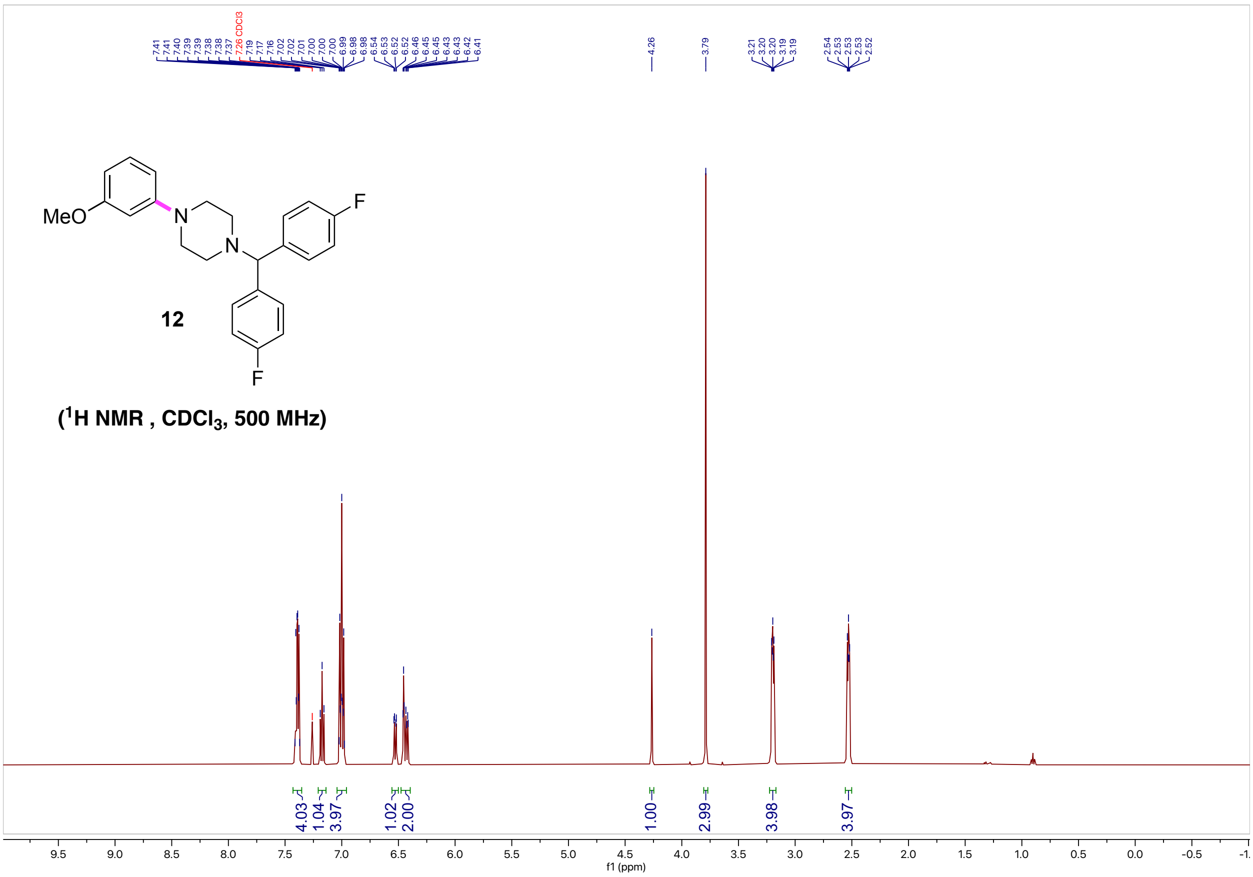
MeO
N
N
F
12
F
(¹H NMR , CDCl3, 500 MHz)
4.03
1.04
3.97
1.02
2.00
1.00
2.99
3.98
3.97
f1 (ppm)

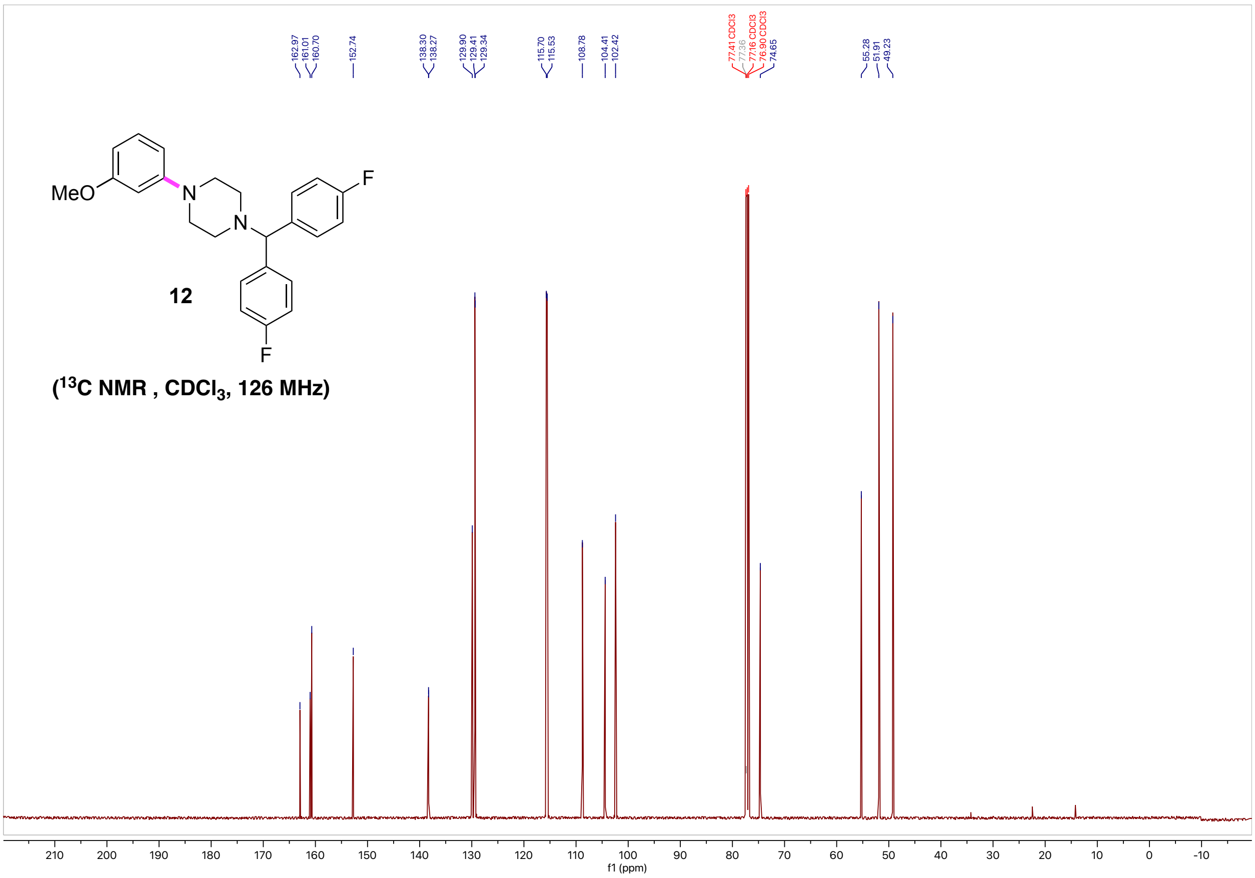
MeO
N
N
F
12
F
(¹³C NMR , CDCl3, 126 MHz)
162.97
161.01
160.70
152.74
138.30
138.27
129.90
129.41
129.34
115.70
115.53
108.78
104.41
102.42
77.41 CDCl3
77.16 CDCl3
76.90 CDCl3
74.65
55.28
51.91
49.23
f1 (ppm)

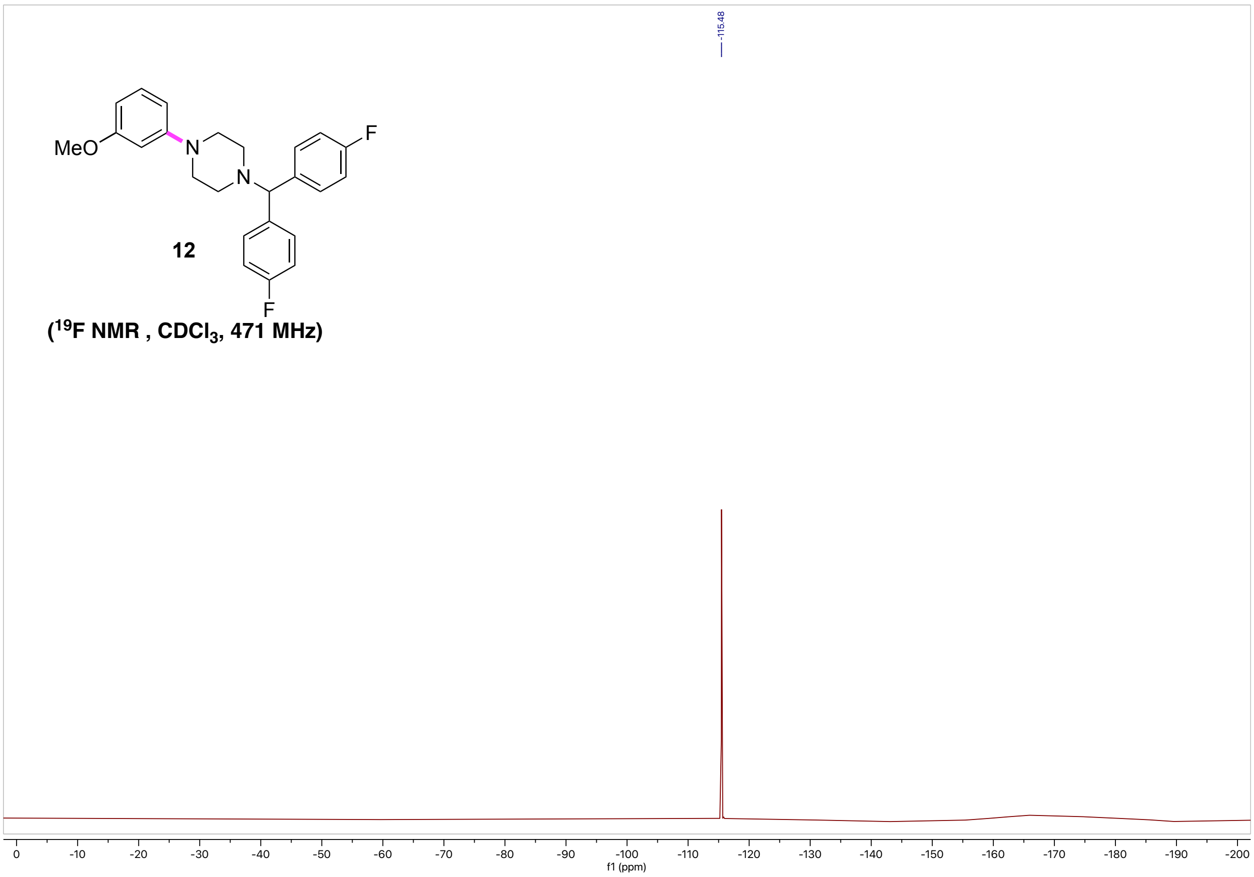

12

(^{19}F NMR , $CDCl_3$, 471 MHz)

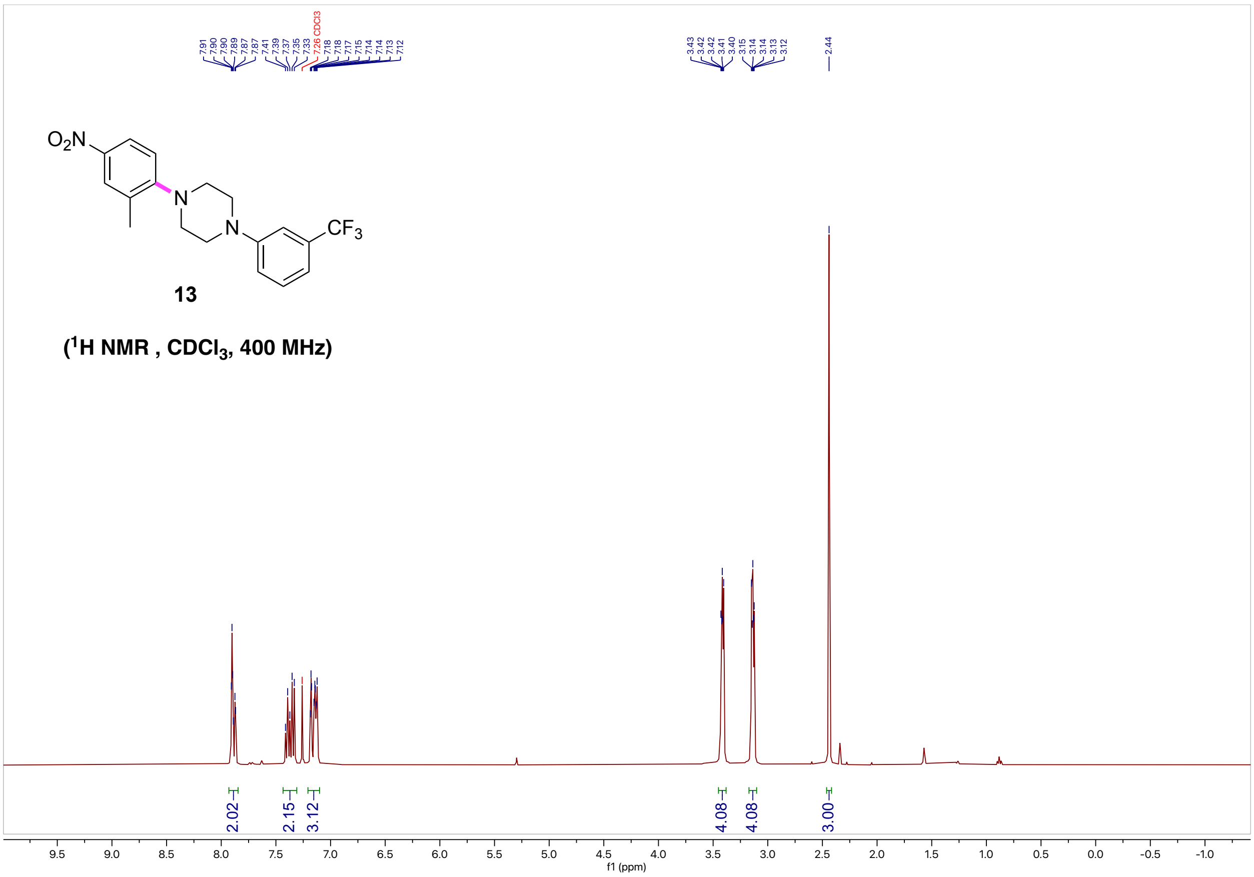
7.91
7.90
7.90
7.89
7.87
7.87
7.41
7.39
7.37
7.35
7.33
7.26 CDCl3
7.18
7.18
7.17
7.15
7.14
7.14
7.13
7.12
3.43
3.42
3.42
3.41
3.40
3.15
3.14
3.14
3.13
3.12
2.44
O_2N
N
N
CF_3
13
(^{1}H NMR , $CDCl_3$, 400 MHz)
2.02
2.15
3.12
4.08
4.08
3.00
9.5 9.0 8.5 8.0 7.5 7.0 6.5 6.0 5.5 5.0 4.5 4.0 3.5 3.0 2.5 2.0 1.5 1.0 0.5 0.0 -0.5 -1.0
f1 (ppm)

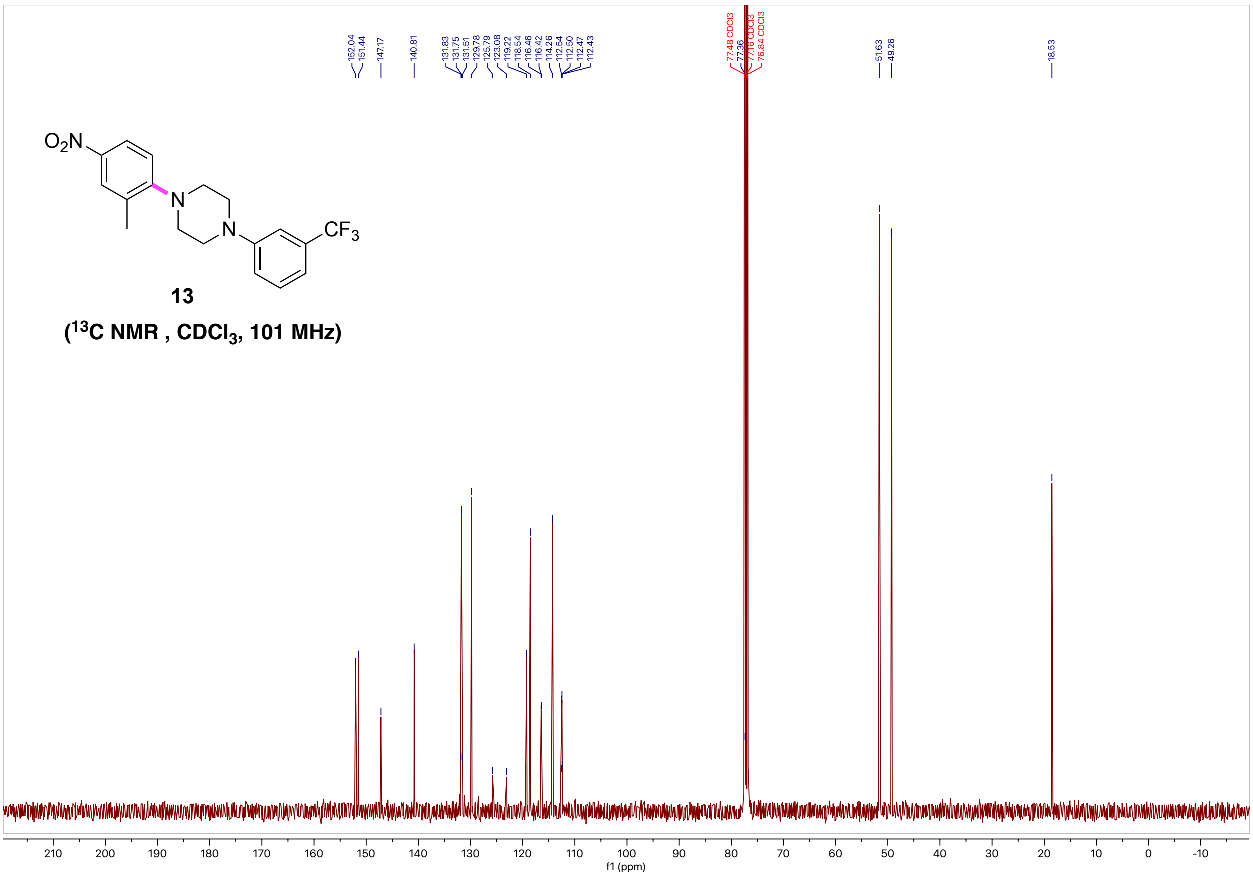
152.04
151.44
147.17
140.81
131.83
131.75
131.51
129.78
125.79
123.08
119.22
118.54
116.46
116.42
114.26
112.54
112.50
112.47
112.43
77.48 CDCl3
77.36
77.16 CDCl3
76.84 CDCl3
51.63
49.26
18.53
O_2N
N
N
CF_3
13
(^{13}C NMR , $CDCl_3$, 101 MHz)
210 200 190 180 170 160 150 140 130 120 110 100 90 80 70 60 50 40 30 20 10 0 -10
f1 (ppm)

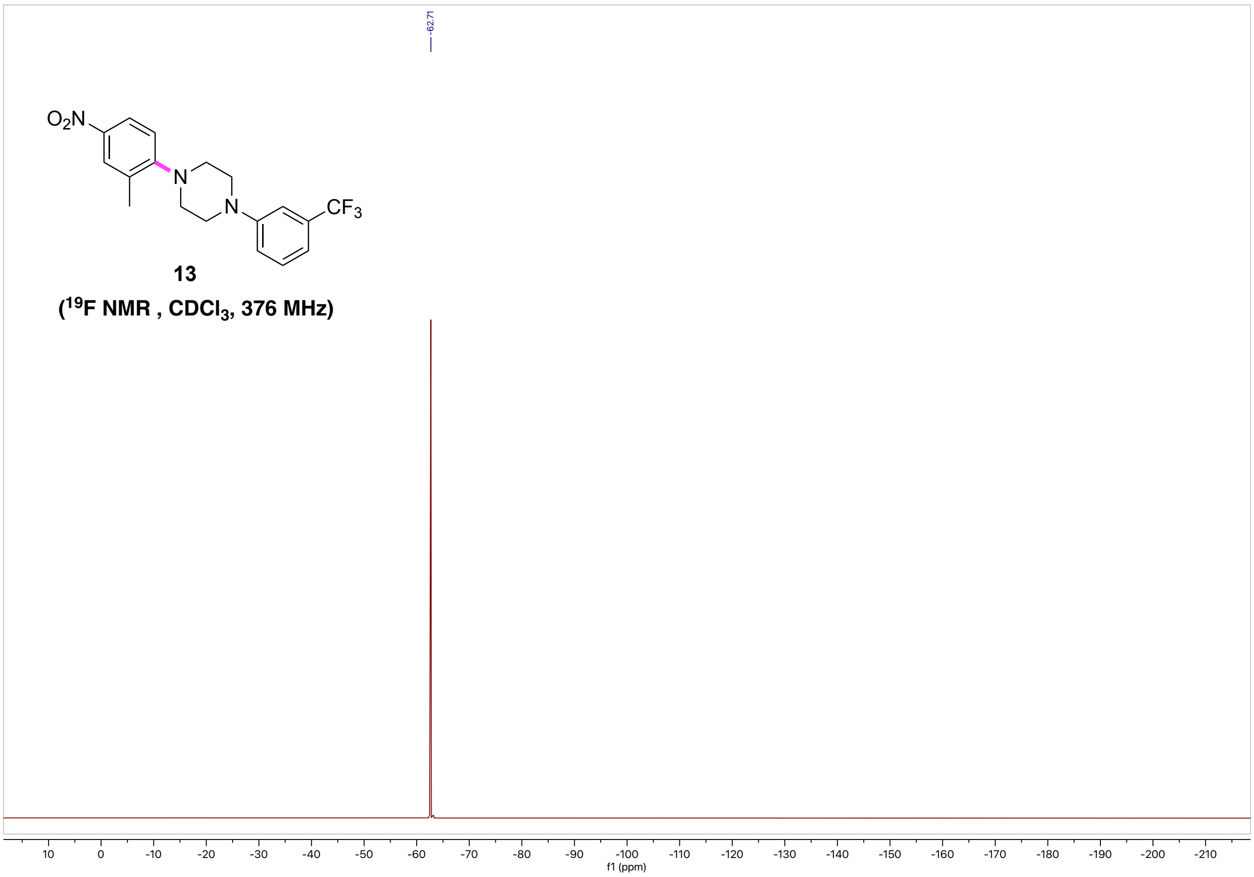
-62.71
O_2N
N
N
CF_3
13
(^{19}F NMR , $CDCl_3$, 376 MHz)
10
0
-10
-20
-30
-40
-50
-60
-70
-80
-90
-100
-110
-120
-130
-140
-150
-160
-170
-180
-190
-200
-210
f1 (ppm)

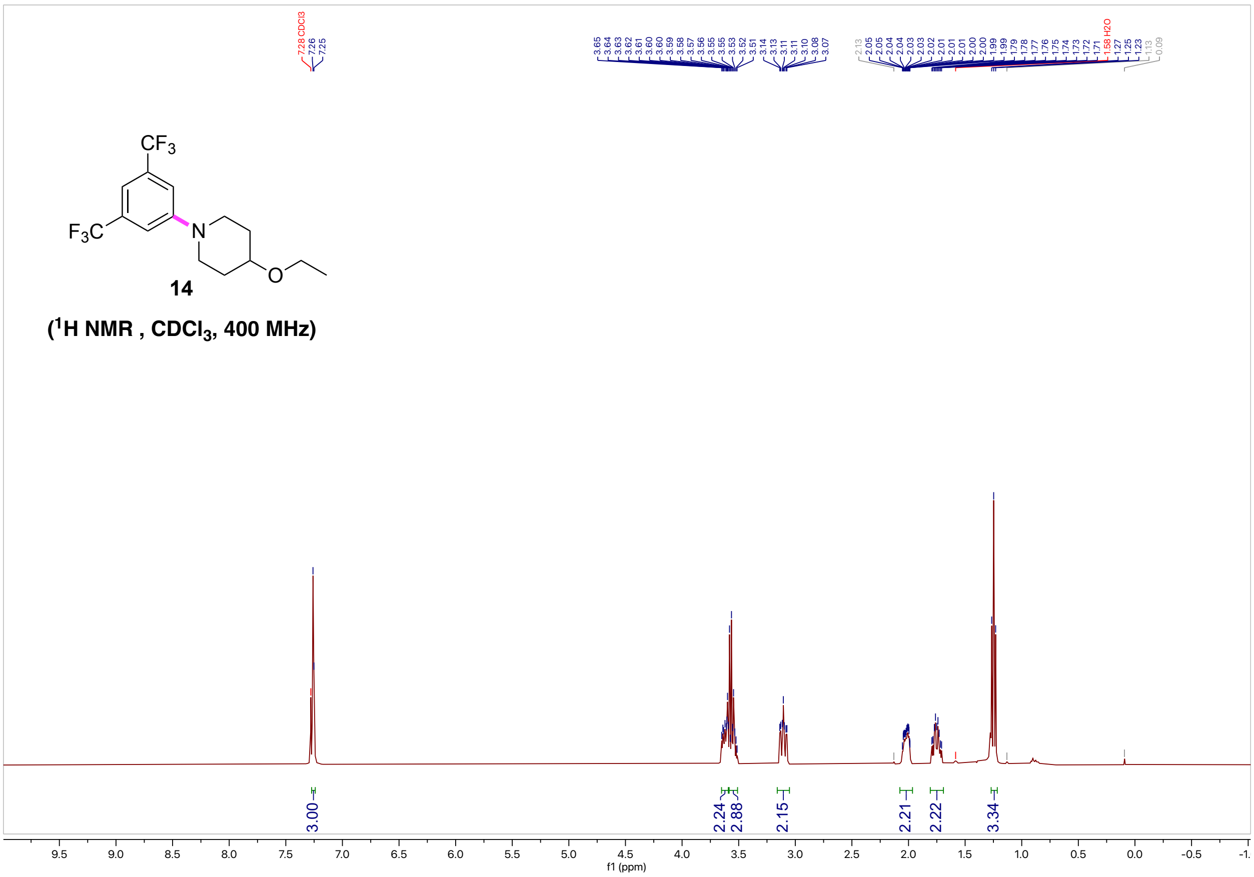
CF3
F3C
N
O
14
(1H NMR , CDCl3, 400 MHz)
f1 (ppm)

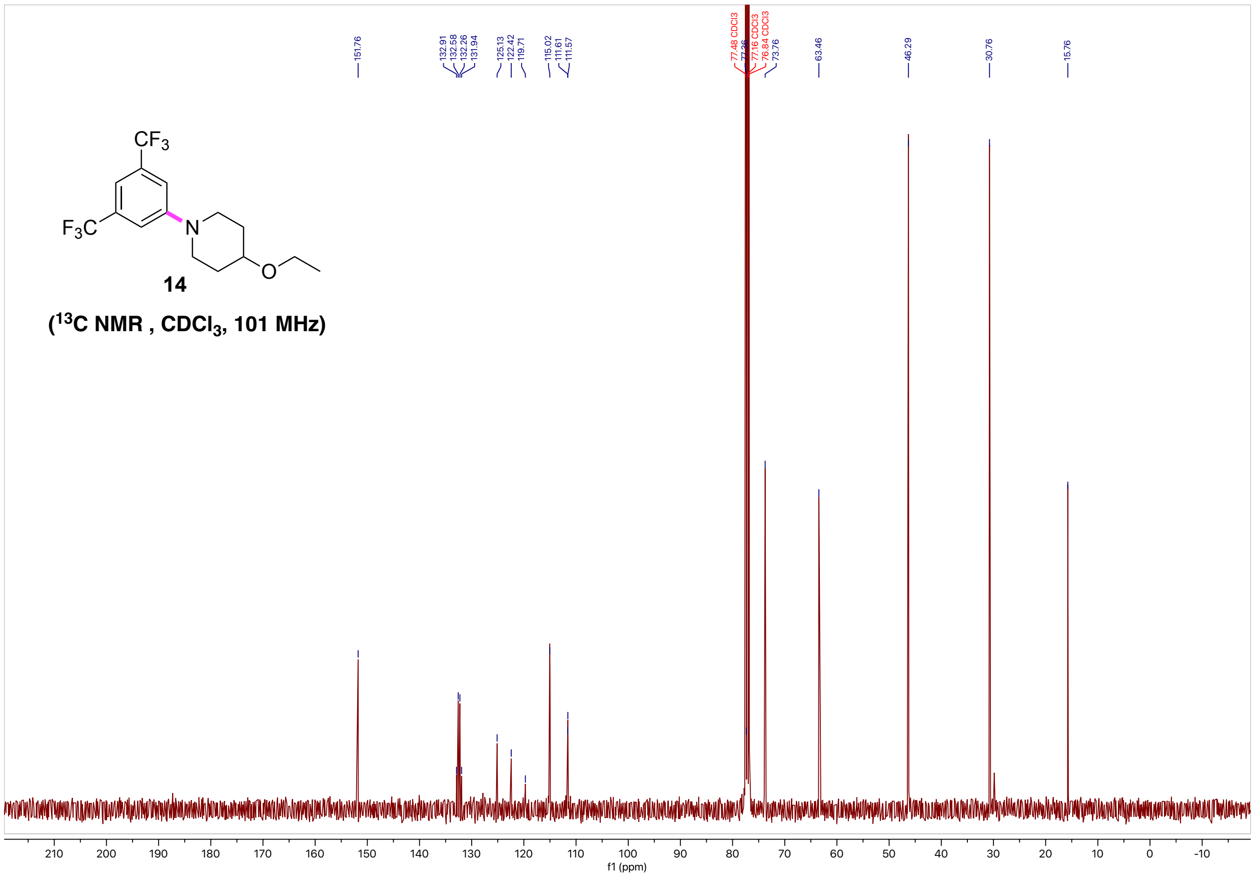
CF3
F3C
N
O
14
(13C NMR , CDCl3, 101 MHz)
f1 (ppm)

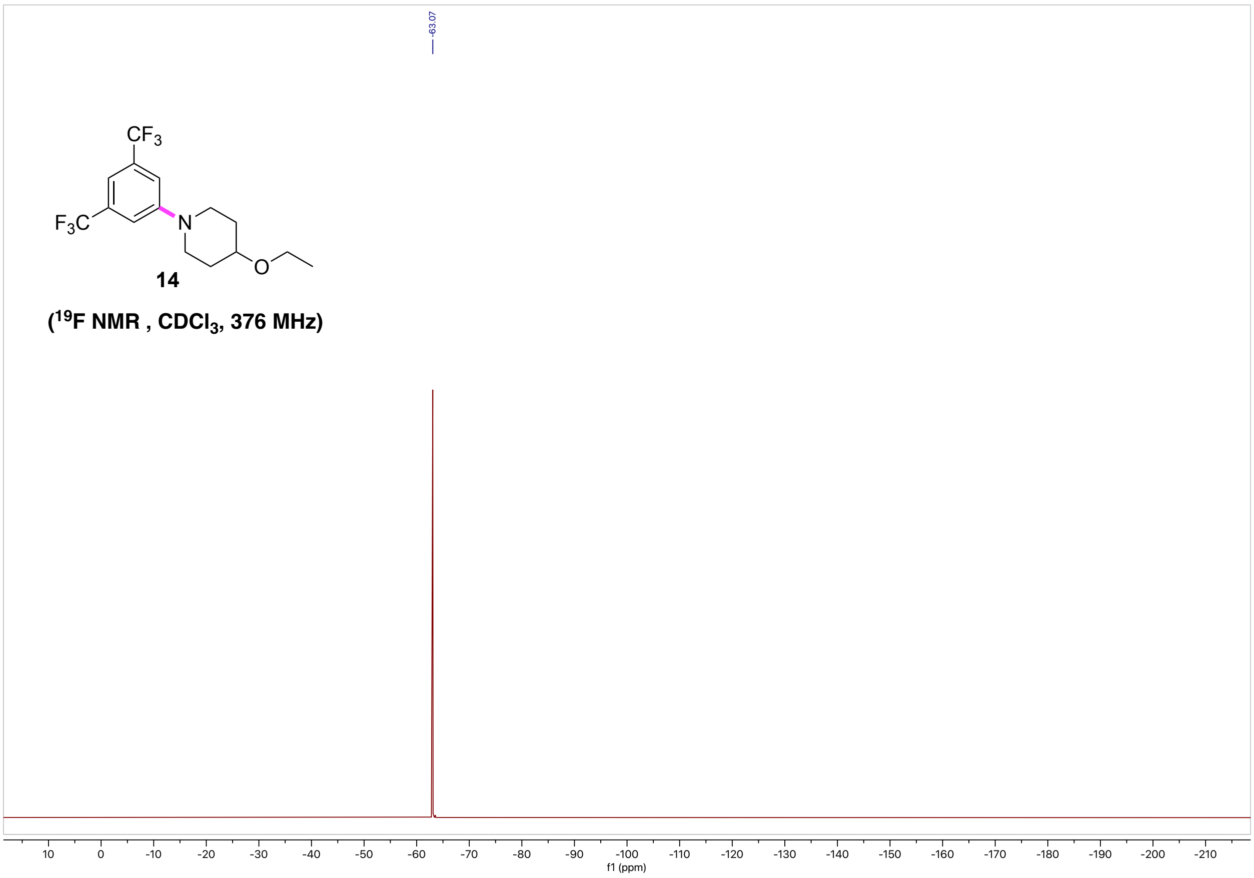

14

(^{19}F NMR , $CDCl_3$, 376 MHz)

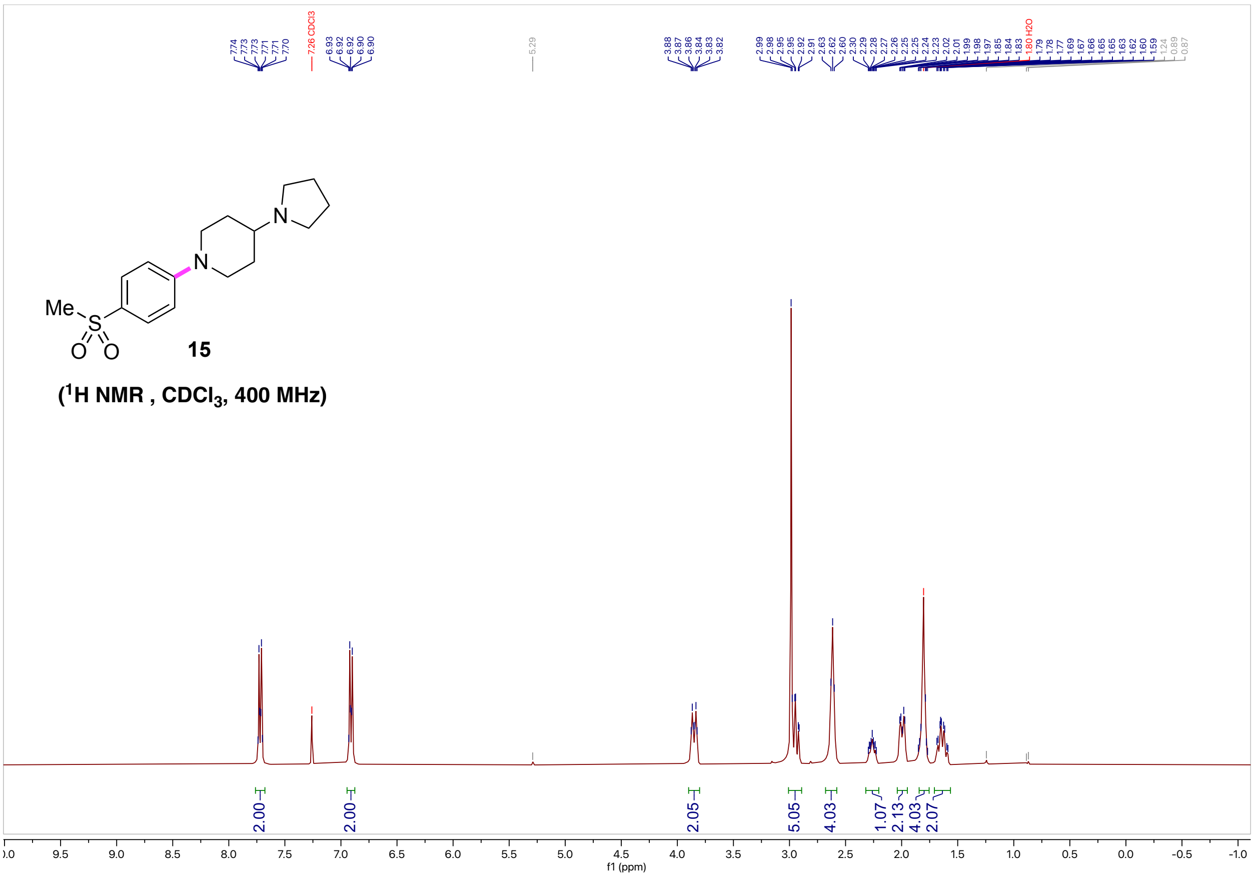
Me
S
O O
N
N
15
(¹H NMR , CDCl₃, 400 MHz)
f1 (ppm)

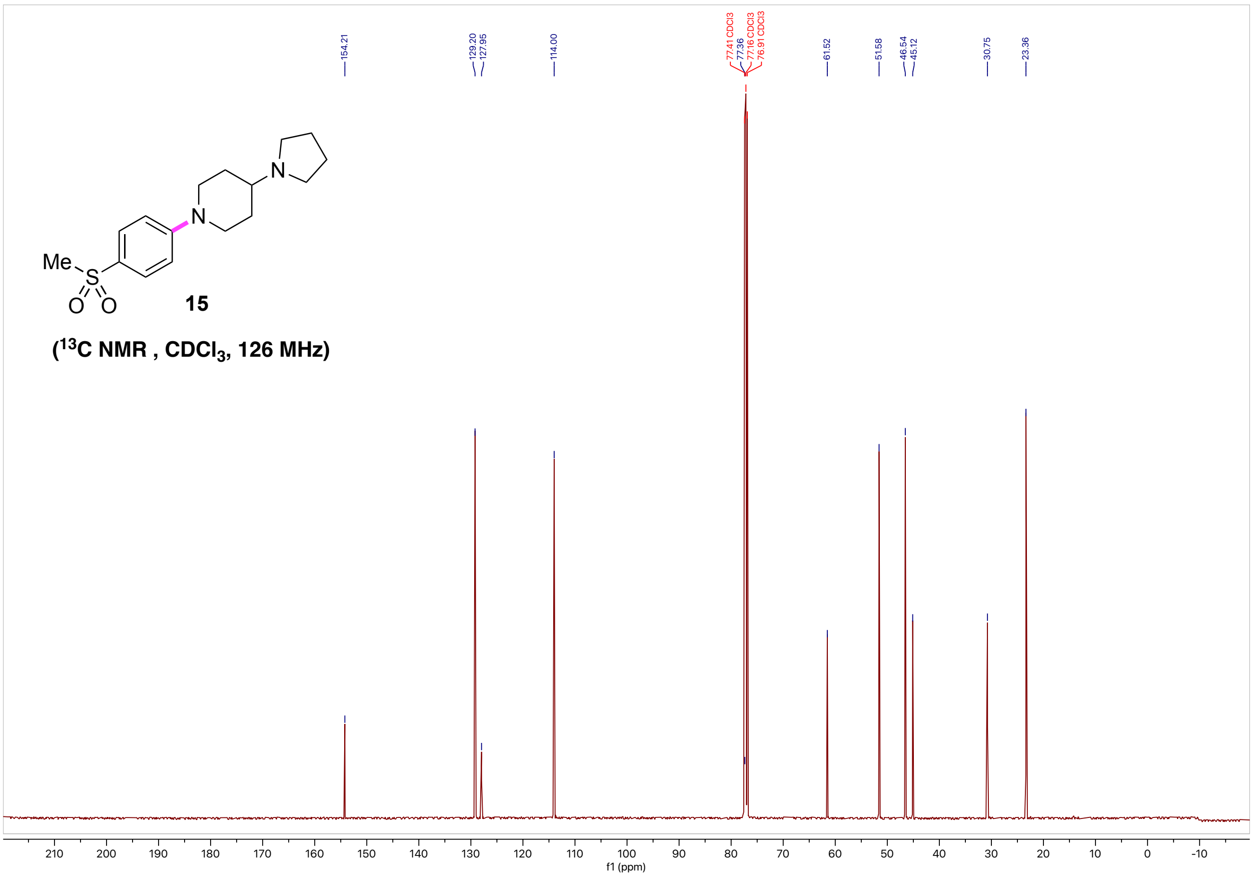
Me
S
O O
N
N
15
(¹³C NMR , CDCl₃, 126 MHz)
f1 (ppm)

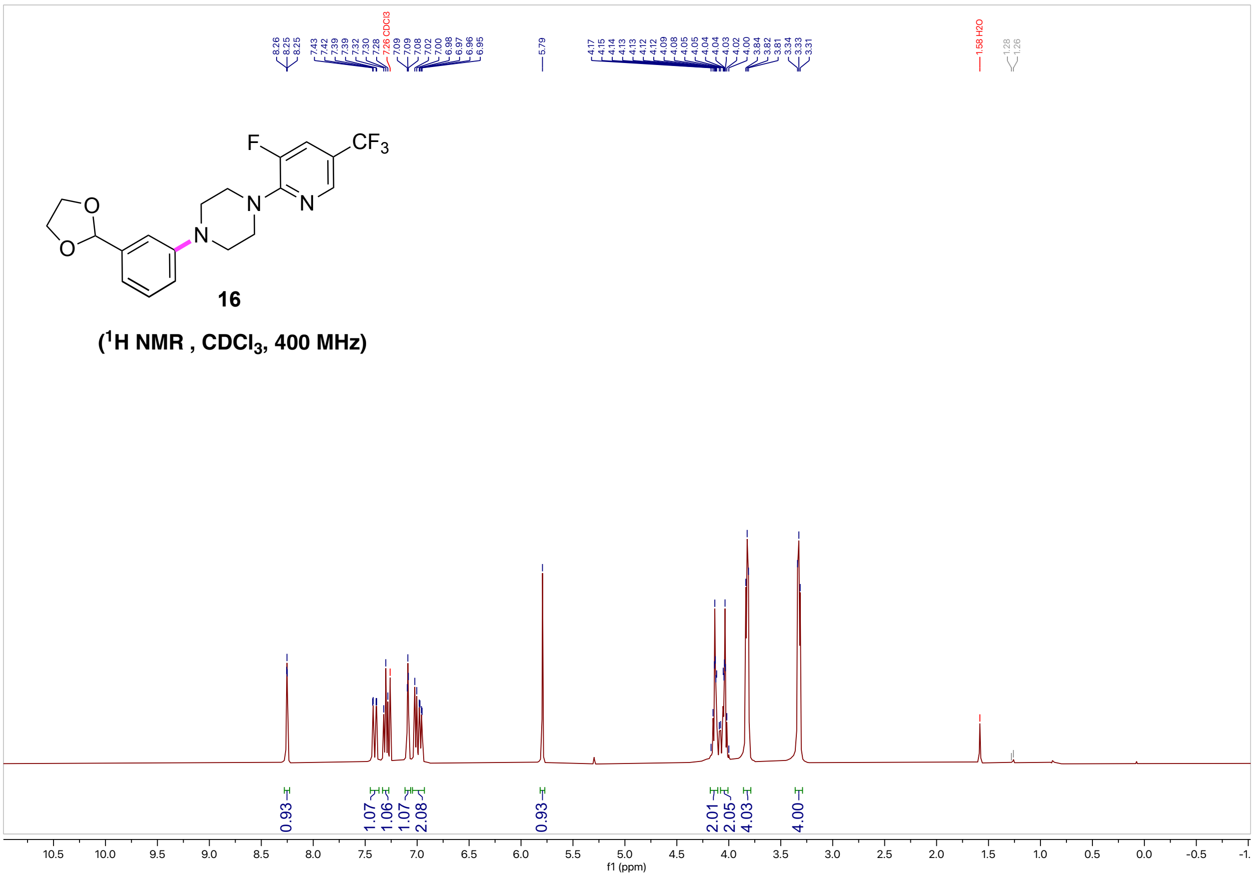
8.26
8.25
8.25
7.43
7.42
7.39
7.39
7.32
7.30
7.28
7.26 CDCl3
7.09
7.09
7.08
7.02
7.00
6.98
6.97
6.96
6.95
5.79
4.17
4.15
4.14
4.13
4.12
4.12
4.09
4.08
4.05
4.05
4.04
4.04
4.03
4.02
4.00
3.84
3.82
3.81
3.34
3.33
3.31
1.58 H2O
1.28
1.26
F
CF3
N
N
N
O
O
16
(1H NMR , CDCl3, 400 MHz)
0.93
1.07
1.06
1.07
2.08
0.93
2.01
2.05
4.03
4.00
f1 (ppm)

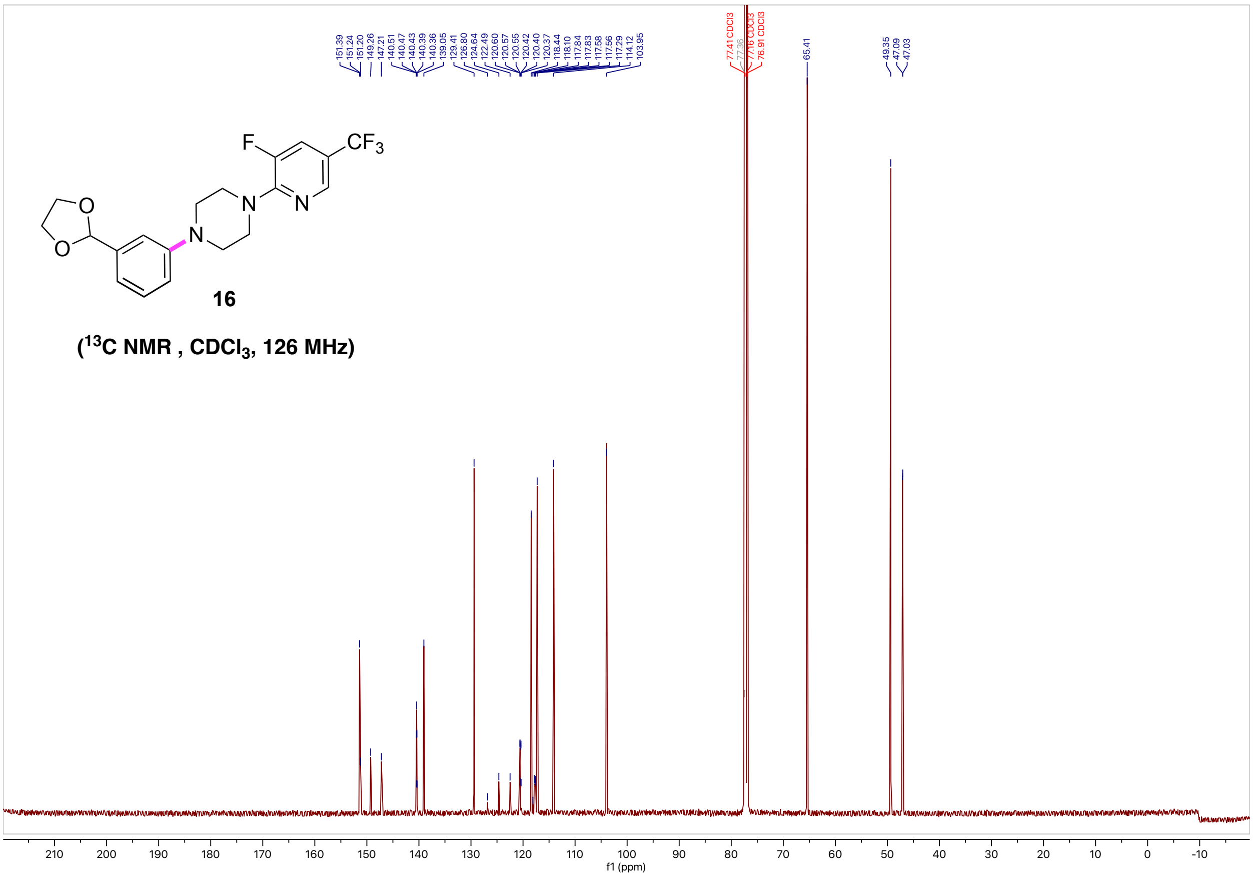
151.39
151.24
151.20
149.26
147.21
140.51
140.47
140.43
140.39
140.36
139.05
129.41
126.80
124.64
122.49
120.60
120.57
120.55
120.42
120.40
120.37
118.44
118.10
117.84
117.83
117.58
117.56
117.29
114.12
103.95
77.41 CDCl3
77.16 CDCl3
76.91 CDCl3
65.41
49.35
47.09
47.03
F
CF3
N
N
N
O
O
16
(13C NMR , CDCl3, 126 MHz)
f1 (ppm)

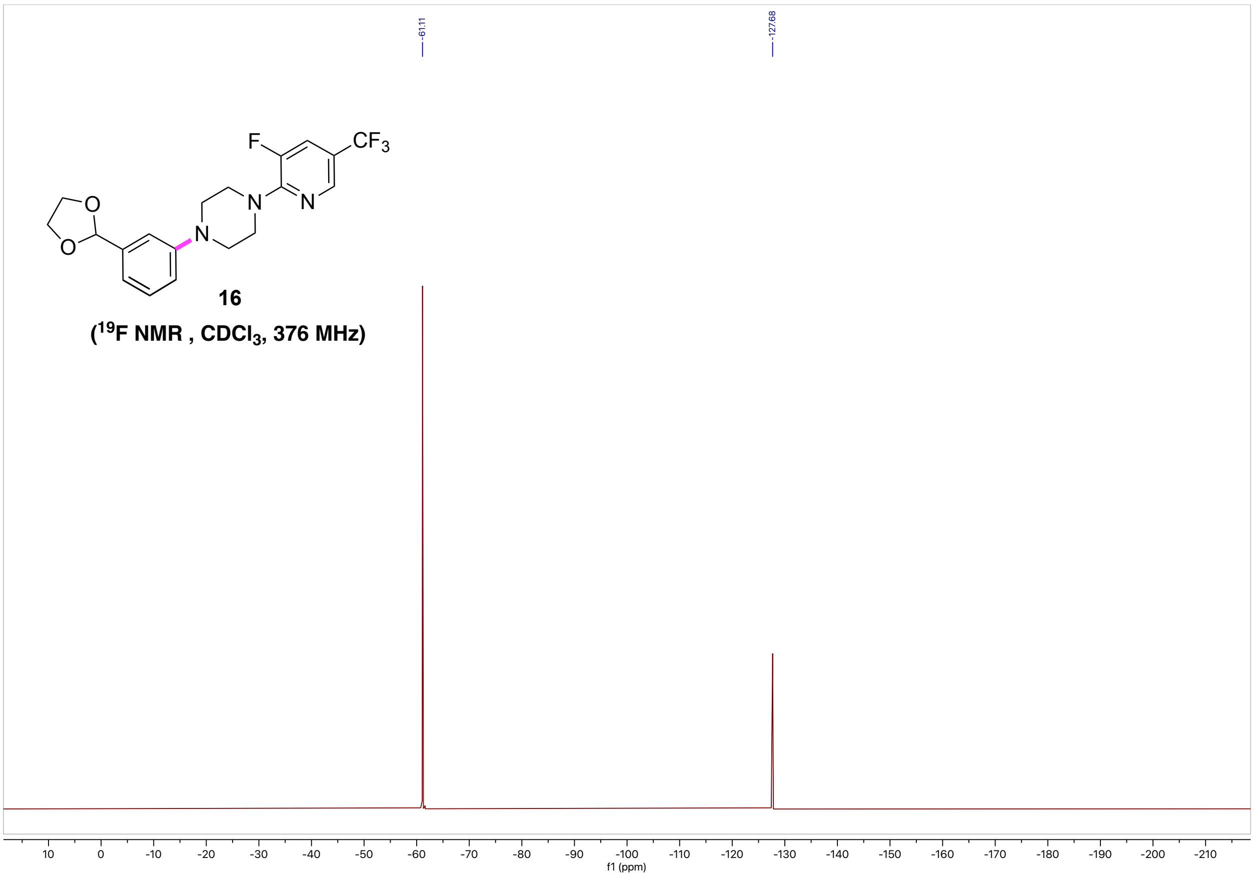

-61.11
-127.68
F
CF3
N
N
N
O
O
16
(19F NMR , CDCl3, 376 MHz)
10 0 -10 -20 -30 -40 -50 -60 -70 -80 -90 -100 -110 -120 -130 -140 -150 -160 -170 -180 -190 -200 -210
f1 (ppm)

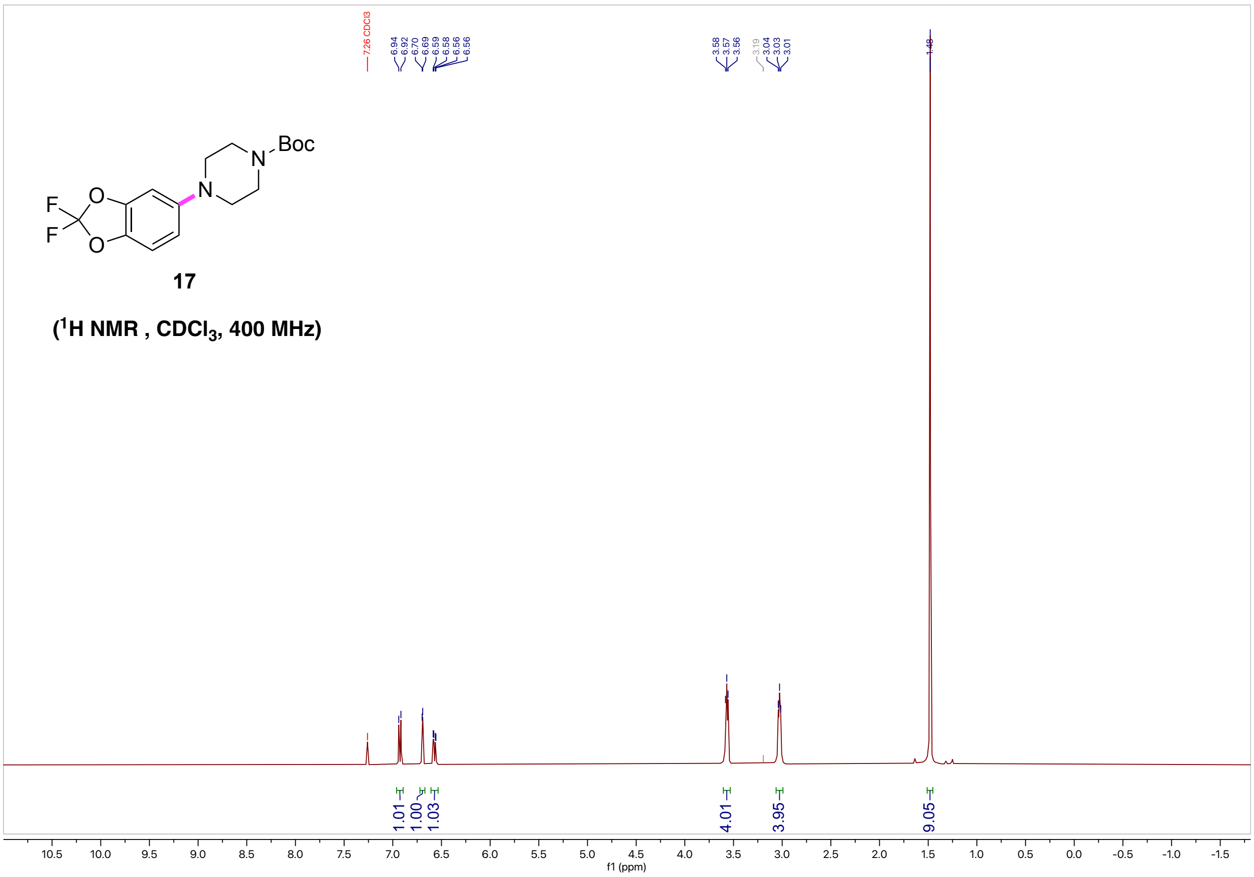
7.26 CDCl3
6.94
6.92
6.70
6.69
6.59
6.58
6.56
6.56
3.58
3.57
3.56
3.19
3.04
3.03
3.01
1.48
Boc
N
N
F
F
O
O
17
(¹H NMR , CDCl3, 400 MHz)
1.01
1.00
1.03
4.01
3.95
9.05
f1 (ppm)

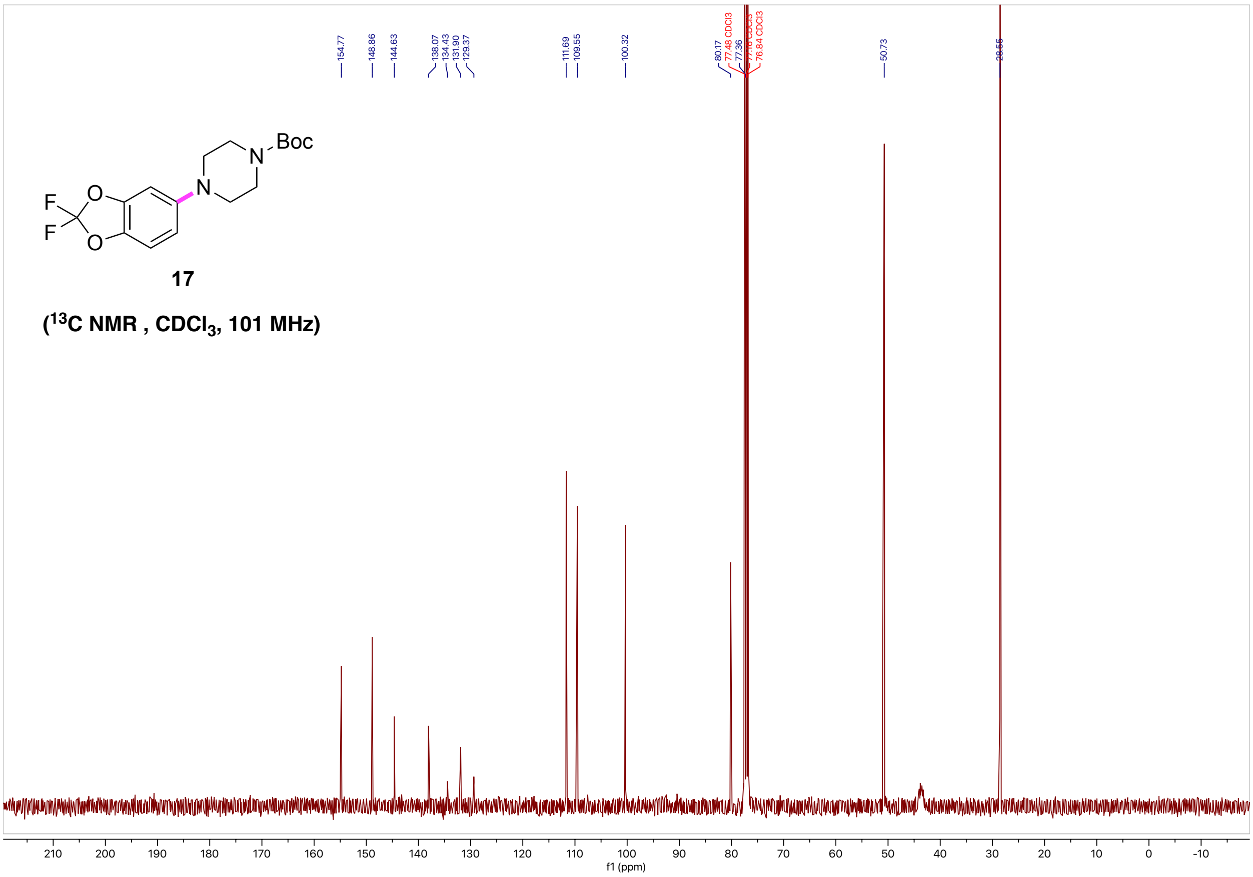
154.77
148.86
144.63
138.07
134.43
131.90
129.37
111.69
109.55
100.32
80.17
77.48 CDCl3
77.36
76.84 CDCl3
50.73
28.55
Boc
N
N
F
F
O
O
17
(¹³C NMR , CDCl3, 101 MHz)
f1 (ppm)

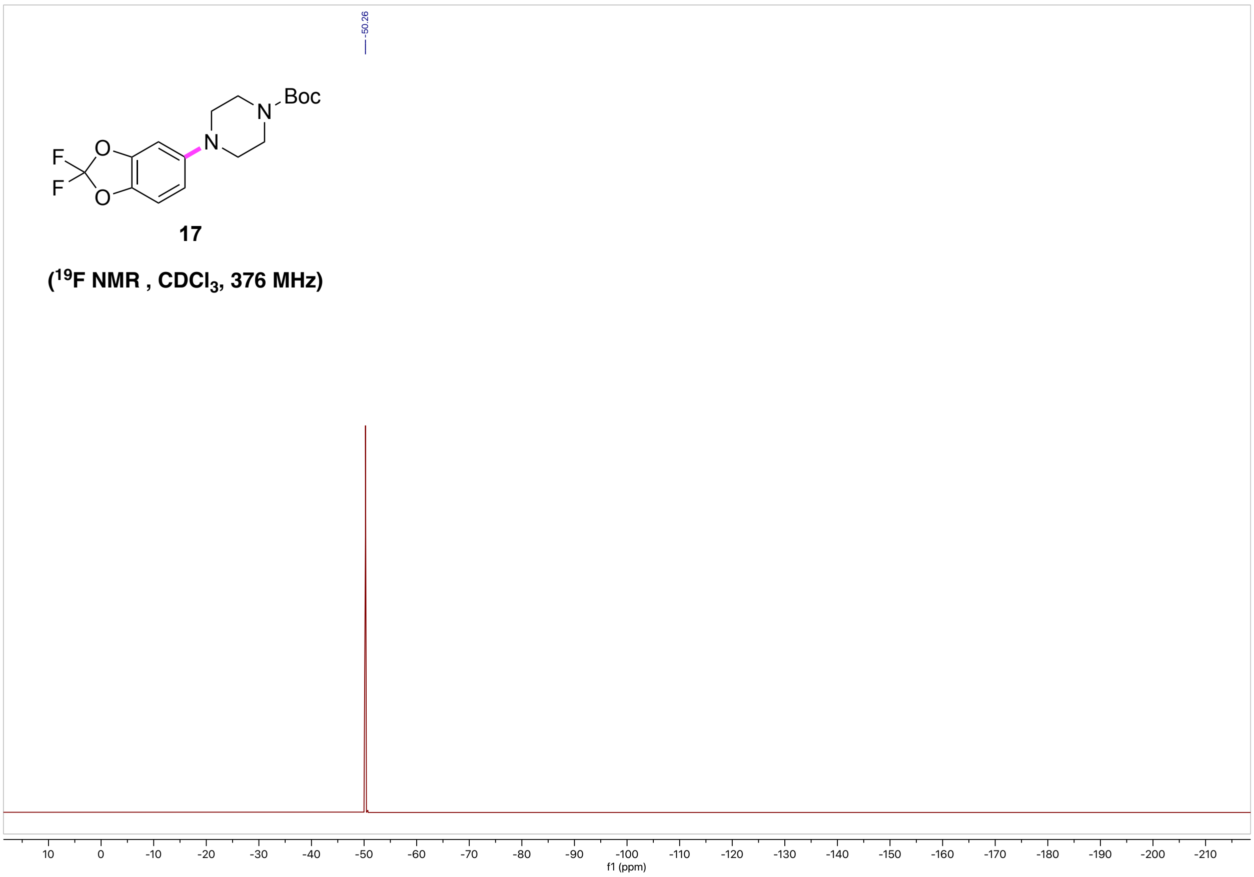
-50.26
Boc
N
N
F
F
O
O
17
(19F NMR , CDCl3, 376 MHz)
10 0 -10 -20 -30 -40 -50 -60 -70 -80 -90 -100 -110 -120 -130 -140 -150 -160 -170 -180 -190 -200 -210
f1 (ppm)

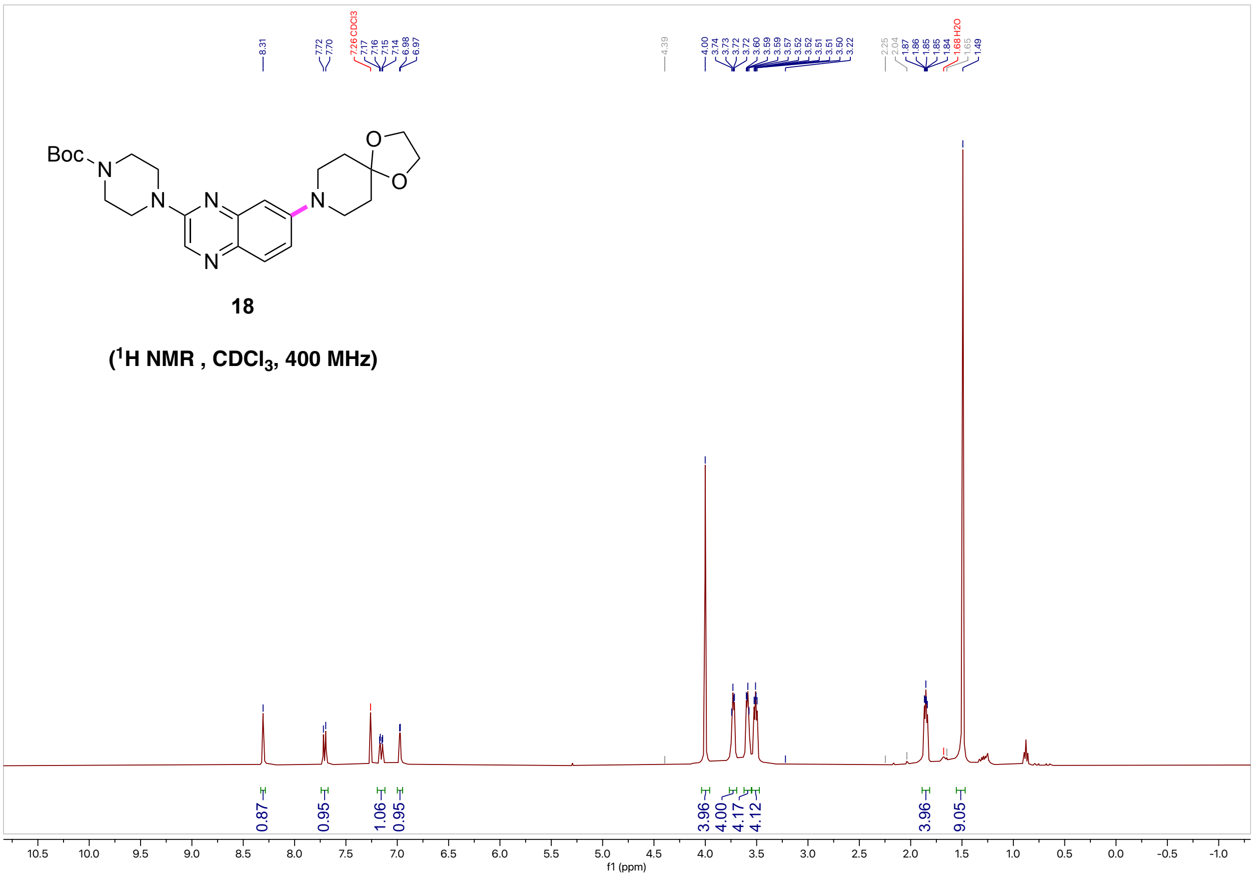

18

(^{1}H NMR , $CDCl_3$, 400 MHz)

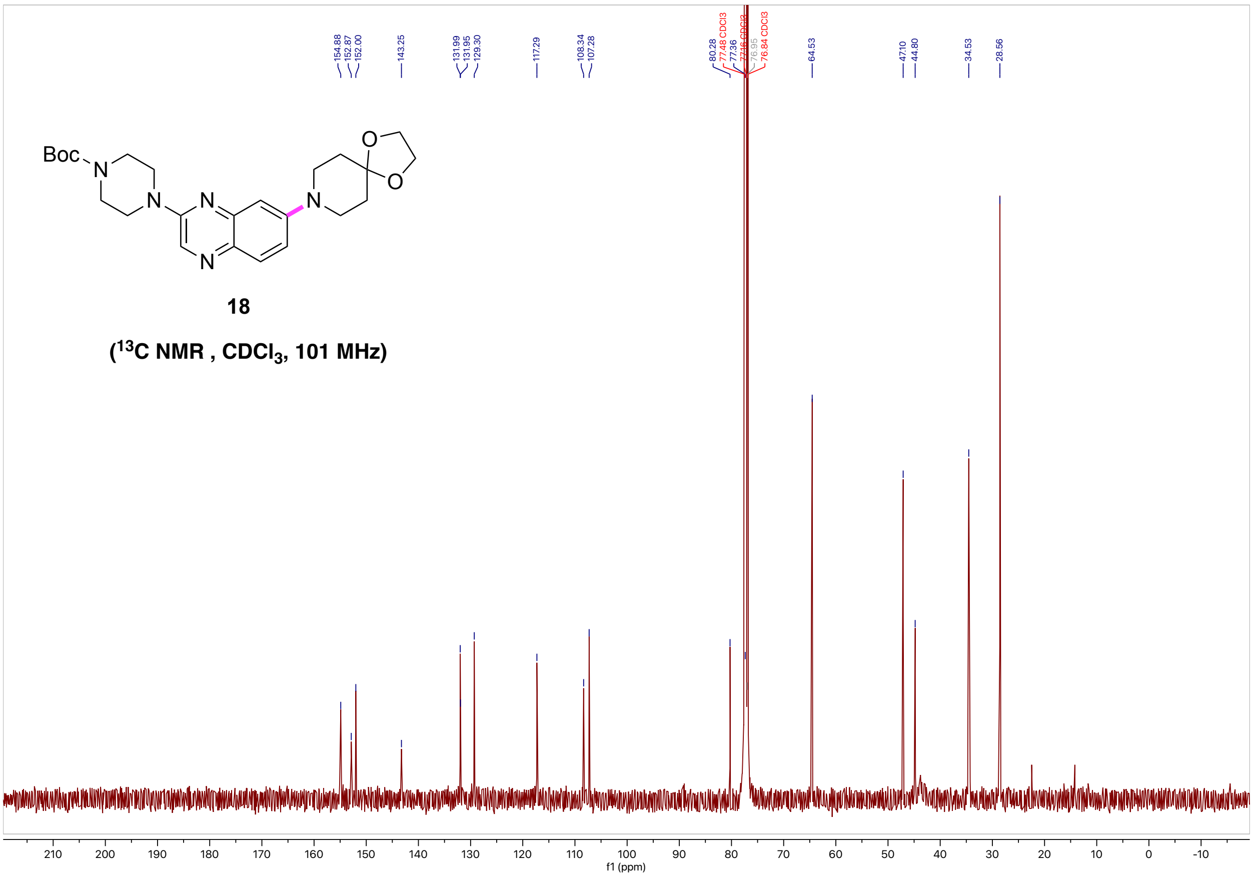

18

(^{13}C NMR , $CDCl_3$, 101 MHz)

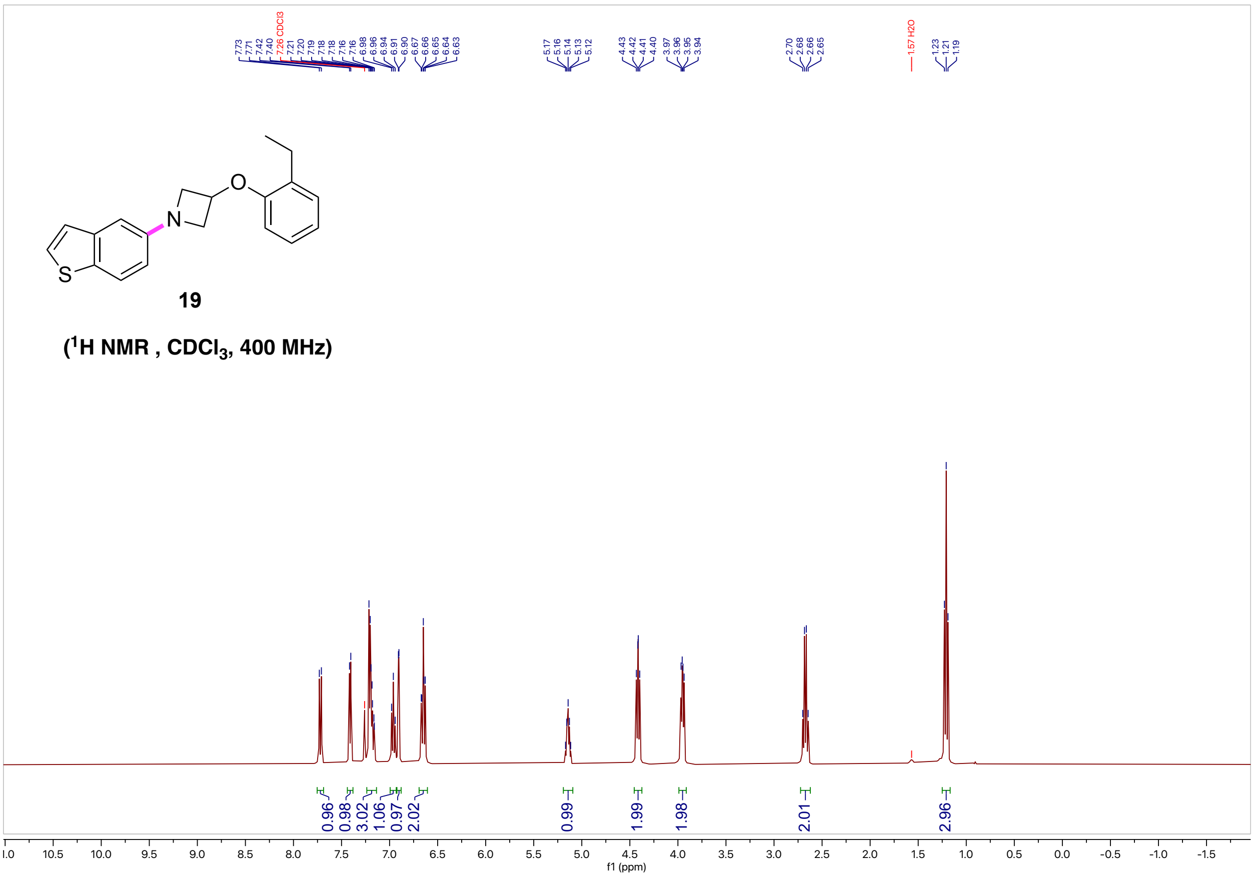
7.73
7.71
7.42
7.40
7.26 CDCl3
7.21
7.20
7.19
7.18
7.18
7.16
7.16
6.98
6.96
6.94
6.91
6.90
6.67
6.66
6.65
6.64
6.63
5.17
5.16
5.14
5.13
5.12
4.43
4.42
4.41
4.40
3.97
3.96
3.95
3.94
2.70
2.68
2.66
2.65
1.57 H2O
1.23
1.21
1.19
N
O
S
19
(1H NMR , CDCl3, 400 MHz)
0.96
0.98
3.02
1.06
0.97
2.02
0.99
1.99
1.98
2.01
2.96
f1 (ppm)

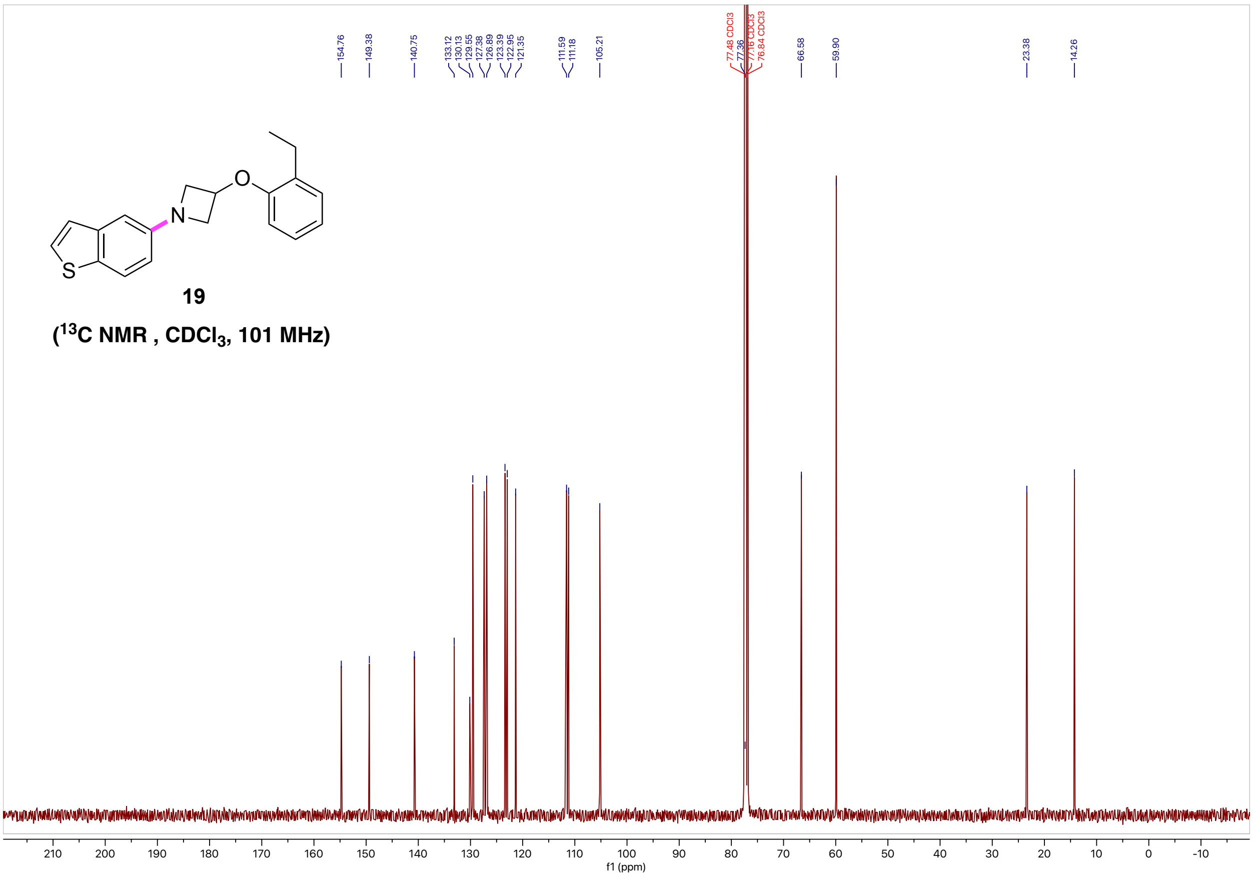
154.76
149.38
140.75
133.12
130.13
129.55
127.38
126.89
123.39
122.95
121.35
111.59
111.18
105.21
77.48 CDCl3
77.36
77.16 CDCl3
76.84 CDCl3
66.58
59.90
23.38
14.26
N
O
S
19
(13C NMR , CDCl3, 101 MHz)
f1 (ppm)

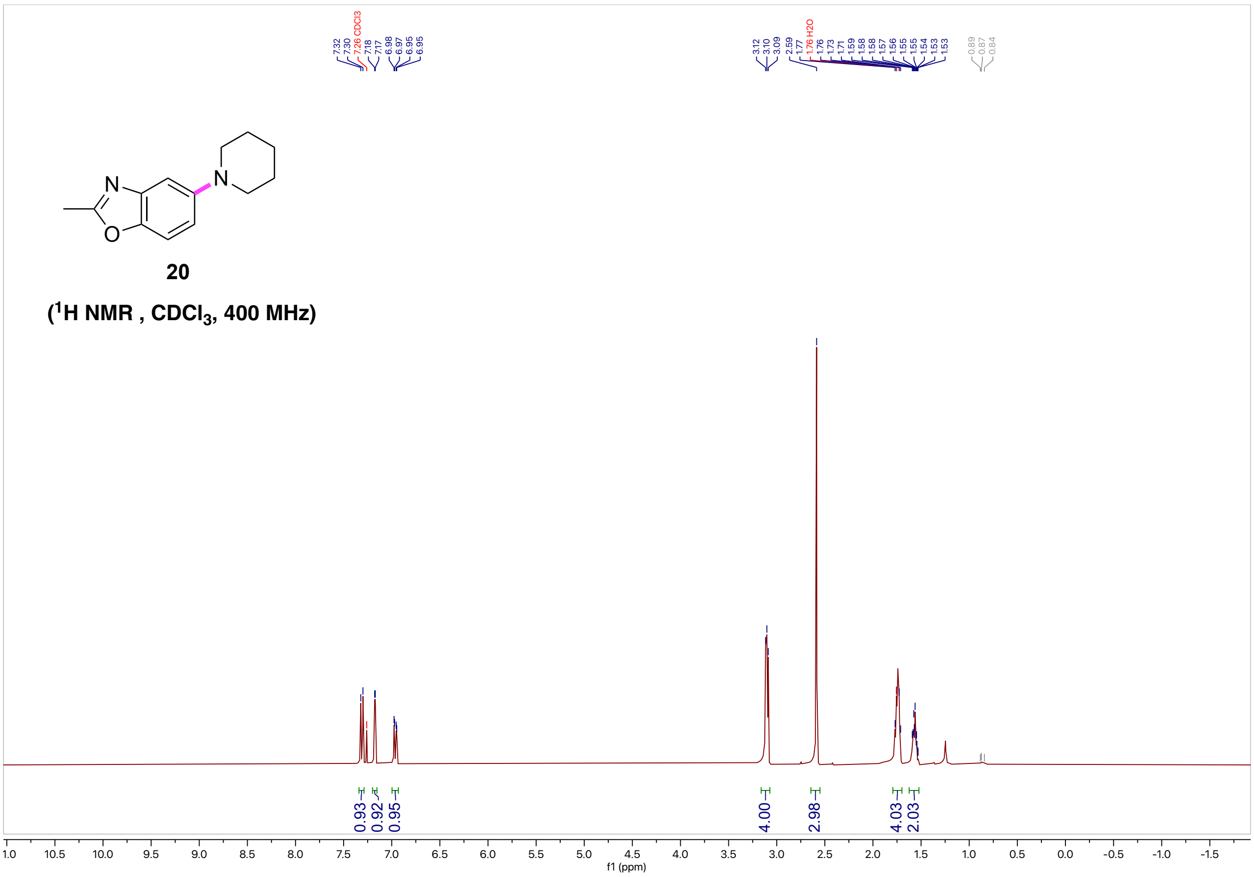

20

(^{1}H NMR , $CDCl_3$, 400 MHz)

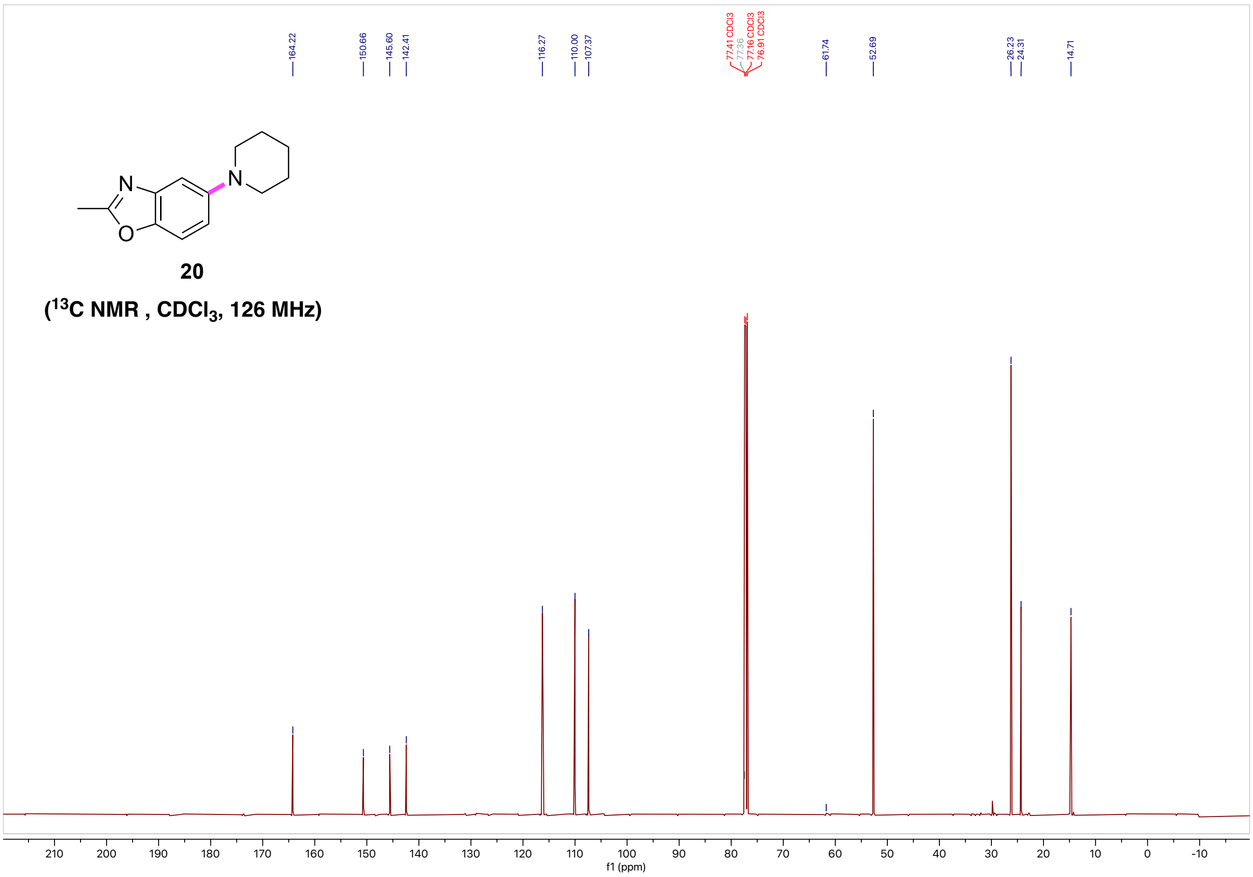

20

(^{13}C NMR , $CDCl_3$, 126 MHz)

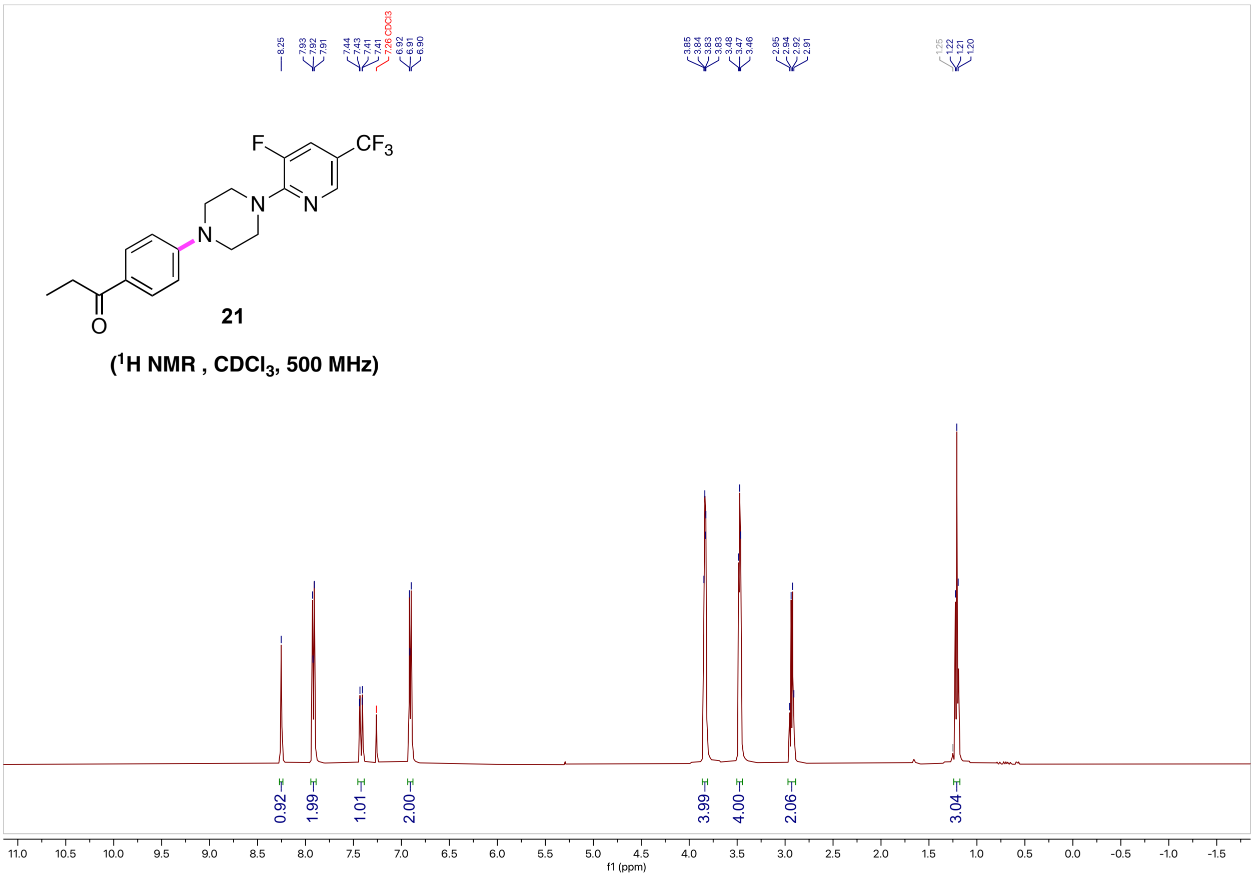
F
CF3
N
N
N
O
21
(1H NMR , CDCl3, 500 MHz)
f1 (ppm)

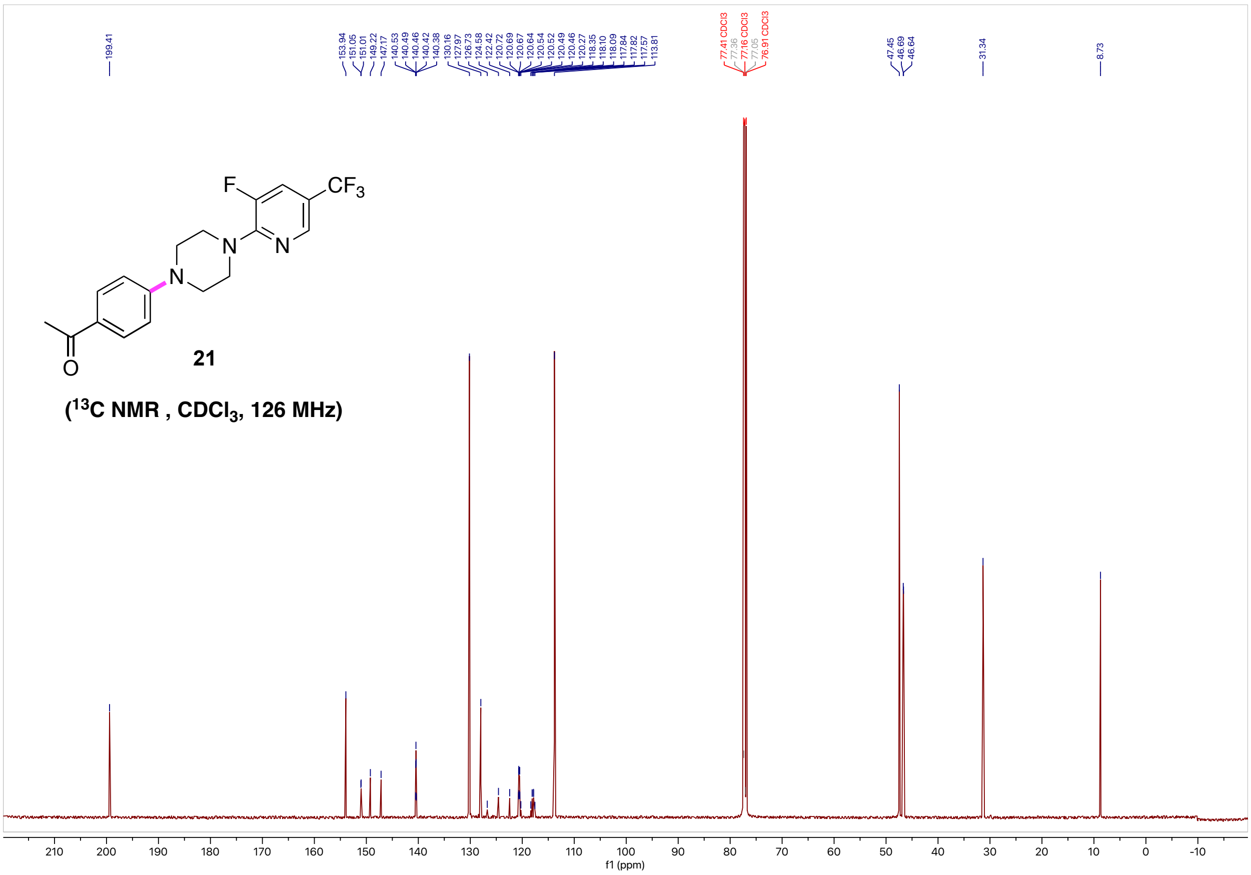
F
CF3
N
N
N
O
21
(13C NMR , CDCl3, 126 MHz)
f1 (ppm)

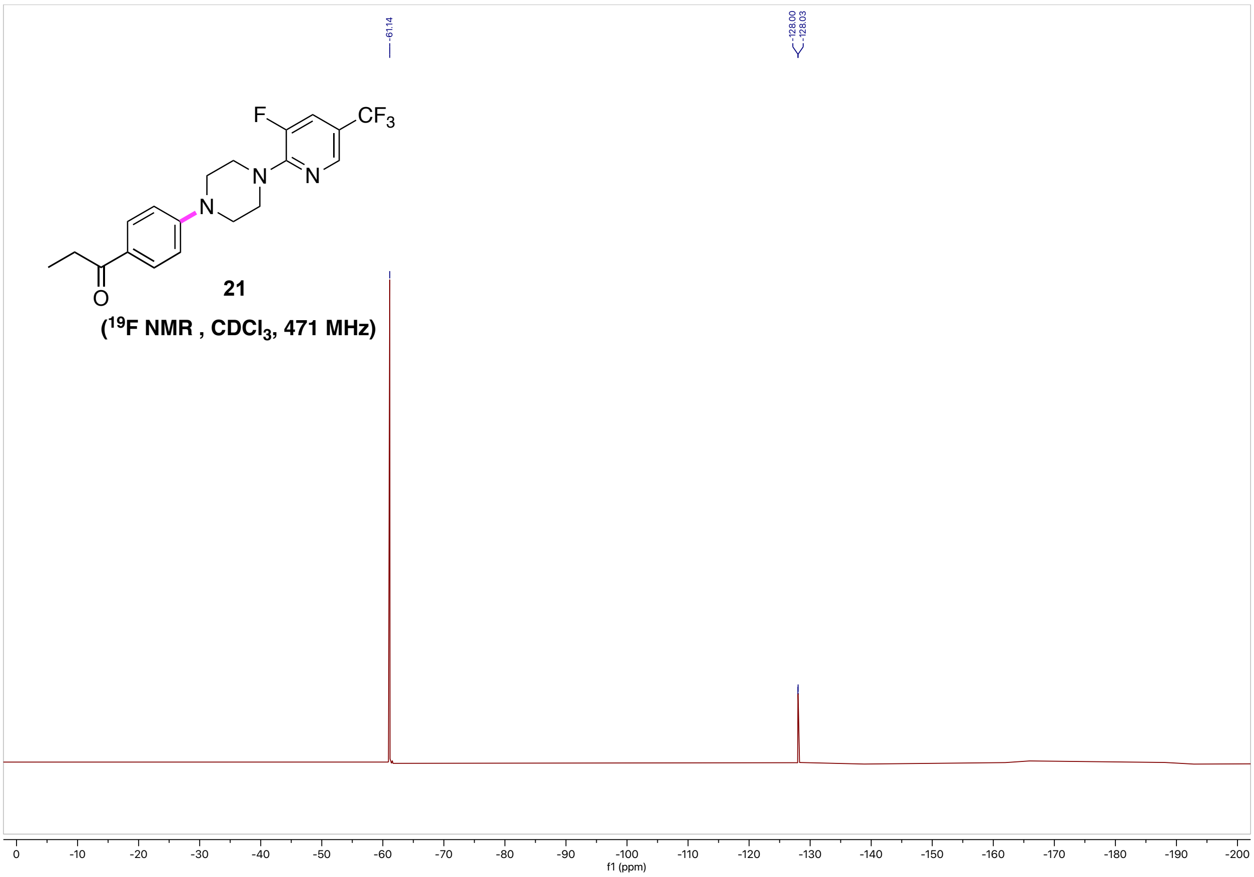

-61.14
-128.00
-128.03
F
CF3
N
N
N
O
21
(19F NMR , CDCl3, 471 MHz)
0
-10
-20
-30
-40
-50
-60
-70
-80
-90
-100
-110
-120
-130
-140
-150
-160
-170
-180
-190
-200
f1 (ppm)

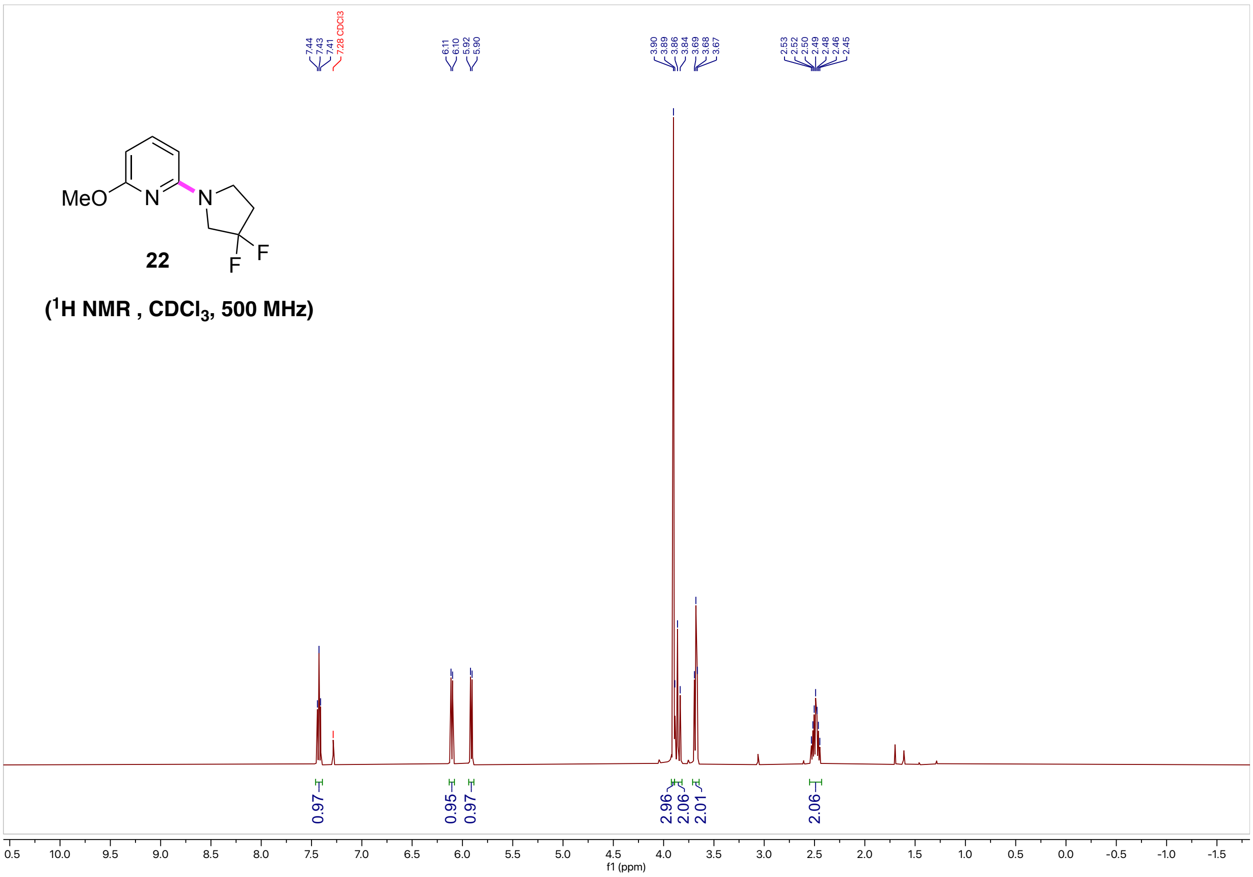
MeO
N
N
F
F
22
(¹H NMR , CDCl₃, 500 MHz)
f1 (ppm)

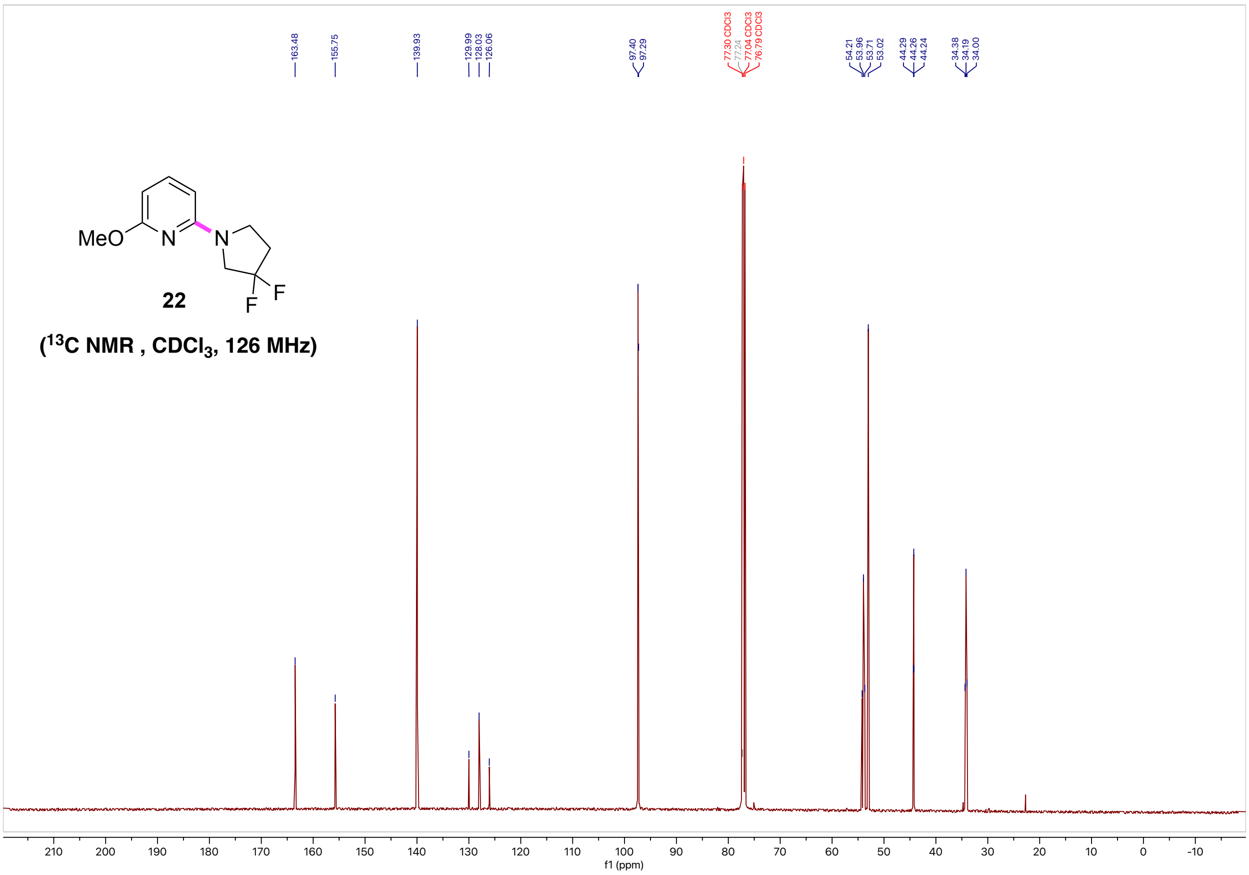
MeO
N
N
F
F
22
(¹³C NMR , CDCl₃, 126 MHz)
f1 (ppm)

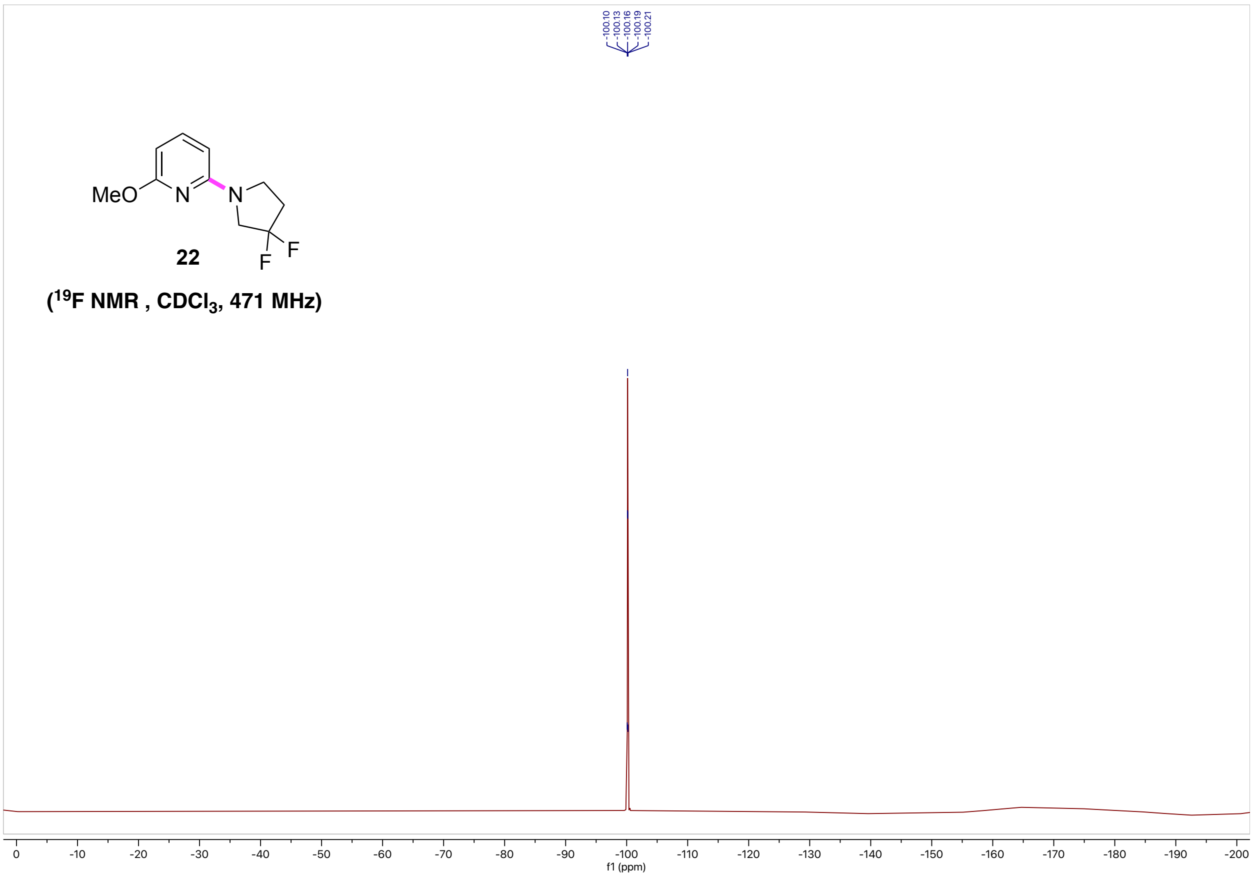
-100.10
-100.13
-100.16
-100.19
-100.21
MeO
N
N
F
F
22
(19F NMR , CDCl3, 471 MHz)
0
-10
-20
-30
-40
-50
-60
-70
-80
-90
-100
-110
-120
-130
-140
-150
-160
-170
-180
-190
-200
f1 (ppm)

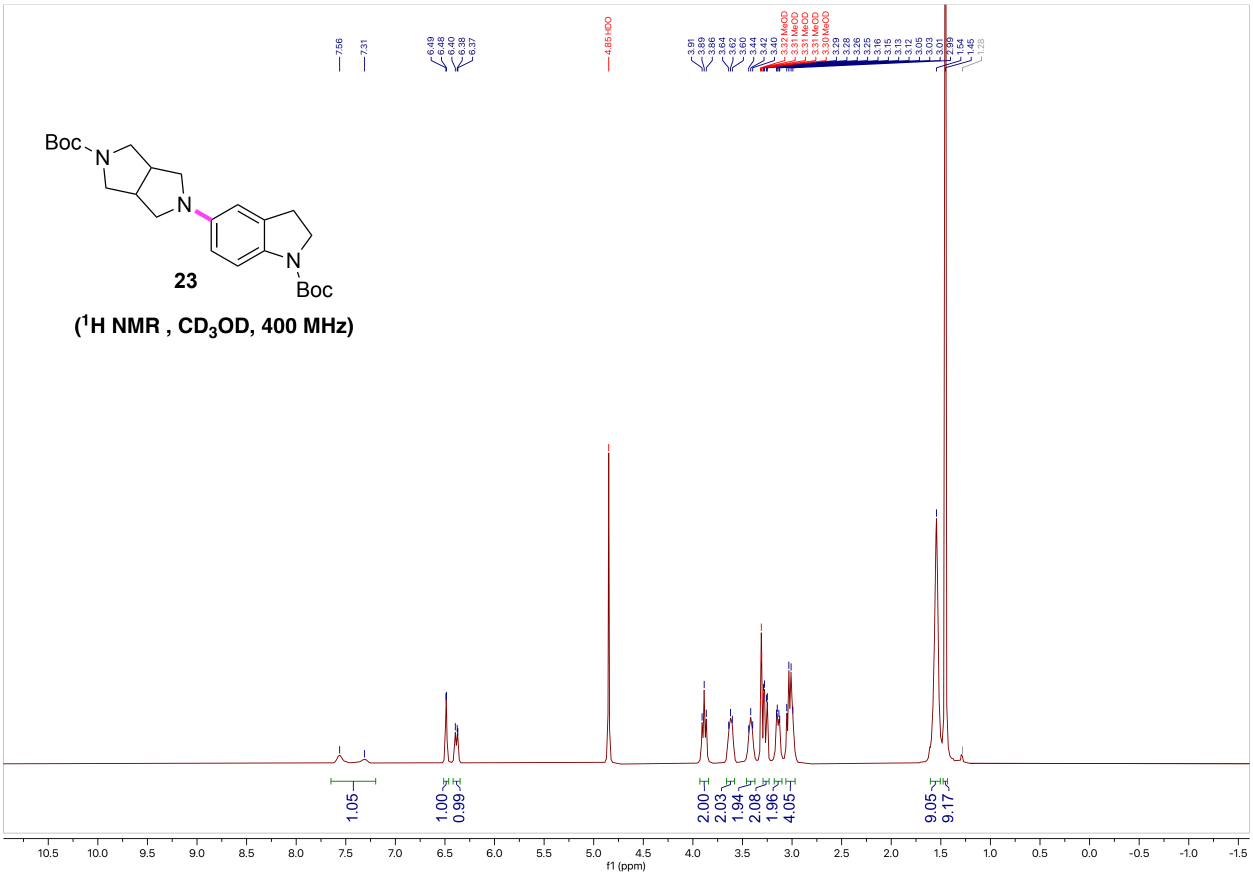
23
(^{1}H NMR , CD_3OD, 400 MHz)

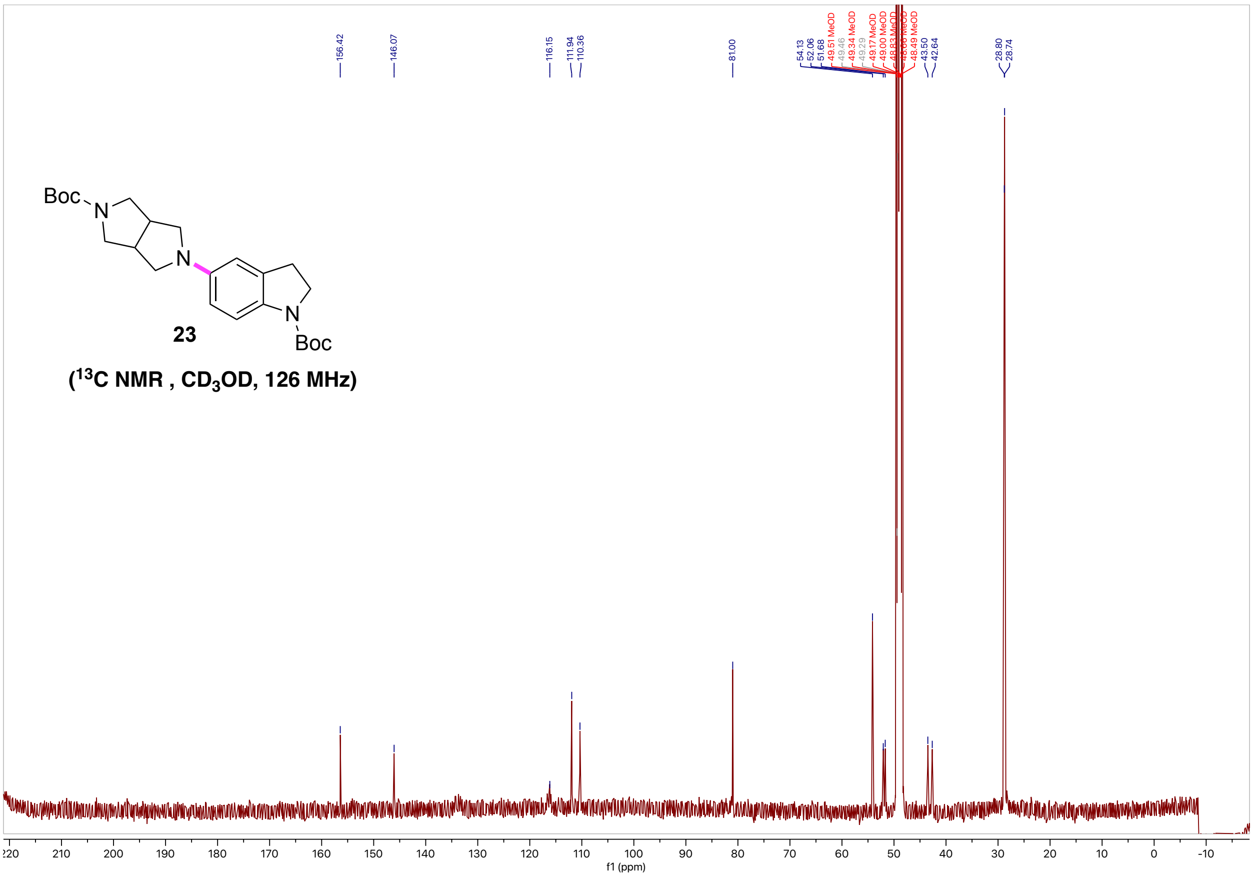
23
(^{13}C NMR , CD_3OD, 126 MHz)

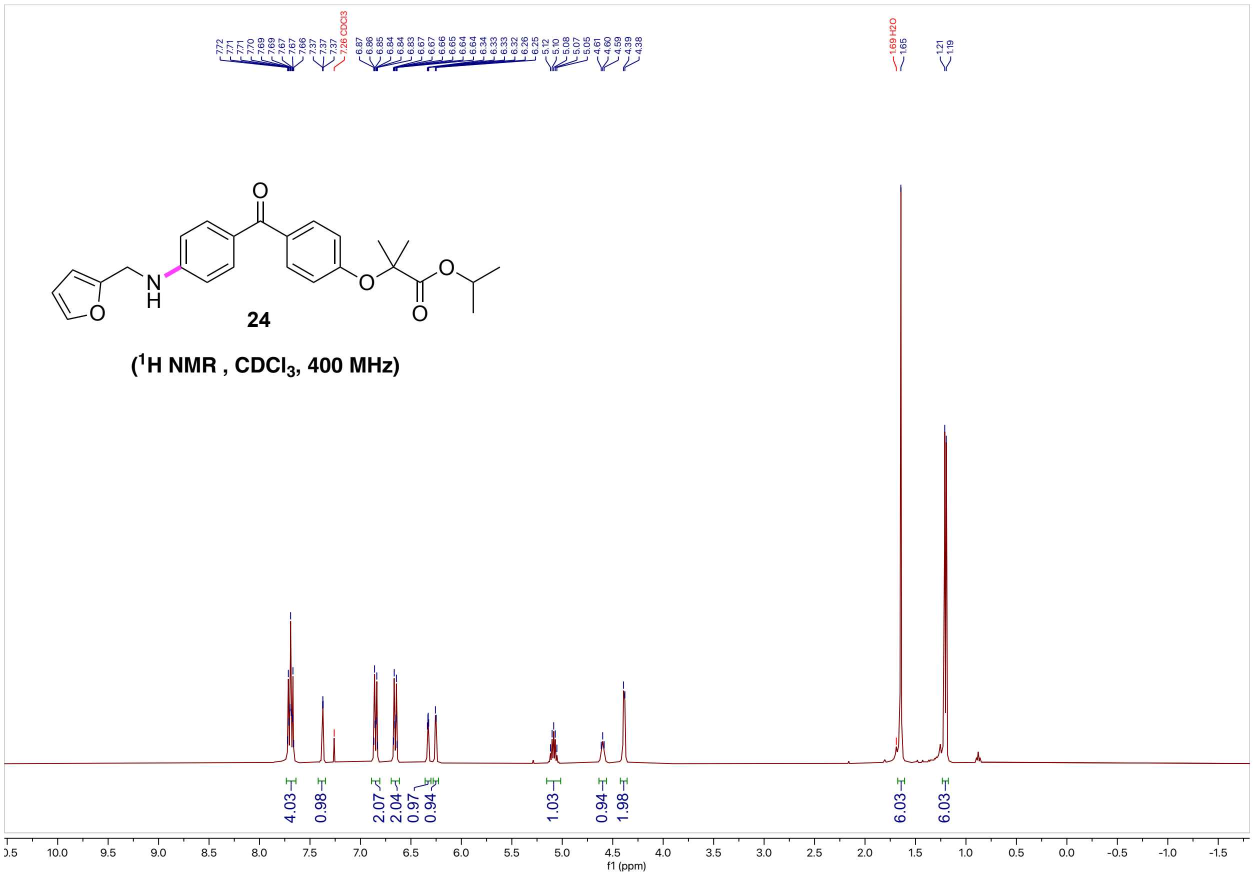
24
(¹H NMR , CDCl₃, 400 MHz)
f1 (ppm)

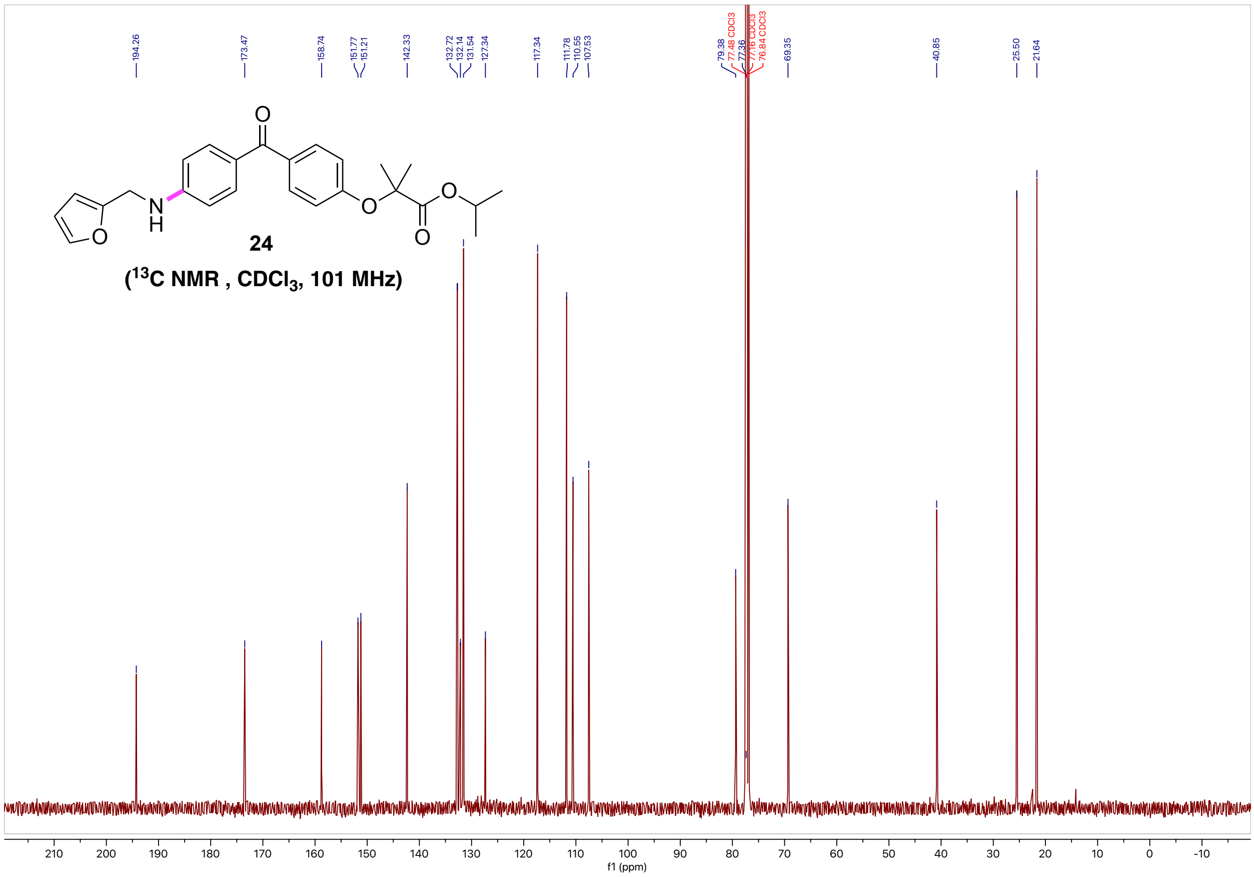
24
(¹³C NMR , CDCl₃, 101 MHz)
f1 (ppm)

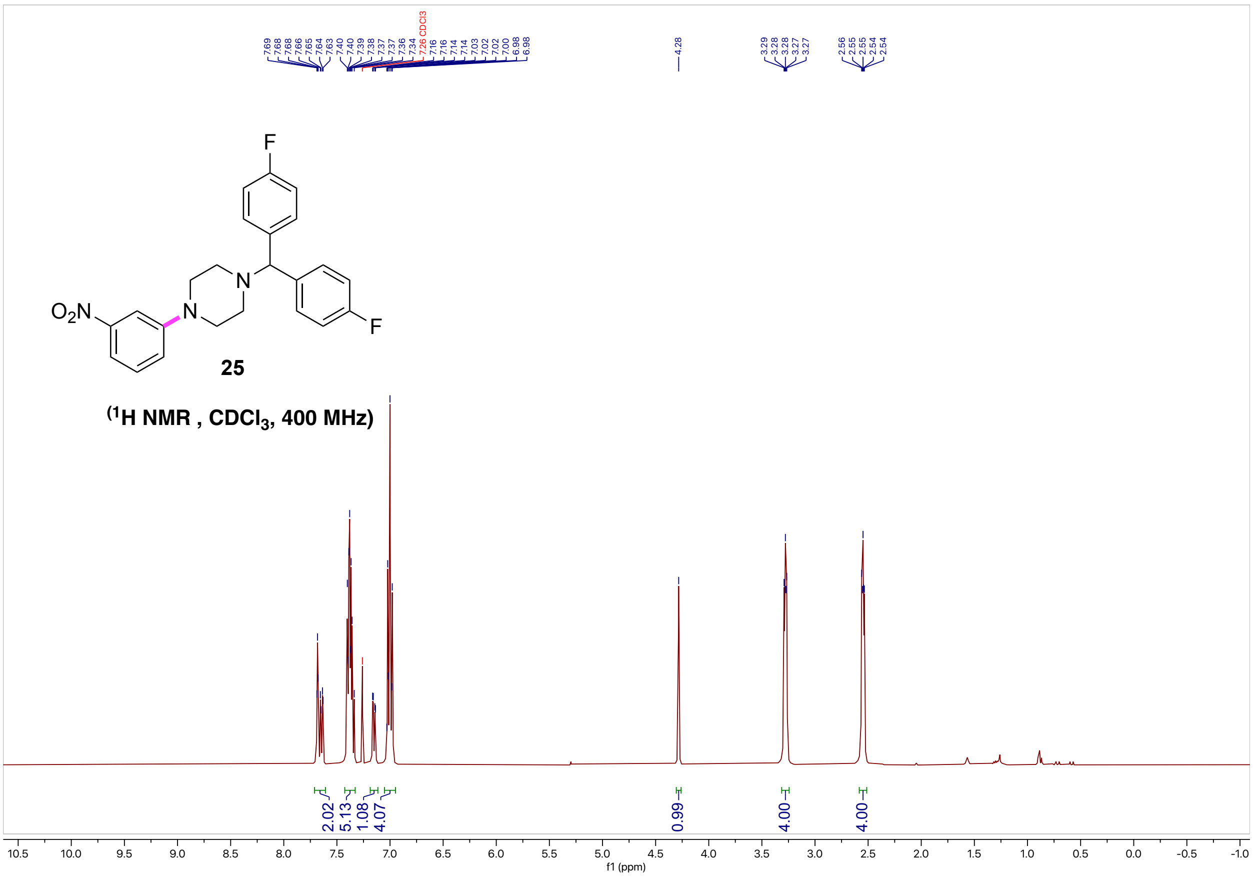
25
(^{1}H NMR , CDCl$_3$, 400 MHz)

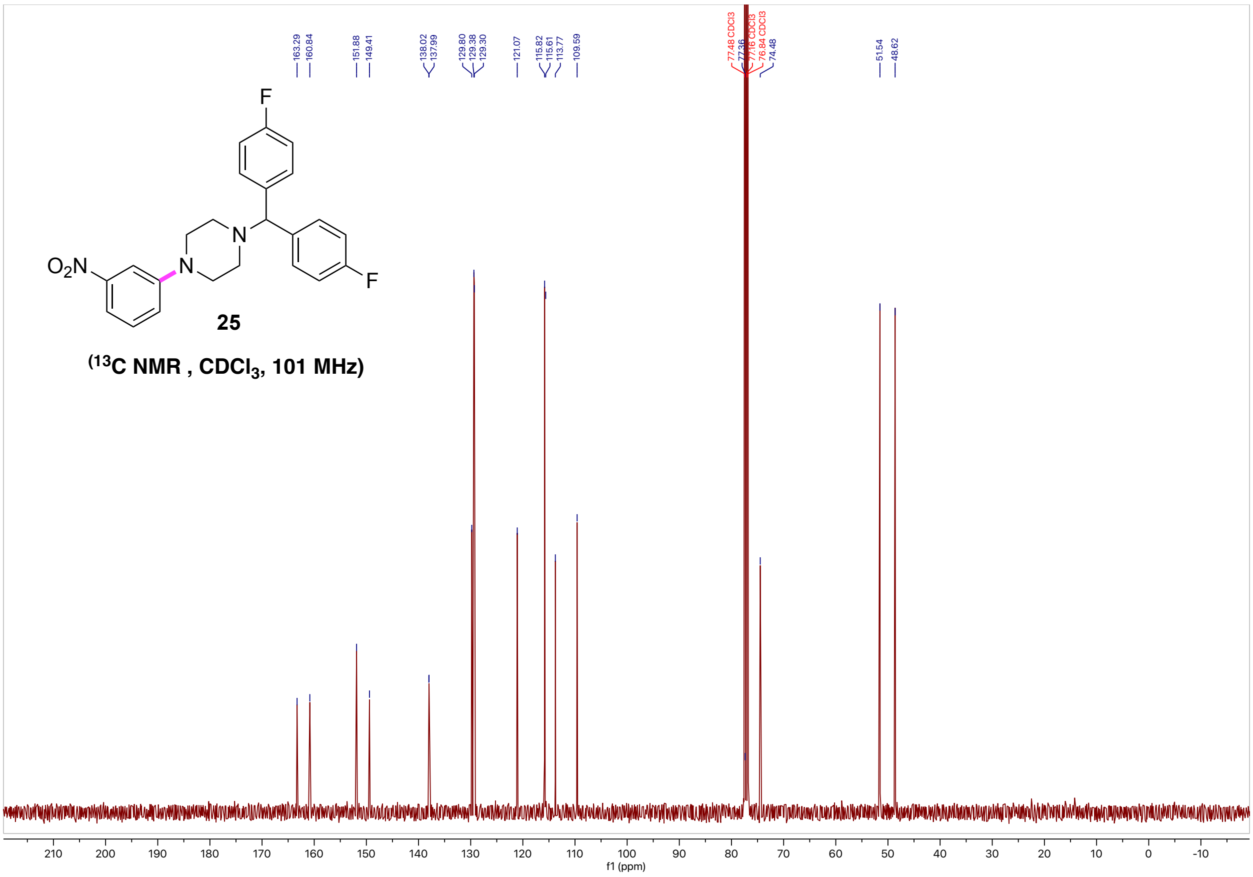
25
(^{13}C NMR , CDCl$_3$, 101 MHz)

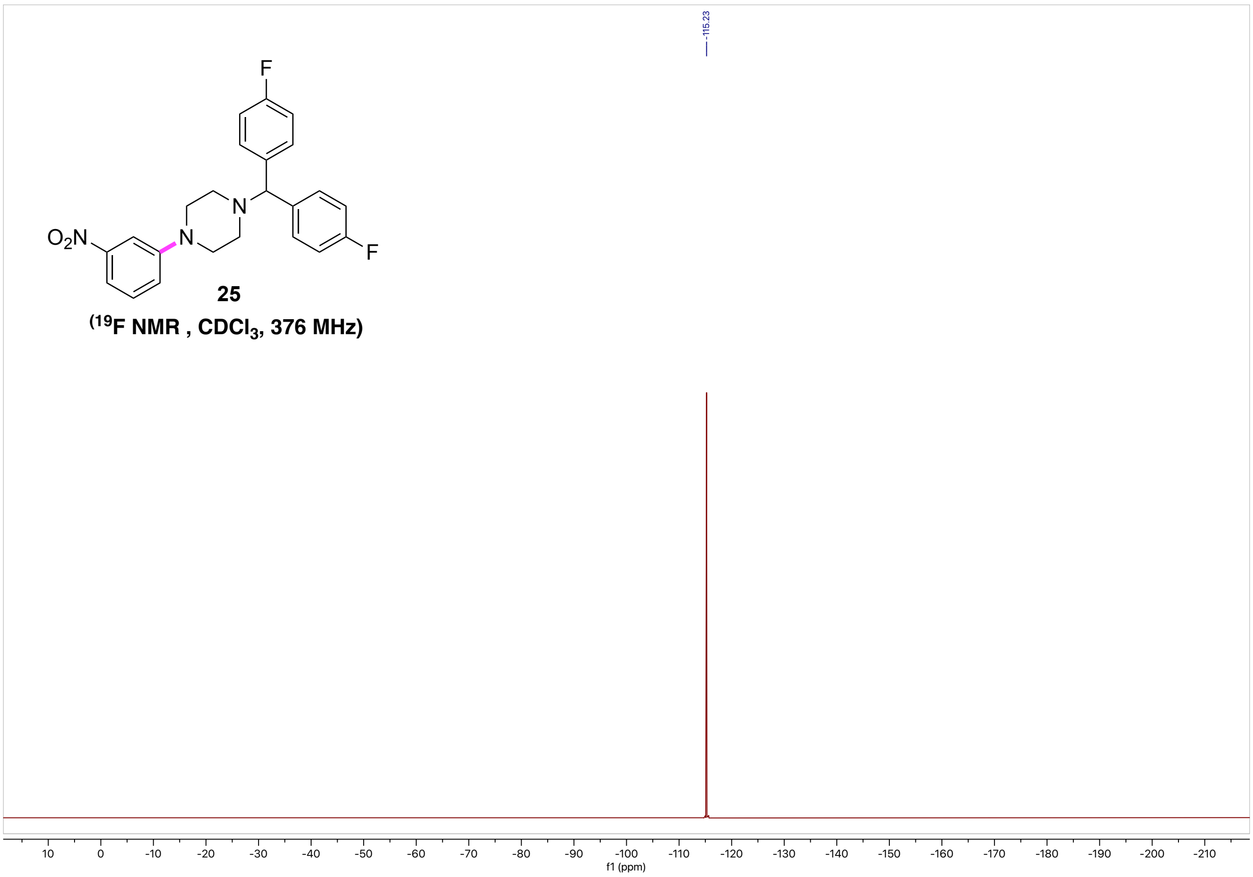
-115.23
F
F
O_2N
N
N
25
(^{19}F NMR , $CDCl_3$, 376 MHz)
10 0 -10 -20 -30 -40 -50 -60 -70 -80 -90 -100 -110 -120 -130 -140 -150 -160 -170 -180 -190 -200 -210
f1 (ppm)

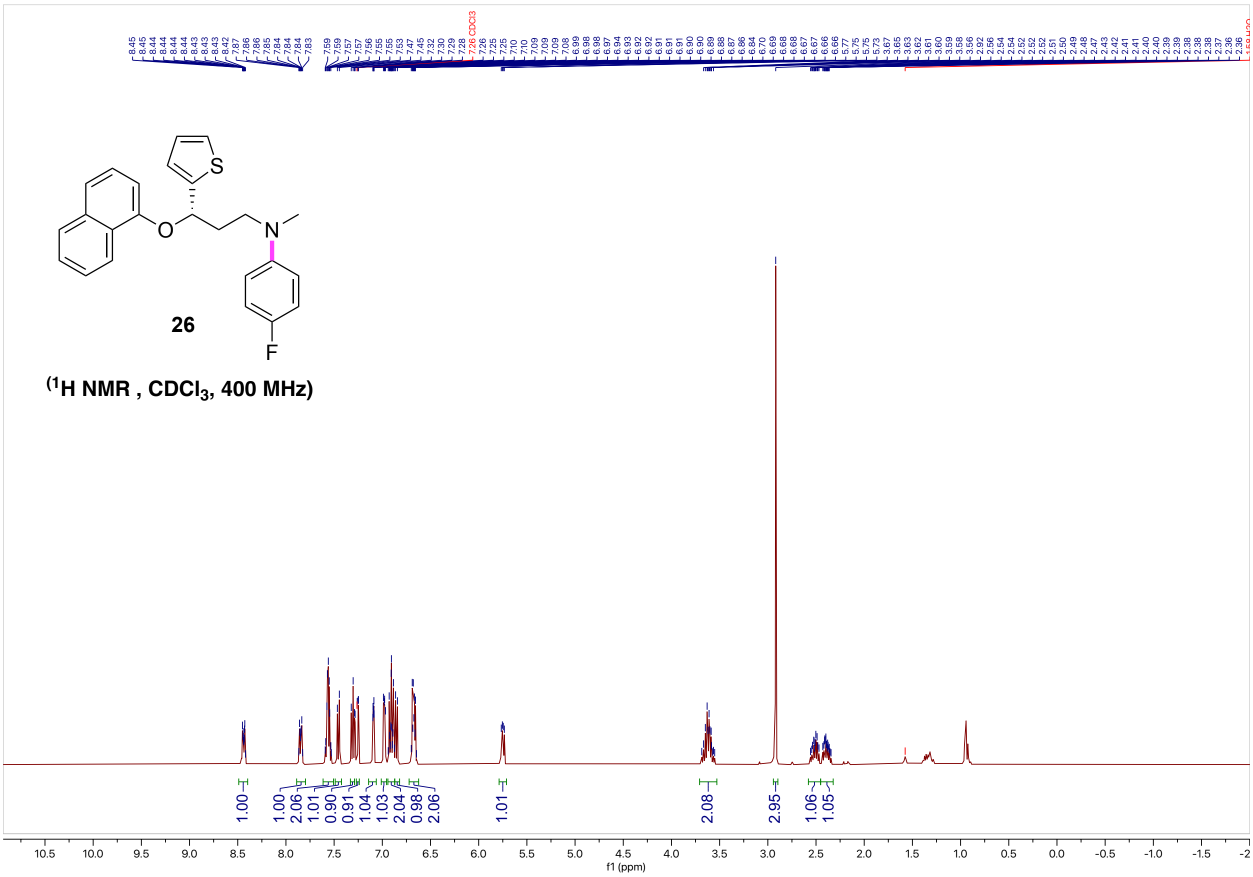
26
(^{1}H NMR , $CDCl_3$, 400 MHz)
f1 (ppm)

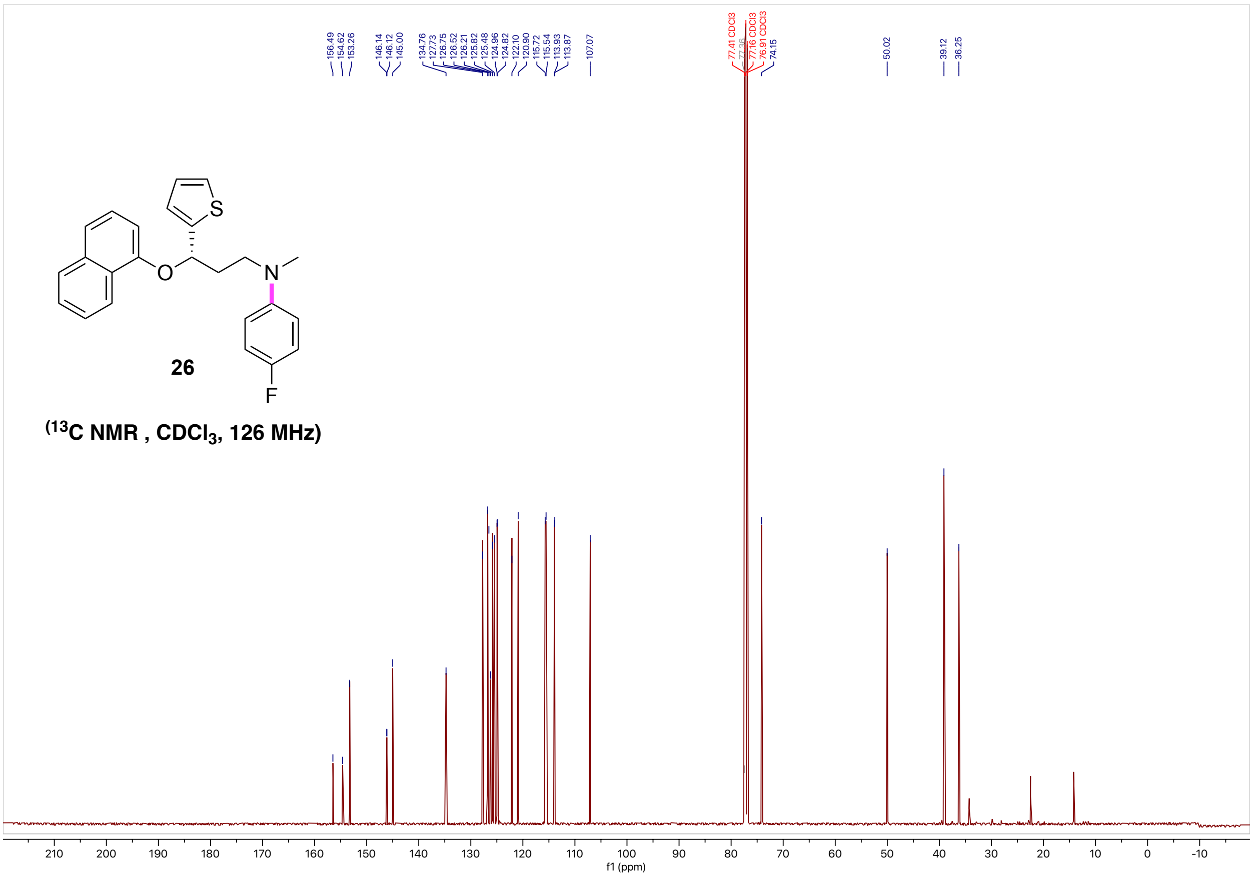
26
(^{13}C NMR , $CDCl_3$, 126 MHz)
f1 (ppm)

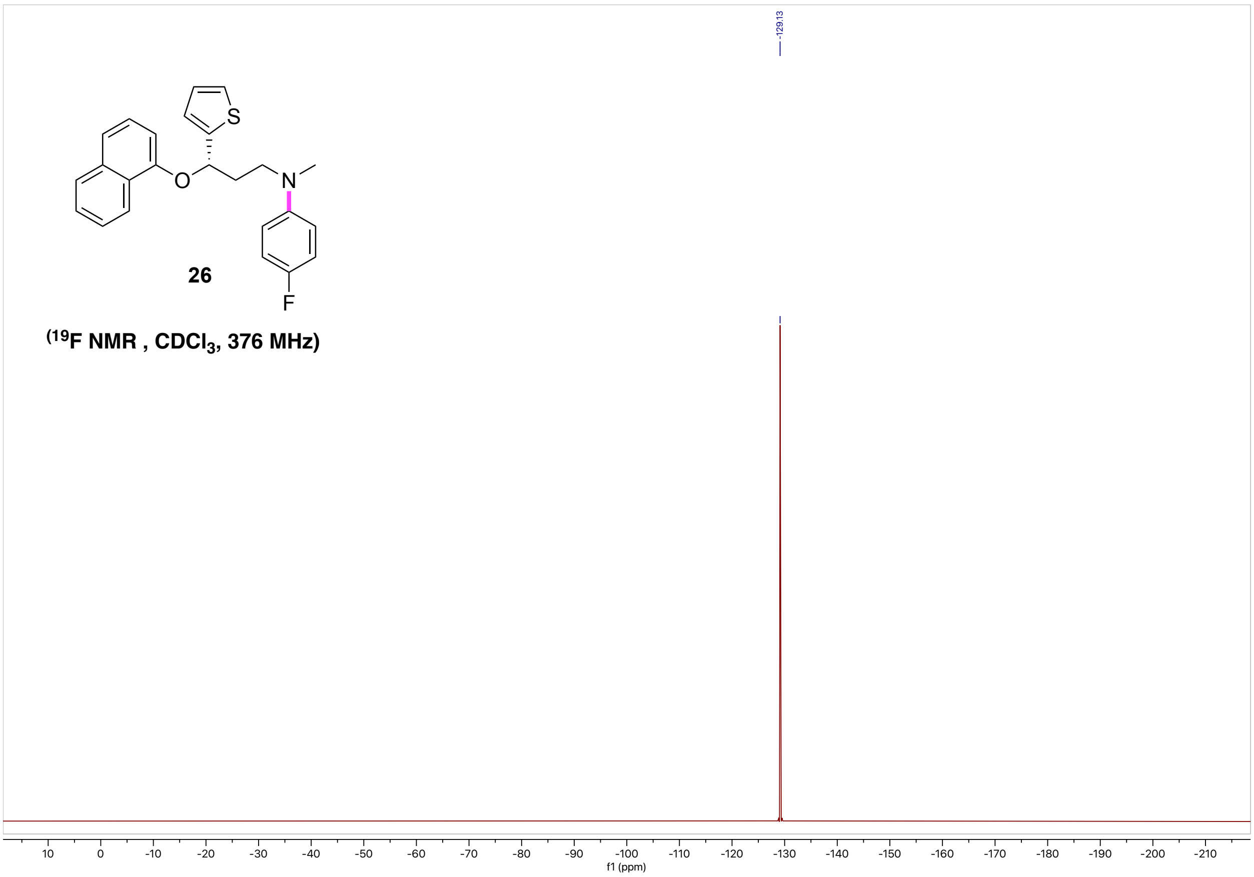

26

(^{19}F NMR , $CDCl_3$, 376 MHz)

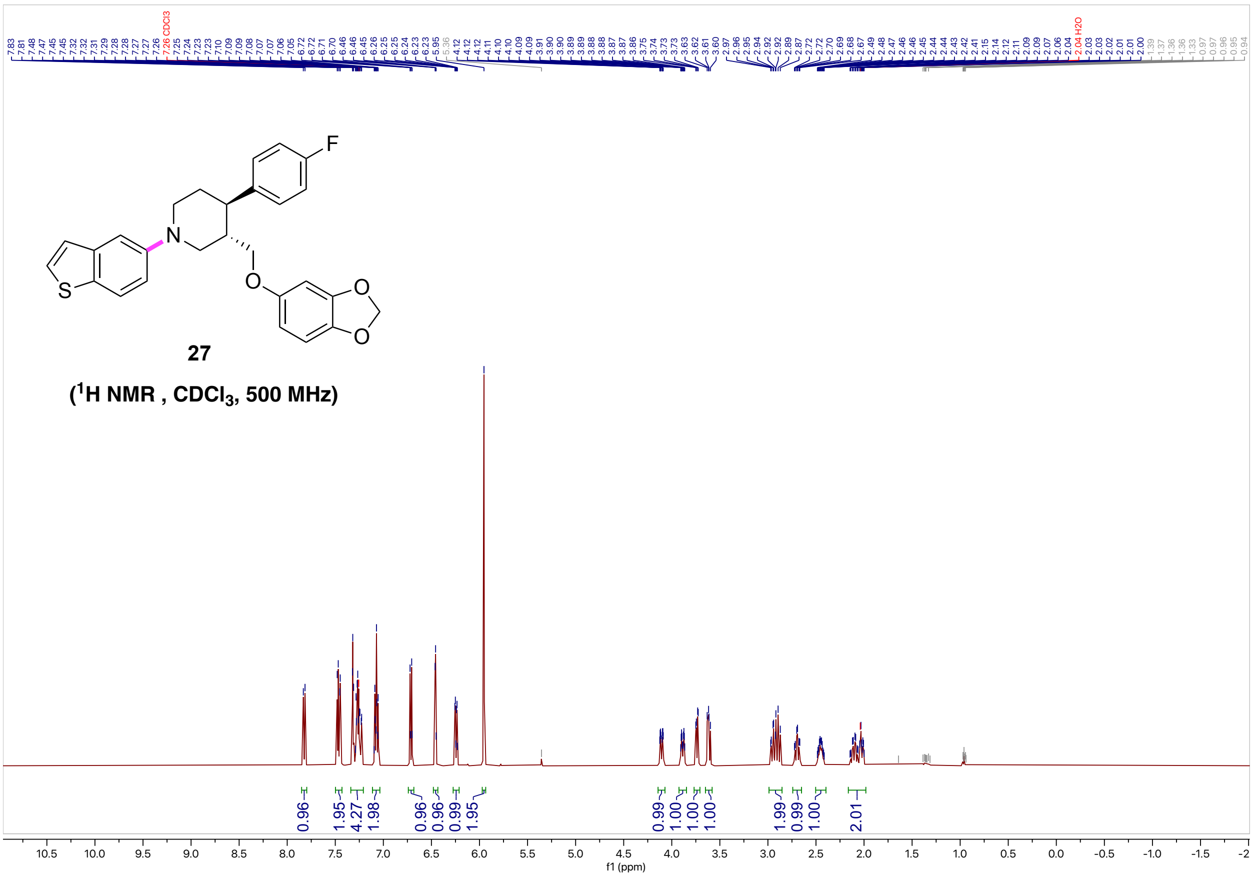
27
(^{1}H NMR , $CDCl_3$, 500 MHz)

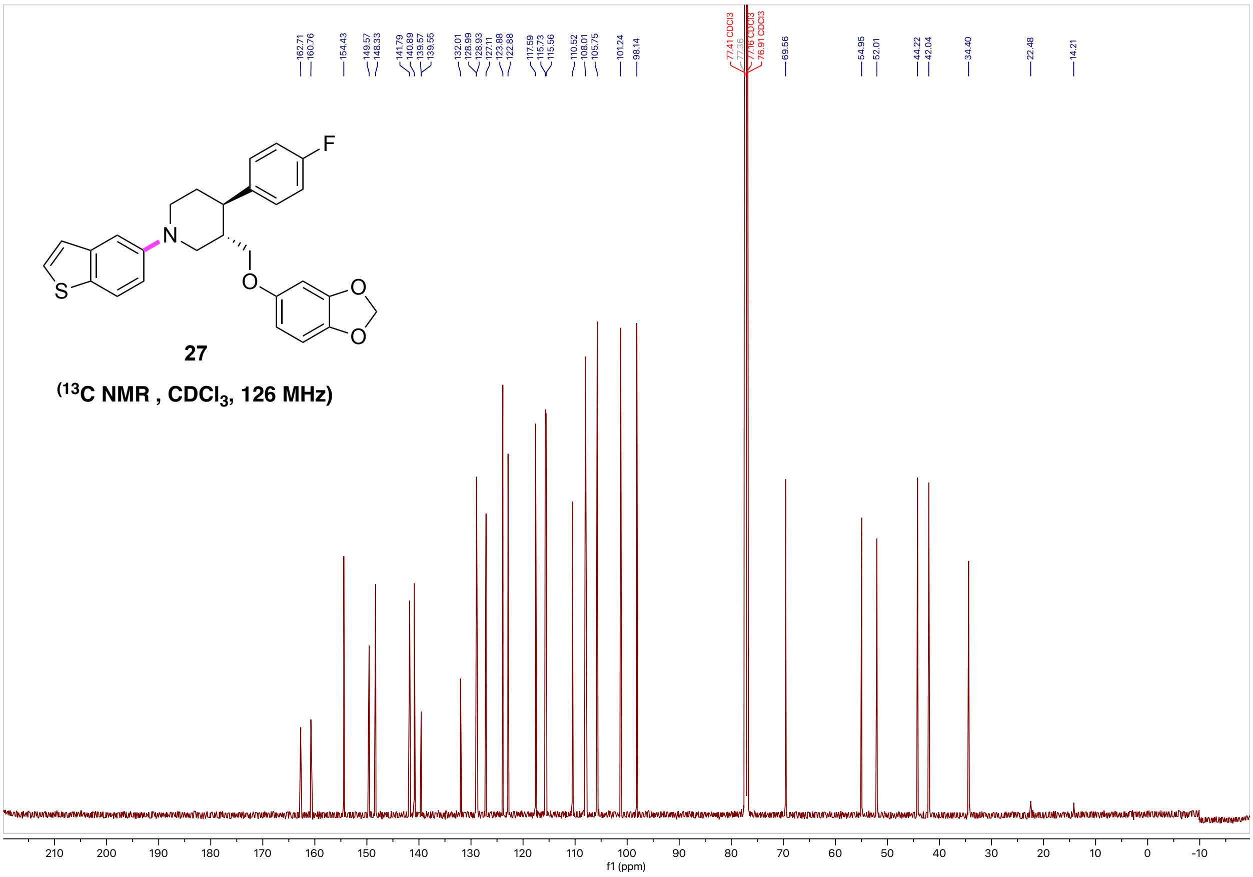
27
(^{13}C NMR , $CDCl_3$, 126 MHz)

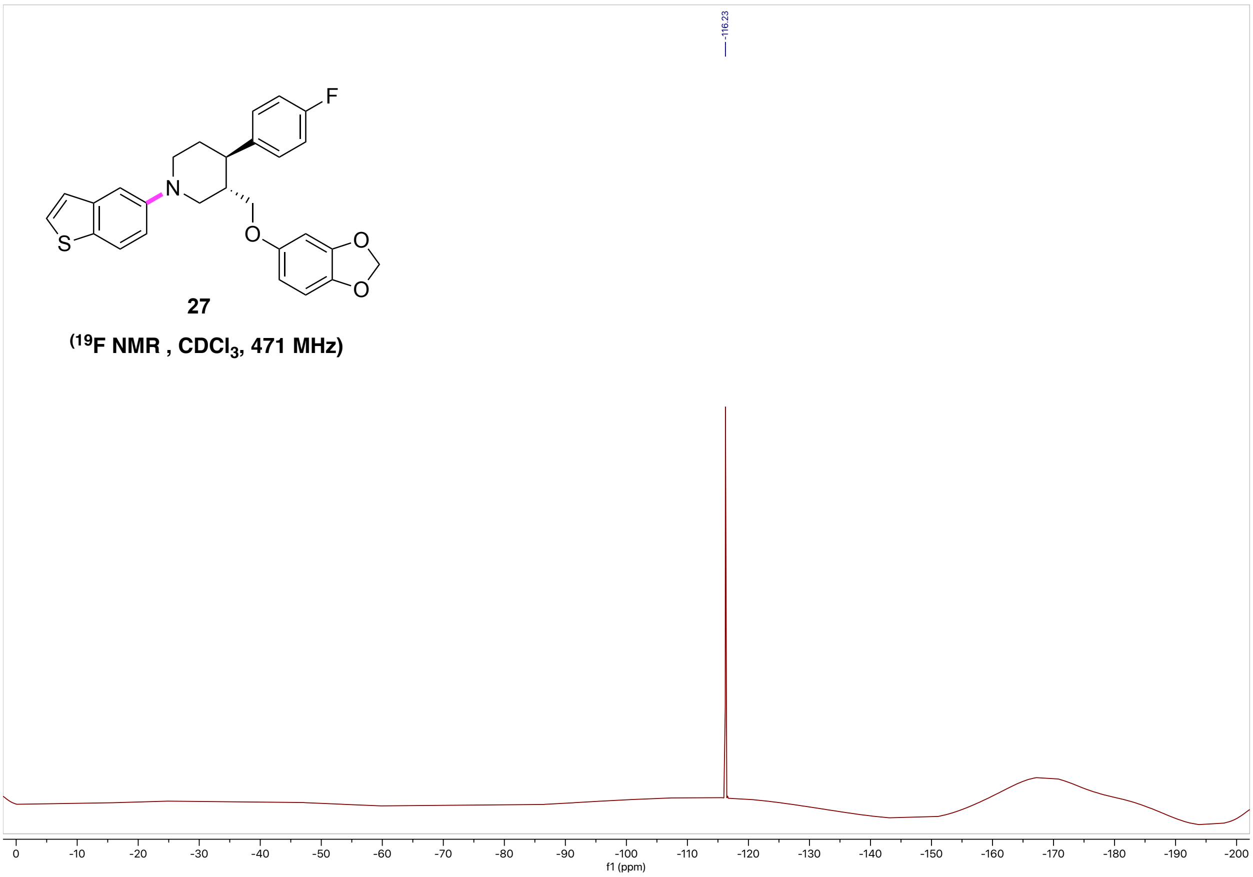

-116.23
F
N
S
O
O
O
27
(19F NMR , CDCl3, 471 MHz)
0
-10
-20
-30
-40
-50
-60
-70
-80
-90
-100
-110
-120
-130
-140
-150
-160
-170
-180
-190
-200
f1 (ppm)

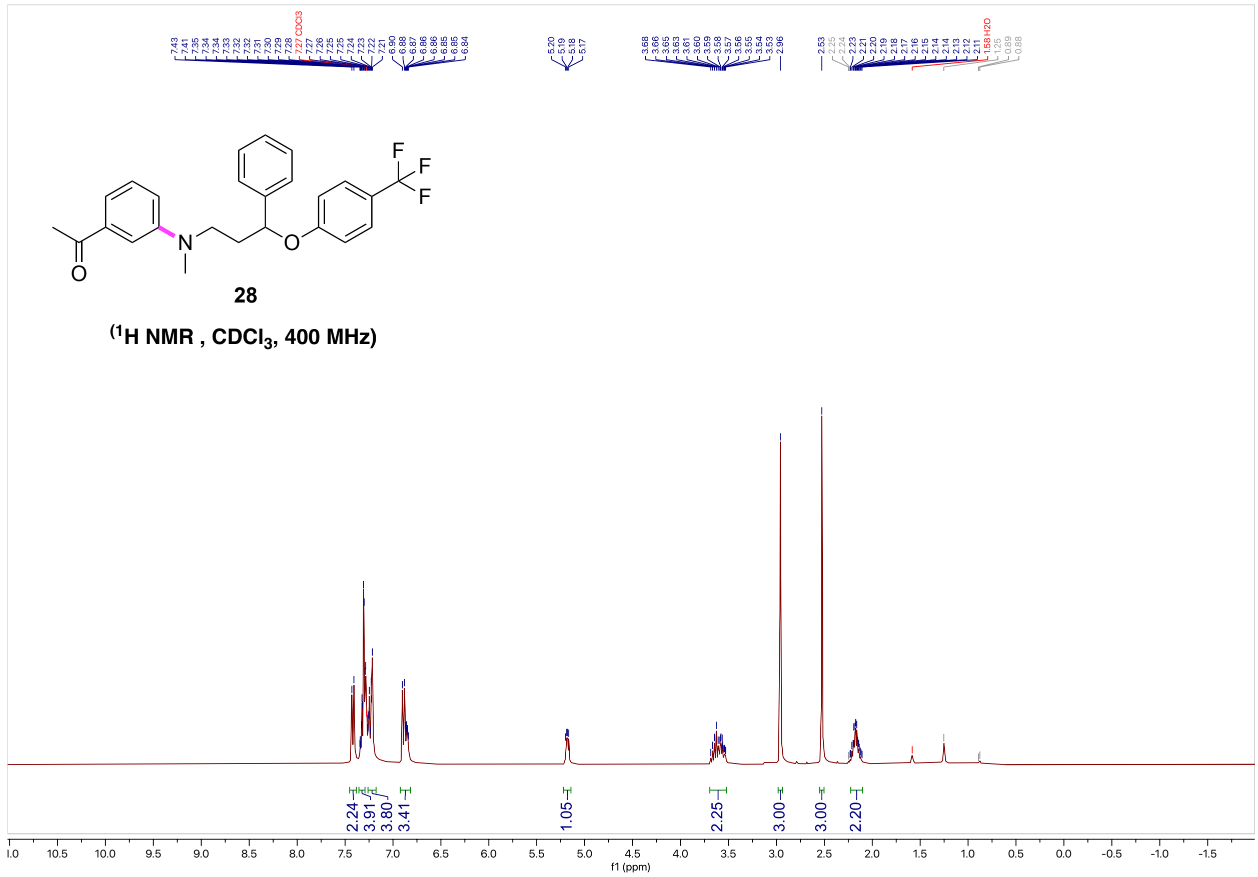

28
(1H NMR , CDCl3, 400 MHz)
f1 (ppm)

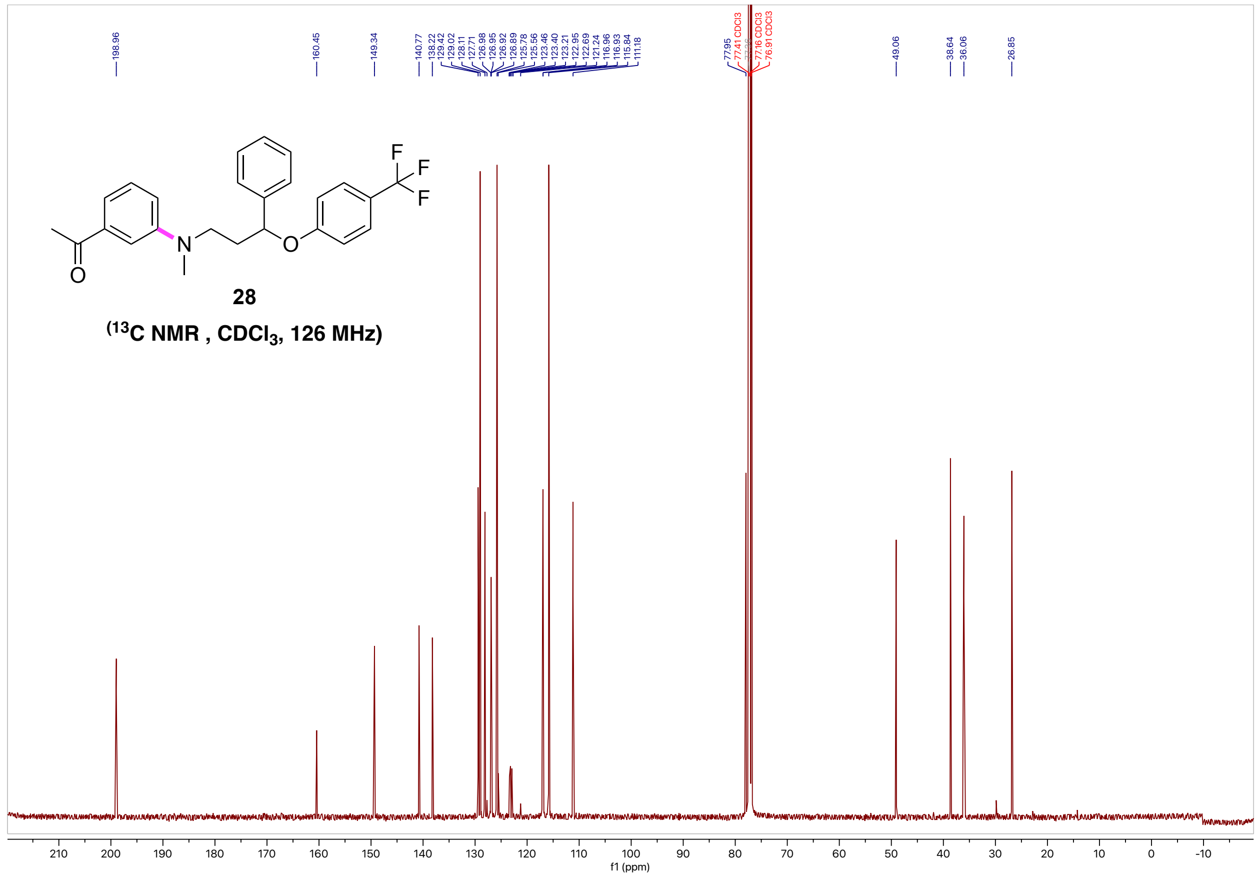

28
(13C NMR , CDCl3, 126 MHz)
f1 (ppm)

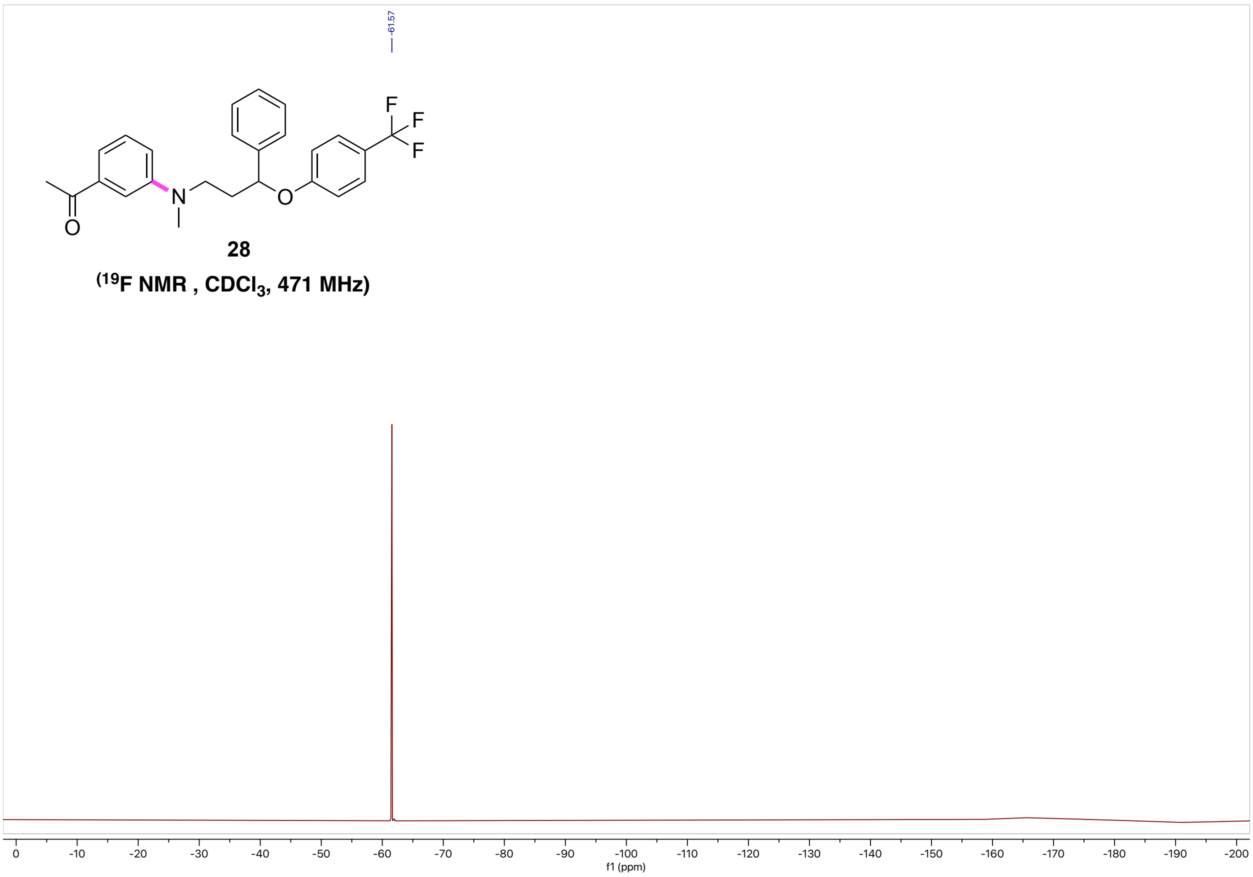
-61.57
F
F
F
N
O
O
28
(19F NMR , CDCl3, 471 MHz)
0 -10 -20 -30 -40 -50 -60 -70 -80 -90 -100 -110 -120 -130 -140 -150 -160 -170 -180 -190 -200
f1 (ppm)

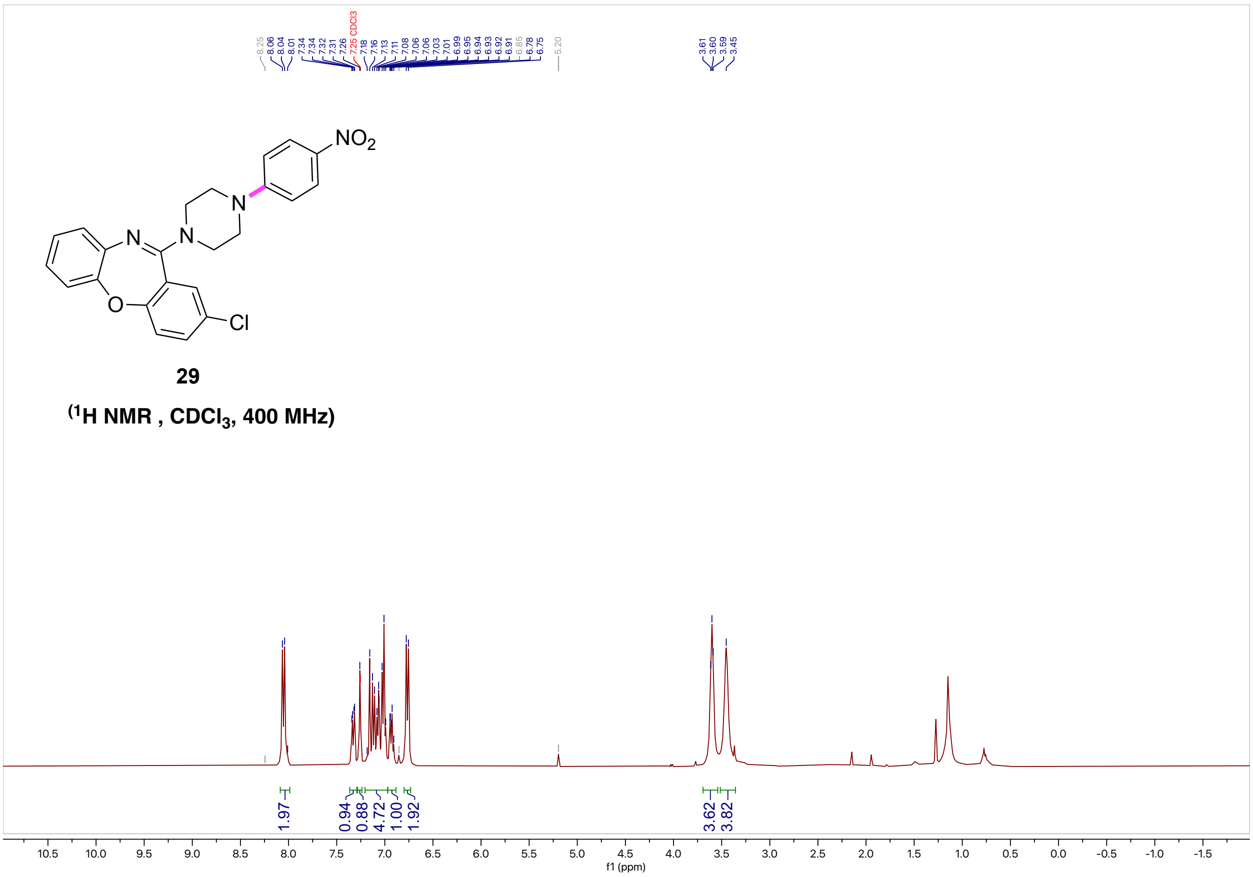
8.25
8.06
8.04
8.01
7.34
7.34
7.32
7.31
7.26
7.25 CDCl3
7.18
7.16
7.13
7.11
7.08
7.06
7.06
7.03
7.01
6.99
6.95
6.94
6.93
6.92
6.91
6.85
6.78
6.75
5.20
3.61
3.60
3.59
3.45
NO_2
N
N
N
O
Cl
29
(^{1}H NMR , $CDCl_3$, 400 MHz)
1.97
0.94
0.88
4.72
1.00
1.92
3.62
3.82
10.5
10.0
9.5
9.0
8.5
8.0
7.5
7.0
6.5
6.0
5.5
5.0
4.5
4.0
3.5
3.0
2.5
2.0
1.5
1.0
0.5
0.0
-0.5
-1.0
-1.5
f1 (ppm)

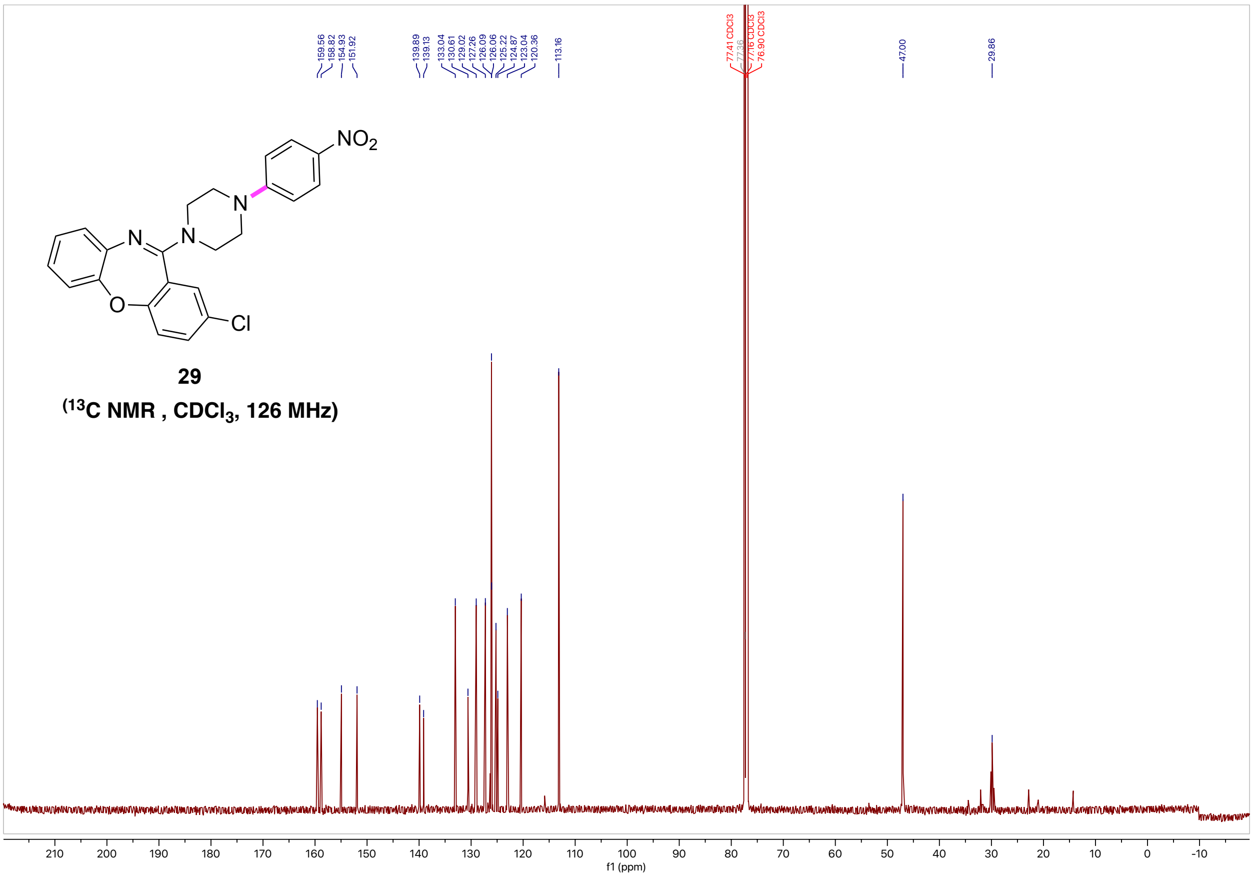
159.56
158.82
154.93
151.92
139.89
139.13
133.04
130.61
129.02
127.26
126.09
126.06
125.22
124.87
123.04
120.36
113.16
77.41 CDCl3
77.36
77.16 CDCl3
76.90 CDCl3
47.00
29.86
NO_2
N
N
N
O
Cl
29
(^{13}C NMR , $CDCl_3$, 126 MHz)
210
200
190
180
170
160
150
140
130
120
110
100
90
80
70
60
50
40
30
20
10
0
-10
f1 (ppm)

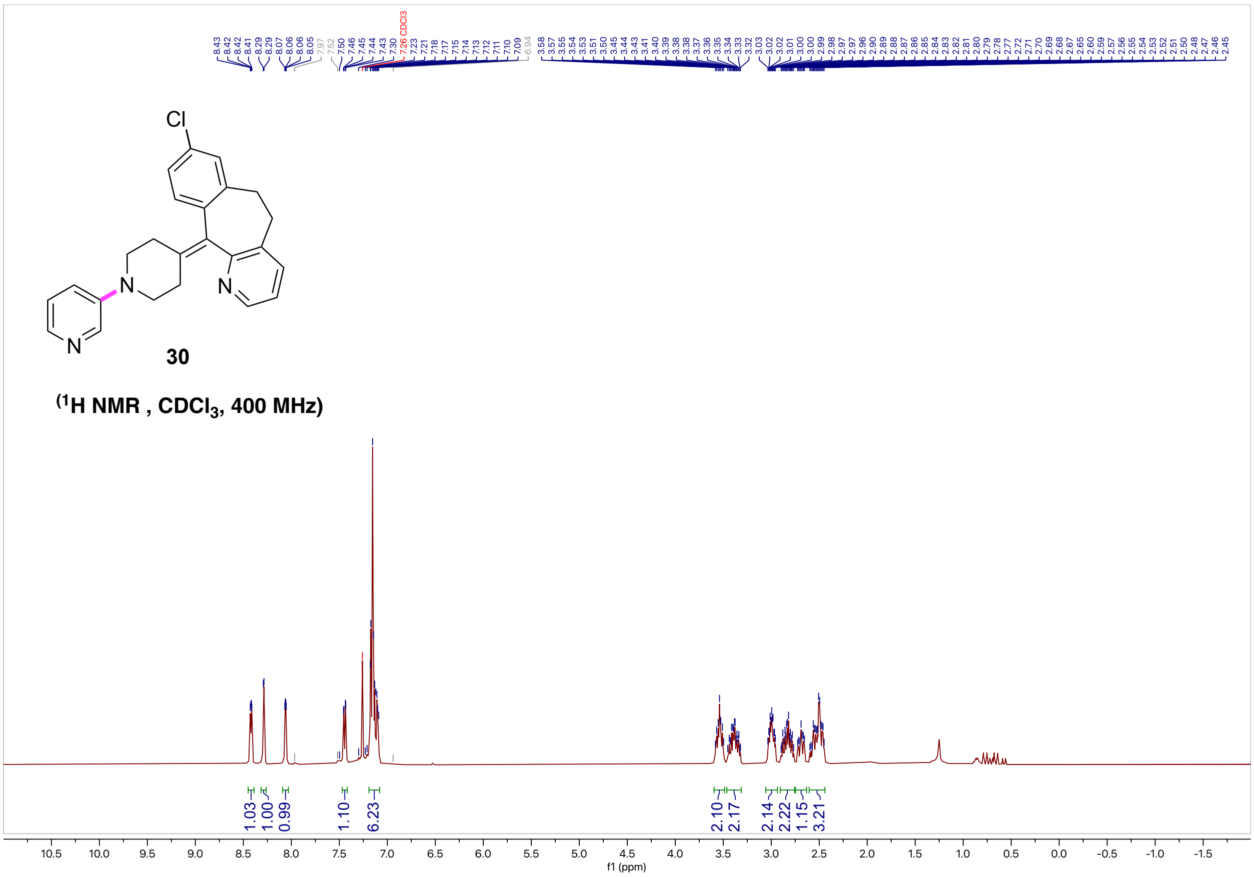
Cl
N
N
N
30
(1H NMR , CDCl3, 400 MHz)
f1 (ppm)

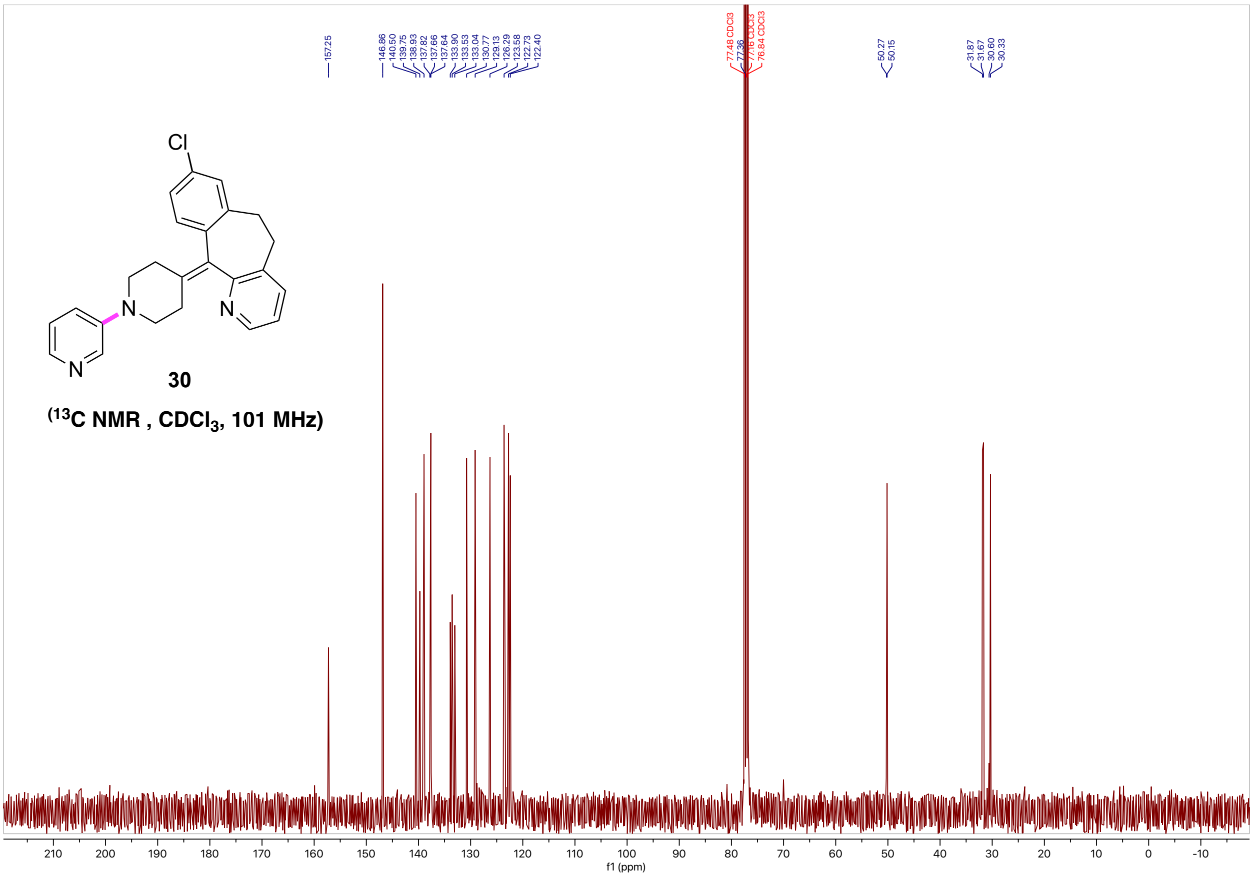
Cl
N
N
N
30
(13C NMR , CDCl3, 101 MHz)
f1 (ppm)

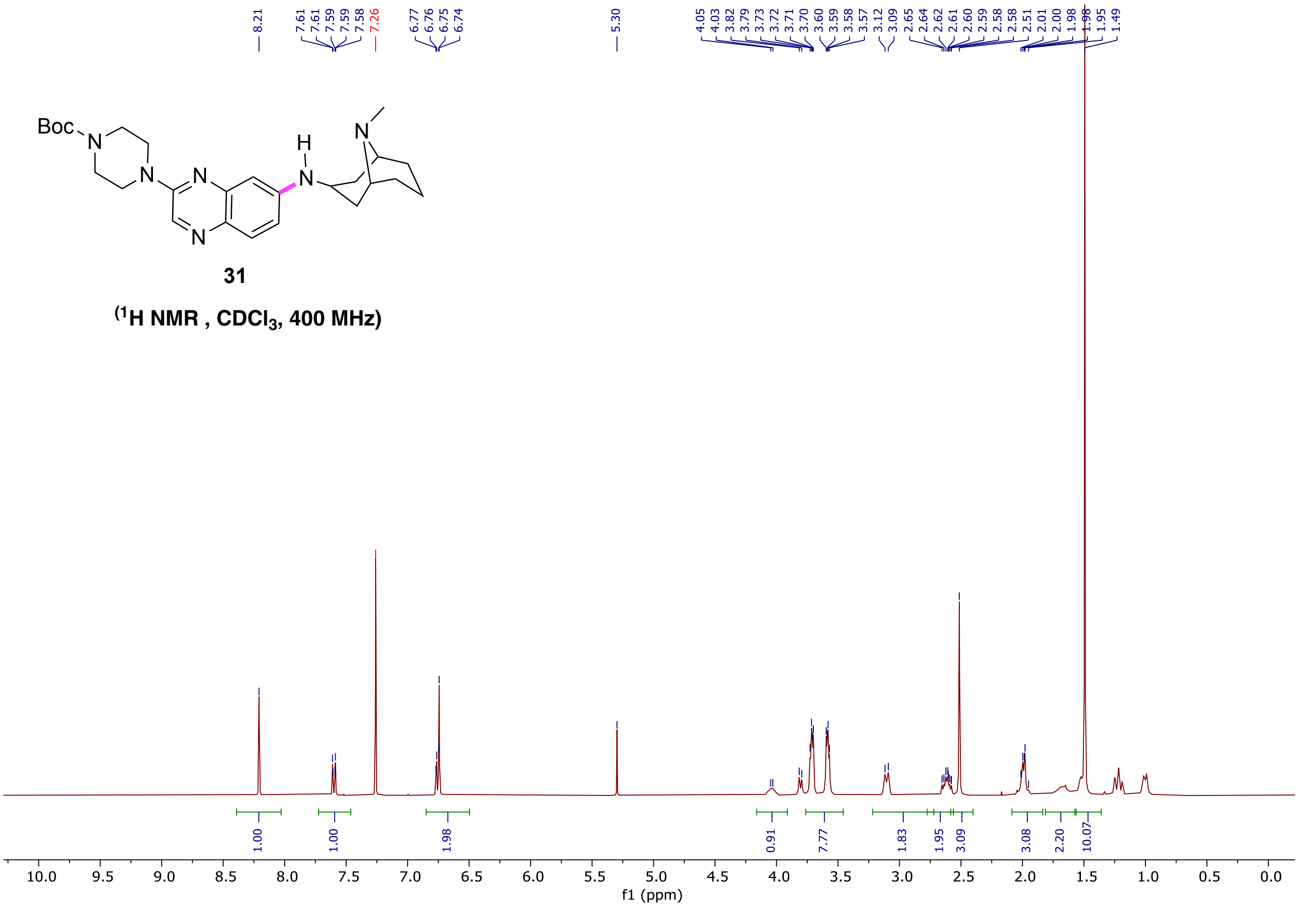
Boc
N
N
N
N
H
N
N
31
(1H NMR , CDCl3, 400 MHz)
f1 (ppm)

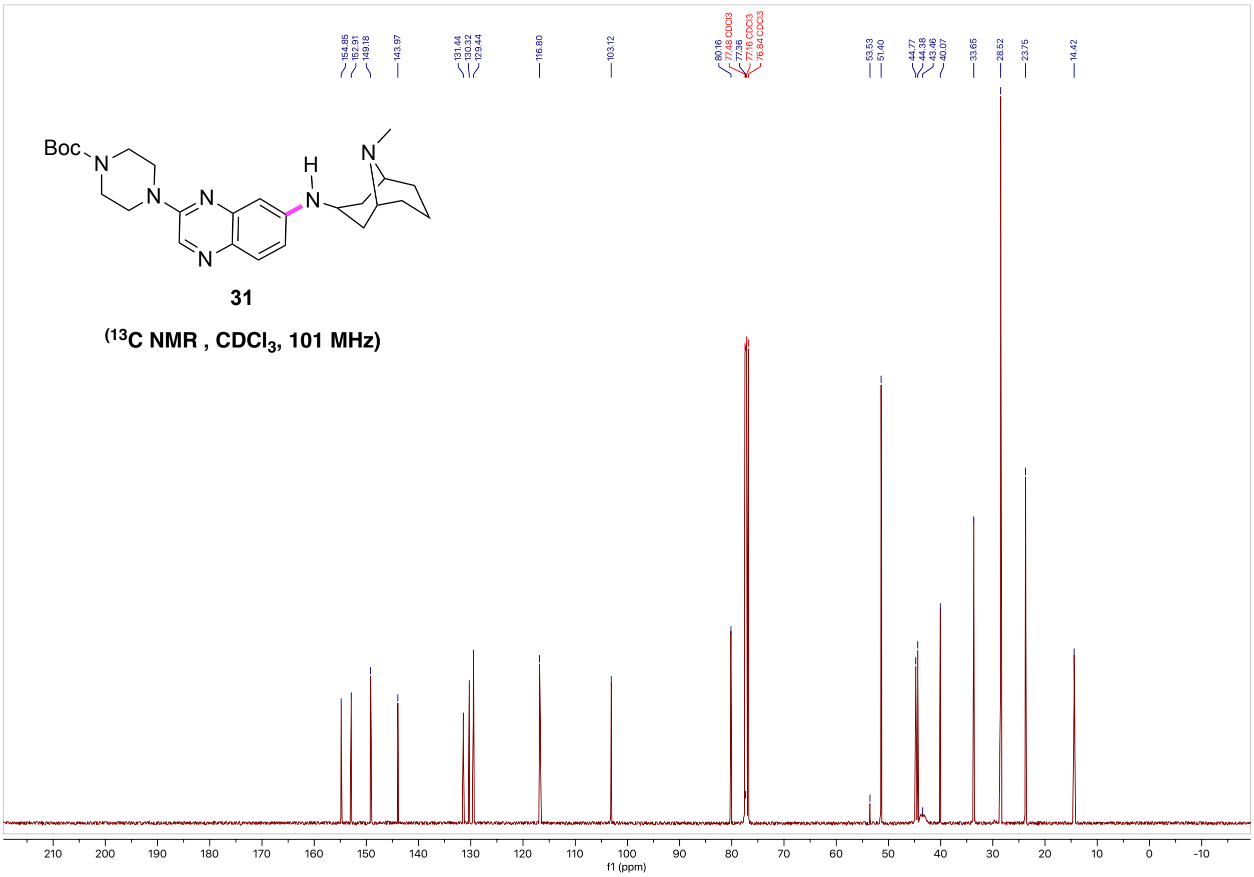
Boc
N
N
N
N
H
N
N
31
(13C NMR , CDCl3, 101 MHz)
f1 (ppm)

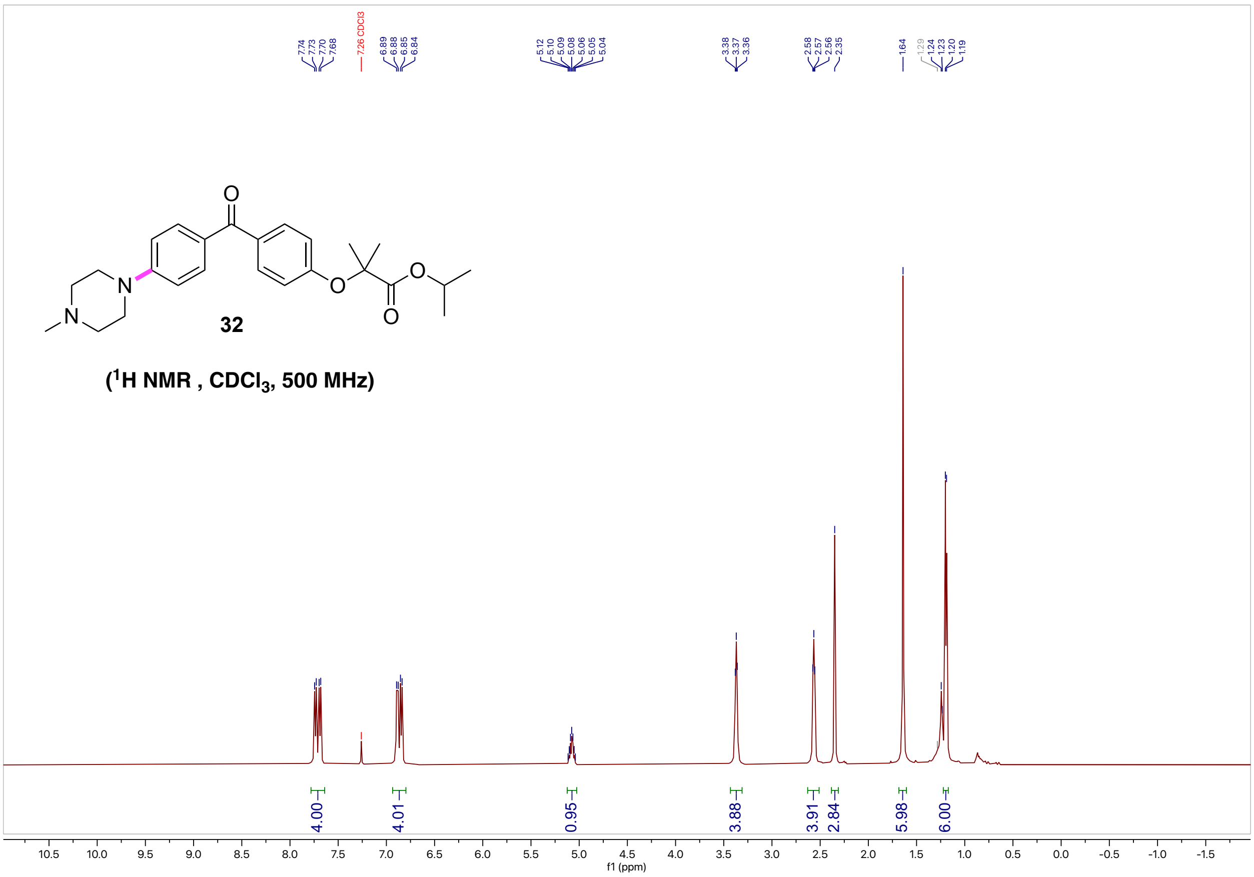
32
(¹H NMR , CDCl₃, 500 MHz)
f1 (ppm)

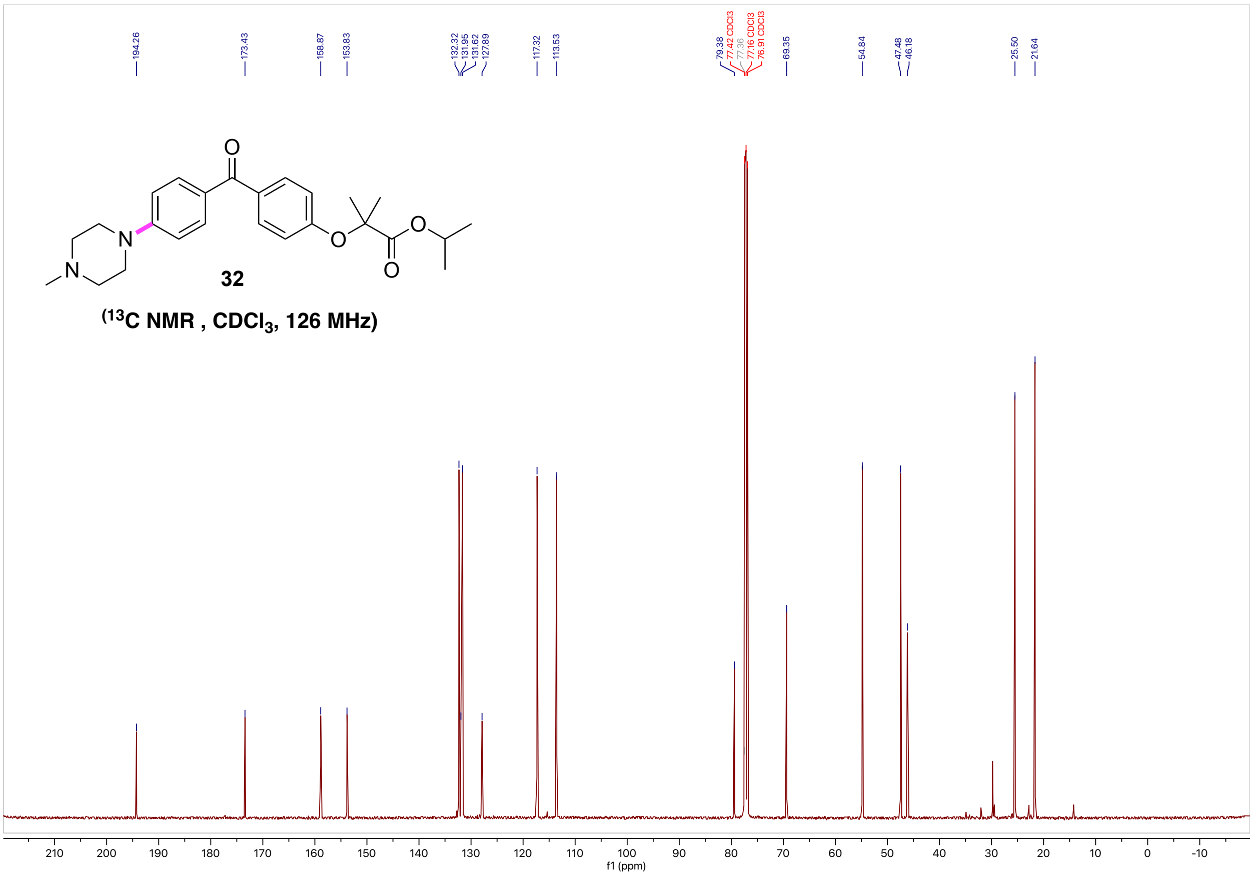
32
(¹³C NMR , CDCl₃, 126 MHz)
f1 (ppm)

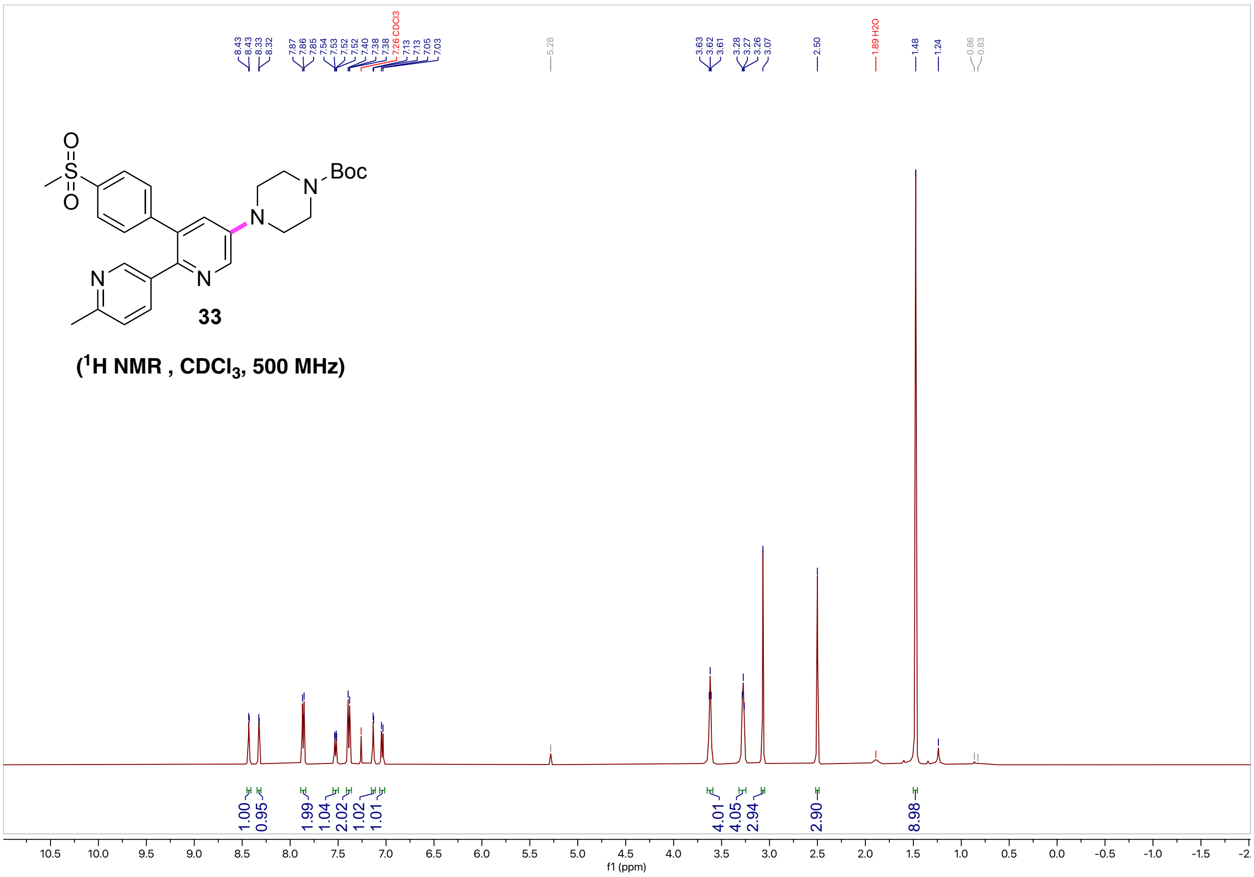
Boc
33
(1H NMR , CDCl3, 500 MHz)
f1 (ppm)

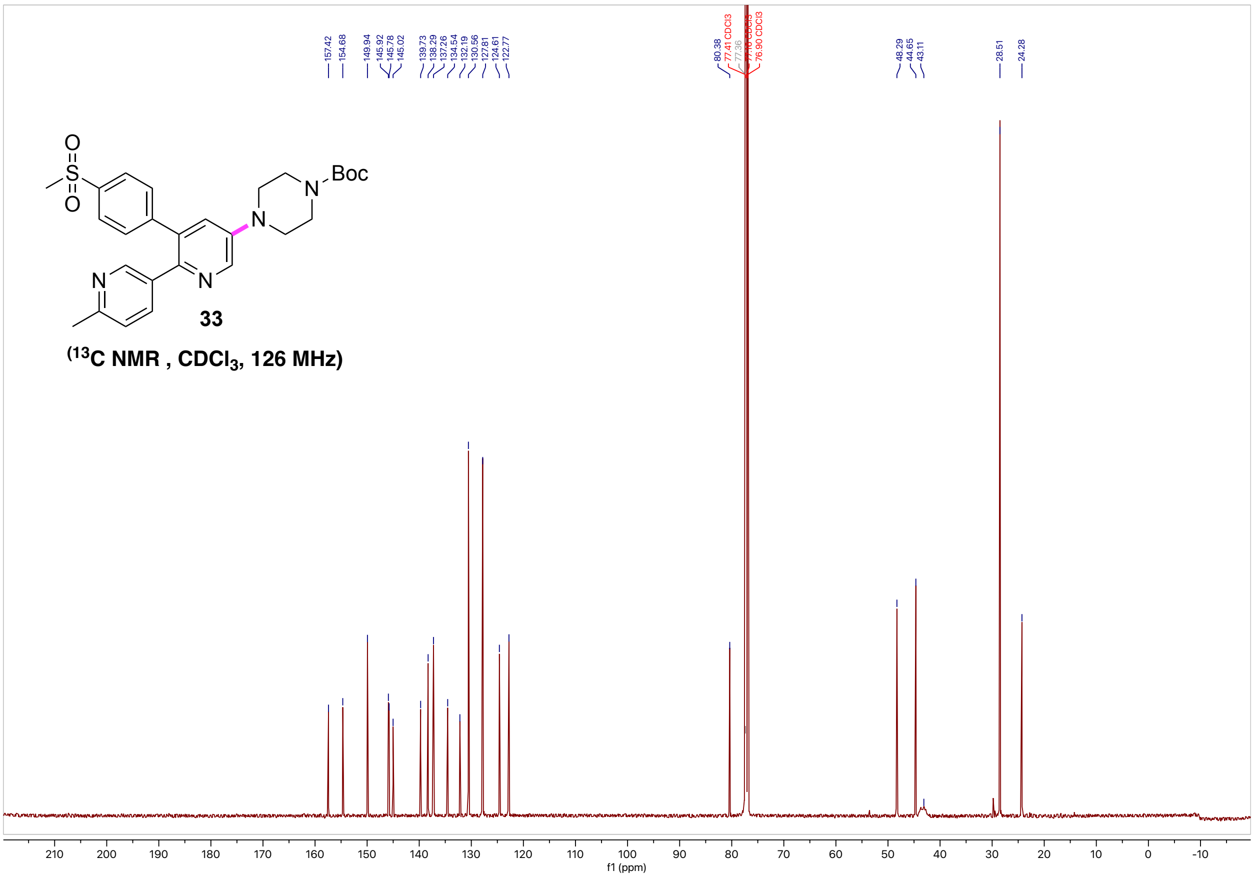
Boc
33
(13C NMR , CDCl3, 126 MHz)
f1 (ppm)

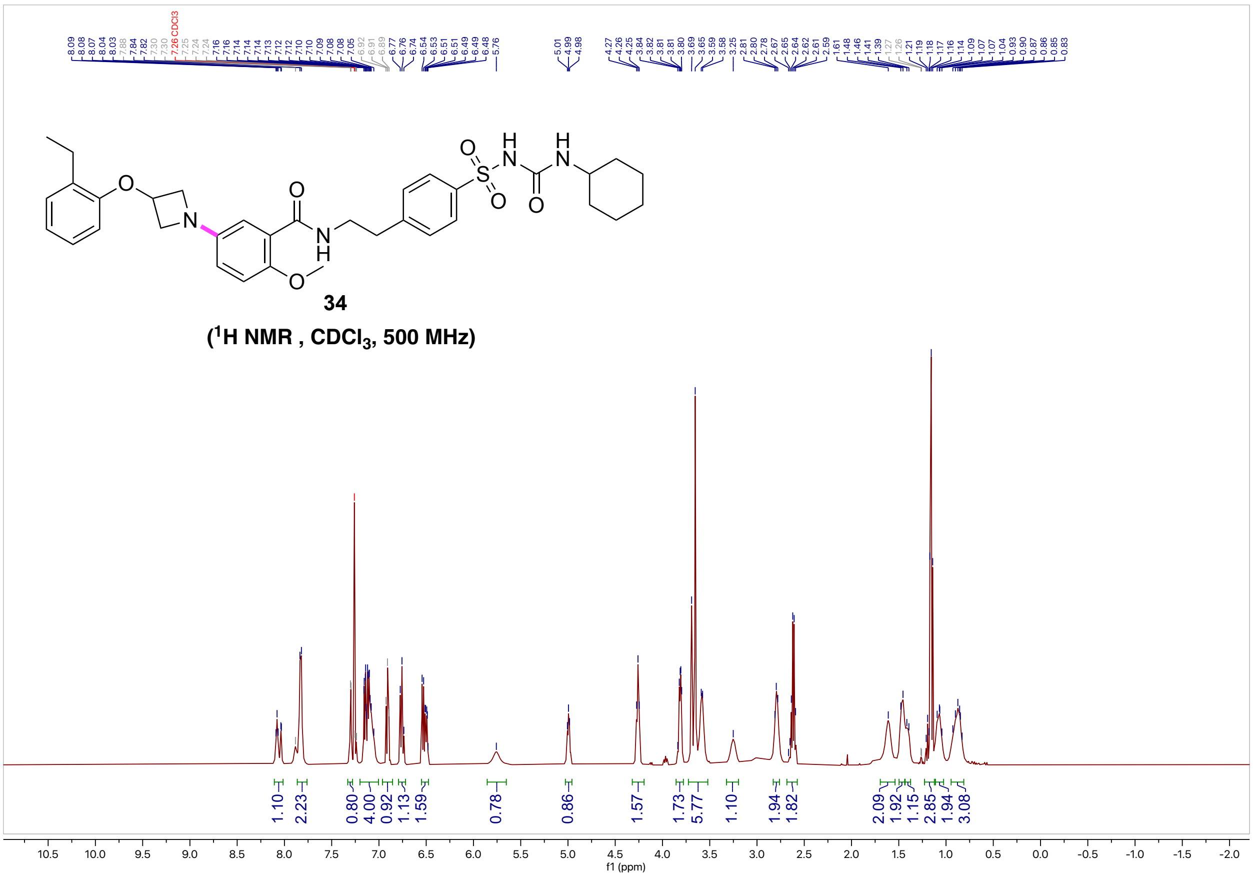

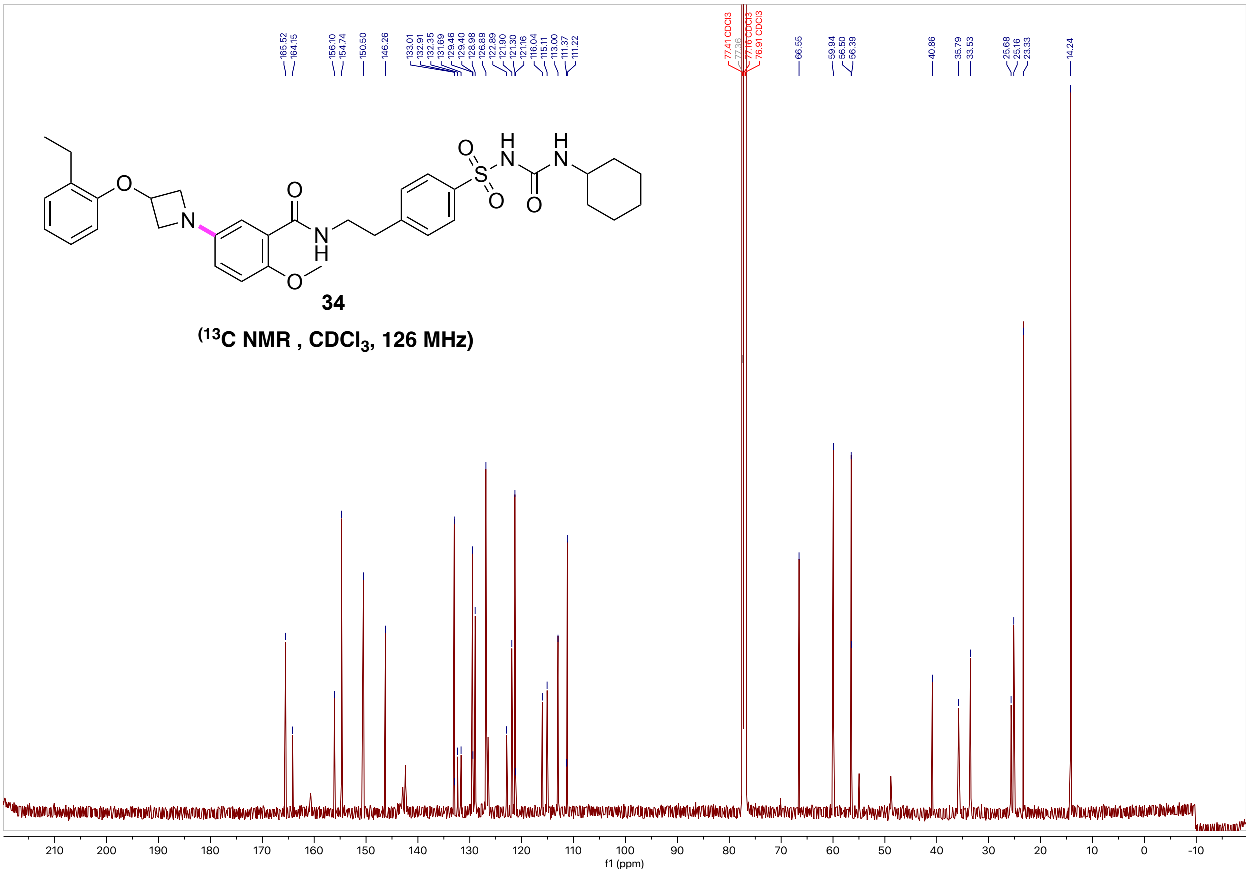

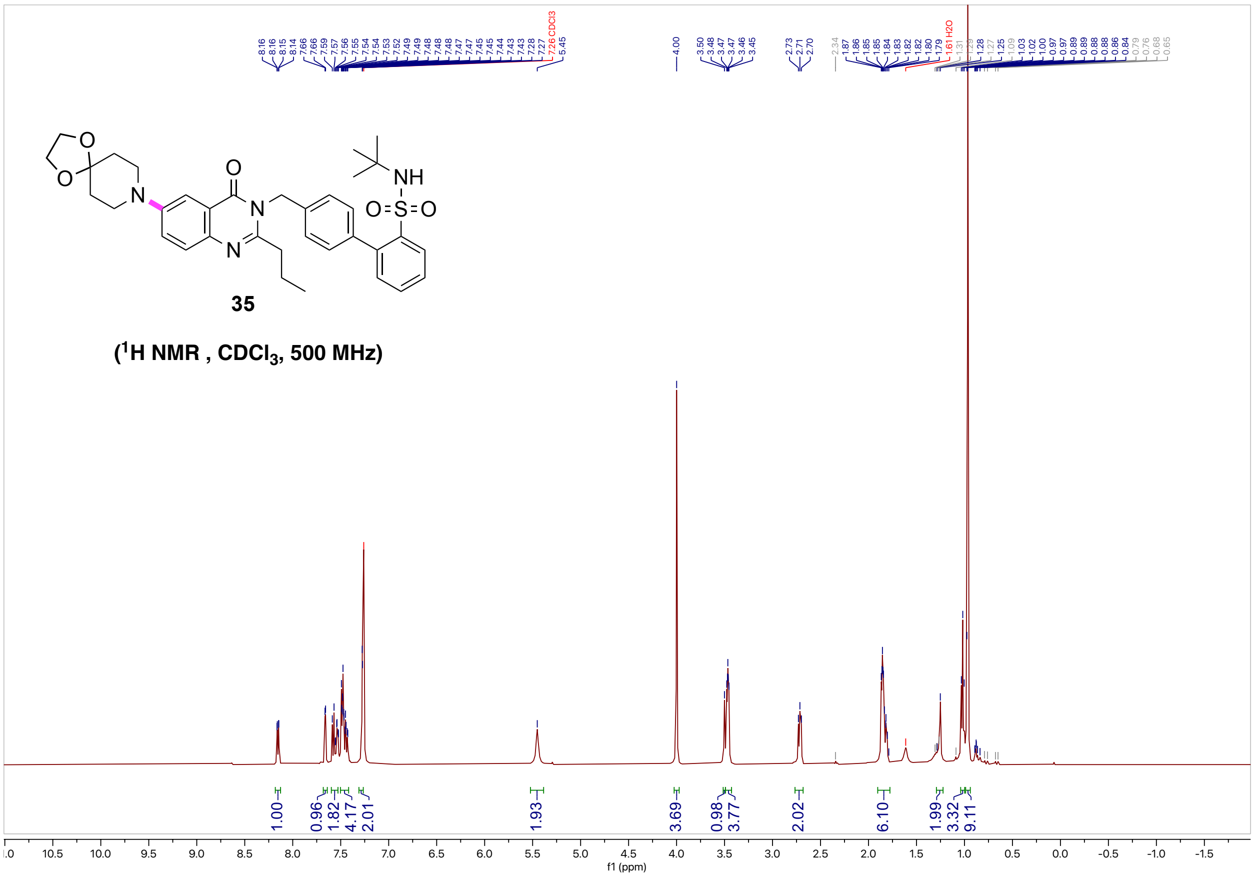

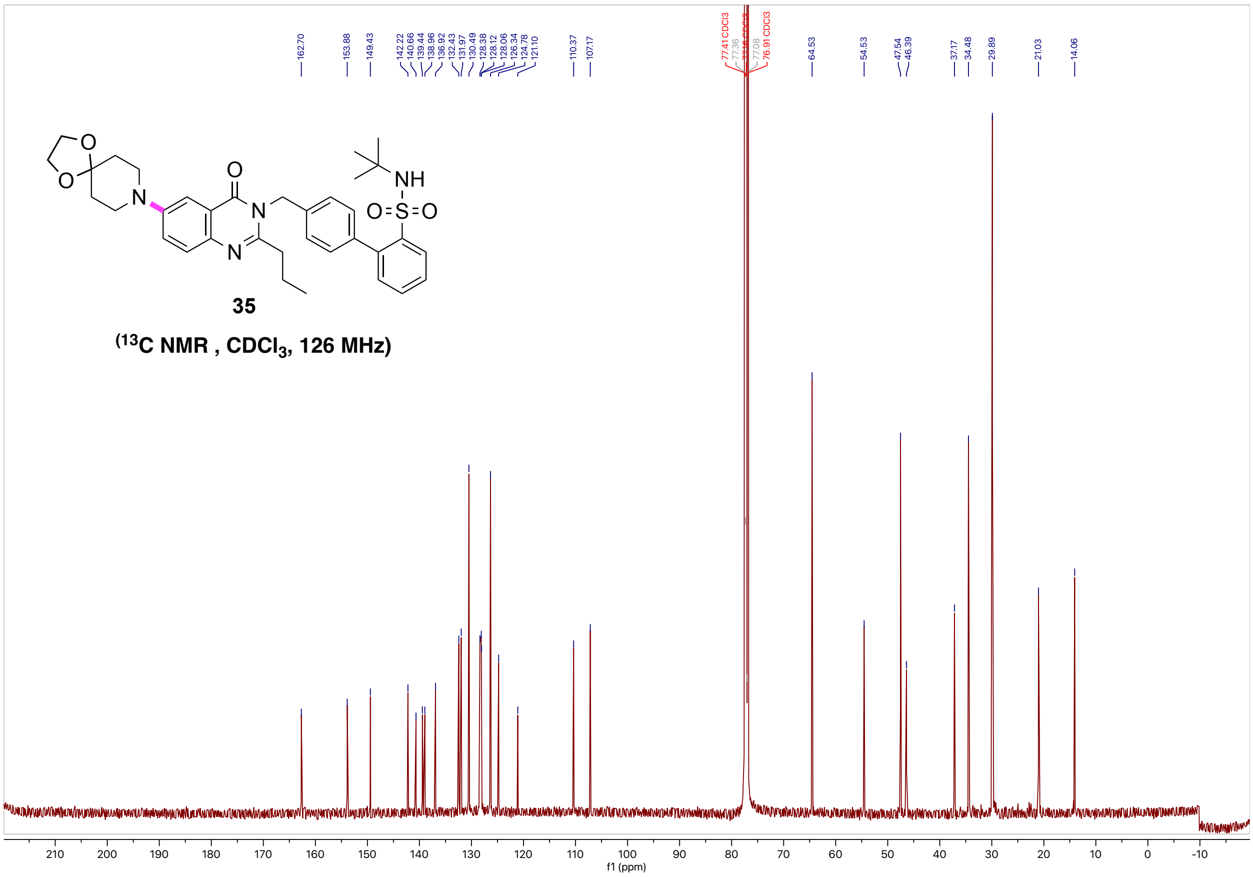

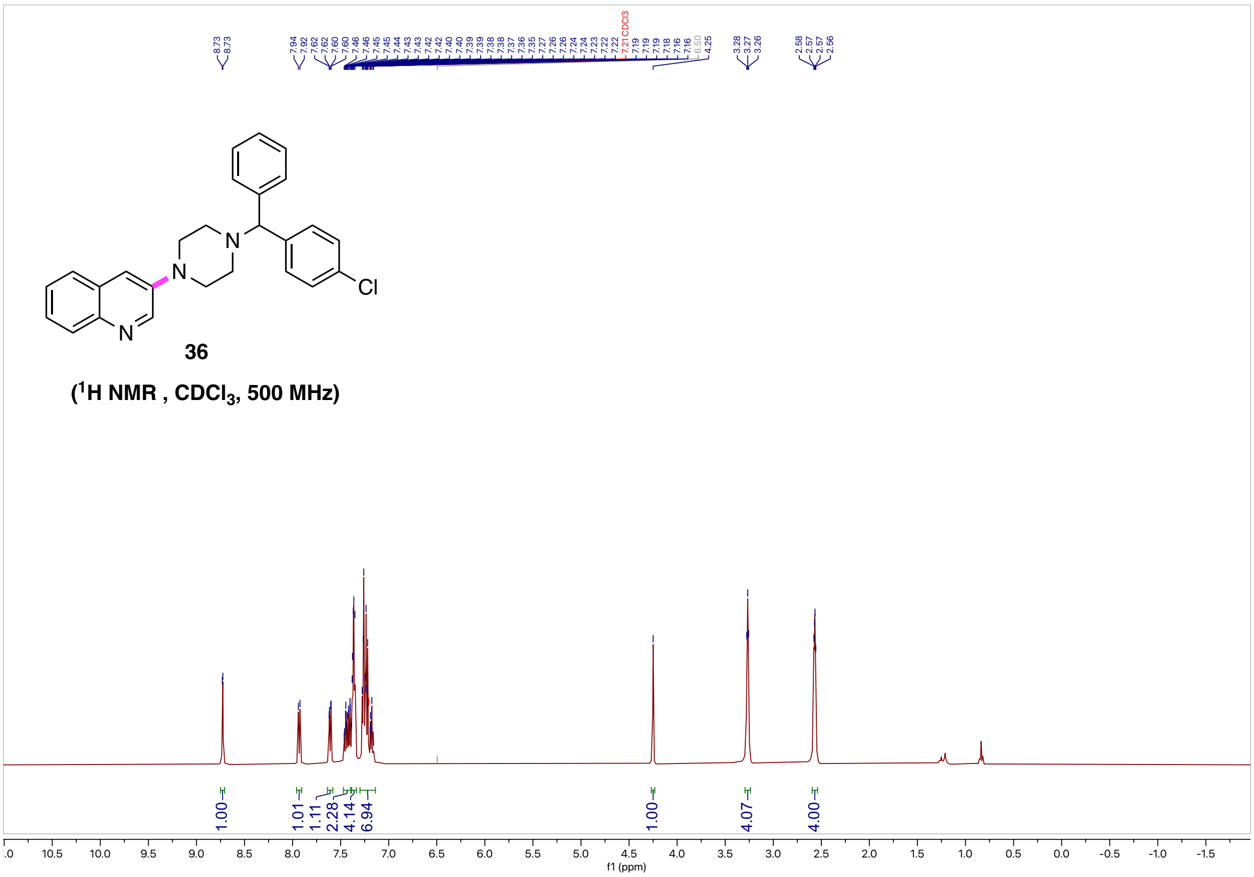
36
(1H NMR , CDCl3, 500 MHz)
f1 (ppm)

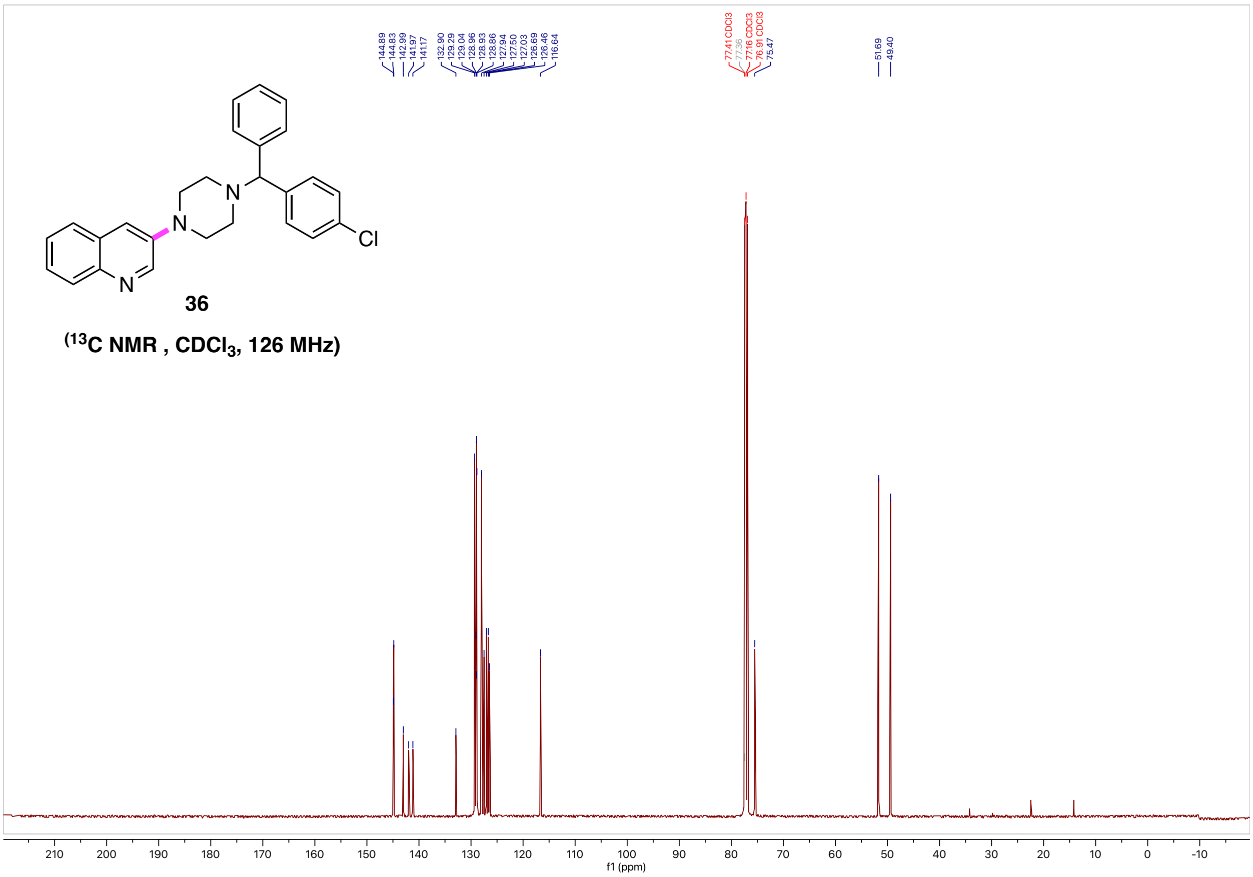
36
(13C NMR , CDCl3, 126 MHz)
f1 (ppm)

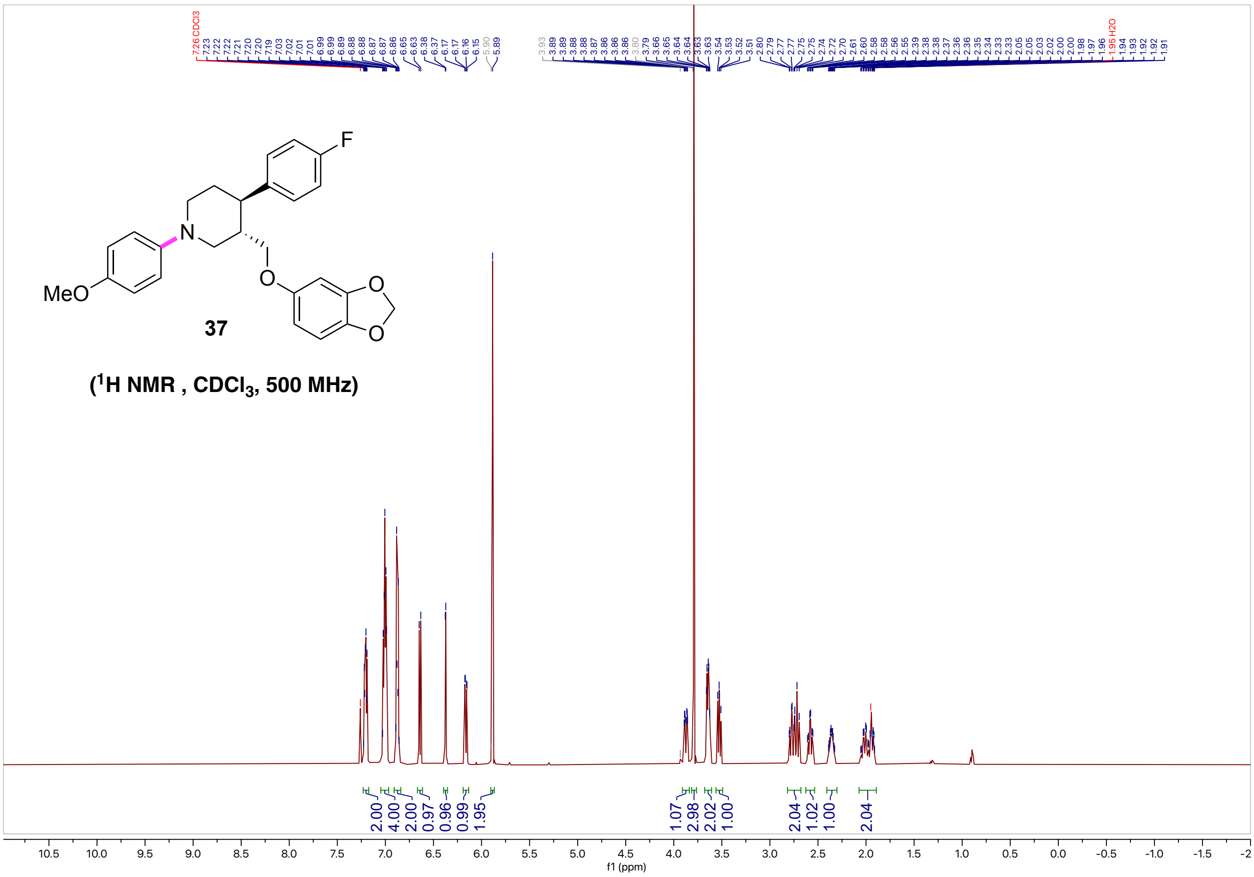

MeO
N
F
O
O
O
37
(¹H NMR , CDCl3, 500 MHz)
f1 (ppm)

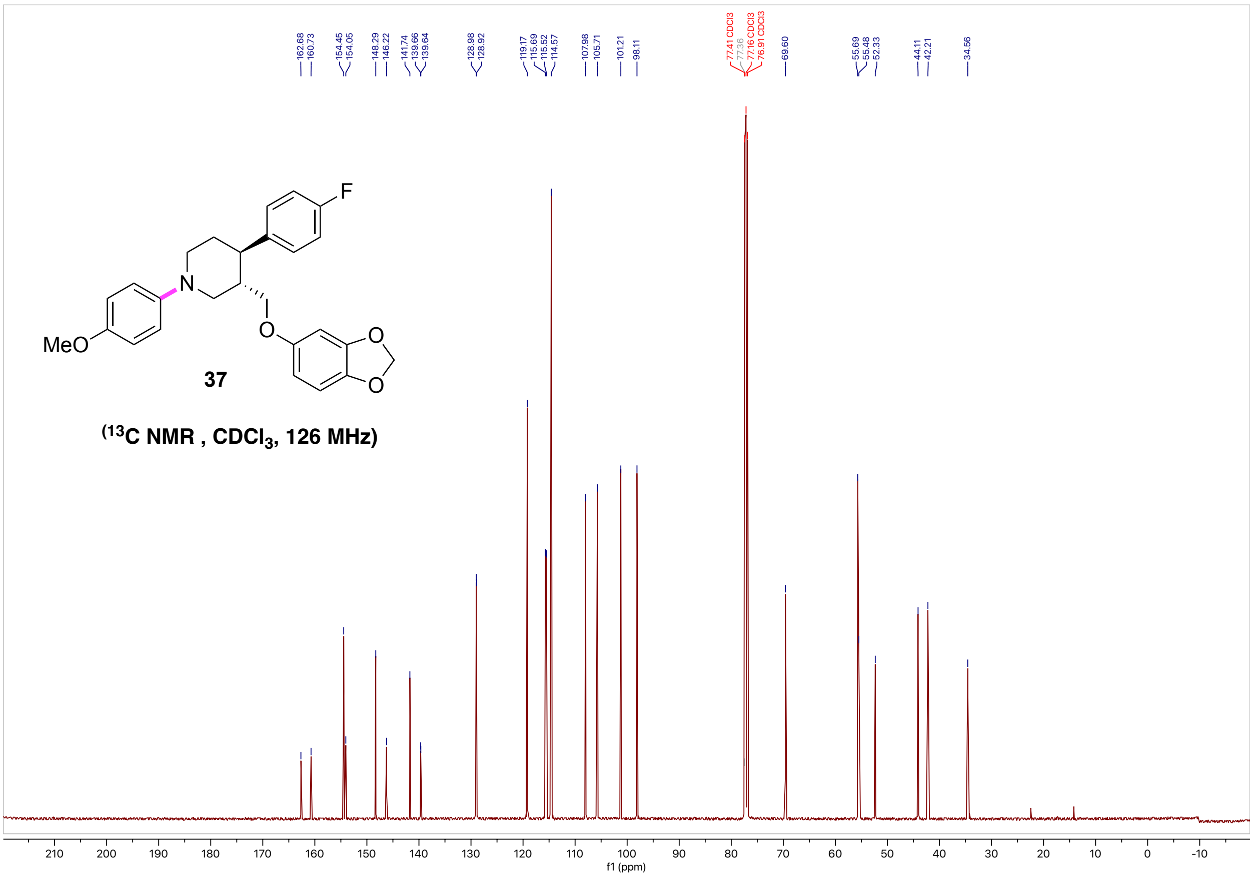

MeO
N
F
O
O
O
37
(¹³C NMR , CDCl3, 126 MHz)
f1 (ppm)

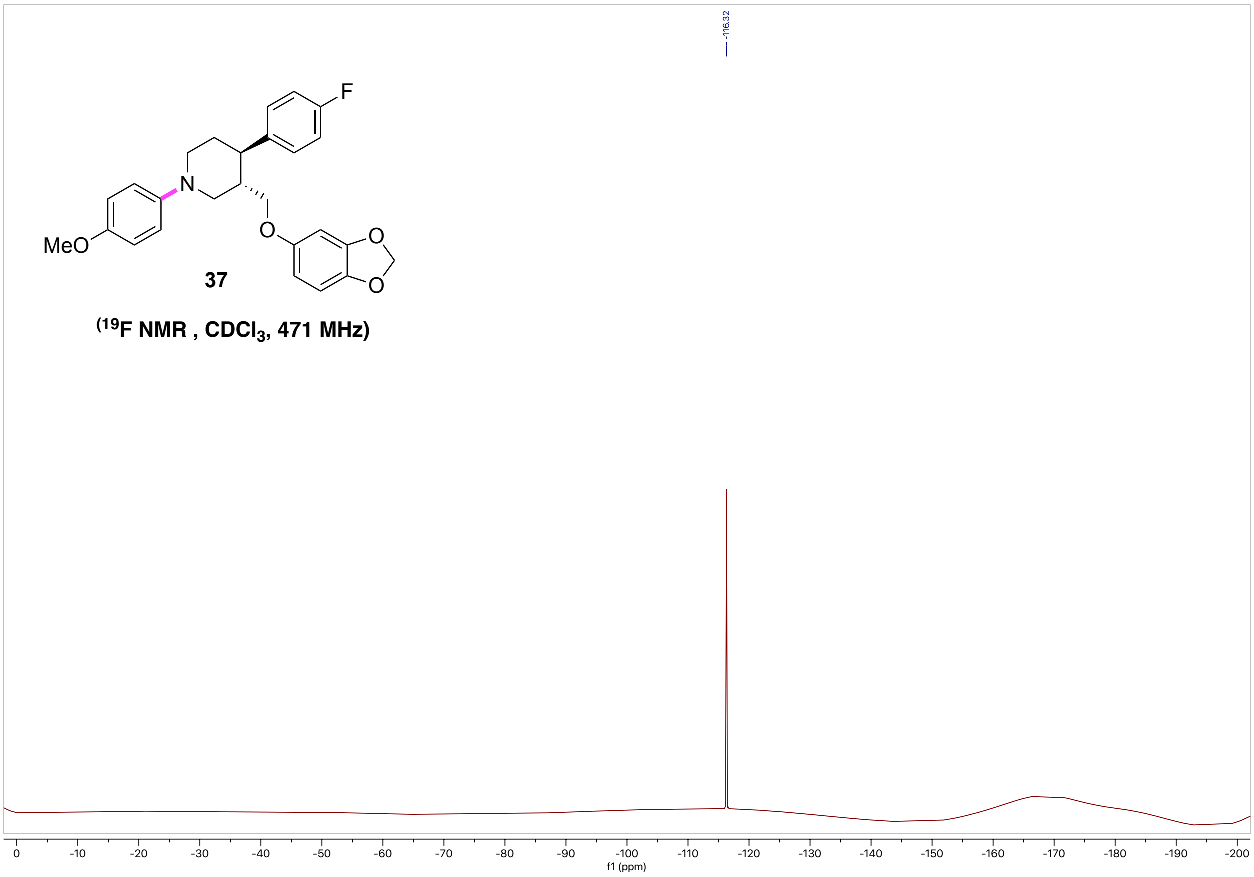
-116.32
MeO
N
F
O
O
O
37
(^{19}F NMR , CDCl$_3$, 471 MHz)
0
-10
-20
-30
-40
-50
-60
-70
-80
-90
-100
-110
-120
-130
-140
-150
-160
-170
-180
-190
-200
f1 (ppm)

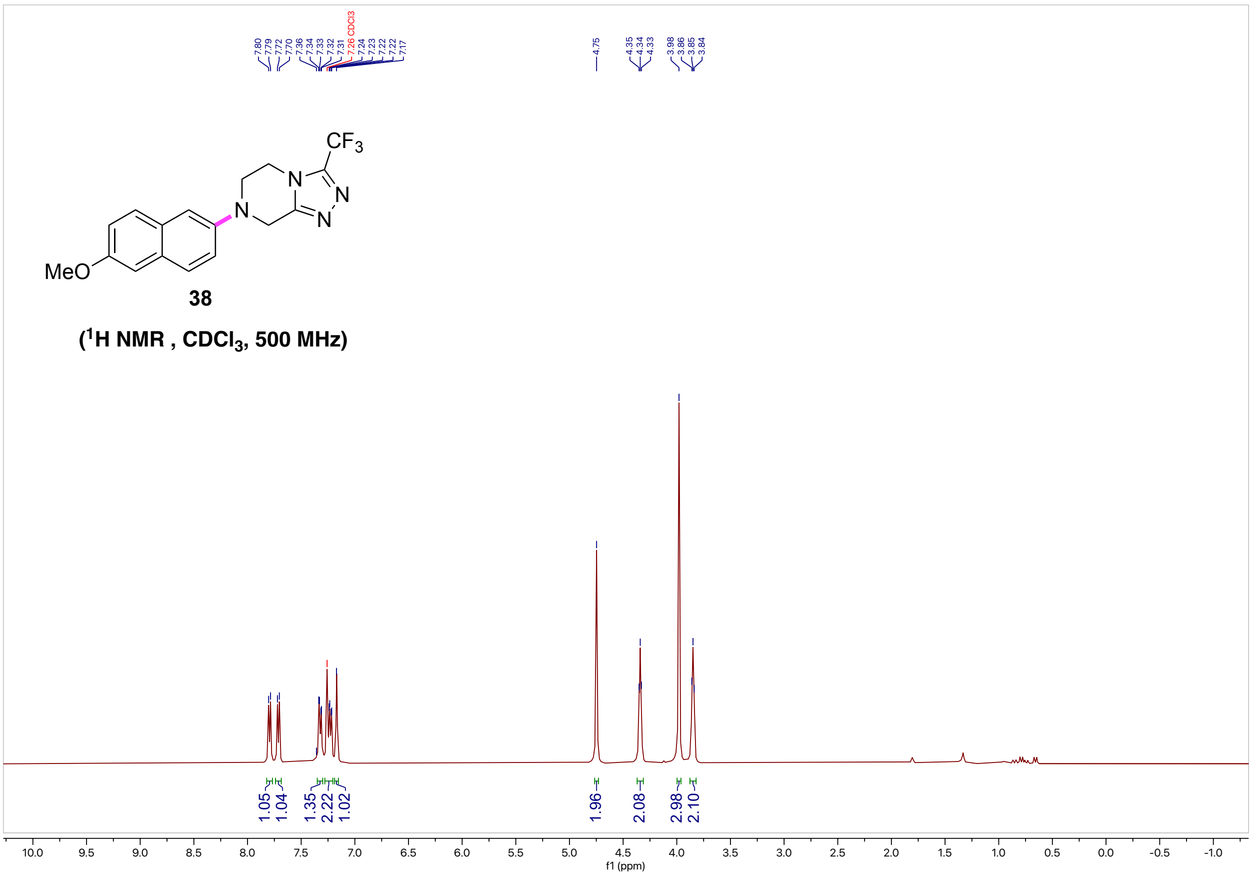
7.80
7.79
7.72
7.70
7.36
7.34
7.33
7.32
7.31
7.26 CDCl3
7.24
7.23
7.22
7.22
7.17
4.75
4.35
4.34
4.33
3.98
3.86
3.85
3.84
CF3
N
N
N
N
N
MeO
38
(1H NMR , CDCl3, 500 MHz)
1.05
1.04
1.35
2.22
1.02
1.96
2.08
2.98
2.10
f1 (ppm)

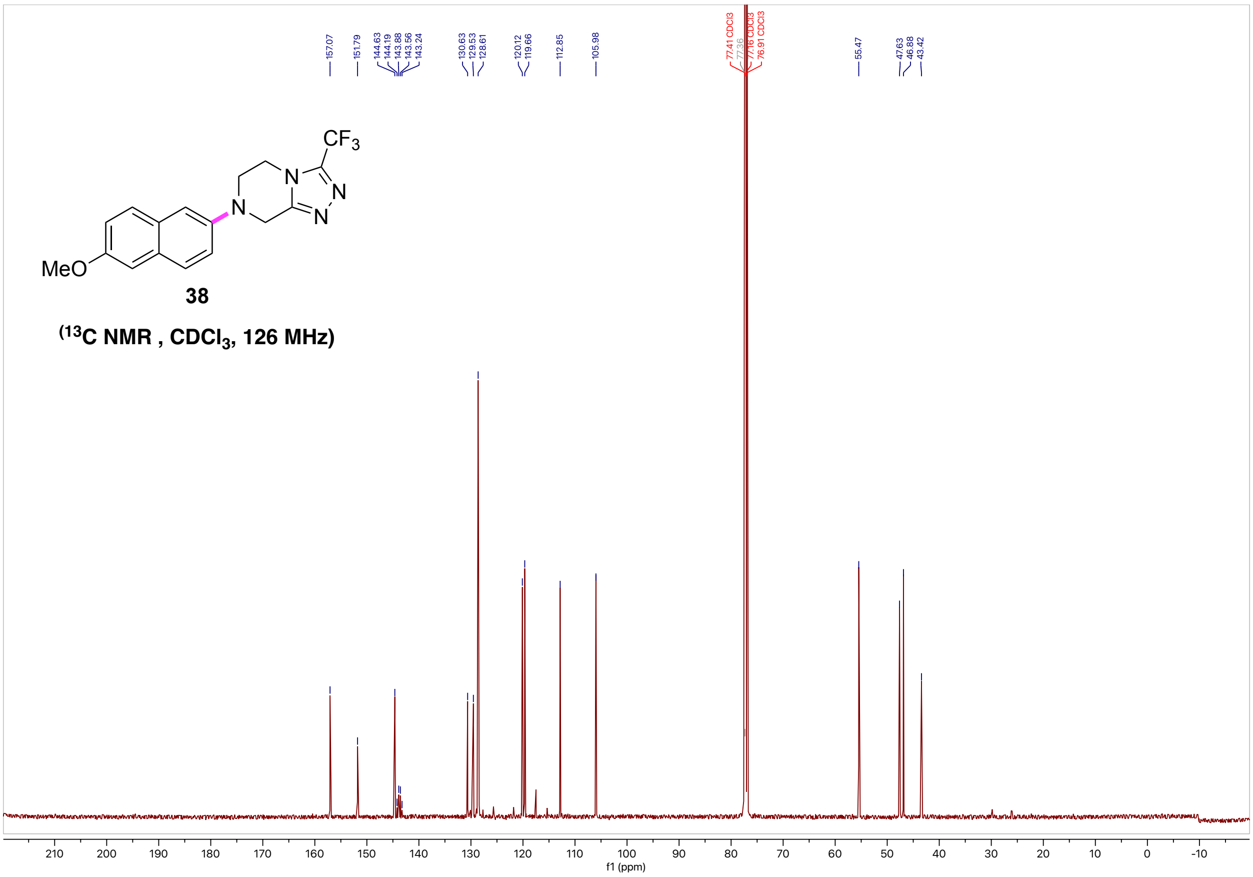
157.07
151.79
144.63
144.19
143.88
143.56
143.24
130.63
129.53
128.61
120.12
119.66
112.85
105.98
77.41 CDCl3
77.16 CDCl3
76.91 CDCl3
55.47
47.63
46.88
43.42
CF3
N
N
N
N
N
MeO
38
(13C NMR , CDCl3, 126 MHz)
f1 (ppm)

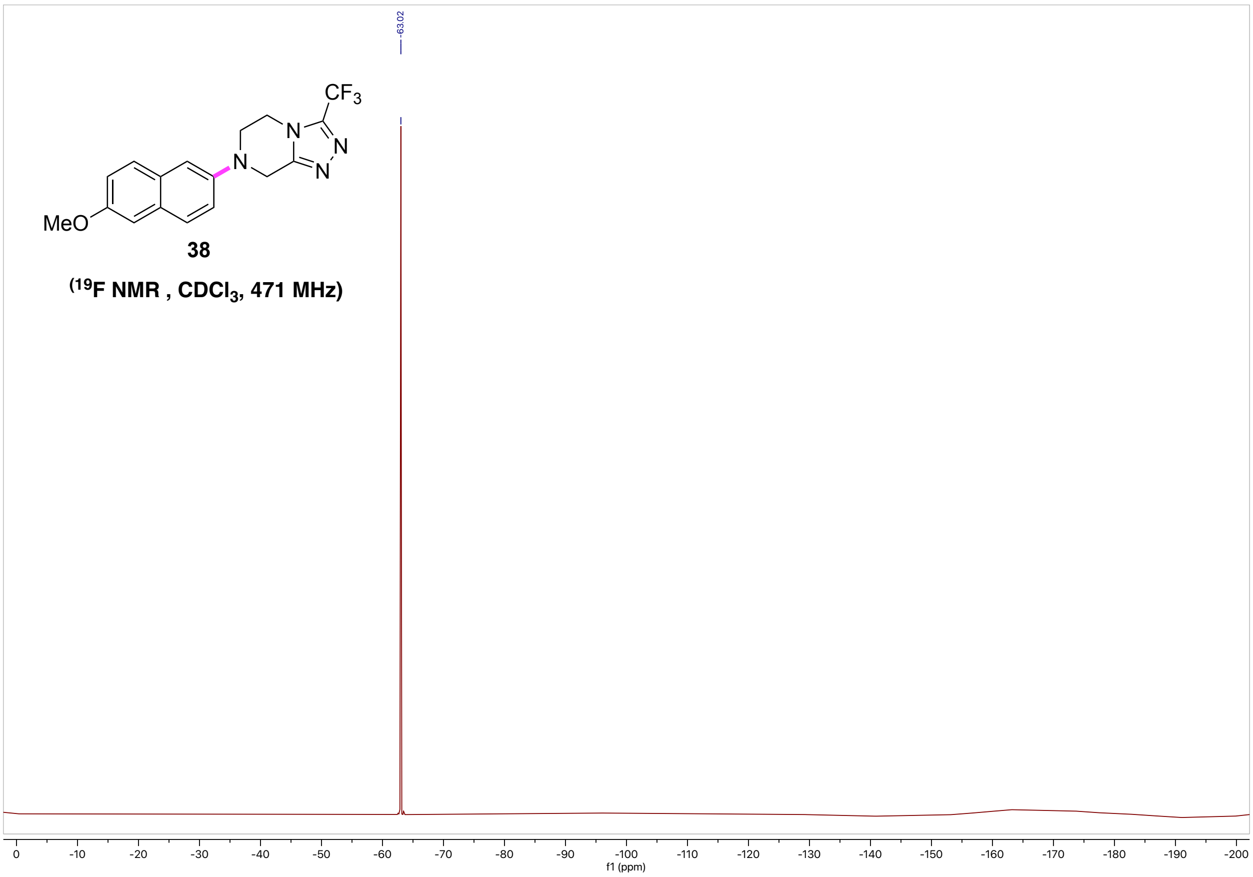
-63.02
CF3
N
N
N
N
MeO
38
(19F NMR , CDCl3, 471 MHz)
0
-10
-20
-30
-40
-50
-60
-70
-80
-90
-100
-110
-120
-130
-140
-150
-160
-170
-180
-190
-200
f1 (ppm)

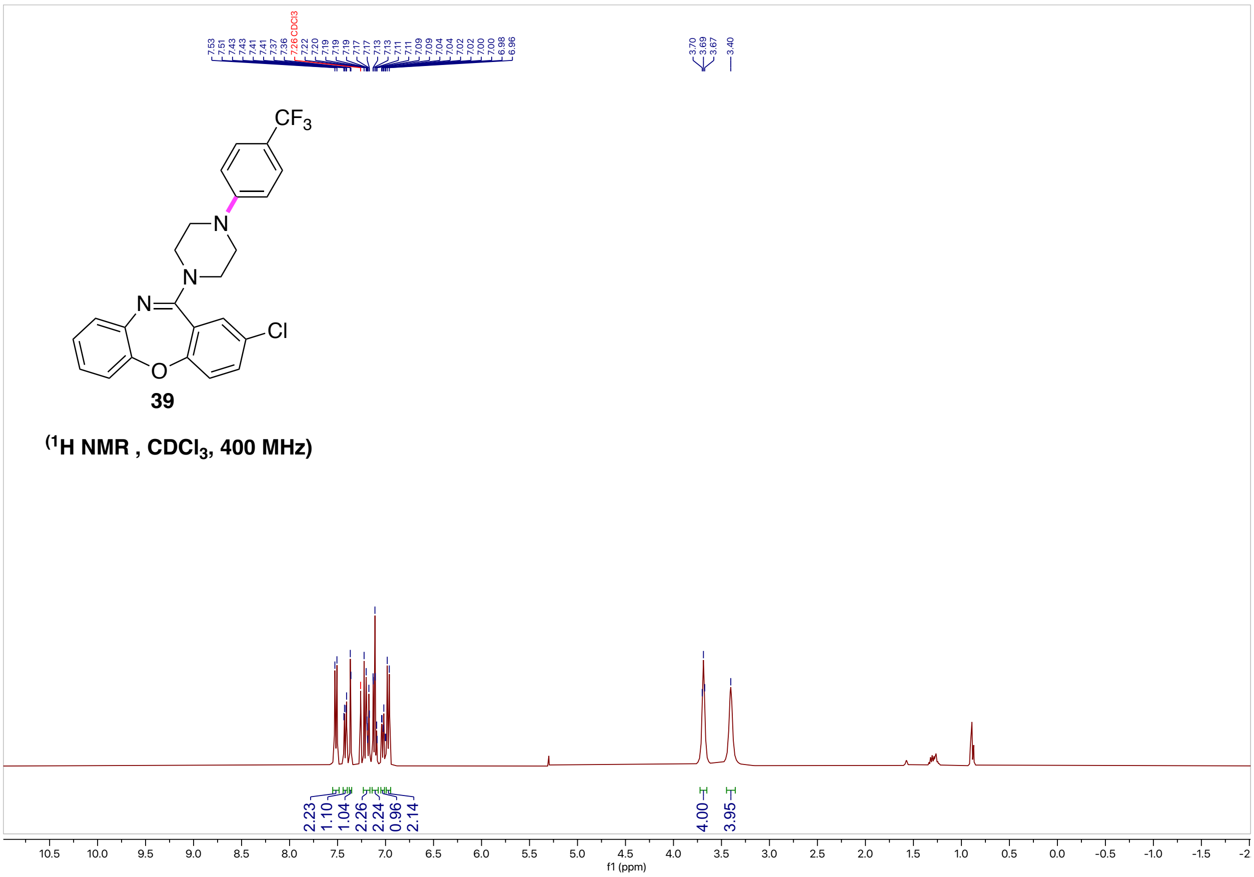

CF3
N
N
N
Cl
O
39
(1H NMR , CDCl3, 400 MHz)
f1 (ppm)

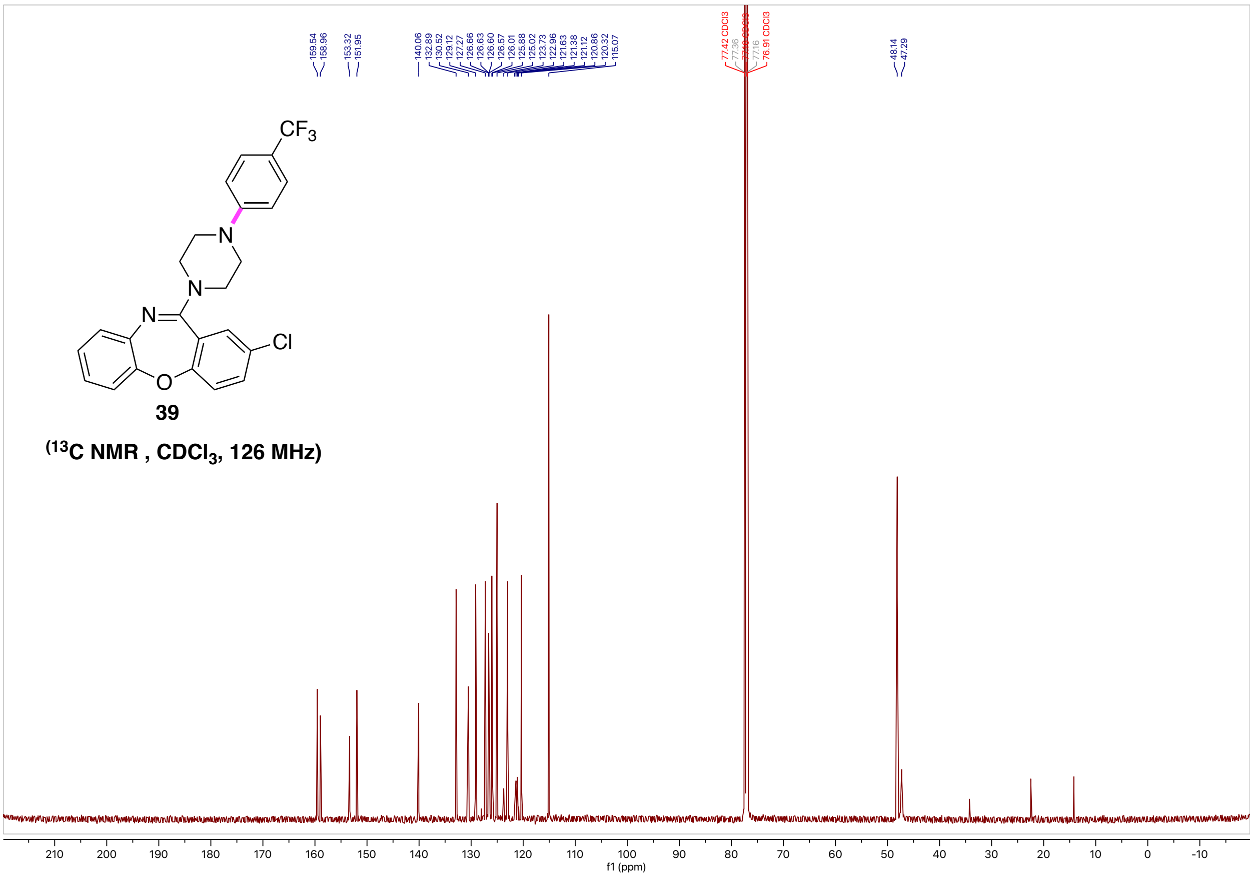

CF3
N
N
N
Cl
O
39
(13C NMR , CDCl3, 126 MHz)
f1 (ppm)

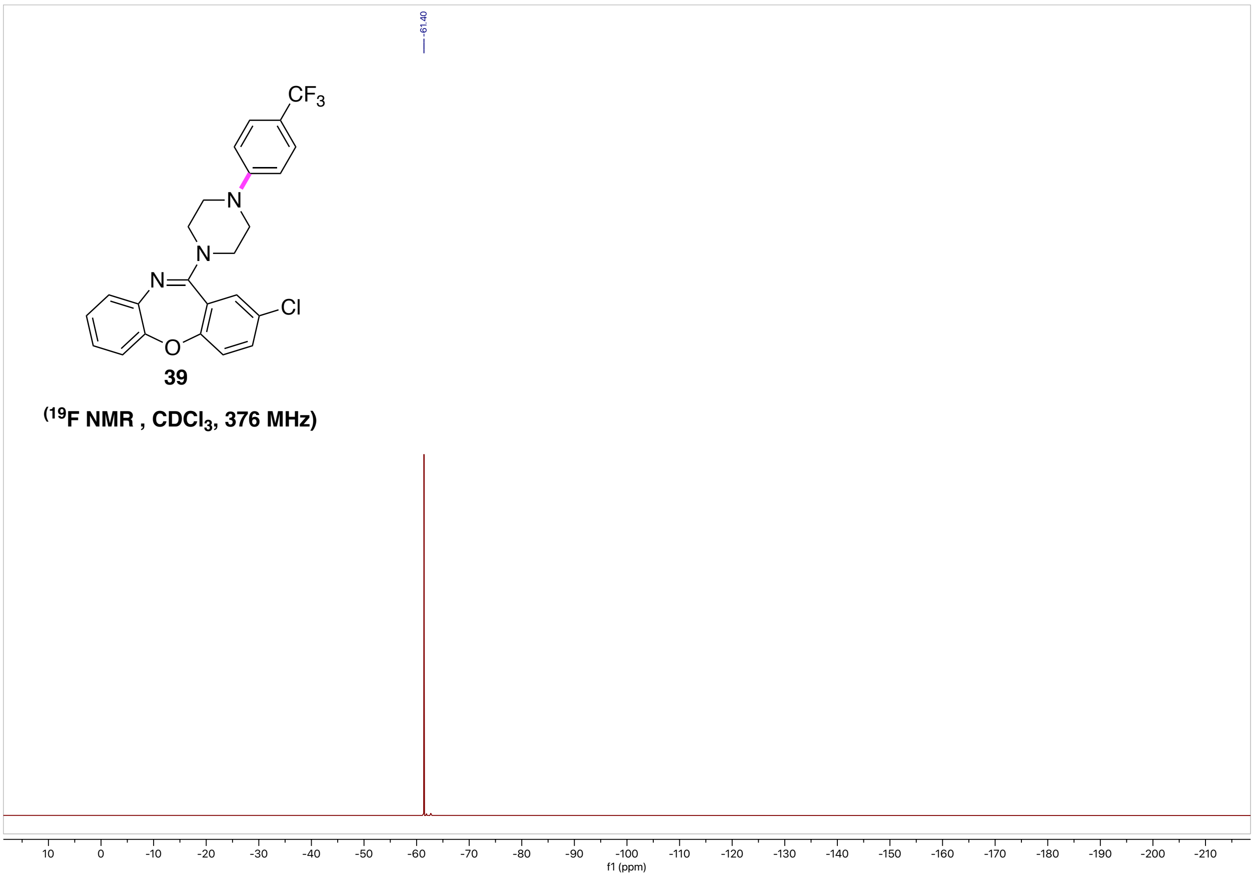
-61.40
CF3
N
N
N
Cl
O
39
(19F NMR , CDCl3, 376 MHz)
10 0 -10 -20 -30 -40 -50 -60 -70 -80 -90 -100 -110 -120 -130 -140 -150 -160 -170 -180 -190 -200 -210
f1 (ppm)

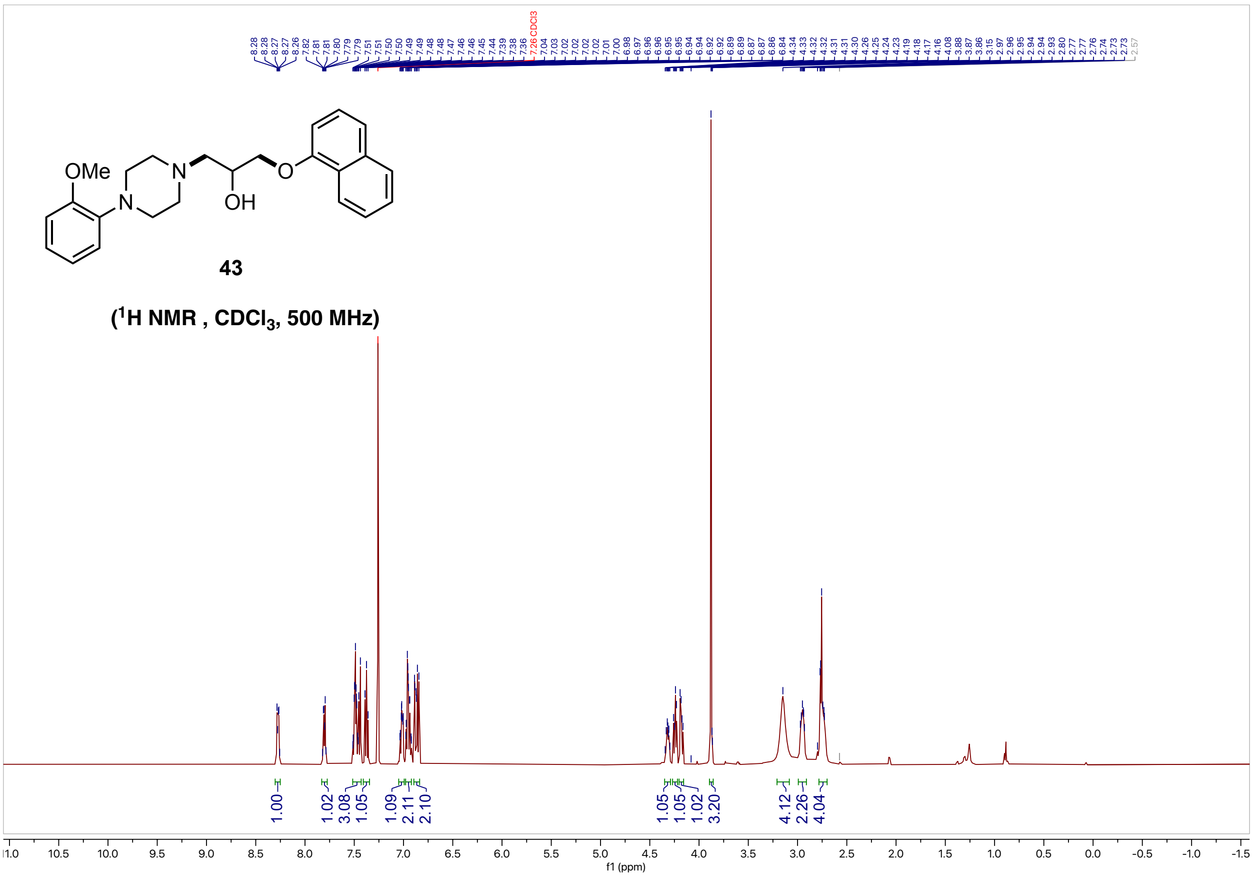

OMe
N
N
OH
O
43
(1H NMR , CDCl3, 500 MHz)
f1 (ppm)

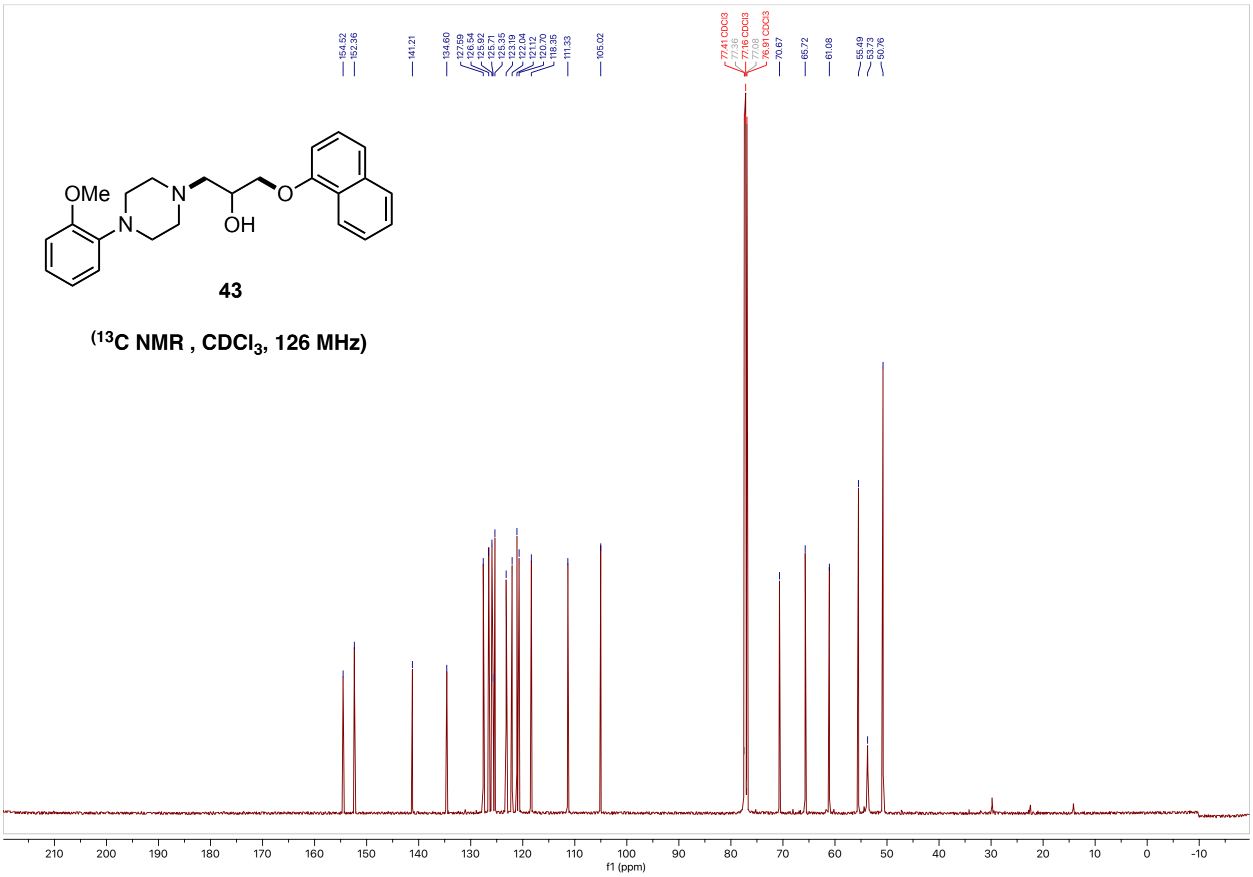

OMe
N
N
OH
O
43
(13C NMR , CDCl3, 126 MHz)
154.52
152.36
141.21
134.60
105.02
70.67
65.72
61.08
55.49
53.73
50.76
f1 (ppm)
